# Synthesis of 3,3-Disubstituted Thietane Dioxides

**DOI:** 10.1021/acs.joc.4c01843

**Published:** 2024-10-11

**Authors:** Peerawat Saejong, Jianing Zhong, Juan J. Rojas, Andrew J. P. White, Chulho Choi, James A. Bull

**Affiliations:** † Department of Chemistry, Imperial College London, Molecular Sciences Research Hub, White City Campus, Wood Lane, London W12 0BZ, U.K.; ‡ Medicine Design, Pfizer Research and Development, Groton, Connecticut 06340, United States

## Abstract

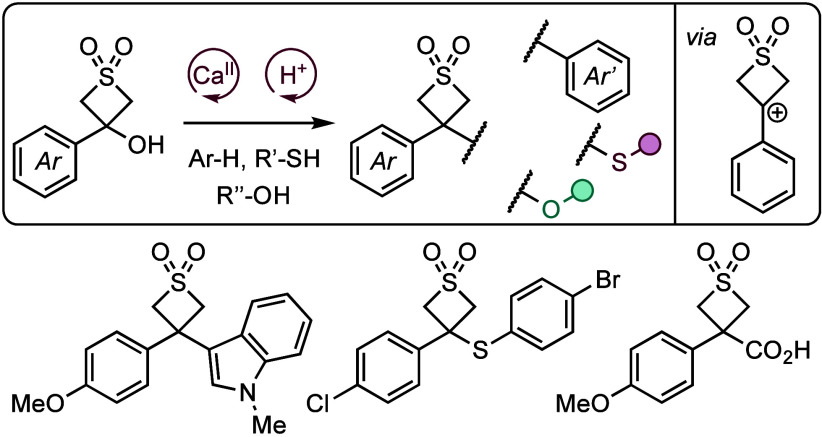

4-Membered heterocycles have been increasingly exploited in medicinal chemistry and, as small polar motifs, often show important influence on activity and physicochemical properties. Thietane dioxides similarly offer potential in both agricultural and pharmaceutical applications but are notably understudied. Here we report a divergent approach to 3,3-disubstituted thietane dioxide derivatives by forming carbocations on the 4-membered ring with catalytic Lewis or Brønsted acids. Benzylic tertiary alcohols of the thietane dioxides are coupled directly with arenes, thiols, and alcohols.

## Introduction

**Figure 1:**
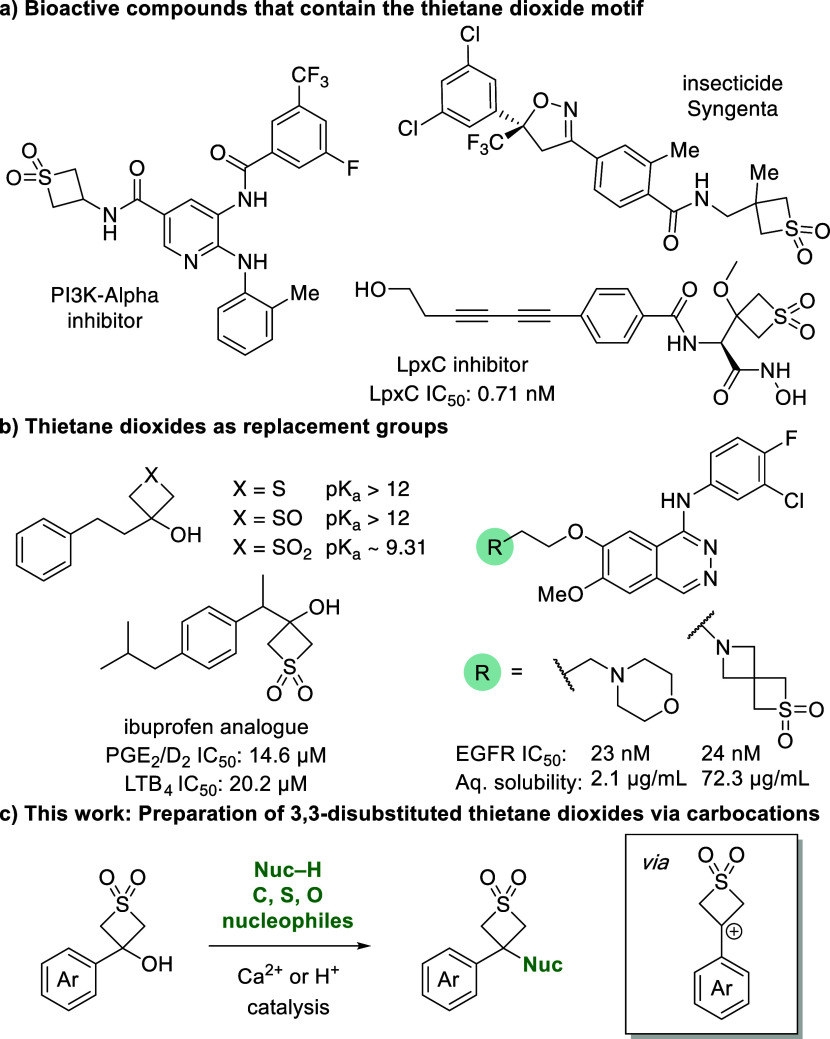
a) Examples of thietane dioxides in medicinal chemistry and agricultural applications. b) Thietane dioxides as replacement groups. c) This work: synthesis of 3,3-disubstituted thietane dioxide derivatives.

4-Membered heterocycles have been of notable interest in medicinal chemistry due to the potential to provide attractive polar and 3-dimensional motifs of low molecular weight and high H-bonding potential.^1^ Recent years have seen extensive development of the applications of oxetanes and azetidines.^2^ On the other hand, thietanes and their oxidized forms are much less studied and as such present interesting opportunities for development.^3^ Thietane dioxides in particular present interesting potential, being stable to further oxidation, that has been little exploited. The thietane dioxides may be considered expanded sulfones, though the oxygen atoms are rotated by 90° in the thietane dioxides, in plane with the substituents at the 3-position. Compounds bearing thietane dioxides have been reported in biologically active compounds in medicinal and agrochemistry (Figure 1). Recently a PI3K-Alpha inhibitor containing a thietane dioxide was reported as a potential cancer therapeutic.^4^ LpxC inhibitors containing thietane dioxides were disclosed as potential antibacterial agents, whereby a cocrystal with the enzyme displayed H-bonding with a lysine side chain, benefiting from the expanded size of the thietane dioxide compared to a methyl sulfone.^5^ Syngenta patented a series compounds containing pendant thietane dioxides as insecticides.^6^ Preliminary investigations have also studied thietane dioxide derivatives as replacements for carbonyl groups in carboxylic acids which maintained some acidity in comparison to oxetanols,^7^ including in ibuprofen analogues, and in spirocyclic morpholine analogues as solubilizing motifs (Figure 1b).^8^

We have been interested in the synthesis of 3,3-disubstituted aryl-oxetanes and azetidines through the catalytic generation of carbocationic intermediates. The use of Lewis (Li^+^, Ca^2+^, and Fe^3+^ salts) and Brønsted acid catalysts has proven useful in selectively activating the 4-membered ring benzylic tertiary alcohols for Friedel–Crafts alkylation,^9^ and alkylation of thiols^10^ and alcohols.^11,12^ We envisaged that a similar approach may be viable on thietane dioxides, and as such provide a facile route to 3,3-disubstituted thietane dioxides, exploiting thietane-3-one as a readily available precursor.^13^ Here we report the development of a calcium-catalyzed reaction of 3-aryl-thietan-3-ol dioxides with arene and thiol nucleophiles, and a Brønsted acid catalyzed reaction with alcohols. This strategy provides arylthietane dioxide derivatives in a short divergent route expanding the available chemical space of 4-membered heterocycles. The 3,3-disubstituted products display high chemical stability and potential for further diversification.

## Results and Discussion

**Scheme 1:**
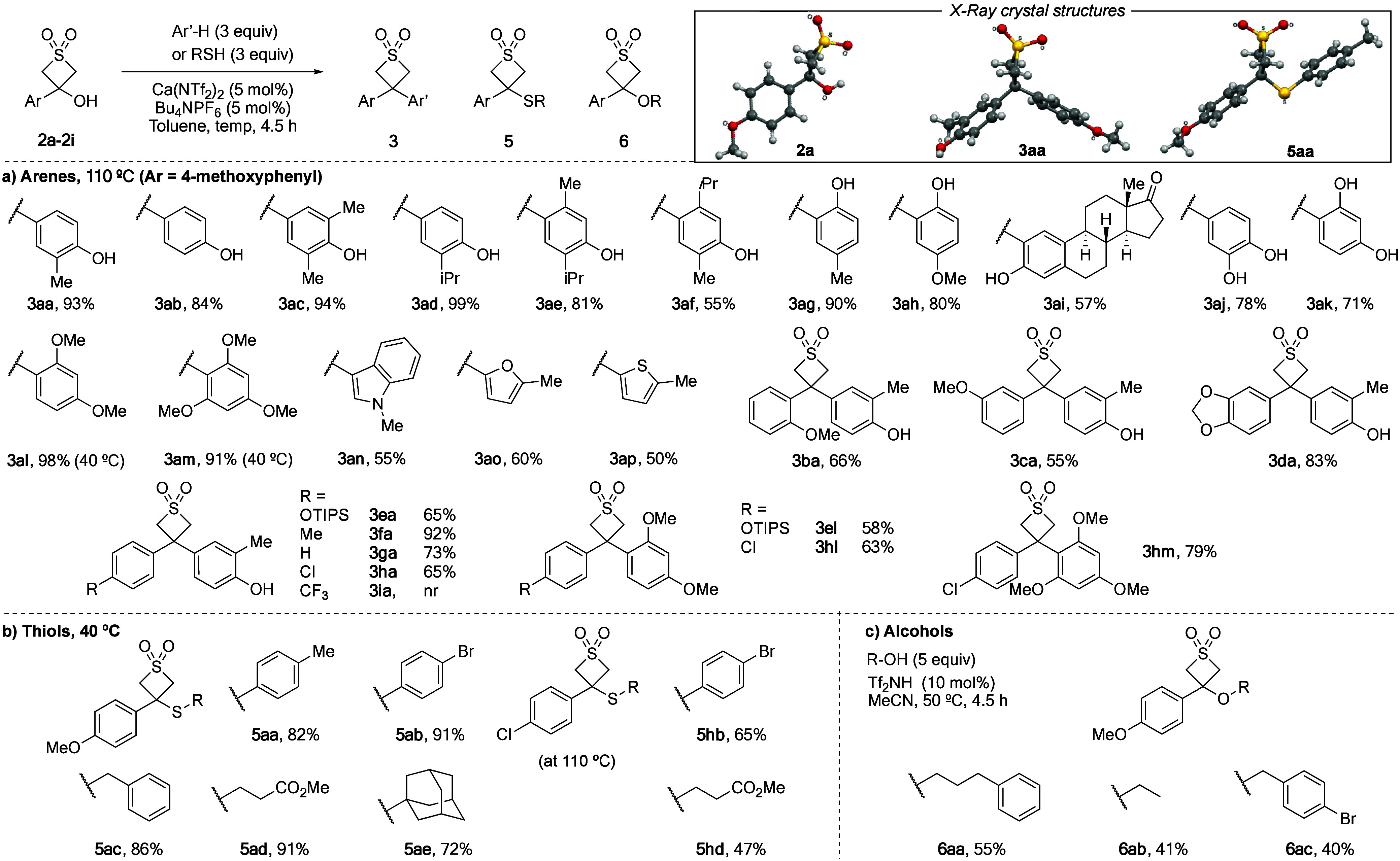
Reaction Scope with Arene, Thiol, and Alcohol Nucleophiles

The study started from thietane-3-one, a readily available inexpensive precursor that reacts as a typical ketone with Grignard or organolithium reagents for the preparation of thietanols **1**,^14^ which were readily converted to thietane dioxides **2** by oxidation with *m*CPBA (Table 1). Initial studies to generate the carbocation used thietanol dioxide **2a**, which was readily prepared by the addition of commercial 4-methoxyphenylmagnesium bromide solution to thietane-3-one on >5 g scale. Based on our previous conditions with oxetanols and azetidinols,^9a^ we then surveyed Lewis acids and Brønsted acids for the dehydrative generation of the benzylic carbocation on the thietane dioxide to trap with arene nucleophiles. Treating alcohol **2a** with lithium salts in the presence of *o*-cresol under conditions successful for oxetanes formed the diarylthietane dioxide **3aa** in low yield (Table 1 entry 1). A similar quantity of stable 3-aryl-2*H*-thiete 1,1-dioxide **4a** was also formed, presumably through E1 elimination from the carbocation intermediate. On the other hand, Ca^2+^, Fe^3+^, and H^+^ catalysts all gave full conversion of the substrate and good yields of the diarylthietane dioxide product, but still with significant amounts of the elimination product **4a** (entries 2–4). The solvent could be changed from dichloromethane to toluene as a more acceptable solvent for use on scale with similar results (entry 5, with Ca^II^ catalyst). A preliminary reaction scope was then examined using the Ca-catalyst due to ease of handling, but less reactive substrates required elevated temperatures to initiate a reaction. Therefore, we reexamined higher temperature conditions on the model substrate **2a**. Pleasingly the thietane dioxide derivatives displayed full stability under elevated temperatures in toluene and moreover gave a notable increase in yield and decrease in formation of the thiete dioxide side product. Using 110 °C provided quantitative conversion, and a 93% isolated yield of **3aa** (entry 7 and Scheme 1).

**Table 1:**
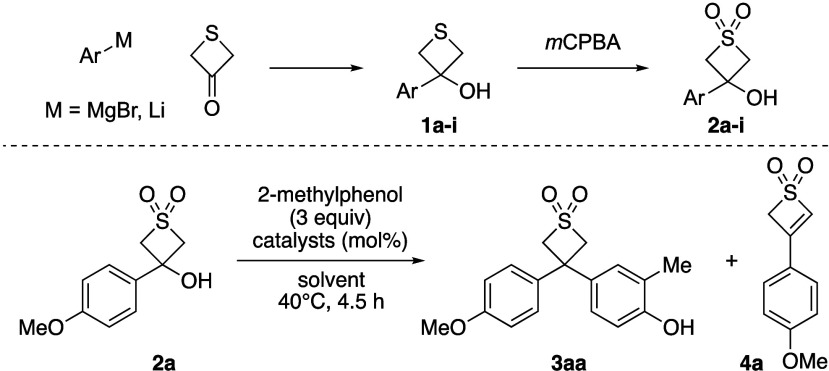
Selected Optimization for Friedel–Crafts Reaction from Thietanol-Dioxide **2a** and *o*-Cresol

			yield (%)^b^	
entry^a^	catalyst (mol %)	solvent	**3aa**	**4a**
1	Li(NTf_2_) (11) + *n*Bu_4_NPF_6_ (5.5)	CH_2_Cl_2_	24	17
2	FeCl_3_ (5)	CH_2_Cl_2_	74	12
3	Ca(NTf_2_)_2_ (5) + *n*Bu_4_NPF_6_ (5)	CH_2_Cl_2_	75	21
4	HNTf_2_ (10)	CH_2_Cl_2_	82	18
5	Ca(NTf_2_)_2_ (5) + *n*Bu_4_NPF_6_ (5)	toluene	63	37
6^c^	Ca(NTf_2_)_2_ (5) + *n*Bu_4_NPF_6_ (5)	toluene	72	23
7^d^	Ca(NTf_2_)_2_ (5) + *n*Bu_4_NPF_6_ (5)	toluene	97	0

a Reactions on a 0.20 mmol scale.

b Yields calculated by analysis of the ^1^H NMR spectrum of the crude reaction mixture using 1,3,5-trimethoxybenzene as internal standard.

c Reaction run at 60 °C.

d Reaction run at 110 °C.

With high yielding conditions in hand, we examined the scope of the Friedel–Crafts reaction, through the variation of arene nucleophiles and thietane dioxide substrates (Scheme 1). Phenols were successful with complete C4 regioselectivity when that position was unsubstituted (**3aa**–**3ak**). Phenol itself gave diaryl thietane dioxide **3ab** in 84% yield on a 1 mmol scale. Substituents at C3 were tolerated, including larger isopropyl groups, with little reduction in yield (**3ae**, **3af**). 4-Substituted phenols were alkylated at C2 in high yields, including with cholesterol as a nucleophile (**3ag**–**3ai**). Catechol and resorcinol nucleophiles were also successful and yielded single regioisomers (**3aj**, **3ak**). In contrast to previous observations with oxetanes,^9a,9d^ there was no indication of opening of the thietane dioxide ring by *ortho*-hydroxyl groups. Nonphenolic arenes di- and trimethoxybenzene reacted effectively at 40 °C, with 98%, and 91% isolated yields (**3al**, **3am**), without elimination to the thiete dioxide. Heterocycle nucleophiles *N*-methylindole, 2-methylfuran and 2-methylthiophene were also successful (**3an–3ap**). Electron-poor arenes like 4-bromophenol were unsuccessful, yielding only the elimination product **4a**. Aniline nucleophiles were unsuccessful and returned unreacted **2a**.

Varied substitution patterns were well tolerated on the arene of the thietanol dioxide (**3ba**–**3hm**). Methoxy substituents were tolerated in *ortho*- and *meta*-positions as well as a benzodioxole ring (**3ba**–**3da**). Substituents in the *para* position were also well tolerated, including an OTIPS group without any observed deprotection (**3ea**, **3el**), electron neutral tolyl and phenyl derivatives (**3fa**, **3ga**), and electron withdrawing 4-chlorophenyl derivative (**3ha**, **3hl**). A *para*-CF_3_ derivative was unsuccessful, with recovered starting materials even under thermal activation up to 180 °C in dichlorobenzene. Attempts to extend the process to thietane and thietane oxide substrates were also unsuccessful, resulting in degradation, likely due to the transannular involvement of the sulfur lone pairs, which is not possible with the thietane dioxides. We propose thietane dioxides form a planar carbocation intermediate, analogous to that described for oxetanes.^15^

Next, we extended the reaction to thiol nucleophiles, to form 3-sulfanyl thietane dioxides (Scheme 1b). Both aromatic and aliphatic thiols were well tolerated in the alkylation with thietanol dioxide **2a**, and the reaction proceeded smoothly at 40 °C (**5aa**–**5ae**). Alkylation using the less electron-rich thietanol dioxide **2h** was also successful (**5hb**, **5hd**) but required higher thermal activation (110 °C). Although the direct application of the reaction conditions to alcohol nucleophiles was unsuccessful, changing to Brønsted acid catalysis (Tf_2_NH, 10 mol %) in MeCN achieved the *O*-alkylation of primary and benzylic alcohols (**6aa**–**6ac**). Secondary alcohols were not tolerated due to a reversible C–O bond formation but irreversible elimination step funnelling the material to thiete dioxide **4a**.

Several derivatives were further characterized by X-ray diffraction analysis of single crystals (**2a**, **3aa**, **3ac**, and **5aa**, Scheme 1 boxed). Thietanol **2a** showed a puckered thietane dioxide ring (29.4°) toward the hydroxyl group, suggestive of an intramolecular H-bond. On the other hand, diarylthietane dioxides were less puckered (**3aa** 14.0°; **3ac** 16.9°) and the toluene sulfide derivative **5aa** displayed a planar thietane dioxide ring (1° puckering angle). The dihedral conformation about the thietane-C–S bond is such that the tolyl group is aligned to the thietane S=O.

**Scheme 2:**
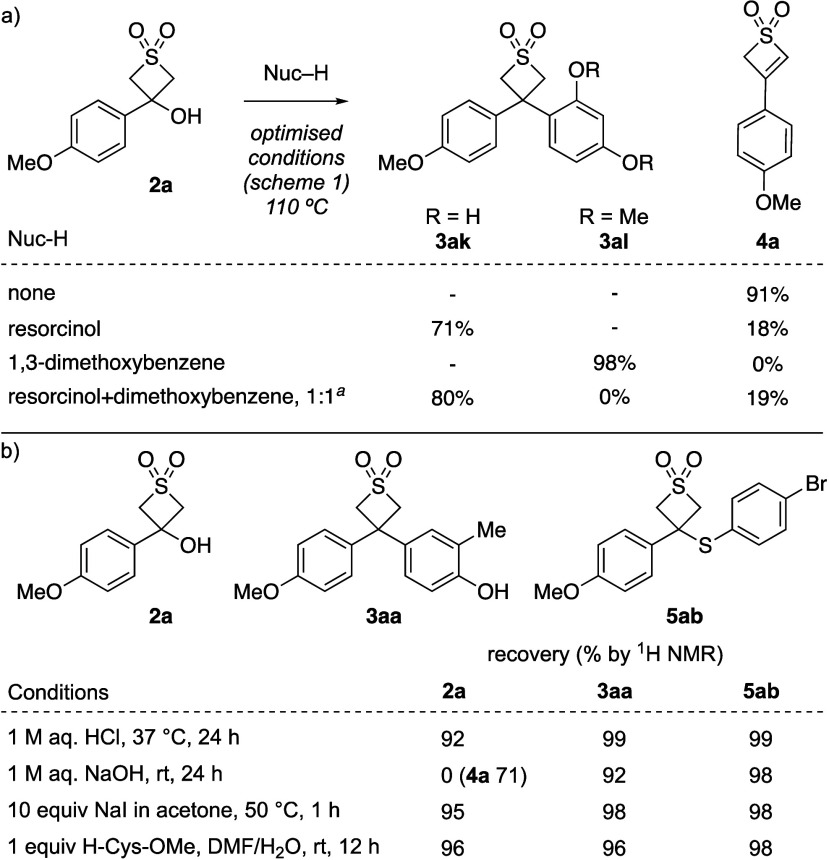
Competition experiments and stability studies yield by ^1^H NMR for competition experiment.

To better understand the effect of different nucleophiles and temperature on the reaction, a series of control experiments was performed. In the absence of a nucleophile, the thiete dioxide product formed through elimination was isolated in high yield (91%, Scheme 2a). Thiete dioxides have themselves have been demonstrated as suitable substrates for further reactions including cycloaddition, metalation and C–H functionalization.^14^ The effect of phenolic nucleophiles on elimination were investigated in a competition experiment. Both resorcinol and dimethoxybenzene undergo Friedel–Crafts alkylation with thietanol **2a** in high yield, but **4a** is formed only with resorcinol. A competition experiment with a 1:1 mixture of resorcinol and dimethoxybenzene gave only the phenolic diaryl product **3ak**, but also formed thiete **4a**, suggestive of a noninnocent role of the phenolic hydroxyl groups in the elimination process (Scheme 2a). Treating **2a** with resorcinol alone does not result in elimination, suggesting a role as a basic site, perhaps via an O-linked intermediate.^9b^ Resubmitting thiete **4a** to the optimized reaction conditions with dimethoxybenzene gave only recovered **4a**. On the other hand, treating **4a** with resorcinol under the optimized conditions formed **3ak** in a high 88% yield. Subjecting **3ak** to the reaction conditions in the presence of dimethoxybenzene gave no reaction suggesting the reaction is not reversible. Together, this suggests a more complex role for the phenol nucleophiles to both promote the elimination pathway but also return the thiete dioxide to the catalytic cyclic through protonation. Indeed, reacting thiete **4a** with o-cresol gave **3aa** in low yield 37% (by ^1^H NMR). This explains the beneficial effect of the higher reaction temperature as the carbocation can be regenerated from the side product **4a** in the presence of the acidic nucleophile.

The chemical stability of 3,3-disubstituted thietane-1,1-dioxides was investigated by submitting thietan-3-ol **2a**, diarylthietane dioxide **3aa**, and sulfide **5ab** to a range of conditions (Scheme 2b). In general, quantitative recovery of the substrates was observed across acidic (1 M HCl at 37 °C) and basic conditions (1 M NaOH), as well as in the presence of nucleophiles (NaI, and cysteine methyl ester). On treatment with aqueous 1 M NaOH, thietan-3-ol dioxide **2a** degraded via elimination to thiete **4a**.

**Scheme 3:**
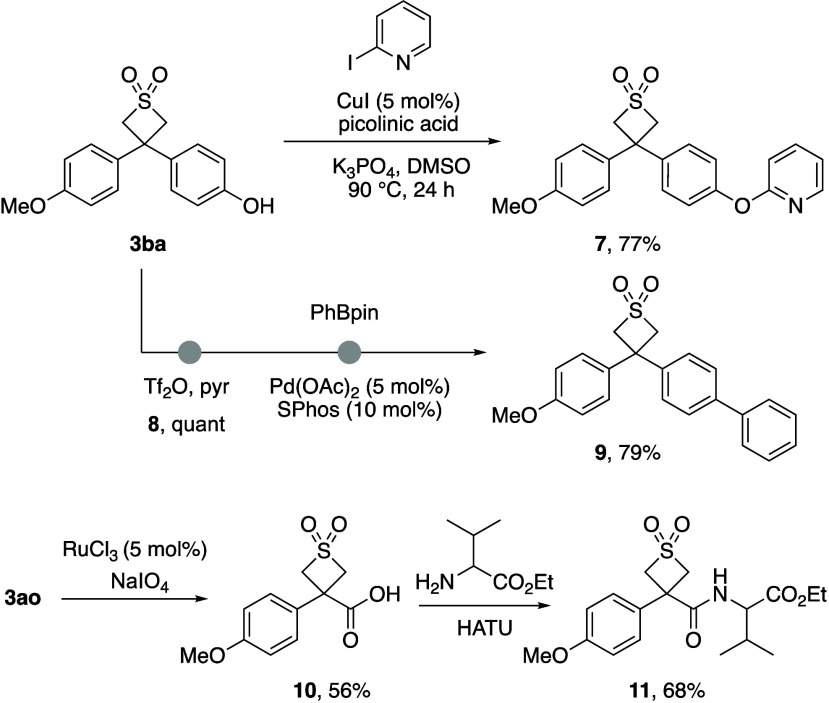
Further functionalisation of 3,3-diarylthietane dioxides

The phenolic functionality provides a handle for further functionalization through cross-coupling processes which was demonstrated with transition metal catalysis (Scheme 3). Ullmann arylation of **3ba** with iodopyridine gave ether **7**. Triflation was achieved in quantitative yield, which allowed for Suzuki–Miyaura coupling to form biaryl derivative **9**. Carboxylic acid derivative **10** was prepared from furan **3ao** by selective oxidative cleavage using ruthenium catalysis.^16^ The acid was readily applied in amide bond formation with standard conditions to give amide **11**.

Overall, we present protocols for the preparation of 3,3-substituted thietane dioxides through the formation of carbocation intermediates. The application of increased temperatures was important to minimize formation of a thiete dioxide. We expect thietane dioxides to see broader application in medicinal chemistry with the development of new methods for their preparation, and the increase in commercial availability. This methodology provides a rapid and divergent approach to these disubstituted derivatives, and form C–C, C–S, and C–O bonds directly onto the intact 4-membered ring. The thietane dioxide rings display high chemical stability and are suitable for application in further cross-coupling and derivatization reactions.

## Experimental Section

### General Experimental Considerations

All nonaqueous reactions were run under an inert atmosphere (argon) with flame-dried or oven-dried glassware using standard techniques. Anhydrous solvents were obtained by filtration through drying columns (toluene, CH_2_Cl_2_) or used directly from commercial sources (MeCN) without drying. Reactions that required thermal activation were heated using a water bath (for temperatures up to 25 °C) or a silicone oil bath (for temperatures >25 °C). Flash column chromatography was performed using 230–400 mesh silica with the indicated solvent system according to standard techniques. Analytical thin-layer chromatography (TLC) was performed on precoated, glassbacked silica gel plates. Visualization of the developed chromatogram was performed by UV absorbance (254 nm) or aqueous potassium permanganate stains. Infrared spectra (ν_max_, FTIR ATR) were recorded in reciprocal centimeters (cm^–1^). Nuclear magnetic resonance spectra were recorded on 400 MHz spectrometers. Chemical shifts for ^1^H NMR spectra are recorded in parts per million from tetramethylsilane with the residual protic solvent resonance as the internal standard (chloroform: δ = 7.27 ppm, (CD_3_)_2_SO: δ = 2.50 ppm, CD_3_OD: δ = 3.31 ppm, acetone-*d*_6_: δ = 2.05 ppm). Data is reported as follows: chemical shift [multiplicity (s = singlet, d = doublet, t = triplet, quartet = q, pentet = p, m = multiplet and br = broad), coupling constant in Hz, integration, assignment]. ^13^C NMR spectra were recorded with complete proton decoupling. Chemical shifts are reported in parts per million from tetramethylsilane with the solvent resonance as the internal standard (^13^CDCl_3_: δ = 77.0 ppm, (^13^CD_3_)_2_SO: δ = 39.5 ppm, ^13^CD_3_OD: δ = 49.0 ppm, (^13^CD_3_)_2_O: δ = 29.8 ppm). *J* values are reported in Hz. Assignments of ^1^H/^13^C spectra were made by the analysis of δ/*J* values, and HSQC experiments as appropriate. ^19^F NMR spectra are indirectly referenced to CFCl_3_ automatically by direct measurement of the absolute frequency of the deuterium lock signal by the spectrometer hardware. Melting points were recorded using an Optimelt MPA100 apparatus and are uncorrected. The high-resolution mass spectrometry (HRMS) analyses were performed using electrospray ion source (ESI) or pneumatically assisted atmospheric pressure chemical ionization (APCI) using an atmospheric solids analysis probe (ASAP). ESI was performed using a Waters LCT Premier equipped with an ESI source operated in positive or negative ion mode. The software used was MassLynx 4.1. This software does not account for the electron and all the calibrations/references are calculated accordingly, i.e. [M + H]^+^ is detected and the mass is calibrated to output [M + H]. APCI was performed using an Orbitrap XL or Xevo G2S using an ASAP to insert samples into the APCI source. The sample was introduced at ambient temperature and the temperature increased until the sample vaporized. In mass spectrometry for thietan-3-ols, in some instances the ionization method fragmented the substrate to generate a carbocation, whereby [M – OH]^+^ was often found instead of [M + H]^+^.

Reagents: Commercial reagents were used as supplied or purified by standard techniques where necessary. Trifluoromethanesulfonimide (Tf_2_NH) was purchased from Fluorochem (CAS: 82113-65-3, product code: 093934), stored under argon in the fridge (+4 °C) and used without further purification. Calcium(II) bis(trifluoromethanesulfonimide) (Ca(NTf_2_)_2_) was purchased from Tokyo Chemical Industry (TCI) (CAS: 165324-09-4, product code: C3263), stored under argon in the desiccator. The concentration of *n*-BuLi (1.6 M in hexanes, purchased from Sigma-Aldrich, CAS: 109-72-8) was determined by titration with salicylaldehyde phenylhydrazone as an indicator before each reaction using a literature procedure.^17^ An average of three titrations was taken.

### Synthesis of Thietanols from Thetan-3-one

#### 3-(4-Methoxyphenyl)thietan-3-ol (**1a**)

4-Methoxyphenyl magnesium bromide (0.5 M in THF, 100 mL, 50.0 mmol, 1.1 equiv) was added dropwise to a solution of thietane-3-one (4.01 g, 45.5 mmol, 1.0 equiv) in THF (141 mL, 0.24 M) at −78 °C. After stirring at −78 °C for 30 min, the reaction mixture was warmed up to 25 °C and stirred for 1 h. The reaction was then quenched with sat. NH_4_Cl (80 mL). The mixture was extracted with CH_2_Cl_2_ (3 × 50 mL). The combined organic layers were dried over Na_2_SO_4_, filtered, and concentrated in vacuo using a rotary evaporator. Purification by flash column chromatography (5–10% EtOAc/pentane) afforded 3-(4-methoxyphenyl)thietan-3-ol **1a** as yellow oil (5.40 g, 71%). R_f_ = 0.30 (25% EtOAc/pentane); IR (film)/cm^–1^ 3401 (OH), 2994, 2935, 2833, 1610, 1580, 1511, 1462, 1441, 1362, 1301, 1249, 1211, 1178, 1108, 1032, 956, 830, 658, 579, 551; ^1^H NMR (400 MHz, CDCl_3_) δ 7.58 (d, *J* = 8.8 Hz, 2H, 2 × Ar–H), 6.94 (d, *J* = 8.8 Hz, 2H, 2 × Ar–H), 3.84 (s, 3H, OCH_3_), 3.65 (d, *J* = 10.4 Hz, 2H, C*H*H–S–C*H*H), 3.59 (d, *J* = 10.4 Hz, 2H, CH*H*–S–CH*H*), 2.77 (s, 1H, OH); ^13^C{^1^H} NMR (101 MHz, CDCl_3_) δ 159.3 (Ar–C_q_OMe), 136.7 (Ar–C_q_), 125.6 (2 × Ar–C), 113.9 (2 × Ar–C), 78.9 (C_q_), 55.3 (OCH_3_), 42.6 (CH_2_SCH_2_); HRMS (ESI) *m*/*z* calculated for C_10_H_11_OS [M – OH]^+^: 179.0531, found: 179.0536. The observed characterization data (IR, ^1^H and ^13^C NMR) were consistent with that previously reported.^14^

#### 3-(2-Methoxyphenyl)thietan-3-ol (**1b**)

*i*PrMgCl·LiCl (1.30 M in THF, 2.54 mL, 3.3 mmol, 1.1 equiv) was added dropwise over 5 min to a solution of 2-iodoanisole (0.45 mL, 3.6 mmol, 1.2 equiv) in THF (4.0 mL) at 0 °C. The reaction mixture was stirred at 0 °C for a further 10 min and warmed to 25 °C for 2 h. A solution of thietanone (264 mg, 3.0 mmol, 1.0 equiv) in THF (6.0 mL) was added dropwise to the reaction mixture at −78 °C, then leave them to stir for 1 h. Following a further 24 h at 25 °C the reaction mixture was cooled to 0 °C and then quenched with sat. aq. NH_4_Cl (25 mL). The aqueous portion was extracted with Et_2_O (3 × 25 mL). The organic extracts were combined, dried over Na_2_SO_4_, filtered and concentrated under reduced pressure. Purification by flash chromatography (70% Et_2_O/pentane) afforded 3-(2-methoxyphenyl)thietan-3-ol **1b** as yellow oil (412 mg, 70%). R_f_ = 0.31 (30% EtOAc/hexane); IR (film)/cm^–1^ 3429 (OH), 2938, 2833, 1599, 1489, 1459, 1434, 1353, 1289,1233, 1177, 1020, 747, 577; ^1^H NMR (400 MHz, CDCl_3_) δ 7.46 (dd, *J* = 7.7, 1.7 Hz, 1H, Ar–H), 7.33 (td, *J* = 7.8, 1.6 Hz, 1H, Ar–H), 7.04 (td, *J* = 7.5, 1.1 Hz, 1H, Ar–H), 6.96 (d, *J* = 8.2 Hz, 1H, Ar–H), 4.24 (s, 1H, OH), 3.90 (s, 3H, OCH_3_), 3.66 (d, *J* = 10.1 Hz, 2H, C*H*H–S–C*H*H), 3.62 (d, *J* = 10.1 Hz, 2H, CH*H*–S–CH*H*); ^13^C{^1^H} NMR (101 MHz, CDCl_3_) δ 156.7 (Ar–C_q_), 130.8 (Ar–C_q_), 129.3 (Ar–C), 125.5 (Ar–C), 120.9 (Ar–C), 111.2 (Ar–C), 78.9 (C_q_), 55.3 (O–CH_3_), 40.2 (2 × S–CH_2_).

#### 3-(3-Methoxyphenyl)thietan-3-ol (**1c**)

3-Methoxyphenyl magnesium bromide (1.0 M in THF, 11 mL, 11 mmol, 1.1 equiv) was added dropwise to a solution of thietane-3-one (881 g, 10 mmol, 1.0 equiv) in THF (10 mL, 0.24 M) at −78 °C. After stirring at −78 °C for 30 min, the reaction mixture was warmed up to 25 °C and stirred for 3 h. The reaction was then quenched with sat. NH_4_Cl (50 mL). The mixture was extracted with CH_2_Cl_2_ (3 × 30 mL). The combined organic layers were dried over Na_2_SO_4_, filtered, and concentrated in vacuo using a rotary evaporator. Purification by flash column chromatography (10% EtOAc/pentane) afforded 3-(3-methoxyphenyl)thietan-3-ol **1c** as yellow oil (1.14 mg, 58%). R_f_ = 0.18 (20% EtOAc/pentane); IR (film)/cm^–1^ 3398 (OH), 2936, 2832, 1771, 1582, 1485, 1427, 1287, 1211, 1171, 1036, 964, 842, 781, 692, 564, 474; ^1^H NMR (400 MHz, CDCl_3_) δ 7.35 (d, *J* = 8.2 Hz, 1H, Ar–H), 7.26 (s, 1H, Ar–H), 7.20 (t, *J* = 2.2 Hz, 1H, Ar–H), 6.88 (dd, *J* = 8.2, 2.2 Hz, 1H, Ar–H), 3.84 (s, 3H, OCH_3_), 3.65 (d, *J* = 10.1 Hz, 2H, C*H*H–S–C*H*H), 3.56 (d, *J* = 10.1 Hz, 2H, CH*H*–S–CH*H*); ^13^C{^1^H} NMR (101 MHz, CDCl_3_) δ 159.9 (Ar–C_q_OMe), 146.2 (Ar–C_q_), 129.8 (Ar–C), 116.5 (Ar–C), 113.3 (Ar–C), 110.2 (Ar–C), 79.1 (C_q_), 55.4 (OCH_3_), 42.4 (2 × CH_2_–SO_2_); HRMS (APCI) *m*/*z* calculated for C_10_H_11_OS [M – OH]^+^: 179.0525, Found: 179.0527.

#### 3-(Benzo[*d*][1,3]dioxol-5-yl)thietan-3-ol (**1d**)

*i*PrMgCl·LiCl (1.30 M in THF, 4.23 mL, 5.5 mmol, 1.1 equiv) was added dropwise over 5 min to a solution of 5-iodo-1,3-benzodioxole (1.49 g, 6.0 mmol, 1.2 equiv) in THF (4.0 mL) at 0 °C. The reaction mixture was stirred at 0 °C for a further 10 min and warmed to 25 °C for 2 h. A solution of thietanone (411 mg, 5.0 mmol, 1.0 equiv) in THF (6.0 mL) was added dropwise to the reaction mixture at −78 °C, then leave them to stir for 1 h. Following a further 24 h at 25 °C the reaction mixture was cooled to 0 °C and then quenched with sat. aq. NH_4_Cl (25 mL). The aqueous portion was extracted with Et_2_O (3 × 25 mL). The organic extracts were combined, dried over Na_2_SO_4_, filtered and concentrated under reduced pressure. Purification by flash chromatography (70% Et_2_O/pentane) afforded 3-(benzo[*d*][1,3]dioxol-5-yl)thietan-3-ol **1d** as yellow oil (410 mg, 39%). R_f_ = 0.32 (30% EtOAc/hexane); IR (film)/cm^–1^ 3370 (OH), 2937, 2889, 1484, 1435, 1233, 1171, 1031, 930, 860, 807, 561, 471; ^1^H NMR (400 MHz, CDCl_3_) δ 7.14 (d, *J* = 1.9 Hz, 1H, Ar–H), 7.11 (dd, *J* = 8.1, 1.9 Hz, 1H, Ar–H), 6.82 (d, *J* = 8.1 Hz, 1H, Ar–H), 5.97 (s, 2H, O–CH_2_–O), 3.61 (d, *J* = 10.5 Hz, 2H, C*H*H–S–C*H*H), 3.54 (d, *J* = 10.4 Hz, 2H, CH*H*–S–CH*H*); ^13^C{^1^H} NMR (101 MHz, CDCl_3_) δ 148.2 (Ar–C_q_), 147.5 (Ar–C_q_), 138.9 (Ar–C_q_), 117.9 (Ar–C), 108.3 (Ar–C), 105.6 (Ar–C), 101.5 (S–CH_2_), 79.4 (C_q_), 42.9 (O–CH_2_); HRMS (APCI) *m*/*z* Calculated for C_10_H_11_O_3_S [M + H]^+^: 211.0423; Found: 211.0422.

#### 3-(4-((Triisopropylsilyl)oxy)phenyl)thietan-3-ol (**1e**)

*i*PrMgCl·LiCl (1.30 M in THF, 2.54 mL, 3.3 mmol, 1.1 equiv) was added dropwise over 5 min to a solution of (4-iodophenoxy)triisopropylsilane (0.45 mL, 3.6 mmol, 1.2 equiv) in THF (4.0 mL) at 0 °C. The reaction mixture was stirred at 0 °C for a further 10 min and warmed to 25 °C for 2 h. A solution of thietan-3-one (411 mg, 3.0 mmol, 1.0 equiv) in THF (6.0 mL) was added dropwise to the reaction mixture at −78 °C, then leave them to stir for 1 h. Following a further 24 h at 25 °C the reaction mixture was cooled to 0 °C and then quenched with sat. aq. NH_4_Cl (25 mL). The aqueous portion was extracted with Et_2_O (3 × 25 mL). The combined organic layers were dried over Na_2_SO_4_, filtered and concentrated under reduced pressure. Purification by flash chromatography (70% Et_2_O/pentane) afforded 3-(4-((triisopropylsilyl)oxy)phenyl)thietan-3-ol **1e** as yellow oil (122 mg, 12%). R_f_ = 0.36 (30% EtOAc/hexane); IR (film)/cm^–1^ 3380 (OH), 2941, 2865, 1605, 1509, 1462, 1263, 1172, 1058, 1012, 995, 910, 881, 835, 682, 655, 554; ^1^H NMR (400 MHz, CDCl_3_) δ 7.50 (d, *J* = 8.7 Hz, 2H, 2 × Ar–H), 6.91 (d, *J* = 8.7 Hz, 2H, 2 × Ar–H), 3.62 (d, *J* = 9.8 Hz, 2H, C*H*H–S–*CH*H), 3.57 (d, *J* = 9.8 Hz, 2H, CH*H*–S–CH*H*), 1.28 (q, *J* = 7.0 Hz, 3H, 3 × Si–CH), 1.12 (d, *J* = 7.0 Hz, 18H, 6 × CH_3_); ^13^C{^1^H} NMR (101 MHz, CDCl_3_) δ 155.8 (Ar–C_q_), 137.2 (Ar–C_q_), 125.5 (2 × Ar–C), 119.8 (2 × Ar–C), 78.9 (C_q_), 42.6 (CH_2_–S–CH_2_), 17.9 (6 × CH_3_), 12.7 (3 × Si–C); HRMS (APCI) *m*/*z* Calculated for C_18_H_29_O_2_SSi [M – H]^+^: 337.1652; Found: 337.1662.

#### 3-Hydroxy-3-(*p*-tolyl)thietane (**1f**)

4-Methylphenyl magnesium bromide (0.45 M in Et_2_O, 29 mL, 13 mmol, 1.3 equiv) was added dropwise to a solution of thietan-3-one (882 mg, 10 mmol, 1 equiv) in anhydrous THF (20 mL, 0.5 M) at −78 °C in a 100 mL round-bottom flask. The reaction mixture was stirred at −78 °C for 30 min, warmed to 25 °C and stirred for further 1 h. Sat. aq. NH_4_Cl (50 mL) was added and phases were separated. The aqueous phase was extracted with CH_2_Cl_2_ (3 × 50 mL). The organic layers were combined, washed with brine (100 mL), dried over Na_2_SO_4_, filtered and concentrated in vacuo using a rotatory evaporator. Purification by flash chromatography (20% Et_2_O/pentane) afforded 3-hydroxy-3-(p-tolyl)thietane **1f** as a pale-yellow oil (1.36 g, 75%). R_f_ = 0.26 (30% Et_2_O/pentane); IR (film)/cm^–1^ 3370 (OH), 2982, 2936, 1908, 1610, 1513, 1446, 1370, 1267, 1208, 1173, 1111, 1051, 948, 880, 820; ^1^H NMR (400 MHz, CDCl_3_) δ 7.56–7.54 (2 H, m, 2 × Ar–H), 7.25–7.23 (2 H, m, 2 × Ar–H), 3.65 (2 H, d, *J* = 10.4 Hz, C*H*H–S–C*H*H), 3.59 (2 H, d, *J* = 10.4 Hz, CH*H*–S–CH*H*), 2.81 (1 H, s, OH), 2.38 (3 H, s, CH_3_); ^13^C{^1^H} NMR (101 MHz, CDCl_3_) δ 141.5 (Ar–C_q_), 137.8 (Ar–C_q_), 129.3 (2 × Ar–C), 124.2 (2 × Ar–C), 79.0 (C_q_), 42.5 (CH_2_–S–CH_2_), 21.1 (CH_3_); HRMS (EI) *m*/*z* Calculated for C_10_H_12_OS^.+^ [M]^.+^: 180.0603, Found: 180.0599.

#### 3-Phenylthietan-3-ol (**1g**)

Phenyl magnesium bromide (1.0 M in THF, 50 mL, 50.0 mmol, 1.1 equiv) was added dropwise to a solution of thietane-3-one (4.01 g, 45.5 mmol, 1.0 equiv) in THF (141 mL, 0.24 M) at −78 °C. After stirring at −78 °C for 30 min, the reaction mixture was warmed up to 25 °C and stirred for 1 h. The reaction was then quenched with sat. NH_4_Cl (80 mL). The mixture was extracted with CH_2_Cl_2_ (3 × 50 mL). The combined organic layers were dried over Na_2_SO_4_, filtered, and concentrated in vacuo using a rotary evaporator. Purification by flash column chromatography (5–10% EtOAc/pentane) afforded 3-phenylthietan-3-ol **1g** as yellow oil (5.19 g, 68%). R_f_ = 0.42 (20% EtOAc/pentane); IR (film)/cm^–1^ 3369 (OH), 3057, 2937, 1493, 1447, 1361, 1210, 1174, 1052, 1028, 954, 913, 758, 693, 758; ^1^H NMR (400 MHz, CDCl_3_) δ 7.67 (dd, *J* = 7.6, 1.7 Hz, 2H, Ar–H), 7.43 (dd, *J* = 8.4, 6.7 Hz, 2H, Ar–H), 7.39–7.31 (m, 1H, Ar–H), 3.67 (d, *J* = 9.9 Hz, 2H, C*H*H–S–C*H*H), 3.59 (d, *J* = 10.1 Hz, 2H, CH*H*–S–CH*H*); ^13^C{^1^H} NMR (101 MHz, CDCl_3_) δ 144.4 (Ar–C_q_), 128.6 (2 × Ar–C), 128.0 (Ar–C), 124.2 (2 × Ar–C), 79.0 (C_q_), 42.4 (2 × CH_2_–S). HRMS (APCI) *m*/*z* Calculated for C_9_H_10_OS [M]^+^: 166.0447; Found: 166.0455. The observed characterization data (IR, ^1^H and ^13^C NMR) were consistent with that previously reported.^14^

#### 3-(4-Chlorophenyl)thietan-3-ol (**1h**)

4-Chlorophenyl magnesium bromide (1.0 M in 2-methyl tetrahydrofuran, 11 mL, 11.0 mmol, 1.1 equiv) was added dropwise to a solution of thietane-3-one (881.3 g, 10.0 mmol, 1.0 equiv) in THF (29 mL, 0.25 M) at −78 °C. After stirring at −78 °C for 30 min, the reaction mixture was warmed up to 25 °C and stirred for 3 h. The reaction was then quenched with sat. NH_4_Cl (80 mL). The mixture was extracted with CH_2_Cl_2_ (3 × 50 mL). The combined organic layers were dried over Na_2_SO_4_, filtered, and concentrated in vacuo using a rotary evaporator. Purification by flash column chromatography (5–10% EtOAc/pentane) afforded 3-(4-chlorophenyl)thietan-3-ol **1h** as yellow oil (1.09 g, 55%). R_f_ = 0.42 (20% EtOAc/pentane); IR (film)/cm^–1^ 3366 (OH), 2938, 1595, 1489, 1398, 1210, 1090, 1052, 1010, 824, 543; ^1^H NMR (400 MHz, CDCl_3_) δ 7.59 (d, *J* = 8.6 Hz, 2H, 2 × Ar–H), 7.36 (d, *J* = 8.6 Hz, 2H, 2 × Ar–H), 3.55 (s, 4H, CHH–S–CHH); ^13^C{^1^H} NMR (101 MHz, CDCl_3_) δ 143.1 (Ar–C_q_), 134.1 (Ar–C_q_), 128.9 (2 × Ar–C), 126.0 (2 × Ar–C), 78.8 (C_q_), 42.8 (2 × S–CH_2_). The observed characterization data (IR, ^1^H and ^13^C NMR) were consistent with that previously reported.^18^

#### 3-(4-(Trifluoromethyl)phenyl)thietan-3-ol (**1i**)

*i*PrMgCl·LiCl (1.30 M in THF, 4.8 mL, 6.3 mmol, 1.05 equiv) was added dropwise over 5 min to a solution of 4-iodobenzotrifluoride (0.97 mL, 6.6 mmol, 1.1 equiv) in THF (7.0 mL) at 0 °C. The reaction mixture was stirred at 0 °C for a further 10 min and warmed to 25 °C for 3 h. A solution of thietanone (529 mg, 6.0 mmol, 1.0 equiv) in THF (13.0 mL) was added dropwise to the reaction mixture at 0 °C, Following a further 24 h at 25 °C. The reaction mixture was cooled to 0 °C and then quenched with sat. aq. NH_4_Cl (25 mL). The aqueous portion was extracted with Et_2_O (3 × 25 mL). The organic extracts were combined, dried over Na_2_SO_4_, filtered and concentrated under reduced pressure. Purification by flash chromatography (10% EtOAc/pentane) afforded 3-(4-(trifluoromethyl)phenyl)thietan-3-ol **1i** as a yellow oil (952 mg, 68%). R_f_ = 0.55 (20% EtOAc/pentane); IR (film)/cm^–1^ 3400 (OH), 2942, 1619, 1409, 1322, 1213, 1163, 1110, 1067, 1015, 955, 840, 702, 609, 472; ^1^H NMR (400 MHz, CDCl_3_) δ 7.84 (d, *J* = 8.1 Hz, 2H, Ar–H), 7.67 (d, *J* = 8.2 Hz, 2H, Ar–H), 3.61 (s, 4H, CHH–S–CHH); ^13^C{^1^H} NMR (101 MHz, CDCl_3_) δ 148.2 (Ar–C_q_), 130.3 (q, *J* = 32.6 Hz, Ar–C_q_), 125.6 (q, *J* = 3.8 Hz, 2 × Ar–C), 124.0 (q, *J* = 271 Hz, CF_3_), 124.7 (2 × Ar–C), 78.6 (C_q_), 42.6 (2 × S–CH_2_); ^19^F NMR (377 MHz, CDCl_3_) δ −62.6; HRMS (APCI) *m*/*z* Calculated for C_10_H_8_SOF_3_ [M – H]^−^: 233.0253; Found: 233.0242.

### Synthesis of Thietanol Dioxides by *m*CPBA Oxidation: General procedure A

*m*-CPBA (3.0 equiv) was added portionwise to a solution of thietan-3-ol (1.0 equiv) in CH_2_Cl_2_ (0.13 M) at 0 °C. After stirring at 0 °C for 5 min, the reaction mixture was warmed to 25 °C and stirred for 3.5 h. The reaction was then quenched with sat. aq. NaHCO_3_ (50 mL) followed by 50 mL CH_2_Cl_2_. The phases were separated and the organic layer was further washed with NaHCO_3_ (20 mL). The aqueous layer was extracted with CH_2_Cl_2_ (2 × 50 mL). The organic layers were combined, dried over Na_2_SO_4_, filtered and concentrated in vacuo. Purification by flash column chromatography afforded the thietan-3-ol dioxide.

#### 3-Hydroxy-3-(4-methoxyphenyl)thietane 1,1-dioxide (**2a**)

Performed using general procedure A with thietanol **1a** (1.05 g, 5 mmol) and *m*-CPBA (77%, 3.36 g, 15.0 mmol). Purification by flash column chromatography (20–30% acetone/pentane) afforded 3-hydroxy-3-(4-methoxyphenyl)thietane 1,1-dioxide **2a** as a white solid (296 mg, 80%). R_f_ = 0.16 (30% acetone/hexane); mp = 127–129 °C; IR (film)/cm^–1^ 3486 (OH), 3024, 2955, 2913, 2840, 1610, 1512, 1466, 1416, 1376, 1291, 1253, 1209, 1179, 1132, 1111, 1033, 1010, 964, 894, 827, 748, 646, 601, 550, 486, 475, 424; ^1^H NMR (400 MHz, CDCl_3_) δ 7.42 (d, *J* = 8.8 Hz, 2H, 2 × Ar–H), 6.96 (d, *J* = 8.8 Hz, 2H, 2 × Ar–H), 4.63 (d, *J* = 14.9 Hz, 2H, C*H*H–S–C*H*H), 4.42 (d, *J* = 14.9 Hz, 2H, CH*H*–S–CH*H*), 3.84 (s, 3H, OCH_3_); ^13^C{^1^H} NMR (101 MHz, CDCl_3_) δ 159.9 (Ar–C_q_OMe), 133.1 (Ar–*C*_q_C_q_), 126.2 (2 × Ar–CH), 114.4 (2 × Ar–CH), 78.2 (CH_2_SO_2_CH_2_), 64.6 (C_q_), 55.4 (OCH_3_); HRMS (EI) *m*/*z* calculated for C_10_H_12_O_4_S^.+^ [M]^.+^: 228.0451, Found: 228.0447.

#### 3-Hydroxy-3-(2-methoxyphenyl)thietane 1,1-dioxide (**2b**)

Performed using general procedure A with thietanol **1b** (196 mg, 1.0 mmol) and *m*-CPBA (77%, 672 mg, 3.0 mmol). Purification by flash column chromatography (30% acetone/pentane) afforded 3-hydroxy-3-(2-methoxyphenyl)thietane 1,1-dioxide **2b** a white solid (226 mg, 99%). R_f_ = 0.36 (30% acetone/pentane); mp = 165–168 °C; IR (film)/cm^–1^ 3422 (OH), 3042, 2969, 1484, 1458, 1296, 1258, 1220, 1162, 1168, 1100, 1023, 752, 676, 544, 444, 424; ^1^H NMR (400 MHz, CDCl_3_) δ 7.44–7.29 (m, 2H, 2 × Ar–H), 7.10–6.89 (m, 2H, 2 × Ar–H), 4.75 (d, *J* = 15.0 Hz, 2H, C*H*H–SO_2_–C*H*H), 4.37 (d, *J* = 14.9 Hz, 2H, CH*H*–SO_2_–CH*H*), 3.94 (s, 3H, CH_3_); ^13^C{^1^H} NMR (101 MHz, CDCl_3_) δ 156.3 (Ar–C_q_), 130.5 (Ar–C), 128.2 (Ar–C_q_), 125.9 (Ar–C), 121.1 (Ar–C), 111.3 (Ar–C), 76.3 (2 × C–SO_2_), 63.7 (C_q_), 55.5 (CH_3_); HRMS (APCI) *m*/*z* Calculated for C_10_H_11_O_4_S [M – H]^+^: 227.0373; Found: 227.0373.

#### 3-Hydroxy-3-(3-methoxyphenyl)thietane 1,1-dioxide (**2c**)

Performed using general procedure A with thietanol **1c** (392 mg, 2.0 mmol) and *m*-CPBA (77%, 1.34 g, 6.0 mmol). Purification by flash column chromatography (20% acetone/pentane) afforded 3-hydroxy-3-(3-methoxyphenyl)thietane 1,1-dioxide **2c** as yellow oil (373 mg, 82%); R_f_ = 0.30 (30% acetone/pentane); IR (film)/cm^–1^ 3458 (OH), 2961, 1602, 1586, 1430, 1125, 1314 (S=O), 1291, 1205, 1158, 1037, 907, 725, 446; ^1^H NMR (400 MHz, acetone-*d*_6_) δ 7.37 (t, *J* = 8.2 Hz, 1H, Ar–H), 7.20 (m, 2H, 2 × Ar–H), 6.93 (dd, *J* = 8.2, 2.5 Hz, 1H, Ar–H), 5.75 (s, 1H, OH), 4.66 (d, *J* = 15.0 Hz, 2H, C*H*H–S–C*H*H), 4.45 (d, *J* = 15.0 Hz, 2H, CH*H*–S–CH*H*), 3.84 (s, 3H, OCH_3_); ^13^C{^1^H} NMR (101 MHz, acetone-*d*_6_) δ 160.0 (Ar–C_q_), 145.6 (Ar–C_q_), 129.8 (Ar–C), 117.1 (Ar–C), 113.2 (Ar–C), 111.0 (Ar–C), 78.6 (2 × S–CH_2_), 63.1 (C_q_), 54.8 (OCH_3_); HRMS (TOF-ES) *m*/*z* calculated for C_12_H_15_NO_4_SNa [M + MeCN + Na]^+^: 292.0622, Found: 292.0619.

#### 3-(Benzo[*d*][1,3]dioxol-5-yl)-3-hydroxythietane 1,1-dioxide (**2d**)

Performed using general procedure A with thietanol **1d** (252 mg, 1.2 mmol) and *m*-CPBA (77%, 864 mg, 3.6 mmol). Purification by flash column chromatography (30% acetone/pentane) afforded 3-(benzo[*d*][1,3]dioxol-5-yl)-3-hydroxythietane 1,1-dioxide **2d** a white solid (293 mg, 83%). R_f_ = 0.23 (30% acetone/pentane); mp = 140–145 °C; IR (film)/cm^–1^ 3438 (OH), 3039, 2973, 2905, 1685, 1487, 1438, 1291, 1177, 1150, 1031, 985, 918, 764, 624; ^1^H NMR (400 MHz, acetone-*d*_6_) δ 7.10–7.04 (m, 2H, 2 × Ar–H), 6.83 (d, *J* = 8.8 Hz, 1H, Ar–H), 5.99 (s, 2H, O–CH_2_–O), 5.66 (s, 1H, OH), 4.57 (d, *J* = 15.1 Hz, 2H, C*H*H–S–C*H*H), 4.36 (d, *J* = 15.1 Hz, 2H, CH*H*–S–CH*H*); ^13^C{^1^H} NMR (101 MHz, acetone-*d*_6_) δ 148.1 (Ar–C_q_), 147.4 (Ar–C_q_), 138.0 (Ar–C_q_), 118.5 (Ar–C), 107.8 (Ar–C), 105.9 (Ar–C), 101.5 (OCH_2_), 78.4 (2 × S–CH_2_), 63.2 (C_q_); HRMS(APCI) *m*/*z* Calculated for C_10_H_9_O_5_S [M – H]^+^: 241.0172; Found: 241.0165.

#### 3-Hydroxy-3-(4-((triisopropylsilyl)oxy)phenyl)thietane 1,1-dioxide (**2e**)

Performed using general procedure A with thietanol **1e** (336 mg, 1.0 mmol) and *m*-CPBA (77%, 672 mg, 3.0 mmol). Purification by flash column chromatography (30% acetone/pentane) afforded 3-hydroxy-3-(4-((triisopropylsilyl)oxy)phenyl)thietane 1,1-dioxide **2e** as a white solid (296 mg, 80%). R_f_ = 0.16 (30% acetone/pentane); mp = 105–109 °C; IR (film)/cm^–1^ 3460 (OH), 2944, 2866, 1607, 1511, 1267, 1210, 1167, 1128, 913, 839, 739, 685; ^1^H NMR (400 MHz, CDCl_3_) δ 7.33 (d, *J* = 8.7 Hz, 2H, 2 × Ar–H), 6.92 (d, *J* = 8.7 Hz, 2H, 2 × Ar–H), 4.63 (d, *J* = 14.9 Hz, 2H, C*H*H–SO_2_–C*H*H), 4.40 (d, *J* = 14.9 Hz, 2H, CH*H*–SO_2_–CH*H*), 1.26 (q, *J* = 7.3 Hz, 3H, 3 × Si–CH), 1.10 (d, *J* = 7.3 Hz, 18H, 6 × CH_3_); ^13^C{^1^H} NMR (101 MHz, CDCl_3_) δ 156.6 (Ar–C_q_), 133.4 (Ar–C_q_), 126.1 (2 × Ar–C), 120.3 (2 × Ar–C), 78.3 (CH_2_–SO_2_–CH_2_), 64.6 (C_q_), 17.9 (6 × CH_3_), 12.6 (3 × Si–CH); HRMS (APCI) *m*/*z* Calculated for C_18_H_31_O_4_SSi [M + H]^+^: 371.1707; Found: 371.1706.

#### 3-Hydroxy-3-(*p*-tolyl)thietane 1,1-dioxide (**2f**)

Performed using general procedure A with thietanol **1f** (180 mg, 1.0 mmol) and *m*-CPBA (77%, 672.4 mg, 3.0 mmol). Purification by flash column chromatography (30% acetone/pentane) afforded 3-hydroxy-3-(*p*-tolyl)thietane 1,1-dioxide **2f** a white solid (177 mg, 83%). R_f_ = 0.36 (30% acetone/pentane); mp = 114–119 °C; IR (film)/cm^–1^ 3455 (OH), 3027, 2958, 1515, 1383, 1307, 1206, 1165, 1126, 1040, 1008, 971, 818, 764, 595, 547, 483; ^1^H NMR (400 MHz, CDCl_3_) δ 7.37 (d, *J* = 8.0 Hz, 2H, Ar–H), 7.23 (d, *J* = 8.0 Hz, 2H, Ar–H), 4.59 (d, *J* = 15.1 Hz, 2H, C*H*H–S–C*H*H), 4.40 (d, *J* = 15.1 Hz, 2H, CH*H*–S–CH*H*), 2.37 (s, 3H, CH_3_); ^13^C{^1^H} NMR (101 MHz, CDCl_3_) δ 138.7 (Ar–C_q_), 138.5 (Ar–C_q_), 129.7 (2 × Ar–C), 124.6 (2 × Ar–C), 78.4 (2 × S–CH_2_), 64.4 (C_q_), 21.0 (CH_3_); HRMS (APCI) *m*/*z* Calculated for C_10_H_10_O_3_S [M – H]^−^: 211.0434; Found: 211.0428.

#### 3-Hydroxy-3-phenylthietane 1,1-dioxide (**2g**)

Performed using general procedure A with thietanol **1g** (333 mg, 2.0 mmol) and *m*-CPBA (77%, 1.34 g, 6.0 mmol). Purification by flash column chromatography (30% acetone/pentane) afforded 3-hydroxy-3-phenylthietane 1,1-dioxide as a **2g** white solid (317 mg, 80%). R_f_ = 0.16 (30% acetone/pentane); mp = 103–109 °C; IR (film)/cm^–1^ 3458 (OH), 3020, 2960, 1384, 1311, 1211, 1168, 1128, 1008, 972, 763, 699, 494; ^1^H NMR (400 MHz, CDCl_3_) δ 7.52 (d, *J* = 7.0 Hz, 2H, 2 × Ar–H), 7.45 (t, *J* = 7.5 Hz, 2H, 2 × Ar–H), 7.39 (t, *J* = 7.2 Hz, 1H, Ar–H), 4.65 (d, *J* = 15.0 Hz, 2H, C*H*H–S–C*H*H), 4.43 (d, *J* = 15.0 Hz, 2H, CH*H*–S–CH*H*), 3.25 (s, 1H, OH); ^13^C{^1^H} NMR (101 MHz, CDCl_3_) δ 141.2 (Ar–C_q_), 129.1 (2 × Ar–C), 128.8 (Ar–C), 124.7 (2 × Ar–C), 78.5 (2 × SO_2_–CH_2_), 64.7 (C_q_); HRMS (APCI) *m*/*z* Calculated for C_9_H_11_O_3_S [M + H]^+^: 199.0423; Found: 199.0423.

#### 3-(4-Chlorophenyl)-3-hydroxythietane 1,1-dioxide (**2h**)

Performed using general procedure A with thietanol **1h** (401 mg, 2.0 mmol) and *m*-CPBA (77%, 1.34 g, 6.0 mmol). Purification by flash column chromatography (30% acetone/pentane) afforded 3-(4-chlorophenyl)-3-hydroxythietane 1,1-dioxide **2h** a white solid (232.7 mg, 50%). R_f_ = 0.36 (30% acetone/pentane); mp = 168–175 °C; IR (film)/cm^–1^ 3480 (OH), 3021, 1490, 1297,1214, 1177, 1132, 1096, 1011, 828, 754, 638, 543; ^1^H NMR (400 MHz, DMSO) δ 7.57 (d, *J* = 8.7 Hz, 2H, 2 × Ar–H), 7.48 (d, *J* = 8.6 Hz, 2H, 2 × Ar–H), 6.83 (s, 1H, OH), 4.68 (d, *J* = 15.3 Hz, 2H, C*H*H–S–C*H*H), 4.37 (d, *J* = 15.4 Hz, 2H, CH*H*–S–CH*H*); ^13^C{^1^H} NMR (101 MHz, DMSO) δ 143.0 (Ar–C_q_), 132.4 (Ar–C_q_), 128.3 (2 × Ar–C), 127.2 (2 × Ar–C), 78.3 (2 × CH_2_–S), 62.5 (C_q_); HRMS (APCI) *m*/*z* Calculated for C_9_H_10_O_3_S^35^Cl [M + H]^+^: 233.0034; Found: 233.0034.

#### 3-Hydroxy-3-(4-(trifluoromethyl)phenyl)thietane 1,1-dioxide (**2i**)

Performed using general procedure A with thietanol **1g** (469 mg, 2.0 mmol) and *m*-CPBA (77%, 1.34 g, 6.0 mmol). Purification by flash column chromatography (20% acetone/pentane) afforded 3-hydroxy-3-(4-(trifluoromethyl)phenyl)thietane 1,1-dioxide **2i** as a white solid (280.2 mg, 51%). R_f_ = 0.47 (20% acetone/pentane); mp = 138–141 °C; IR (film)/cm^–1^ 3457 (OH), 3031, 2963, 1619, 1411, 1388, 1320 (S=O), 1212, 1166, 1111, 1068, 1014, 976, 843, 638, 513, 422; ^1^H NMR (400 MHz, acetone-*d*_6_) δ 7.90 (d, *J* = 8.1 Hz, 2H, 2 × Ar–H), 7.79 (d, *J* = 8.1 Hz, 2H, 2 × Ar–H), 6.04 (s, 1H, OH), 4.72 (d, *J* = 15.3 Hz, 2H, C*H*H–SO_2_–C*H*H), 4.50 (d, *J* = 15.3 Hz, 2H, CH*H*–SO_2_–CH*H*); ^13^C{^1^H} NMR (101 MHz, acetone-*d*_6_) δ 149.4 (Ar–C_q_), 130.0 (q, *J* = 32.3 Hz, Ar–C_q_), 126.8 (2 × Ar–C), 126.3 (q, *J* = 32.3 Hz, 2 × Ar–C) 126.3 (q, *J* = 272.5 Hz, CF_3_), 79.6 (2 × CH_2_–SO_2_), 63.8 (C_q_); ^19^F NMR (377 MHz, acetone-*d*_6_) δ −63.1; HRMS (ESI) *m*/*z* Calculated for C_10_H_8_SO_3_F_3_ [M – H]^−^: 265.0152; Found: 265.0142.

### Friedel–Crafts Reactions with Thietan-3-ol Dioxides: General Procedure B

Calcium(II) bis(trifluoromethanesulfonimide) (6.0 mg, 0.01 mmol, 0.05 equiv) and tetrabutylammonium hexafluorophosphate (4.0 mg, 0.01 mmol, 0.05 equiv) were added sequentially to a solution of thietane-3-ol dioxide (0.20 mmol, 1 equiv) and arene (0.60 mmol, 3 equiv) in toluene (0.4 mL, 0.5 M) in reaction vial. The reaction vial was sealed under argon, and the mixture was heated at 110 °C for 4.5 h then cooled to rt. Sat. aq. NaHCO_3_ (15 mL) was added followed by CH_2_Cl_2_ (15 mL). The phases were separated and the aqueous layer was extracted with CH_2_Cl_2_ (2 × 15 mL). The organic layers were combined, dried over Na_2_SO_4_, filtered and concentrated in vacuo. Purification by flash column chromatography afforded the diarylthietane dioxide.

#### 3-(4-Hydroxy-3-methylphenyl)-3-(4-methoxyphenyl)thietane 1,1-dioxide (**3aa**)

Performed using general procedure B with thietanol dioxide **2a** (45.7 mg, 0.20 mmol, 1 equiv) and 2-methylphenol (65.3 mg, 0.60 mmol, 3 equiv). Purification by flash column chromatography (3–5% Et_2_O/CH_2_Cl_2_) afforded diarylthietane dioxide **3aa** as a white solid (59.2 mg, 93%). R_f_ = 0.18 (3% Et_2_O/CH_2_Cl_2_); IR (film)/cm^–1^ 3432 (OH), 3024, 2957, 2929, 2837, 1607, 1509, 1460, 1413, 1396, 1305 (SO_2_), 1270, 1249, 1214, 1183, 1116, 1031, 909, 824, 771, 731, 600, 550, 486; ^1^H NMR (400 MHz, CDCl_3_) δ 7.19–7.17 (m, 2H, 2 × Ar–H), 6.99 (d, *J* = 2.6 Hz, 1H, Ar–H), 6.95–6.93 (dd, *J* = 8.3, 2.6 Hz, 1H, Ar–H), 6.89–6.86 (m, 2H, 2 × Ar–H), 6.72–6.69 (d, *J* = 8.3 Hz, 1H, Ar–H), 5.06 (s, 1H, OH), 4.86–4.83 (d, *J* = 12.9 Hz, 2H, C*H*H–SO_2_–C*H*H), 4.82–4.79 (d, *J* = 12.9 Hz, 2H, CH*H*–SO_2_–CH*H*), 3.80 (s, 3H, OCH_3_), 2.21 (s, 3H, CH_3_); ^13^C{^1^H} NMR (101 MHz, CDCl_3_) δ 158.5 (Ar–C_q_O), 153.0 (Ar–C_q_O), 137.0 (Ar–C_q_), 136.7 (Ar–C_q_), 129.3 (Ar–C), 127.7 (2 × Ar–C), 125.2 (Ar–C), 124.5 (Ar–*C*_*q*_), 115.1 (Ar–C), 114.2 (2 × Ar–C), 76.8 (CH_2_–SO_2_–CH_2_), 55.3 (OCH_3_), 36.4 (C_q_), 16.0 (CH_3_); HRMS (APCI) *m*/*z* Calculated for C_17_H_19_O_4_S^+^ [M + H]^+^: 319.0999, Found: 319.1002.

#### 3-(4-Hydroxyphenyl)-3-(4-methoxyphenyl)thietane 1,1-dioxide (**3ab**)

[1 mmol scale reaction] Calcium(II) bis(trifluoromethanesulfonimide) (30.0 mg, 0.05 mmol, 0.05 equiv) and tetrabutylammonium hexafluorophosphate (19.3 mg, 0.05 mmol, 0.05 equiv) were added sequentially to a solution of thietane dioxide **2a** (228 mg, 1.0 mmol, 1 equiv) and phenol (282 mg, 3.0 mmol, 3.0 equiv) in toluene (2.0 mL, 0.5 M). The reaction mixture was stirred at 40 °C for 4.5 h then sat. aq. NaHCO_3_ (30 mL) was added followed by CH_2_Cl_2_ (30 mL). The phases were separated and the aqueous layer was extracted with CH_2_Cl_2_ (2 × 30 mL). The organic layers were combined, dried over Na_2_SO_4_, filtered and concentrated in vacuo using a rotatory evaporator. Purification by flash column chromatography (0–2% Et_2_O/CH_2_Cl_2_) afforded diarylthietane dioxide **3ab** as a white solid (255 mg, 83%); mp = 186–188 °C; IR (film)/cm^–1^ 3459 (OH), 2952, 1755, 1606, 1510, 1306, 1210, 1180, 1127, 1013, 831, 766, 644, 545; ^1^H NMR (400 MHz, CDCl_3_) δ 7.16 (d, *J* = 8.9 Hz, 2H, 2 × Ar–H), 7.12 (d, *J* = 8.7 Hz, 2H, 2 × Ar–H), 6.87 (d, *J* = 8.9 Hz, 2H, 2 × Ar–H), 6.79 (d, *J* = 8.7 Hz, 2H, 2 × Ar–H), 5.05 (s, 1H, OH), 4.82 (s, 4H, CHH–SO_2_–CHH), 3.79 (s, 3H, OCH_3_); ^13^C{^1^H} NMR (101 MHz, CDCl_3_) δ 158.6 (Ar–C_q_), 154.7 (Ar–C_q_), 137.0 (Ar–C_q_), 136.7 (Ar–C_q_), 128.0 (2 × Ar–C), 127.7 (2 × Ar–C), 115.7 (2 × Ar–C), 114.2 (2 × Ar–C), 77.0 (2 × CH_2_–SO_2_), 55.3 (OCH_3_), 36.4 (C_q_); HRMS(ESI) *m*/*z* Calculated for C_16_H_15_O_4_S [M – H]^−^: 303.0697; Found: 303.0697.

#### 3-(4-Hydroxy-3,5-dimethylphenyl)-3-(4-methoxyphenyl)thietane 1,1-dioxide (**3ac**)

Performed using general procedure B with thietanol dioxide **2a** (45.7 mg, 0.20 mmol, 1 equiv) and 2,6-dimethylphenol (74.8 mg, 0.6 mmol, 3.0 equiv). Purification by flash column chromatography (0–2% Et_2_O/CH_2_Cl_2_) afforded diarylthietane dioxide **3ac** as a white solid (62.5 mg, 94%). R_f_ = 0.38 (5% Et_2_O/CH_2_Cl_2_); mp = 186–188 °C; IR (film)/cm^–1^ 3493, 3026, 2958, 2837, 1607, 1512, 1490, 1462, 1393, 1308 (SO_2_ st), 1253, 1216, 1183, 1129, 1030, 910, 833, 732; ^1^H NMR (400 MHz, CDCl_3_) δ 7.19 (d, *J* = 8.8 Hz, 2H, 2 × Ar–H), 6.87 (d, *J* = 8.8 Hz, 2H, 2 × Ar–H), 6.84 (s, 2H, 2 × Ar–H), 4.85 (d, *J* = 13.2 Hz, 2H, C*H*H–S–C*H*H), 4.79 (d, *J* = 13.2 Hz, 3H, CH*H*–S–CH*H*), 4.76 (s, 1H, OH), 3.80 (s, 3H, OCH_3_), 2.21 (s, 6H, 2 × CH_3_); ^13^C{^1^H} NMR (101 MHz, CDCl_3_) δ 158.5 (Ar–C_q_OMe), 151.3 (Ar–C_q_), 137.1 (Ar–C), 136.0 (Ar–C), 127.6 (2 × Ar–C), 126.8(2 × Ar–C), 123.5 (2 × Ar–*C*_*q*_), 114.1 (2 × Ar–CH), 76.7 (CH_2_–SO_2_–CH_2_), 55.3 (OCH_3_), 36.3 (C_q_), 16.1 (2 × CH_3_); HRMS (APCI) *m*/*z* Calculated for C_18_H_19_SO_4_^–^ [M – H]^−^: 331.1010, Found: 331.0995.

#### 3-(4-Hydroxy-3-isopropylphenyl)-3-(4-methoxyphenyl)thietane 1,1-dioxide (**3ad**)

Performed using general procedure B with thietanol dioxide **2a** (45.7 mg, 0.20 mmol, 1 equiv) and 2-isopropylphenol (0.081 mL, 0.60 mmol, 3 equiv). Purification by flash column chromatography (40% EtOAc/hexane) afforded diarylthietane dioxide **3ad** as a white solid (68.8 mg, 99%). R_f_ = 0.15 (40% EtOAc/hexane); mp = 165–168 °C; IR (film)/cm^–1^ 3397 (OH), 3024, 2962, 1607, 1510, 1463, 1422, 1311, 1241, 1183, 1132, 1024, 845, 818, 777, 647, 541, 484; ^1^H NMR (400 MHz, CDCl_3_) δ 7.17 (d, *J* = 8.8 Hz, 2H, Ar–H), 7.06 (d, *J* = 2.6 Hz, 1H, Ar–H), 6.93–6.83 (m, 3H, Ar–H), 6.68 (d, *J* = 8.3 Hz, 1H, Ar–H), 4.95 (s, 1H, OH), 4.89–4.77 (m, 4H, 2 × C*H*H–SO_2_–C*H*H), 3.80 (s, 3H, OCH_3_), 3.17 (p, *J* = 6.9 Hz, 1H, CH), 1.21 (d, *J* = 6.9 Hz, 6H, 2 × CH_3_); ^13^C{^1^H} NMR (101 MHz, CDCl_3_) δ 158.5 (Ar–C_q_), 152.0 (Ar–C_q_), 137.0 (2 × Ar–C_q_), 135.0 (Ar–C_q_), 127.7 (2 × Ar–C), 124.9 (Ar–C), 124.8 (Ar–C), 115.4 (Ar–C), 114.2 (2 × Ar–C), 77.2 (2 × SO_2_–CH_2_), 55.3 (O–CH_3_), 36.4 (C_q_), 27.4 (CH), 22.4 (2 × CH_3_); HRMS(APCI) *m*/*z* Calculated for C_19_H_21_O_4_S [M – H]^+^: 345.1155; Found: 345.1158.

#### 3-(4-Hydroxy-5-isopropyl-2-methylphenyl)-3-(4-methoxyphenyl)thietane 1,1-dioxide (**3ae**)

Performed using general procedure B with thietanol dioxide **2a** (45.7 mg, 0.20 mmol, 1 equiv) and thymol (90.0 mg, 0.60 mmol, 3 equiv). Purification by flash column chromatography (25–30% EtOAc/pentane) afforded diarylthietane dioxide **3ae** as a yellow solid (58.4 mg, 81%). R_f_ = 0.43 (30% EtOAc/pentane); mp = 200–204 °C; IR (film)/cm^–1^ 3357 (OH), 2958, 1610, 1582, 1510, 1461, 1408, 1303, 1223, 1183, 1156, 1129, 1107, 1016, 830, 807, 785, 551, 485; ^1^H NMR (400 MHz, CDCl_3_) δ 7.25 (d, *J* = 8.9 Hz, 2H, Ar–H), 7.12 (s, 1H, Ar–H), 6.85 (d, *J* = 8.9 Hz, 2H, Ar–H), 6.59 (s, 1H, Ar–H), 4.90 (d, *J* = 14.7 Hz, 2H, C*H*H–SO_2_–C*H*H), 4.72 (d, *J* = 14.7 Hz, 2H, CH*H*–SO_2_–CH*H*), 3.80 (s, 3H, OCH_3_), 3.26 (sept, *J* = 6.9 Hz, 1H, CH), 1.85 (s, 3H, CH_3_), 1.33 (d, *J* = 6.9 Hz, 6H, 2 × CH_3_); ^13^C{^1^H} NMR (101 MHz, CDCl_3_) δ 158.5 (Ar–C_q_), 152.3 (Ar–C_q_), 136.1 (Ar–C_q_), 134.7 (Ar–C_q_), 133.9 (Ar–C_q_), 131.5 (Ar–C_q_), 127.3 (2 × Ar–C), 124.9 (Ar–C), 119.5 (2 × Ar–C), 114.0 (Ar–C), 76.3 (CH_2_–SO_2_), 55.3 (OCH_3_), 36.5 (C_q_), 27.2 (CH), 22.6 (2 × CH_3_), 20.5 (CH_3_); HRMS(ES-ToF) *m*/*z* Calculated for C_20_H_25_O_4_S [M + H]^+^: 361.1474; Found: 361.1479.

#### 3-(4-Hydroxy-2-isopropyl-5-methylphenyl)-3-(4-methoxyphenyl)thietane 1,1-dioxide (**3af**)

Performed using general procedure B with thietanol dioxide **2a** (45.7 mg, 0.20 mmol, 1 equiv) and carvacrol (0.092 mL, 0.60 mmol, 3 equiv). Purification by flash column chromatography (25–30% EtOAc/pentane) afforded diarylthietane dioxide **3af** as a white solid (39.6 mg, 55%). R_f_ = 0.43 (30% EtOAc/pentane); mp = 190–195 °C; IR (film)/cm^–1^ 3491 (OH), 2958, 2867, 1610, 1572, 1511, 1304, 1280, 1188, 1136, 1099, 1036, 905, 826, 784, 532, 476; ^1^H NMR (400 MHz, CDCl_3_) δ 7.23 (d, *J* = 8.9 Hz, 2H, Ar–H), 7.05 (s, 1H, Ar–H), 6.81 (d, *J* = 8.9 Hz, 2H, Ar–H), 6.71 (s, 1H, Ar–H), 4.84 (d, *J* = 13.6 Hz, 2H, C*H*H–SO_2_–C*H*H), 4.70 (d, *J* = 14.5 Hz, 2H, CH*H*–SO_2_–CH*H*), 3.77 (s, 3H, OCH_3_), 2.30 (sept, *J* = 8.9 Hz, 1H, CH), 2.28 (s, 3H, CH_3_), 0.85 (d, *J* = 6.7 Hz, 6H, 2 × CH_3_); ^13^C{^1^H} NMR (101 MHz, CDCl_3_) δ 158.4 (Ar–C_q_), 153.8 (Ar–C_q_), 146.6 (Ar–C_q_), 137.1 (Ar–C_q_), 132.8 (Ar–C_q_), 129.0 (Ar–C), 127.3 (2 × Ar–C), 120.7 (Ar–C_q_), 114.9 (Ar–C), 114.0 (2 × Ar–C), 76.6 (CH_2_–SO_2_), 55.3 (OCH_3_), 36.2 (C_q_), 30.0 (CH), 23.8 (2 × CH_3_), 15.6 (CH_3_); HRMS(APCI) *m*/*z* Calculated for C_20_H_25_O_4_S [M + H]^+^: 360.1468; Found: 360.1461.

#### 3-(2-Hydroxy-5-methylphenyl)-3-(4-methoxyphenyl)thietane 1,1-dioxide (**3ag**)

Performed using general procedure B with thietanol dioxide **2a** (45.7 mg, 0.20 mmol, 1 equiv) and *p*-cresol (0.062 mL, 0.60 mmol, 3 equiv). Purification by flash column chromatography (2–5% Et_2_O/CH_2_Cl_2_) afforded diarylthietane dioxide **3ag** a white solid (54.4 mg, 90%). R_f_ = 0.56 (10% Et_2_O/CH_2_Cl_2_); mp = 178–180 °C; IR (film)/cm^–1^ 3376, 3040, 2922, 1608, 1509, 1414, 1388, 1299, 1250, 1220, 1183, 1120, 1029, 841, 814, 768, 632, 614, 546; ^1^H NMR (400 MHz, DMSO-*d*_6_) δ 9.51 (s, 1H, OH), 7.39 (d, *J* = 8.9 Hz, 2H, 2 × Ar–H), 7.15 (d, *J* = 1.8 Hz, 1H, Ar–H), 6.90 (dd, *J* = 8.5, 1.8 Hz, 1H, Ar–H), 6.85 (d, *J* = 8.9 Hz, 2H, 2 × Ar–H), 6.66 (d, *J* = 8.5 Hz, 1H, Ar–H), 4.84 (s, 4H, CHH–S–CHH), 3.71 (s, 3H, OCH_3_), 2.23 (s, 3H, CH_3_); ^13^C{^1^H} (101 MHz, DMSO) δ 158.1 (Ar–C_q_), 152.5 (Ar–C_q_), 136.5 (Ar–C_q_), 130.9 (Ar–C), 129.3 (Ar–C), 128.3 (Ar–C), 128.2 (2 × Ar–C), 128.0 (Ar–C_q_), 116.4 (Ar–C), 113.9 (2 × Ar–C), 75.0 (CHH–S–CHH), 55.5 (OCH_3_), 36.1 (C_q_), 20.7 (CH_3_); HRMS (ESI) *m*/*z* Calculated for C_17_H_17_O_4_S [M – H]^−^: 317.0853; Found: 317.0851.

#### 3-(2-Hydroxy-5-methoxyphenyl)-3-(4-methoxyphenyl)thietane 1,1-dioxide (**3ah**)

Performed using general procedure B with thietanol dioxide **2a** (45.7 mg, 0.20 mmol, 1 equiv) and 4-methoxyphenol (74.5 mg, 0.60 mmol, 3 equiv). Purification by flash column chromatography (2–5% Et_2_O/CH_2_Cl_2_) afforded diarylthietane dioxide **3ah** as a white solid (53.7 mg, 80%). R_f_ = 0.56 (10% Et_2_O/CH_2_Cl_2_); mp = 178–180 °C; IR (film)/cm^–1^ 3410 (OH), 2956, 2928, 1608, 1511, 1424, 1308, 1253, 1209, 1186, 1167, 1134, 1033, 811, 545, 501; ^1^H NMR (400 MHz, acetone-*d*_6_) δ 7.49 (d, *J* = 9.1 Hz, 2H, 2 × Ar–H), 6.99 (d, *J* = 2.9 Hz, 1H Ar–H), 6.85 (d, *J* = 9.1 Hz, 2H, 2 × Ar–H), 6.81–6.71 (m, 2H, 2 × Ar–H), 4.95 (d, *J* = 14.9 Hz, 2H, C*H*H–S–C*H*H), 4.83 (d, *J* = 14.9 Hz, 2H, CH*H*–S–CH*H*), 3.77 (s, 3H, OCH_3_), 3.76 (s, 3H, OCH_3_); ^13^C{^1^H} NMR (101 MHz, acetone-*d*_6_) δ 158.4 (Ar–C_q_), 153.1 (Ar–C_q_), 148.1 (Ar–C_q_), 136.2 (Ar–C_q_), 131.8 (Ar–C_q_), 127.8 (2 × Ar–C), 116.8 (Ar–C), 113.8 (Ar–C), 113.4 (2 × Ar–C), 113.3 (Ar–C), 74.8 (2 × C–SO_2_), 55.1 (OCH_3_), 54.6 (OCH_3_), 35.7 (C_q_); HRMS (ESI) *m*/*z* Calculated for C_17_H_17_O_5_S [M – H]^−^: 333.0802; Found: 333.0802.

#### (8*R*,9*S*,13*S*,14*S*)-3-Hydroxy-2-(3-(4-methoxyphenyl)-1,1-dioxidothietan-3-yl)-13-methyl-6,7,8,9,11,12,13,14,15,16-decahydro-17*H*-cyclopenta[*a*]phenanthren-17-one (**3ai**)

Performed using general procedure B with thietanol dioxide **2a** (45.7 mg, 0.20 mmol, 1 equiv) and estrone (162 mg, 0.60 mmol, 3 equiv). Purification by flash column chromatography (50% acetone/pentane) afforded diarylthietane dioxide **3ai** as a white solid (30.9 mg, 57%). R_f_ = 0.23 (50% acetone/pentane); IR (film)/cm^–1^ 3402 (OH), 2929, 2861, 1734, 1610, 1511, 1416, 1315, 1253, 1217, 1186, 1143, 1122, 1033, 828, 734, 545; ^1^H NMR (400 MHz, CDCl_3_) δ 7.31 (d, *J* = 8.5 Hz, 2H, Ar–H), 7.08 (s, 1H, Ar–H), 6.83 (d, *J* = 8.5 Hz, 2H, Ar–H), 6.48 (s, 1H, Ar–H), 5.18 (s, 1H, OH), 4.97–4.84 (m, 2H, CHH–SO_2_–CHH), 4.75 (ddd, *J* = 14.3, 10.1, 3.7 Hz, 2H, CHH–SO_2_–CHH), 3.76 (s, 3H, OCH_3_), 2.90–1.34 (m, 15H), 0.92 (s, 3H, CH_3_); ^13^C{^1^H} NMR (101 MHz, CDCl_3_) δ 221.0 (C_q_=O), 158.5 (Ar–C_q_), 150.7 (Ar–C_q_), 137.9 (Ar–C_q_), 135.7 (Ar–C_q_), 132.4 (Ar–C_q_), 127.6 (Ar–C_q_), 127.4 (2 × Ar–C), 124.5 (Ar–C), 116.9 (Ar–C), 114.0 (2 × Ar–C), 75.6 (2 × C–SO_2_), 55.3 (OCH_3_), 50.3 (CH), 48.0 (C_q_), 44.0 (CH), 38.3 (CH), 35.9 (CH_2_), 35.4 (CH_2_), 31.5 (CH_2_), 29.0 (CH_2_), 26.3 (C_q_), 26.1 (CH_2_), 21.6 (CH_2_), 13.9 (CH_3_). HRMS (APCI) *m*/*z* Calculated for C_28_H_32_SO_5_ [M + H]^+^: 481.2043; Found: 481.2041.

#### 3-(3,4-Dihydroxyphenyl)-3-(4-methoxyphenyl)thietane 1,1-dioxide (**3aj**)

Performed using general procedure B with thietanol dioxide **2a** (45.7 mg, 0.20 mmol, 1 equiv) and catechol (66.0 mg, 0.60 mmol, 3 equiv). Purification by flash column chromatography (1–10% Et_2_O/CH_2_Cl_2_) afforded diarylthietane dioxide **3aj** a white solid (47.4 mg, 78%). R_f_ = 0.29 (5% Et_2_O/CH_2_Cl_2_); mp = 182–186 °C; IR (film)/cm^–1^ 3396 (OH), 2959, 2838, 1689, 1607, 1512, 1435, 1293, 1251, 1220, 1183, 1125, 1030, 828, 812, 785, 634, 546, 486; ^1^H NMR (400 MHz, MeOD) δ 7.26 (d, *J* = 8.9 Hz, 2H, Ar–H), 6.88 (d, *J* = 8.7 Hz, 2H, Ar–H), 6.77–6.62 (m, 3H, Ar–H), 4.80 (s, 4H, 2 × C*H*H–SO_2_–C*H*H), 3.76 (s, 3H, O–CH_3_); ^13^C{^1^H} NMR (101 MHz, MeOD) δ 159.9 (Ar–C_q_), 146.5 (Ar–C_q_), 145.4 (Ar–C_q_), 138.8 (Ar–C_q_), 138.4 (Ar–C_q_), 128.9 (2 × Ar–C), 118.9 (Ar–C), 116.2 (Ar–C), 115.2 (Ar–C), 115.0 (2 × Ar–C), 77.2 (2 × SO_2_–CH_2_), 55.7 (C_q_), 37.7 (O–CH_3_); HRMS (APCI) *m*/*z* Calculated for C_16_H_15_O_5_S [M – H]^+^: 319.0646; Found: 319.0640.

#### 3-(2,4-Dihydroxyphenyl)-3-(4-methoxyphenyl)thietane 1,1-dioxide (**3ak**)

Performed using general procedure B with thietanol dioxide **2a** (45.7 mg, 0.20 mmol, 1 equiv) and resorcinol (66.0 mg, 0.60 mmol, 3 equiv). The reaction was quenched by the addition of sat. aq. NaHCO_3_ (15 mL), then extracted with EtOAc (3 × 15 mL). The combined organic layers were dried over Na_2_SO_4_, filtered and concentrated in vacuo using a rotary evaporator. Purification by flash column chromatography (1–10% Et_2_O/CH_2_Cl_2_) afforded diarylthietane dioxide **3ak** white solid (43.2 mg, 71%). R_f_ = 0.29 (5% Et_2_O/CH_2_Cl_2_); IR (film)/cm^–1^ 3401 (OH), 2963, 1607, 1513, 1303, 1251, 1217, 1186, 1130, 1114, 1031, 832, 544; ^1^H NMR (400 MHz, acetone-*d*_6_) δ 7.41 (d, *J* = 8.9 Hz, 2H, 2 × Ar–H), 7.18 (d, *J* = 8.7 Hz, 1H, Ar–H), 6.83 (d, *J* = 8.9 Hz, 2H, 2 × Ar–H), 6.40 (d, *J* = 3.3 Hz, 2H, 2 × Ar–H), 4.86 (d, *J* = 14.6 Hz, 2H, C*H*H–S–C*H*H), 4.76 (d, *J* = 14.4 Hz, 2H, CH*H*–S–CH*H*), 3.73 (s, 3H, OCH_3_); ^13^C{^1^H} NMR (101 MHz, acetone-*d*_6_) δ 158.2 (Ar–C_q_), 158.0 (Ar–C_q_), 155.4 (Ar–C_q_), 137.1 (Ar–C_q_), 128.3 (Ar–C), 127.7 (2 × Ar–C), 122.4 (Ar–C_q_), 113.4 (2 × Ar–C), 106.6 (Ar–C), 103.5 (Ar–C), 75.2 (2 × S–CH_2_), 54.6 (OCH_3_), 34.9 (C_q_); HRMS(ES-ToF) *m*/*z* Calculated for C_16_H_17_O_5_S [M + H]^+^: 321.0797; Found: 321.0790.

#### 3-(2,4-Dimethoxyphenyl)-3-(4-methoxyphenyl)thietane 1,1-dioxide (**3al**)

Performed using general procedure B with thietanol dioxide **2a** (45.7 mg, 0.20 mmol, 1 equiv) and 1,3-dimethoxybenzene (84.7 mg, 0.60 mmol, 3.0 equiv), with the reaction conducted at 40 °C. Purification by flash column chromatography (25–30% EtOAc/hexane) afforded diarylthietane dioxide **3al** as a pale pink oil (68.4 mg, 98%). R_f_ = 0.20 (35% EtOAc/hexane); IR (film)/cm^–1^ 3001, 2960, 2837, 1608, 1582, 1508, 1461, 1416, 1393, 1311 (SO_2_), 1252, 1208, 1185, 1156, 1126, 1030, 970, 912, 830, 804, 731, 641, 543, 503; ^1^H NMR (400 MHz, CDCl_3_) δ 7.26–7.24 (2 H, d, *J* = 8.8 Hz, 2 × Ar–C), 7.11–7.09 (1 H, d, *J* = 8.5 Hz, Ar–H), 6.83–6.81 (2 H, d, *J* = 8.8 Hz, 2 × Ar–H), 6.53–6.50 (1 H, dd, *J* = 8.5, 2.3 Hz, Ar–H), 6.46 (1 H, d, *J* = 2.3, Ar–H), 4.86–4.82 (2 H, d, *J* = 14.6 Hz, C*H*H–SO_2_–C*H*H), 4.74–4.70 (2 H, d, *J* = 14.6 Hz, CH*H*–SO_2_–CH*H*), 3.82 (3 H, s, OCH_3_), 3.78 (3 H, s, OCH_3_), 3.70 (3 H, s, OCH_3_); ^13^C{^1^H} NMR (101 MHz, CDCl_3_) δ 160.7 (Ar–C_q_OMe), 158.2 (Ar–C_q_OMe), 157.7 (Ar–C_q_OMe), 136.2 (Ar–C_q_), 127.8 (Ar–C), 127.3 (2 × Ar–C), 124.3 (Ar–C_q_), 113.8 (2 × Ar–C), 104.0 (Ar–C), 99.8 (Ar–C), 75.7 (CH_2_–SO_2_–CH_2_), 55.4 (OCH_3_), 55.3 (OCH_3_), 55.2 (OCH_3_), 35.0 (C_q_); HRMS (ESI) *m*/*z* Calculated for C_18_H_21_O_5_S [M + H]: 349.1110, Found: 349.1110.

#### 3-(4-Methoxyphenyl)-3-(2,4,6-trimethoxyphenyl)thietane 1,1-dioxide (**3am**)

Performed using general procedure B with thietanol dioxide **2a** (45.7 mg, 0.20 mmol, 1 equiv) and 1,3,5-trimethoxybenzene (101 mg, 0.60 mmol, 3 equiv), with the reaction conduct at 40 °C. Purification by flash column chromatography (1–10% Et_2_O/CH_2_Cl_2_) afforded diarylthietane dioxide **3am** as a white solid (68.8 mg, 91%). R_f_ = 0.29 (5% Et_2_O/CH_2_Cl_2_); mp = 198–206 °C; IR (film)/cm^–1^ 2942, 2836, 1608, 1585, 1459, 1414, 1296, 1234, 1117, 1035, 968, 817, 633, 549, 522; ^1^H NMR (400 MHz, CDCl_3_) δ 7.48 (d, *J* = 8.6 Hz, 2H, Ar–H), 6.83 (d, *J* = 8.6 Hz, 2H, Ar–H), 6.15 (s, 2H, 2 × Ar–H), 4.93–4.70 (m, 4H, 2 × C*H*H–SO_2_–C*H*H), 3.81 (d, *J* = 3.5 Hz, 9H, 3 × O–CH_3_), 3.78 (s, 3H, O–CH_3_); ^13^C{^1^H} NMR (101 MHz, CDCl_3_) δ 160.6 (Ar–C_q_), 158.3 (Ar–C_q_), 158.0 (2 × Ar–C_q_), 136.1 (Ar–C_q_), 127.1 (2 × Ar–C), 113.7 (2 × Ar–C), 113.6 (Ar–C_q_), 91.5 (2 × Ar–C), 76.0 (2 × SO_2_–CH_2_), 55.7 (2 × O–CH_3_), 55.4 (O–CH_3_), 55.2 (O–CH_3_), 34.2 (C_q_); HRMS(ESI) *m*/*z* Calculated for C_19_H_23_O_6_S [M + H]^+^: 379.1215; Found: 379.1201.

#### 3-(4-Methoxyphenyl)-3-(1-methyl-1*H*-indol-3-yl)thietane 1,1-dioxide (**3an**)

Performed using general procedure B with thietanol dioxide **2a** (45.7 mg, 0.20 mmol, 1 equiv) and *N*-methyl indole (0.075 mL, 0.60 mmol, 3 equiv). Purification by flash column chromatography (2–5% Et_2_O/CH_2_Cl_2_) afforded diarylthietane dioxide **3an** as a white solid (37.5 mg, 55%). R_f_ = 0.56 (10% Et_2_O/CH_2_Cl_2_); mp = 185–188 °C; IR (film)/cm^–1^ 3021, 1609, 1514, 1316, 1256, 1219, 1127, 1101, 1022, 825, 749, 642, 549; ^1^H NMR (400 MHz, CDCl_3_) δ 7.37–7.31 (m, 3H, Ar–H), 7.28–7.24 (m, 1H, Ar–H), 7.22 (d, *J* = 8.1 Hz, 1H, Ar–H), 7.05 (ddd, *J* = 8.0, 6.9, 1.0 Hz, 1H, Ar–H), 6.92 (s, 1H, Ar–H), 6.86 (d, *J* = 8.9 Hz, 2H, Ar–H), 4.95–4.79 (m, 4H, 2 × C*H*H–SO_2_–C*H*H), 3.79 (s, 6H, O–CH_3_ and N–CH_3_); ^13^C{^1^H} NMR (101 MHz, CDCl_3_) δ 158.6 (Ar–C_q_), 138.1 (Ar–C_q_), 135.9 (Ar–C_q_), 127.7 (2 × Ar–C), 127.3 (Ar–C), 125.3 (Ar–C_q_), 122.3 (Ar–C), 119.7 (Ar–C), 119.7 (Ar–C), 117.8 (Ar–C_q_), 114.0 (2 × Ar–C), 109.9 (Ar–C), 77.0 (2 × SO_2_–CH_2_), 55.3 (O–CH_3_), 32.9 (N–CH_3_), 31.8 (C_q_); HRMS (ESI) *m*/*z* Calculated for C_19_H_20_O_3_SN [M + H]^+^: 342.1164; Found: 342.1164.

#### 3-(4-Methoxyphenyl)-3-(5-methylfuran-2-yl)thietane 1,1-dioxide (**3ao**)

Performed using general procedure B with thietanol dioxide **2a** (45.7 mg, 0.20 mmol, 1 equiv) and 2-methylfuran (54.1 μL, 0.60 mmol, 3 equiv). Purification by flash column chromatography (10% acetone/pentane) afforded diarylthietane dioxide **3ao** as a brown oil (35.1 mg, 60%); R_f_ = 0.26 (20% acetone/pentane); IR (film)/cm^–1^ 2959, 2837, 1609, 1512, 1321, 1251, 1211, 1133, 1027, 912, 831, 781, 731, 542, 493; ^1^H NMR (400 MHz, CDCl_3_) δ 7.20 (d, *J* = 8.8 Hz, 2H, Ar–H), 6.90 (d, *J* = 8.8 Hz, 2H, Ar–H), 5.93 (d, *J* = 3.1 Hz, 1H, CH_furan_), 5.89 (d, *J* = 1.6 Hz, 1H, CH_furan_), 4.81 (d, *J* = 14.1 Hz, 2H, C*H*H–SO_2_–C*H*H), 4.74–4.61 (m, 2H, CH*H*–SO_2_–CH*H*), 3.81 (s, 3H, OCH_3_), 2.26 (s, 3H, CH_3_); ^13^C{^1^H} NMR (101 MHz, CDCl_3_) δ 158.9 (Ar–C_q_), 153.2 (Ar–C_q_), 153.0 (Ar–C_q_), 133.6 (Ar–C_q_), 127.6 (2 × Ar–C), 114.2 (2 × Ar–C), 108.8 (C_furan_), 106.5 (C_furan_), 75.3 (CH_2_–SO_2_–CH_2_), 55.3 (OCH_3_), 33.0 (C_q_), 13.6 (CH_3_); HRMS (APCI) *m*/*z* Calculated for C_15_H_17_SO_4_^+^ [M + H]^+^: 293.0842, Found: 293.0829.

#### 3-(4-Methoxyphenyl)-3-(5-methylthiophen-2-yl)thietane 1,1-dioxide (**3ap**)

Performed using general procedure B with thietanol dioxide **2a** (45.7 mg, 0.20 mmol, 1 equiv) and 2-methylthiophene (53.3 μL, 0.60 mmol, 3 equiv). Purification by flash column chromatography (10% acetone/pentane) afforded diarylthietane dioxide **3ap** as a brown oil (30.9 mg, 50%). R_f_ = 0.26 (20% acetone/pentane); IR (film)/cm^–1^ 3023, 2958, 2837, 1609, 1512, 1321, 1252, 1222, 1184, 1130, 1031, 831, 802, 557, 536, 484; ^1^H NMR (400 MHz, CDCl_3_) δ 7.21 (d, *J* = 8.8 Hz, 2H, Ar–H), 6.90 (d, *J* = 8.8 Hz, 2H, Ar–H), 6.80 (d, *J* = 3.5 Hz, 1H, CH_thiophene_), 6.57 (d, *J* = 3.0 Hz, 1H, CH_thiophene_), 4.85 (d, *J* = 13.8 Hz, 2H, C*H*H–SO_2_–C*H*H), 4.78 (d, *J* = 13.9 Hz, 2H, CH*H*–SO_2_–CH*H*), 3.81 (s, 3H, OCH_3_), 2.39 (d, *J* = 1.1 Hz, 3H, CH_3_); ^13^C{^1^H} NMR (101 MHz, CDCl_3_) δ 158.9 (Ar–C_q_), 147.6 (Ar–C_q_), 140.6 (Ar–C_q_), 136.1 (Ar–C_q_), 127.5 (2 × Ar–C), 125.0 (C_thiophene_), 124.9 (C_thiophene_), 114.2 (2 × Ar–C), 77.7 (CH_2_–SO_2_–CH_2_), 55.3 (OCH_3_), 34.5 (C_q_), 15.3 (CH_3_); HRMS (APCI) *m*/*z* Calculated for C_15_H_16_S_2_O_3_ [M + H]^+^: 309.0614; Found: 309.0611.

#### 3-(4-Hydroxy-3-methylphenyl)-3-(2-methoxyphenyl)thietane 1,1-dioxide (**3ba**)

Performed using general procedure B with thietanol dioxide **2b** (45.7 mg, 0.20 mmol, 1 equiv) and *o*-cresol (62.0 μL, 0.60 mmol, 3 equiv). Purification by flash column chromatography (10% EtOAc/pentane) afforded diarylthietane dioxide **3ba** as a white solid (62.2 mg, 66%). R_f_ = 0.10 (10% EtOAc/pentane); mp = 192–195 °C; IR (film)/cm^–1^ 3391 (OH), 3043, 2946, 1600, 1510, 1488, 1451, 1429, 1289, 1232, 1195, 1168, 1128, 1105, 1054, 1020, 972, 809, 759, 614, 496; ^1^H NMR (400 MHz, CDCl_3_) δ 7.30 (ddd, *J* = 8.2, 7.7, 1.7 Hz, 1H, Ar–H), 7.17 (dd, *J* = 7.7, 1.7 Hz, 1H, Ar–H), 7.07 (d, *J* = 2.6 Hz, 1H, Ar–H), 7.05–6.96 (m, 2H, 2 × Ar–H), 6.88 (dd, *J* = 8.2, 1.1 Hz, 1H, Ar–H), 6.62 (d, *J* = 8.4 Hz, 1H, Ar–H), 5.00 (s, 1H, OH), 4.86 (d, *J* = 14.8 Hz, 2H, C*H*H–S–C*H*H), 4.76 (d, *J* = 14.8 Hz, 2H, CH*H*–S–CH*H*), 3.74 (s, 3H, OCH_3_), 2.17 (s, 3H, CH_3_); ^13^C{^1^H} NMR (101 MHz, CDCl_3_) δ 156.6 (Ar–C_q_), 152.8 (Ar–C_q_), 135.3 (Ar–C_q_), 132.0 (Ar–C_q_), 129.3 (Ar–C), 129.0 (Ar–C), 127.3 (Ar–C), 125.0 (Ar–C), 123.9 (Ar–C_q_), 120.7 (Ar–C), 114.7 (Ar–C), 111.9 (Ar–C), 75.3 (CHH–S–CHH), 55.2 (OCH_3_), 35.6 (C_q_), 16.1 (CH_3_); HRMS (ESI) *m*/*z* Calculated for C_17_H_19_O_4_S [M + H]^+^: 319.1009; Found: 319.1028.

#### 3-(4-Hydroxy-3-methylphenyl)-3-(3-methoxyphenyl)thietane 1,1-dioxide (**3ca**)

Performed using general procedure B with thietanol dioxide **2c** (45.7 mg, 0.20 mmol, 1 equiv) and *o*-cresol (62.0 μL, 0.60 mmol, 3 equiv). Purification by flash column chromatography (20% EtOAc/pentane) afforded diarylthietane dioxide **3ca** as a white solid (34.9 mg, 55%). R_f_ = 0.15 (30% EtOAc/pentane); mp = 169–172 °C; IR (film)/cm^–1^ 3444 (OH), 3024, 2958, 2838, 1664, 1585, 1510, 1489, 1431l, 1316, 1271, 1221, 1126, 1050, 814, 780, 731, 481, 445; ^1^H NMR (400 MHz, CDCl_3_) δ 7.29 (d, *J* = 7.9 Hz, 1H, Ar–H), 7.00 (d, *J* = 2.7 Hz, 1H, Ar–H), 6.95 (dd, *J* = 8.4, 2.7 Hz, 1H, Ar–H), 6.86 (ddd, *J* = 7.9, 2.0, 0.9 Hz, 1H, Ar–H), 6.82–6.77 (m, 1H, Ar–H), 6.76 (dd, *J* = 2.2, 0.9 Hz, 1H, Ar–H), 6.71 (d, *J* = 8.4 Hz, 1H, Ar–H), 4.96 (s, 1H, OH), 4.83 (s, 4H, CHH–S–CHH), 3.78 (s, 3H, OCH_3_), 2.21 (s, 3H, CH_3_); ^13^C{^1^H} NMR (101 MHz, CDCl_3_) δ 159.9 (Ar–C_q_), 153.1 (Ar–C_q_), 146.6 (Ar–C_q_), 136.2 (Ar–C_q_), 130.1 (Ar–C), 129.3 (Ar–C), 125.3 (Ar–C), 124.5 (Ar–C_q_), 118.7 (Ar–C), 115.1 (Ar–C), 113.4 (Ar–C), 111.8 (Ar–C), 76.6 (CHH–S–CHH), 55.3 (OCH_3_), 37.0 (C_q_), 16.1 (CH_3_); HRMS (ESI) *m*/*z* Calculated for C_17_H_19_O_4_S [M + H]^+^: 319.1004; Found: 319.1009.

#### 3-(Benzo[*d*][1,3]dioxol-5-yl)-3-(4-hydroxy-3-methylphenyl)thietane 1,1-dioxide (**3da**)

Performed using general procedure B with thietanol dioxide **2d** (48.4 mg, 0.20 mmol, 1 equiv) and *o*-cresol (62.0 μL, 0.60 mmol, 3 equiv). Purification by flash column chromatography (1:1:5 Et_2_O/CH_2_Cl_2_/pentane) afforded diarylthietane dioxide **3da** as colorless oil (55.2 mg, 83%). R_f_ = 0.19 (1:1:5 Et_2_O/CH_2_Cl_2_/pentane); IR (film)/cm^–1^ 3431 (OH), 3023, 1609, 1503, 1484, 1436, 1313, 1238, 1212, 1149, 1112, 1034, 930, 906, 809, 769, 727, 596, 473, 439; ^1^H NMR (400 MHz, CDCl_3_) δ 7.08–6.88 (m, 2H, Ar–H), 6.83–6.63 (m, 4H, Ar–H), 5.95 (s, 2H, O–CH_2_–O), 4.97 (s, 1H, OH), 4.87–4.70 (m, 4H, CHH–SO_2_–CHH), 2.22 (s, 3H, CH_3_); ^13^C{^1^H} NMR (101 MHz, CDCl_3_) δ 153.0 (Ar–C_q_), 148.3 (Ar–C_q_), 146.7 (Ar–C_q_), 138.8 (Ar–C_q_), 136.5 (Ar–C_q_), 129.2 (Ar–C), 125.2 (Ar–C), 124.5 (Ar–C_q_), 119.6 (Ar–C), 115.1 (Ar–C), 108.2 (Ar–C), 107.4 (Ar–C), 101.4 (O–CH_2_–O), 76.6 (CHH–SO_2_–CHH), 36.9 (C_q_), 16.0 (CH_3_); HRMS (APCI) *m*/*z* Calculated for C_17_H_15_O_5_S [M – H]^−^:331.0646; Found: 331.0641.

#### 3-(4-Hydroxy-3-methylphenyl)-3-(4-((triisopropylsilyl)oxy)phenyl)thietane 1,1-dioxide (**3ea**)

Performed using general procedure B with thietanol dioxide **2e** (74.2 mg, 0.20 mmol, 1 equiv) and *o*-cresol (62.0 μL, 0.60 mmol, 3 equiv). Purification by flash column chromatography (20% EtOAc/pentane) afforded diarylthietane dioxide **3ea** as a white solid (59.9 mg, 65%). R_f_ = 0.41 (30% EtOAc/pentane); mp = 124–127 °C; IR (film)/cm^–1^ 3456, 2945, 2866, 1606, 1510, 1463, 1318, 1271, 1178, 1128, 914, 883, 837, 685; ^1^H NMR (400 MHz, CDCl_3_) δ 7.08 (d, *J* = 8.3 Hz, 2H, Ar–H), 6.95 (d, *J* = 6.8 Hz, 2H, Ar–H), 6.83 (d, *J* = 8.3 Hz, 2H, Ar–H), 6.70 (d, *J* = 8.4 Hz, 1H, Ar–H), 4.82 (s, 4H, CHH–SO_2_–CHH), 2.20 (s, 3H, CH_3_), 1.24 (hept, *J* = 7.3 Hz, 3H, CH), 1.09 (d, *J* = 7.3 Hz, 18H, CH_3_); ^13^C{^1^H} NMR (101 MHz, CDCl_3_) δ 155.2 (Ar–C_q_), 153.0 (Ar–C_q_), 137.4 (Ar–C_q_), 136.7 (Ar–C_q_), 129.5 (Ar–C), 127.7 (2 × Ar–C), 125.4 (Ar–C), 124.4 (Ar–C_q_), 120.1 (2 × Ar–C), 115.1 (Ar–C), 77.1 (CHH–SO_2_–CHH), 36.3 (C_q_), 17.9 (6 × CH_3_), 16.0 (CH_3_), 12.6 (3 × CH); HRMS (APCI) *m*/*z* Calculated for C_25_H_37_O_4_SSi [M + H]^+^:461.2176; Found: 461.2171.

#### 3-(4-Hydroxy-3-methylphenyl)-3-(*p*-tolyl)thietane 1,1-dioxide (**3fa**)

Performed using general procedure B with thietanol dioxide **2f** (42.4 mg, 0.20 mmol, 1 equiv) and *o*-cresol (62.0 μL, 0.60 mmol, 3 equiv). Purification by flash column chromatography (20% EtOAc/pentane) afforded diarylthietane dioxide **3fa** as a white solid (55.6 mg, 92%). R_f_ = 0.24 (30% EtOAc/pentane); mp = 159–163 °C; IR (film)/cm^–1^ 3412 (OH), 2961, 2920, 1609, 1510, 1312, 1272, 1220, 1123, 915, 818, 732, 594, 473; ^1^H NMR (400 MHz, CDCl_3_) δ 7.14 (m, 4H, 4 × Ar–H), 6.99 (d, *J* = 2.6 Hz, 1H, Ar–H), 6.93 (dd, *J* = 8.3, 2.7 Hz, 1H, Ar–H), 6.67 (d, *J* = 8.3 Hz, 1H, Ar–H), 5.21 (s, 1H, OH), 4.83 (m, 4H, CHH–S–CHH), 2.32 (s, 3H, CH_3_), 2.19 (s, 3H, CH_3_); ^13^C{^1^H} NMR (101 MHz, CDCl_3_) δ 153.0 (Ar–C_q_), 142.0 (Ar–C_q_), 137.0 (Ar–C_q_), 136.2 (Ar–C_q_), 129.6 (2 × Ar–C), 129.2 (Ar–C), 126.2 (2 × Ar–C), 125.1 (Ar–C), 124.5 (Ar–C_q_), 115.1 (Ar–C), 76.6 (2 × S–CH_2_), 36.7 (C_q_), 20.9 (CH_3_), 16.0 (CH_3_); HRMS(ESI) *m*/*z* Calculated for C_17_H_19_O_3_S [M + H]^+^: 303.1055; Found: 303.1057.

#### 3-(4-Hydroxy-3-methylphenyl)-3-phenylthietane 1,1-dioxide (**3ga**)

Performed using general procedure B with thietanol dioxide **2g** (39.6 mg, 0.20 mmol, 1 equiv) and *o*-cresol (62.0 μL, 0.60 mmol, 3 equiv). Purification by flash column chromatography (20% EtOAc/pentane) afforded diarylthietane dioxide **3ga** as a white solid (33.3 mg, 73%). R_f_ = 0.15 (30% EtOAc/pentane); mp = 171–173 °C; IR (film)/cm^–1^ 3433 (OH), 3015, 2952, 1504, 1446, 1306, 1271, 1217, 1197, 1169, 1120, 1103, 703, 557, 531, 468, 447, 412; ^1^H NMR (400 MHz, acetone-*d*_6_) δ 8.3 (s, 1H, Ar–H), 7.50–7.45 (m, 2H, 2 × Ar–H), 7.41–7.32 (m, 2H, 2 × Ar–H), 7.29–7.21 (m, 2H, 2 × Ar–H), 7.12 (dd, *J* = 8.3, 2.7 Hz, 1H, Ar–H), 6.80 (d, *J* = 8.3 Hz, 1H, Ar–H), 4.92 (s, 4H, CHH–S–CHH), 2.19 (s, 3H, CH_3_); ^13^C{^1^H} NMR (101 MHz, acetone-*d*_6_) δ 154.2 (Ar–C_q_), 146.3 (Ar–C_q_), 136.3 (Ar–C_q_), 129.1 (Ar–C), 128.6 (2 × Ar–C), 126.6 (Ar–C), 126.4 (2 × Ar–C), 125.0 Ar–C), 124.6 (Ar–C_q_), 114.6 (Ar–C), 75.6 (2 × S–CH_2_), 37.2 (C_q_), 15.5 (CH_3_); HRMS(ESI) *m*/*z* Calculated for C_16_H_17_O_3_S [M + H]^+^: 289.0898; Found: 289.0896.

#### 3-(4-Chlorophenyl)-3-(4-hydroxy-3-methylphenyl)thietane 1,1-dioxide (**3ha**)

Performed using general procedure B with thietanol dioxide **2h** (46.5 mg, 0.20 mmol, 1 equiv) and *o*-cresol (62.0 μL, 0.60 mmol, 3 equiv). Purification by flash column chromatography (20% EtOAc/pentane) afforded diarylthietane dioxide **3ha** as a white solid (41.9 mg, 65%). R_f_ = 0.15 (30% EtOAc/pentane); mp = 159–163 °C; IR (film)/cm^–1^ 3393 (OH), 1610, 1510, 1493, 1394, 1308, 1265, 1246, 1217, 1128, 1090, 1016, 829, 811, 783, 593, 412; ^1^H NMR (400 MHz, acetone-*d*_6_) δ 7.48 (d, *J* = 8.4 Hz, 2H, 2 × Ar–H), 7.36 (d, *J* = 8.4 Hz, 2H, 2 × Ar–H), 7.21 (d, *J* = 2.6 Hz, 1H, Ar–H), 7.10 (dd, *J* = 8.3, 2.6 Hz, 1H, Ar–H), 6.80 (d, *J* = 8.3 Hz, 1H, Ar–H), 4.90 (s, 4H, CHH–S–CHH), 2.17 (s, 3H, CH_3_); ^13^C{^1^H} NMR (101 MHz, acetone-*d*_6_) δ 154.4 (Ar–C_q_), 145.2 (Ar–C_q_), 135.7 (Ar–C_q_), 132.1 (Ar–C_q_), 129.1 (Ar–C), 128.6 (2 × Ar–C), 128.4 (2 × Ar–C), 125.0 (Ar–C), 124.7 (Ar–C_q_), 114.7 (Ar–C), 75.5 (2 × S–CH_2_), 37.1 (CH_3_), 15.5 (C_q_); HRMS(ESI) *m*/*z* Calculated for C_16_H_12_^35^ClO_3_S [M – H]^−^: 321.0358; Found: 321.0358.

#### 3-(2,4-Dimethoxyphenyl)-3-(4-((triisopropylsilyl)oxy)phenyl)thietane 1,1-dioxide (**3el**)

Performed using general procedure B with thietanol dioxide **2e** (74.2 mg, 0.20 mmol, 1 equiv) and 1,3-dimethoxy benzene (79.0 μL, 0.60 mmol, 3 equiv). Purification by flash column chromatography (10% EtOAc/pentane) afforded diarylthietane dioxide **3el** as a white solid (56.9 mg, 58%). R_f_ = 0.10 (10% EtOAc/pentane); mp = 112–115 °C; IR (film)/cm^–1^ 2942, 2865, 1607, 1581, 1507, 1462, 1317, 1209, 1128, 1233, 913, 834, 684; ^1^H NMR (400 MHz, CDCl_3_) δ 7.13 (d, *J* = 8.5 Hz, 2H, Ar–H), 7.09 (d, *J* = 8.5 Hz, 1H, Ar–H), 6.78 (d, *J* = 8.5 Hz, 2H, Ar–H), 6.51 (dd, *J* = 8.5, 2.5 Hz, 1H, Ar–H), 6.45 (d, *J* = 2.4 Hz, 1H, Ar–H), 4.82 (d, *J* = 14.6 Hz, 2H, C*H*H–SO_2_–C*H*H), 4.70 (d, *J* = 14.6 Hz, 2H, CH*H*–SO_2_–CH*H*), 3.81 (s, 3H, OCH_3_), 3.65 (s, 3H, OCH_3_), 1.22 (hept, *J* = 13.7, 6.6 Hz, 3H, CH), 1.07 (d, *J* = 7.3 Hz, 18H, CH_3_); ^13^C{^1^H} NMR (101 MHz, CDCl_3_) δ 161.0 (Ar–C_q_), 158.1 (Ar–C_q_), 155.1 (Ar–C_q_), 137.1 (Ar–C_q_), 128.2 (Ar–C), 127.5 (2 × Ar–C), 124.6 (Ar–C_q_), 120.0 (2 × Ar–C), 104.2 (Ar–C), 100.2 (Ar–C), 76.1 (CHH–SO_2_–CHH), 55.7 (OCH_3_), 55.5 (OCH_3_), 35.1 (C_q_), 18.2 (6 × CH_3_), 12.9 (3 × CH); HRMS (APCI) *m*/*z* Calculated for C_26_H_39_O_5_SSi [M + H]^+^:491.2282; Found: 491.2281.

#### 3-(4-Chlorophenyl)-3-(2,4-dimethoxyphenyl)thietane 1,1-dioxide (**3hl**)

Performed using general procedure B with thietanol dioxide **2h** (46.5 mg, 0.20 mmol, 1 equiv) and 1,3-dimethoxybenzene (39.2 μL, 0.60 mmol, 3 equiv). Purification by flash column chromatography (20% EtOAc/pentane) afforded diarylthietane dioxide **3hl** as a white solid (44.5 mg, 63%). R_f_ = 0.15 (30% EtOAc/pentane); mp = 147–149 °C; IR (film)/cm^–1^ 2961, 2838, 1608, 1582, 1504, 1465, 1437, 1416, 1312, 1210, 1128, 1029, 971, 911, 826, 729, 527; ^1^H NMR (400 MHz, CDCl_3_) δ 7.26 (s, 4H, 4 × Ar–H), 7.13 (d, *J* = 8.4 Hz, 1H, Ar–H), 6.53 (d, *J* = 8.4 Hz, 1H, Ar–H), 6.45 (s, 1H, Ar–H), 4.82 (d, *J* = 16.6 Hz, 2H, 2 × Ar–H), 4.70 (d, *J* = 16.6 Hz, 2H, 2 × Ar–H), 3.82 (s, 3H, OCH_3_), 3.68 (s, 3H, OCH_3_); ^13^C{^1^H} NMR (101 MHz, CDCl_3_) δ 161.0 (Ar–C_q_), 157.6 (Ar–C_q_), 142.8 (Ar–C_q_), 132.8 (Ar–C_q_), 128.6 (2 × Ar–C), 127.7 (Ar–C), 127.6 (2 × Ar–C), 123.5 (Ar–C_q_), 104.2 (Ar–C), 99.9 (Ar–C) 75.5 (2 × S–CH_2_), 55.5 (OCH_3_), 55.4 (OCH_3_), 35.3 (C_q_); HRMS (ESI) *m*/*z* Calculated for C_17_H_18_O_4_S^35^Cl [M + H]^+^: 353.0614; Found: 353.0606.

#### 3-(4-Chlorophenyl)-3-(2,4,6-trimethoxyphenyl)thietane 1,1-dioxide (**3hm**)

Performed using general procedure B with thietanol dioxide **2h** (46.5 mg, 0.20 mmol, 1 equiv) and 1,3,5-trimethoxybenzene (101 mg, 0.60 mmol, 3 equiv). Purification by flash column chromatography (20% EtOAc/pentane) afforded diarylthietane dioxide **3hm** as a white solid (60.8 mg, 79%). R_f_ = 0.15 (30% EtOAc/pentane); mp = 234–238 °C; IR (film)/cm^–1^ 2971, 2939, 2842, 1608, 1588, 1459, 1299, 1120, 1060, 1034, 1012, 814, 524; ^1^H NMR (400 MHz, CDCl_3_) δ 7.47 (d, *J* = 8.5 Hz, 2H, 2 × Ar–H), 7.24 (d, *J* = 7.2 Hz, 2H, 2 × Ar–H), 6.12 (s, 2H, 2 × Ar–H), 4.82 (d, *J* = 16.3 Hz, 2H, C*H*H–S–C*H*H), 4.72 (d, *J* = 16.3 Hz, 2H, CH*H*–S–CH*H*), 3.80 (s, 3H, OCH_3_), 3.79 (s, 6H, 2 × OCH_3_); ^13^C{^1^H} NMR (101 MHz, CDCl_3_) δ 160.9 (Ar–C_q_), 157.9 (2 × Ar–C_q_), 142.5 (Ar–C_q_), 132.8 (Ar–C_q_), 128.5 (2 × Ar–C), 127.5 (2 × Ar–C), 112.7 (Ar–C_q_), 91.4 (2 × Ar–C), 75.8 (2 × S–CH_2_), 55.7 (2 × OCH_3_), 55.4 (OCH_3_), 34.6 (C_q_); HRMS (ESI) *m*/*z* Calculated for C_18_H_20_SO_5_^35^Cl [M + H]^+^: 383.0714; Found: 383.0723.

#### 3-(4-Methoxyphenyl)-2*H*-thiete 1,1-dioxide (**4a**)

Performed using general procedure B with thietanol dioxide **2a** (45.7 mg, 0.20 mmol, 1 equiv) without nucleophile. Purification by flash column chromatography (10% Et_2_O/pentane) afforded thiete dioxide **4a** as a white solid (33.6 mg, 80%). R_f_ = 0.47 (10% Et_2_O/CH_2_Cl_2_); mp = 191–193 °C; IR (film)/cm^–1^ 3102, 3078, 3018, 2998, 2968, 2945, 2838, 1606, 1564, 1508, 1427, 1277 (S=O), 1253, 1207, 1184, 1149, 1120, 1028, 927, 903, 841, 782, 673, 499, 479, 443; ^1^H NMR (400 MHz, CDCl_3_) δ 7.42–7.40 (2 H, d, *J* = 8.5 Hz, 2 × Ar–H), 6.98–6.96 (2 H, d, *J* = 8.5, 2 × Ar–H), 6.81 (1 H, s, C=CHSO_2_), 4.77 (2 H, s, CH_2_SO_2_), 3.88 (3 H, s, OCH_3_); ^13^C{^1^H} NMR (101 MHz, CDCl_3_) δ 162.7 (Ar–C_q_OMe), 146.7 (C_q_), 133.9 (C_q_=*C*HSO_2_), 129.4 (2 × Ar–C), 121.5 (C_q_), 114.6 (2 × Ar–C), 69.8 (CH_2_–SO_2_), 55.5 (OCH_3_). The observed characterization data (IR, ^1^H and ^13^C NMR) were consistent with that previously reported.^14^

### Thiol Alkylation with Thietan-3-ol Dioxide: General Procedure C

Calcium(II) bis(trifluoromethanesulfonimide) (6.0 mg, 0.01 mmol, 0.05 equiv) and tetrabutylammonium hexafluorophosphate (4.0 mg, 0.01 mmol, 0.05 equiv) were added sequentially to a solution of thietane-ol dioxide (0.20 mmol, 1 equiv) and thiol (0.60 mmol, 3 equiv) in toluene (0.4 mL, 0.5 M) in reaction vial. The reaction vial was sealed under argon, and the mixture was heated at 40 °C for 4.5 h then cooled to rt. Sat. aq. NaHCO_3_ (15 mL) was added followed by CH_2_Cl_2_ (15 mL). The phases were separated and the aqueous layer was extracted with CH_2_Cl_2_ (2 × 15 mL). The organic layers were combined, dried over Na_2_SO_4_, filtered and concentrated in vacuo. Purification by flash column chromatography afforded the thietane dioxide thioether.

#### 3-(4-Methoxyphenyl)-3-(*p*-tolylthio)thietane 1,1-dioxide (**5aa**)

Performed using general procedure C with thietanol dioxide **2a** (45.7 mg, 0.20 mmol, 1 equiv) and 4-methylbenzenethiol (41.4 mg, 0.60 mmol, 3 equiv). Purification by flash column chromatography (20% EtOAc/pentane) afforded 3-(4-methoxyphenyl)-3-(*p*-tolylthio)thietane 1,1-dioxide **5aa** as a white solid (55.1 mg, 82%). R_f_ = 0.30 (20% EtOAc/pentane); mp = 123–127 °C; ^1^H NMR (400 MHz, CDCl_3_) δ 7.06 (d, *J* = 7.8 Hz, 2H, Ar–H), 6.98 (d, *J* = 8.1 Hz, 2H, Ar–H), 6.90 (d, *J* = 8.8 Hz, 2H, Ar–H), 6.81 (d, *J* = 8.8 Hz, 2H, Ar–H), 4.61 (d, *J* = 14.4 Hz, 2H, C*H*H–S–C*H*H), 4.52 (d, *J* = 14.3 Hz, 2H, CH*H*–S–CH*H*), 3.81 (s, 3H, OCH_3_), 2.33 (s, 3H, CH_3_); ^13^C{^1^H} NMR (101 MHz, CDCl_3_) δ 159.0 (Ar–C_q_), 140.5 (Ar–C_q_), 136.6 (2 × Ar–C), 133.9 (Ar–C_q_), 129.9 (2 × Ar–C), 128.0 (2 × Ar–C), 127.3 (Ar–C_q_), 113.7 (2 × Ar–C), 75.5 (CH_2_–S–CH_2_), 55.3 (OCH_3_), 40.5 (C_q_), 21.3 (CH_3_). HRMS (APCI) *m*/*z* calculated for C_17_H_22_O_3_NS_2_ [M + NH_4_]^+^: 352.1036; Found 352.1039.

#### 3-((4-Bromophenyl)thio)-3-(4-methoxyphenyl)thietane 1,1-dioxide (**5ab**)

Performed using general procedure C with thietanol dioxide **2a** (45.7 mg, 0.20 mmol, 1 equiv) and 4-methylbenzenethiol (41.4 mg, 0.60 mmol, 3 equiv). Purification by flash column chromatography (20% EtOAc/pentane) afforded 3-(4-methoxyphenyl)-3-(*p*-tolylthio)thietane 1,1-dioxide **5ab** as a white solid (72.7 mg, 91%). R_f_ = 0.30 (20% EtOAc/pentane); mp = 123–127 °C; ^1^H NMR (400 MHz, CDCl_3_) δ 7.37 (d, *J* = 8.4 Hz, 2H, 2 × Ar–H), 6.90 (dd, *J* = 8.6, 7.7 Hz, 4H, 4 × Ar–H), 6.82 (d, *J* = 8.9 Hz, 2H, 2 × Ar–H), 4.63 (d, *J* = 14.7 Hz, 2H, C*H*H–S–C*H*H), 4.51 (d, *J* = 14.7 Hz, 2H, CH*H*–S–CH*H*), 3.82 (s, 3H, OCH_3_); ^13^C{^1^H} NMR (101 MHz, CDCl_3_) δ 159.1 (Ar–C_q_), 137.8 (2 × Ar–C), 133.5 (Ar–C_q_), 132.3 (2 × Ar–C), 129.8 (Ar–C_q_), 128.0 (2 × Ar–C), 125.1 (Ar–C_q_), 113.9 (2 × Ar–C), 75.7 (CHH–S–CHH), 55.4 (OCH_3_), 40.8 (C_q_); HRMS (APCI) *m*/*z* calculated for C_16_H_19_O_3_N^81^BrS_2_ [M + NH_4_]^+^: 417.9964; Found 417.9963.

#### 3-(Benzylthio)-3-(4-methoxyphenyl)thietane 1,1-dioxide (**5ac**)

Performed using general procedure C with thietanol dioxide **2a** (45.7 mg, 0.20 mmol, 1 equiv) and phenylmethanethiol (35.2 μL, 0.60 mmol, 3 equiv). Purification by flash column chromatography (20% EtOAc/pentane) afforded 3-(benzylthio)-3-(4-methoxyphenyl)thietane 1,1-dioxide **5ac** as a white solid (57.4 mg, 86%). R_f_ = 0.23 (20% EtOAc/pentane); mp = 121–124 °C; ^1^H NMR (400 MHz, CDCl_3_) δ 7.32–7.20 (m, 5H, Ar–H), 7.16 (d, *J* = 6.3 Hz, 2H, Ar–H), 6.95 (d, *J* = 8.8 Hz, 2H, Ar–H), 4.58 (d, *J* = 14.5 Hz, 2H, C*H*H–S–C*H*H), 4.33 (d, *J* = 14.6 Hz, 2H, CH*H*–S–CH*H*), 3.85 (s, 3H, O–CH_3_), 3.48 (s, 2H, S–CH_2_); ^13^C{^1^H} NMR (101 MHz, CDCl_3_) δ 159.1 (Ar–C_q_), 135.9 (Ar–C_q_), 132.5 (Ar–C_q_), 129.0 (2 × Ar–C), 128.7 (2 × Ar–C), 128.1 (2 × Ar–C), 127.5 (Ar–C), 114.1 (2 × Ar–C), 76.7 (CH_2_–S–CH_2_), 55.4 (OCH_3_), 38.3 (C_q_), 36.3 (S–CH_2_); HRMS (APCI) *m*/*z* calculated for C_17_H_22_O_3_NS_2_ [M + NH_4_]^+^: 352.1036; Found 352.1034.

#### Methyl 3-((3-(4-methoxyphenyl)-1,1-dioxidothietan-3-yl)thio)propanoate (**5ad**)

Performed using general procedure C with thietanol dioxide **2a** (45.7 mg, 0.20 mmol, 1 equiv) and methyl 3-mercaptopropanoate (33.2 μL, 0.60 mmol, 3 equiv). Purification by flash column chromatography (20% EtOAc/pentane) afforded 3-(benzylthio)-3-(4-methoxyphenyl)thietane 1,1-dioxide **5ad** as a light yellow gum (60.4 mg, 91%). R_f_ = 0.20 (30% EtOAc/pentane); ^1^H NMR (400 MHz, CDCl_3_) δ 7.22 (d, *J* = 8.8 Hz, 2H, Ar–H), 6.89 (d, *J* = 8.8 Hz, 2H, Ar–H), 4.73 (d, *J* = 14.6 Hz, 2H, C*H*H–S–C*H*H), 4.50 (d, *J* = 14.7 Hz, 2H, CH*H*–S–CH*H*), 3.80 (s, 3H, OCH_3_), 3.63 (s, 3H, OCH_3_), 2.53 (t, *J* = 7.3 Hz, 2H, CH_2_), 2.30 (t, *J* = 7.3 Hz, 2H, CH_2_); ^13^C{^1^H} NMR (101 MHz, CDCl_3_) δ 171.8 (C=O), 159.2 (Ar–C_q_), 133.0 (Ar–C_q_), 127.8 (2 × Ar–C), 114.3 (2 × Ar–C), 76.8 (2 × S–CH_2_), 55.4 (OCH_3_), 52.0 (OCH_3_), 38.1 (C_q_), 32.9 (CH_2_), 26.6 (CH_2_); HRMS (APCI) *m*/*z* calculated for C_14_H_22_O_5_NS_2_ [M + NH_4_]^+^: 348.0934; Found 348.0937.

#### 3-(((3*s*,5*s*,7*s*)-Adamantan-1-yl)thio)-3-(4-methoxyphenyl)thietane 1,1-dioxide (**5ae**)

Performed using general procedure C with thietanol dioxide **2a** (45.7 mg, 0.20 mmol, 1 equiv) and 1-adamantanethiol (101 mg, 0.60 mmol, 3 equiv). Purification by flash column chromatography (20% EtOAc/pentane) afforded 3-(((3s,5s,7s)-adamantan-1-yl)thio)-3-(4-methoxyphenyl)thietane 1,1-dioxide **5ae** as a white solid (54.5 mg, 72%). R_f_ = 0.20 (30% EtOAc/pentane); mp = 163–167 °C; IR (film)/cm^–1^ 2903, 2848, 1720, 1608, 1511, 1451, 1325, 1254, 1213, 1182, 1100, 1031, 826, 731, 546, 498; ^1^H NMR (400 MHz, CDCl_3_) δ 7.40 (d, *J* = 8.8 Hz, 2H, 2 × Ar–H), 6.88 (d, *J* = 8.8 Hz, 2H, 2 × Ar–H), 4.75 (d, *J* = 14.5 Hz, 2H, C*H*H–S–C*H*H), 4.59 (d, *J* = 14.5 Hz, 2H, CH*H*–S–CH*H*), 3.82 (s, 3H, OCH_3_), 1.87 (s, 3H, 3 × CH), 1.63–1.44 (m, 12H, 6 × CH_2_); ^13^C{^1^H} NMR (101 MHz, CDCl_3_) δ 159.0 (Ar–C_q_), 134.3 (Ar–C_q_), 128.4 (2 × Ar–C), 113.8 (2 × Ar–C), 78.7 (CHH–S–CHH), 55.4 (OCH_3_), 50.4 (C_q_), 43.2 (3 × CH_2_), 37.8 (C_q_), 35.9 (3 × CH_2_), 29.5 (3 × CH); HRMS (TOF) *m*/*z* calculated for C_20_H_30_O_3_NS_2_ [M + NH_4_]^+^: 396.1667; Found 396.1679.

#### 3-((4-Bromophenyl)thio)-3-(4-chlorophenyl)thietane 1,1-dioxide (**5hb**)

Performed using general procedure C with thietanol dioxide **2h** (46.5 mg, 0.20 mmol, 1 equiv) and 4-bromothiophenol (56.7 mg, 0.60 mmol, 3 equiv), with the reaction conduct at 110 °C. Purification by flash column chromatography (20% EtOAc/pentane) afforded 3-((4-bromophenyl)thio)-3-(4-chlorophenyl)thietane 1,1-dioxide **5hb** as a light yellow gum (52.5 mg, 65%). R_f_ = 0.20 (30% EtOAc/pentane); IR (film)/cm^–1^ 3013, 2947, 1564, 1491, 1470, 1388, 1323, 1213, 1135, 1090, 1068, 1010, 908, 820, 770, 731, 490, 431; ^1^H NMR (400 MHz, CDCl_3_) δ 7.40 (d, *J* = 8.3 Hz, 2H, 2 × Ar–C), 7.29 (d, *J* = 7.2 Hz, 2H, 2 × Ar–C), 6.91 (t, *J* = 7.3 Hz, 4H, 4 × Ar–C), 4.62 (d, *J* = 13.0 Hz, 2H, C*H*H–S–C*H*H), 4.52 (d, *J* = 13.0 Hz, 2H, CH*H*–S–CH*H*); ^13^C{^1^H} NMR (101 MHz, CDCl_3_) δ 140.2 (Ar–C_q_), 137.9 (2 × Ar–C), 137.8 (Ar–C_q_), 134.2 (2 × Ar–C), 132.6 (2 × Ar–C), 128.9 (Ar–C_q_), 128.1 (Ar–C), 125.6 (Ar–C_q_), 75.5 (2 × S–CH_2_), 40.8 (C_q_); HRMS (ESI) *m*/*z* calculated for C_15_H_12_^79^Br^35^ClO_2_S_2_ [M + Na]^+^: 424.9043; Found 424.9048.

#### Methyl 3-((3-(4-chlorophenyl)-1,1-dioxidothietan-3-yl)thio)propanoate (**5hd**)

Performed using general procedure C with thietanol dioxide **2h** (46.5 mg, 0.20 mmol, 1 equiv) and methyl 3-mercaptopropanoate (33.2 μL, 0.60 mmol, 3 equiv), with the reaction conduct at 110 °C. Purification by flash column chromatography (20% EtOAc/pentane) afforded methyl 3-((3-(4-chlorophenyl)-1,1-dioxidothietan-3-yl)thio)propanoate **5hd** as a light yellow gum (31.5 mg, 47%). R_f_ = 0.20 (30% EtOAc/pentane); IR (film)/cm^–1^ 3019, 2952, 1731 (C=O), 1492, 1402, 1362, 1321, 1249, 1217, 1172, 1135, 1092, 1012, 829, 771, 531, 436; ^1^H NMR (400 MHz, CDCl_3_) δ 7.38 (d, *J* = 6.8 Hz, 2H, 2 × Ar–H), 7.27 (d, *J* = 7.7 Hz, 2H, 2 × Ar–H), 4.72 (d, *J* = 13.2 Hz, 2H, C*H*H–S–C*H*H), 4.53 (d, *J* = 13.2 Hz, 2H, CH*H*–S–CH*H*), 3.66 (s, 3H, OCH_3_), 2.55 (t, *J* = 7.3 Hz, 2H, CH_2_), 2.36 (t, *J* = 7.3 Hz, 2H, CH_2_); ^13^C{^1^H} NMR (101 MHz, CDCl_3_) δ 171.6 (C=O), 139.7 (Ar–C_q_), 134.3 (Ar–C_q_), 129.2 (2 × Ar–C), 127.9 (2 × Ar–C), 76.5 (2 × S–CH_2_), 38.1 (C_q_), 32.7 (CH_2_), 26.5 (CH_2_); HRMS (ESI) *m*/*z* Calculated for C_13_H_15_O_4_S_2_^35^Cl [M – H]^−^: 333.0028; Found: 333.0031.

### Alcohol Alkylation with Thietan-3-ol Dioxide: General Procedure D

Alcohol (1.25 mmol, 5.0 equiv) in reaction vial was added to the solution of bis(trifluoromethanesulfonyl)amine (7.0 mg, 0.025 mmol, 0.1 equiv) in anhydrous acetonitrile (0.85 mL, 0.3 M) and then thietanol dioxide (0.20 mmol, 1 equiv) was added and the reaction vial was sealed, and the mixture stirred for 4.5 h at 50 °C then cooled to rt. Sat. aq. NaHCO_3_ (15 mL) was added followed by CH_2_Cl_2_ (15 mL). The phases were separated and the aqueous layer was extracted with CH_2_Cl_2_ (2 × 15 mL). The organic layers were combined, dried over Na_2_SO_4_, filtered and concentrated in vacuo. Purification by flash column chromatography afforded the thietane dioxide ether.

#### 3-(4-Methoxyphenyl)-3-(3-phenylpropoxy)thietane 1,1-dioxide (**6aa**)

Performed using general procedure D with thietanol dioxide **2a** (45.7 mg, 0.20 mmol, 1 equiv) and 3-phenyl-1-propanol (0.17 mL, 1.25 mmol, 5.0 equiv). Purification by flash column chromatography (10% EtOAc/pentane) afforded thietane dioxide ether **6aa** (38.1 mg, 55%). R_f_ = 0.31 (15% EtOAc/pentane) as colorless oil; IR (film)/cm^–1^ 3026, 2945, 1608, 1513, 1322, 1251, 1212, 1165, 1132, 1068, 1032, 834, 743, 701, 661, 561; ^1^H NMR (400 MHz, CDCl_3_) δ 7.33–7.24 (m, 4H, 4 × Ar–H), 7.24–7.19 (m, 1H, Ar–H), 7.17 (dd, *J* = 8.1, 1.4 Hz, 2H, 2 × Ar–H), 6.98–6.91 (m, 2H, 2 × Ar–H), 4.54–4.41 (m, 4H, CHH–S–CHH), 3.85 (s, 3H, OCH_3_), 3.07 (t, *J* = 6.1 Hz, 2H, OCH_2_), 2.70 (t, *J* = 7.5 Hz, 2H, CH_2_), 1.94–1.82 (m, 2H, CH_2_); ^13^C{^1^H} NMR (101 MHz, CDCl_3_) δ 159.8 (Ar–C_q_), 141.3 (Ar–C_q_), 130.5 (Ar–C_q_), 128.5 (2 × Ar–C), 128.4 (2 × Ar–C), 127.6 (2 × Ar–C), 125.9 (Ar–C), 114.2 (2 × Ar–C), 73.8 (2 × S–CH_2_), 68.4 (C_q_), 63.3 (OCH_3_), 55.4 (OCH_2_), 31.9 (CH_2_), 30.8 (CH_2_); HRMS (ESI) *m*/*z* Calculated for C_19_H_26_O_4_SN [M + NH_4_]^+^: 364.1583; Found: 364.1578.

#### 3-Ethoxy-3-(4-methoxyphenyl)thietane 1,1-dioxide (**6ab**)

Performed using general procedure D with thietanol dioxide **2a** (45.7 mg, 0.20 mmol, 1 equiv) and ethanol (0.073 mL, 1.25 mmol, 5.0 equiv). Purification by flash column chromatography (10% EtOAc/pentane) afforded thietane dioxide ether **6ab** (21.0 mg, 41%). R_f_ = 0.31 (15% EtOAc/pentane) as colorless oil; IR (film)/cm^–1^ 2974, 2932, 2838, 1610, 1515, 1312, 1251, 1213, 1181, 1135, 1031, 975, 833, 766, 662, 563, 486; ^1^H NMR (400 MHz, CDCl_3_) δ 7.29 (d, *J* = 8.4 Hz, 2H, 2 × Ar–H), 6.93 (d, *J* = 8.3 Hz, 2H, 2 × Ar–H), 4.47 (s, 4H, CHH–S–CHH), 3.83 (s, 3H, OCH_3_), 3.13 (q, *J* = 7.0 Hz, 2H, OCH_2_), 1.16 (t, *J* = 7.0 Hz, 3H, CH_3_); ^13^C{^1^H} NMR (101 MHz, CDCl_3_) δ 159.9 (Ar–C_q_), 130.8 (Ar–C_q_), 127.5, (2 × Ar–C), 114.3 (2 × Ar–C), 74.1 (2 × S–CH_2_), 68.4 (C_q_), 60.3 (OCH_3)_, 55.4 (OCH_2_), 15.1 (CH_3_).

#### 3-((4-Bromobenzyl)oxy)-3-(4-methoxyphenyl)thietane 1,1-dioxide (**6ac**)

Performed using general procedure D with thietanol dioxide **2a** (45.7 mg, 0.20 mmol, 1 equiv) and 4-bromobenzyl alcohol (187 mg, 1.25 mmol, 5.0 equiv). Purification by flash column chromatography (10% EtOAc/pentane) afforded thietane dioxide ether **6ac** (31.7 mg, 40%). R_f_ = 0.24 (15% EtOAc/pentane) as white solid; mp = 152–155 °C; IR (film)/cm^–1^3025, 2957, 2838, 1608, 1515,1489,1321, 1250, 1211, 1133, 1033, 833, 807, 766, 513, 425; ^1^H NMR (400 MHz, CDCl_3_) δ 7.45 (d, *J* = 8.4 Hz, 2H, 2 × Ar–CH), 7.32 (d, *J* = 8.8 Hz, 2H, 2 × Ar–CH), 7.14 (d, *J* = 8.5 Hz, 2H, 2 × Ar–CH), 6.96 (d, *J* = 8.8 Hz, 2H, 2 × Ar–CH), 4.54 (s, 4H, CHH–SO_2_–CHH), 4.11 (s, 2H, OCH_2_), 3.85 (s, 3H, OCH_3_); ^13^C{^1^H} NMR (101 MHz, CDCl_3_) δ 160.1 (Ar–C_q_), 135.8 (Ar–C_q_), 131.6 (2 × Ar–C), 129.9 (Ar–C_q_), 129.0 (2 × Ar–C), 121.8 (Ar–C_q_), 114.5 (2 × Ar–C), 73.9 (2 × S–CH_2_), 69.2 (C_q_), 66.1 (OCH_2_), 55.4 (OCH_3_); HRMS (ESI) *m*/*z* Calculated for C_17_H_16_O_4_SBr [M – H]^−^: 394.9958; Found: 394.9960.

### Further Derivatization Reactions

#### 3-(4-Methoxyphenyl)-3-(4-(pyridin-2-yloxy)phenyl)thietane 1,1-dioxide (**7**)

3-(4-Hydroxyphenyl)-3-(4-methoxyphenyl)thietane 1,1-dioxide **3ba** (30.4 mg, 0.10 mmol, 1.0 equiv) and 2-iodopyridine (12.8 μL, 0.12 mmol, 1.2 equiv) were added to the solution of copper iodide (1.0 mg 0.005 mmol, 0.05 equiv), picolinic acid (1.2 mg, 0.01 mmol, 0.1 equiv), and potassium phosphate (42.4 mg, 0.2 mmol, 2.0 equiv) in 0.2 mL DMSO. The reaction was heated to 90 °C, leaving it stir for 24 h, before quenching the reaction with 10 mL water. The aqueous mixture was then extracted with EtOAc (3 × 10 mL). The combined organic layers were dried with Na_2_SO_4_, filtered and solvent removed under reduced pressure. Purification by flash chromatography (20% EtOAc/pentane) afforded 3-(4-methoxyphenyl)-3-(4-(pyridin-2-yloxy)phenyl)thietane 1,1-dioxide **7** (29.5 mg, 77%) as white solid. R_f_ = 0.22 (30% EtOAc/pentane); mp = 163–167 °C; IR (film)/cm^–1^ 3016, 2954, 1605, 1504, 1459, 1428, 1316, 1273, 1210, 1183, 1129, 1027, 885, 836, 783, 546, 412; ^1^H NMR (400 MHz, CDCl_3_) δ 8.21 (d, *J* = 4.8 Hz, 1H, Ar_pyr_–H), 7.73 (td, *J* = 8.0, 2.0 Hz, 1H, Ar_pyr_–H), 7.30 (d, *J* = 8.8 Hz, 2H, 2 × Ar–H), 7.24 (d, *J* = 8.8 Hz, 2H, 2 × Ar–H), 7.14 (d, *J* = 8.7 Hz, 2H, 2 × Ar–H), 7.07–7.01 (m, 1H, Ar_pyr_–H), 6.96 (d, *J* = 8.0 Hz, 1H, Ar_pyr_–H), 6.92 (d, *J* = 8.8 Hz, 2H, 2 × Ar–H), 4.89 (s, 4H, CHH–S–CHH), 3.83 (s, 3H, OCH_3_); ^13^C{^1^H} NMR (101 MHz, CDCl_3_) δ 163.1 (Ar–C_q_), 158.7 (Ar–C_q_), 153.2 (Ar–C_q_), 147.6 (Ar_pyr_–C), 141.0 (Ar–C_q_), 139.6 (Ar_pyr_–C), 136.1 (Ar–C_q_), 127.9 (4 × Ar–C), 121.3 (2 × Ar–C), 118.8 (Ar_pyr_–C), 114.3 (2 × Ar–C), 111.9 (Ar_pyr_–C), 77.0 (2 × S–CH_2_), 55.3 (OCH_3_), 36.5 (C_q_); HRMS (ESI) *m*/*z* Calculated for C_21_H_20_O_4_SN [M + H]^+^: 382.1113; Found: 382.1105.

#### 4-(3-(4-Methoxyphenyl)-1,1-dioxidothietan-3-yl)phenyl trifluoromethanesulfonate (**8**)

3-(4-Hydroxyphenyl)-3-(4-methoxyphenyl)thietane 1,1-dioxide **3ba** (252 mg, 0.82 mmol, 1.0 equiv) and pyridine (0.133 mL, 1.64 mmol, 2 equiv) in CH_2_Cl_2_ (1.7 mL) was slowly added by the triflic anhydride (0.153 mL, 0.90 mmol, 1.1 equiv) at 0 °C. The reaction flask was warmed up to 25 °C using a water bath then left to stir for 3 h, before quenching the reaction with sat. NaHCO_3_. The aqueous mixture was extracted with Et_2_O (3 × 30 mL). The combined organic layers were dried with Na_2_SO_4_, filtered and solvent removed under reduced pressure. Purification by flash chromatography (10% EtOAc/pentane) afforded 4-(3-(4-methoxyphenyl)-1,1-dioxidothietan-3-yl)phenyl trifluoromethanesulfonate **8** (357 mg, 99%) as colorless liquid. R_f_ = 0.51 (20% EtOAc/pentane); IR (film)/cm^–1^ 3015, 2969, 1608, 1515, 1502, 1414, 1321, 1265, 1208, 1129, 1032, 846, 827, 781, 762, 602, 544, 416; ^1^H NMR (400 MHz, CDCl_3_) δ 7.37 (d, *J* = 9.0 Hz, 2H, 2 × Ar–H), 7.25 (d, *J* = 9.0 Hz, 2H, 2 × Ar–H), 7.15 (d, *J* = 8.8 Hz, 2H, 2 × Ar–H), 6.92 (d, *J* = 8.8 Hz, 2H, 2 × Ar–H), 4.89 (d, *J* = 13.7 Hz, 2H, C*H*H–S–C*H*H), 4.80 (d, *J* = 13.7 Hz, 2H, CH*H*–S–CH*H*), 3.81 (s, 3H, OCH_3_); ^13^C{^1^H} NMR (101 MHz, CDCl_3_) δ 158.9 (Ar–C_q_), 148.3 (Ar–C_q_), 145.6 (Ar–C_q_), 135.2 (Ar–C_q_), 128.5 (2 × Ar–C), 127.8 (2 × Ar–C), 121.7 (2 × Ar–C), 114.5 (2 × Ar–C), 76.7 (2 × S–CH_2_), 55.3 (OCH_3_), 36.7 (C_q_); ^19^F NMR (377 MHz, CDCl_3_) δ −72.8; HRMS (ESI) *m*/*z* Calculated for C_17_H_14_O_6_S_2_F_3_ [M – H]^−^: 435.0189; Found: 435.0186.

#### 3-([1,1′-Biphenyl]-4-yl)-3-(4-methoxyphenyl)thietane 1,1-dioxide (**9**)

4-(3-(4-Methoxyphenyl)-1,1-dioxidothietan-3-yl)phenyl trifluoromethanesulfonate **8** (43.6 mg, 0.10 mmol, 1.0 equiv) and phenylboronic acid pinacol ester (30.6 mg, 0.15 mmol, 1.5 equiv) was added to a mixture of palladium(II) acetate (1.1 mg, 0.005 mmol, 0.05 equiv), SPhos (4.1 mg, 0.02 mmol, 0.1 equiv), and K_3_PO_4_ (84.9 mg, 0.20 mmol, 2.0 equiv) in dioxane/H_2_O (1.0 mL, 4:1). The reaction was heated to 65 °C and stirred for 24 h. The mixture was then filtered through Celite using Et_2_O (30 mL) and the solvent was removed under reduced pressure. Purification by flash chromatography (1:1:3 CH_2_Cl_2_/Et_2_O/pentane) afforded 3-([1,1′-biphenyl]-4-yl)-3-(4-methoxyphenyl)thietane 1,1-dioxide **9** (28.9 mg, 79%) as colorless oil; R_f_ = 0.55 (3:3:4 CH_2_Cl_2_/Et_2_O/pentane); IR (film)/cm^–1^ 3029, 2960, 2837, 1608, 1511, 1487, 1396, 1321, 1252, 1223, 1183, 1135, 1031, 829, 736, 766, 699, 578, 498; ^1^H NMR (400 MHz, CDCl_3_) δ 7.57 (t, *J* = 7.5 Hz, 4H, 4 × Ar–H), 7.44 (t, *J* = 7.6 Hz, 2H, 2 × Ar–H), 7.39–7.31 (m, 3H, 3 × Ar–H), 7.29–7.19 (m, 2H, 2 × Ar–H), 6.90 (d, *J* = 6.8 Hz, 2H, 2 × Ar–H), 4.89 (s, 4H, 2 × C*H*H–S–C*H*H), 3.81 (s, 3H, OCH_3_); ^13^C{^1^H} NMR (101 MHz, CDCl_3_) δ 158.7 (Ar–C_q_), 143.8 (Ar–C_q_), 140.2 (Ar–C_q_), 140.0 (Ar–C_q_), 136.3 (Ar–C_q_), 128.9 (2 × Ar–C), 127.6 (3 × Ar–C), 127.0 (2 × Ar–C), 126.9 (2 × Ar–C), 114.3 (2 × Ar–C), 76.7 (2 × S–CH_2_), 55.3 (OCH_3_), 36.8 (C_q_); HRMS (ESI) *m*/*z* Calculated for C_22_H_21_O_3_S [M + H]^+^: 365.1211; Found: 365.1225.

#### 3-(4-Methoxyphenyl)thietane-3-carboxylic acid 1,1-dioxide (**10**)

3-(4-Methoxyphenyl)-3-(5-methylfuran-2-yl)thietane 1,1-dioxide **3ao** (180 mg, 0.60 mmol, 1.0 equiv) was added to the solution of sodium periodate (898 mg, 4.20 mmol, 7.0 equiv) in mixture solvent of 15 mL (1:1:2 heptane/EtOAc/water). Ruthenium chloride (6.2 mg, 0.03 mmol, 0.05 equiv) was added to the reaction tube at 0 °C. Then, the reaction tube was warmed up to 25 °C using a water bath and then left it stir for 16 h, before quenching the reaction with water. The aqueous mixture was added by 30 mL sat. NaS_2_O_3_, then extracted with EtOAc (3 × 10 mL). The combined organic layer was then extracted with 10 mL NaOH three times. The combined aqueous layers were acidified with 1 M HCl until the value of pH was lower than 7. The aqueous solution was then extracted with EtOAc (3 × 20 mL). The combined organic layers were dried with Na_2_SO_4_, filtered and solvent removed under reduced pressure to afford 3-(4-methoxyphenyl)thietane-3-carboxylic acid 1,1-dioxide **10** (86.1 mg, 56%) as a white solid. R_f_ = 0.18 (50% EtOAc/pentane); mp = 152–156 °C; IR (film)/cm^–1^ 3193 (OH), 2960, 2915, 1735 (C=O), 1608, 1513, 1319, 1254, 1211, 1154, 1131, 1029, 833, 784, 734, 496, 442; ^1^H NMR (400 MHz, CDCl_3_) δ 7.23 (d, *J* = 8.9 Hz, 2H, 2 × Ar–H), 6.95 (d, *J* = 8.9 Hz, 2H, 2 × Ar–H), 4.97 (d, *J* = 14.6 Hz, 2H, C*H*H–S–C*H*H), 4.55 (d, *J* = 14.6 Hz, 2H, CH*H*–S–CH*H*), 3.84 (s, 3H, OCH_3_); ^13^C{^1^H} NMR (101 MHz, CDCl_3_) δ 159.9 (C=O), 129.1 (Ar–C_q_), 127.9 (Ar–C_q_), 114.7 (2 × Ar–C), 114.3 (2 × Ar–C), 72.9 (2 × S–CH_2_), 55.5 (OCH_3_), 39.6 (C_q_); HRMS (ESI) *m*/*z* Calculated for C_11_H_12_O_5_S [M – H]^−^: 255.0333; Found: 255.0331.

#### Ethyl (3-(4-methoxyphenyl)-1,1-dioxidothietane-3-carbonyl)valinate (**11**)

3-(4-Methoxyphenyl)thietane-3-carboxylic acid 1,1-dioxide **10** (25.6 mg, 0.10 mmol, 1.0 equiv) and dl-valine ethyl ester hydrochloride (21.8 mg, 0.12 mmol, 1.2 equiv) were added to the solution of DIPEA (55.7 μL, 0.32 mmol, 3.2 equiv) and HATU (45.6 mg, 0.12 mmol, 1.2 equiv) in CH_2_Cl_2_ (0.1 mL) at 0 °C, leave it stir for 1 h. Then, the reaction flask was warmed up to 25 °C using a water bath and then left it stir for 23 h, before quenching the reaction with water. The aqueous mixture was extracted with CH_2_Cl_2_ (3 × 10 mL). The combined organic layers were dried with Na_2_SO_4_, filtered and solvent removed under reduced pressure. Purification by flash chromatography (30–40% EtOAc/pentane) afforded ethyl (3-(4-methoxyphenyl)-1,1-dioxidothietane-3-carbonyl)valinate **11** (25.9 mg, 68%) as colorless oil. R_f_ = 0.31 (50% EtOAc/pentane); IR (film)/cm^–1^ 3289 (NH), 2960, 1735 (C=O), 1640, 1608, 1541, 1508, 1465, 1444, 1396, 1318, 1252, 1219, 1191, 1131, 1031, 833, 800, 691, 669, 572, 449; ^1^H NMR (400 MHz, CDCl_3_) δ 7.28 (d, *J* = 8.8 Hz, 2H, 2 × Ar–H), 6.99 (d, *J* = 8.8 Hz, 2H, 2 × Ar–H), 5.76 (d, *J* = 8.6 Hz, 1H, S–CH*H*), 5.08 (d, *J* = 14.2 Hz, 1H, S–CH*H*), 4.92 (d, *J* = 14.2 Hz, 1H, S–CH*H*), 4.53 (dd, *J* = 13.9, 2.8 Hz, 1H, S–CH*H*), 4.47–4.40 (m, 2H, N–CH + NH), 4.20–4.06 (m, 2H, OCH_2_), 3.84 (s, 3H, OCH_3_), 2.08 (dqd, *J* = 13.8, 6.9, 4.8 Hz, 1H, CH), 1.24 (t, *J* = 7.1 Hz, 3H), 0.78 (d, *J* = 6.9 Hz, 3H, CH_3_), 0.66 (d, *J* = 6.9 Hz, 3H, CH_3_); ^13^C{^1^H} NMR (101 MHz, CDCl_3_) δ 171.3 (C=O), 171.2 (C=O), 159.8 (Ar–C_q_), 129.3 (Ar–C_q_), 128.0 (2 × Ar–C), 115.1 (2 × Ar–C), 73.4 (S–CH_2_), 73.0 (S–CH_2_), 61.6 (OCH_2_), 57.8 (N–CH), 55.5 (OCH_3_), 39.4 (C_q_), 31.1 (CH), 18.9 (CH_3_), 17.3 (CH_3_), 14.2 (CH_3_); HRMS (ESI) *m*/*z* Calculated for C_18_H_26_O_6_SN [M + H]^+^: 384.1481; Found: 384.1498.

## Data Availability

The data underlying this study are available in the published article, in its Supporting Information and openly available in the Imperial College London Research Data Repository at 10.14469/hpc/14599.

## Supporting Information Available

X-ray Crystallography data; Copies of ^1^H and ^13^C NMR spectra (PDF)

## Related Paper

A version of this manuscript was deposited on the preprint repository ChemRxiv.^19^

## References

[ref1] Shearer J.; Castro J. L.; Lawson A. D. J.; MacCoss M.; Taylor R. D. Rings in Clinical Trials and Drugs: Present and Future. J. Med. Chem. 2022, 65, 8699–8712. 10.1021/acs.jmedchem.2c00473. 35730680 PMC9289879

[ref2] aRojas J. J.; Bull J. A. Oxetane in Drug Discovery Campaigns. J. Med. Chem. 2023, 66, 12697–12709. 10.1021/acs.jmedchem.3c01101. 37676858 PMC10544023

[ref3] Francisco K. R.; Ballatore C. Thietanes and Derivatives thereof in Medicinal Chemistry. Curr. Top. Med. Chem. 2022, 22, 1219–1234. 10.2174/1568026622666220511154228. 35546768

[ref4] Boezio A.; Taylor A. M.; Gunaydin H.; Zhang H.; Raynor K. D.; Shortsleeves K. C.; Dipietro L. V.; Pierce L. C. T.; Pabon N.; Mclean T. H.; Giordanetto F.; Pechersky Y.; Wang Q.; Laruvee A.; Chen F.; Maertens G.; Outin J.; Bertrand-Laperle M.; Pal M.; Chitale S.; Deninno M. P. Pi3k-Alpha Inhibitors and Methods of Use Thereof. WO 2023039532 A1, March 16, 2023.

[ref5] Cohen F.; Aggen J. B.; Andrews L. D.; Assar Z.; Boggs J.; Choi T.; Dozzo P.; Easterday A. N.; Haglund C. M.; Hildebrandt D. J.; Holt M. C.; Joly K.; Jubb A.; Kamal Z.; Kane T. R.; Konradi A. W.; Krause K. M.; Linsell M. S.; Machajewski T. D.; Miroshnikova O.; Moser H. E.; Nieto V.; Phan T.; Plato C.; Serio A. W.; Seroogy J.; Shakhmin A.; Stein A. J.; Sun A. D.; Sviridov S.; Wang Z.; Wlasichuk K.; Yang W.; Zhou X.; Zhu H.; Cirz R. T. Optimization of LPXC Inhibitors for Antibacterial Activity and Cardiovascular Safety. ChemMedChem. 2019, 14, 1560–1572. 10.1002/cmdc.201900287. 31283109

[ref6] Renold P.; Zambach W.; Maienfisch P.; Michel N. Insecticidal Compound. WO 2009080250 A2, July 2, 2009.

[ref7] aLassalas P.; Oukoloff K.; Makani V.; James M.; Tran V.; Yao Y.; Huang L.; Vijayendran K.; Monti L.; Trojanowski J. Q.; Lee V. M.; Kozlowski M. C.; Smith A. B.; Brunden K. R.; Ballatore C. Evaluation of Oxetan-3-ol, Thietan-3-ol, and Derivatives Thereof as Bioisosteres of the Carboxylic Acid Functional Group. ACS Med. Chem. Lett. 2017, 8, 864–868. 10.1021/acsmedchemlett.7b00212. 28835803 PMC5554911

[ref8] Zhao F.; Lin Z.; Wang F.; Zhao W.; Dong X. Four-membered Heterocycles-containing 4-Anilino-quinazoline Derivatives as Epidermal Growth Factor Receptor (EGFR) Kinase Inhibitors. Bioorg. Med. Chem. Lett. 2013, 23, 5385–5388. 10.1016/j.bmcl.2013.07.049. 23973168

[ref9] aCroft R. A.; Mousseau J. J.; Choi C.; Bull J. A. Structurally Divergent Lithium Catalyzed Friedel-Crafts Reactions on Oxetan-3-ols: Synthesis of 3,3-Diaryloxetanes and 2,3-Dihydrobenzofurans. Chem.—Eur. J. 2016, 22, 16271–16276. 10.1002/chem.201604031. 27723135 PMC5095816

[ref10] aCroft R. A.; Mousseau J. J.; Choi C.; Bull J. A. Lithium-Catalyzed Thiol Alkylation with Tertiary and Secondary Alcohols: Synthesis of 3-Sulfanyl-Oxetane as Bioisosteres. Chem.—Eur. J. 2018, 24, 818–821. 10.1002/chem.201705576. 29181870 PMC5814735

[ref11] Saejong P.; Rojas J. J.; Denis C.; White A. J. P.; Voisin-Chiret A. S.; Choi C.; Bull J. A. Synthesis of Oxetane and Azetidine Ethers as Ester Isosteres by Brønsted Acid Catalysed Alkylation of Alcohols with 3-Aryl-oxetanols and 3-Aryl-azetidinols. Org. Biomol. Chem. 2023, 21, 5553–5559. 10.1039/D3OB00731F. 37345459

[ref12] Tian D.; Chen G.; Wang X.; Zhang H.-J. Modular Access to Functionalized Oxetanes as Benzoyl Bioisosteres. J. Am. Chem. Soc. 2024, 146, 18011–18018. 10.1021/jacs.4c04504. 38905313

[ref13] Xu J. Recent synthesis of thietanes. Beilstein J. Org. Chem. 2020, 16, 1357–1410. Also see ref (7) 10.3762/bjoc.16.116. 32647542 PMC7323639

[ref14] aBaumann A. N.; Reiners F.; Juli T.; Didier D. Chemodivergent and Stereoselective Access to Fused Isoxazoline Azetidines and Thietane through [3 + 2]-Cycloaddition. Org. Lett. 2018, 20, 6736–6740. 10.1021/acs.orglett.8b02848. 30351958

[ref15] Rojas J. J.; Croft R. A.; Sterling A. J.; Briggs E. L.; Antermite D.; Schmitt D. C.; Blagojevic L.; Haycock P.; White A. J. P.; Duarte F.; Choi C.; Mousseau J. J.; Bull J. A. Amino-Oxetane as Amide Isosters by an Alternative Defluorosulfonylative Coupling of Sulfonyl Fluoride. Nat. Chem. 2022, 14, 160–169. 10.1038/s41557-021-00856-2. 35087220

[ref16] aDubois M. A. J.; Smith M. A.; White A. J. P.; Lee Wei Jie A.; Mousseau J. J.; Choi C.; Bull J. A. Short Synthesis of Oxetane and Azetidine 3-Aryl-3-carboxylic Acid Derivatives by Selective Furan Oxidative Cleavage. Org. Lett. 2020, 22, 5279–5283. 10.1021/acs.orglett.0c01214. 32338914

[ref17] Love B. E.; Jones E. G. The use of Salicylaldehyde Phenylhydrazone as an Indicator for the Titration of Organometallic Reagents. J. Org. Chem. 1999, 64, 3755–3756. 10.1021/jo982433e. 11674512

[ref18] Greb A.; Poh J.-S.; Greed S.; Battilocchio C.; Pasau P.; Blakemore D. C.; Ley S. V. A Versatile Route to Unstable Diazo Compounds *via* Oxadiazolines and Their Use in Aryl-Alkyl Cross-Coupling Reactions. Angew. Chem., Int. Ed. 2017, 56, 16602–16605. 10.1002/anie.201710445. PMC5814725 29088512

[ref19] Saejong P.; Zhong J.; Rojas J. J.; White A. J. P.; Choi C.; Bull J. A. Synthesis of 3,3-Disubstituted Thietane Dioxides [preprint]. ChemRxiv, July 22, 2024. 10.26434/chemrxiv-2024-6c9kc PMC11536365 39392182

