# Supplementary material for: Synthesis of 3,3-Disubstituted Thietane Dioxides
Source: J Org Chem. 2024 Oct 11;89(21):15718–32. doi: 10.1021/acs.joc.4c01843 (PMC11536365; doi:10.1021/acs.joc.4c01843)
Supplement: Supplementary file 1 — jo4c01843_si_001.pdf [file jo4c01843_si_001.pdf]

SUPPORTING INFORMATION

Synthesis of 3,3-Disubstituted Thietane Dioxides

Peerawat Saejong,[a] Jaining Zhong,[a] Juan J. Rojas,[a] Andrew J. P. White,[a] Chulho Choi,[b] and James A. Bull[a]*

[a] Department of Chemistry, Imperial College London, Molecular Sciences Research Hub, White City Campus, Wood Lane W12 0BZ, UK
[b] Pfizer Worldwide Research, Development and Medical, Eastern Point Rd., Groton, CT 06340, USA.

*E-mail: j.bull@imperial.ac.uk

Single crystal X-ray data for 3,3-disubstituted thietane dioxides **2a**, **3aa**, **3ac** and **5aa**. ……………….S2-S5
^{1}H and ^{13}C NMR spectra……………………………………………………………………………….S6-S70
Thietane-3-ols……………………………………………………………………………….S7-S15
Thietane-3-ol-1,1-dioxides…………………………………………………………………….S16-S26
Friedel–Crafts of thietane dioxides……………………………………………………………….S27-S54
C-S alkylation of thietane dioxides ……………………………………………………………….. S55-S61
C-O alkylation of thietane dioxide…………………………………………………………….....S62-S64
Further functionalisation of 3,3-disubstituted thietane dioxide…………………………………….S65-S70
References…………………………………………………………………………………………….S71

Preparation of single crystals

2a: Single crystals of **2a** were grown by dissolving the compound in a solvent mixture of CH_2Cl_2 and hexane (6:4) at 40 °C. The warm mixture (2.0 mL) was added to **2a** (3.1 mg) in a 3 mL vial, which was then capped and left at room temperature until crystals formed.
3aa: For the growth of single crystals of **3aa**, acetone was used to dissolve the compound in a 3 mL vial. The vial was then loosely capped and placed inside a 10 mL vial containing 1.0 mL of heptane. The larger vial was sealed and stored at –20 °C until crystal formation was observed.
3ac and **5aa:** Single crystals of **3ac** and **5aa** were grown slowly in $CDCl_3$ at room temperature.

Crystal Data, Data Collection and Refinement Parameters

Table S1. Crystal Data, Data Collection and Refinement Parameters for the structures of **2a**, **3aa**, **3ac** and **5aa**.

data	2a	3aa	3ac	5aa
**formula**	$C_{10}H_{12}O_4S$	$C_{17}H_{18}O_4S$	$C_{18}H_{20}O_4S$	$C_{17}H_{18}O_3S_2$
**formula weight**	228.26	318.37	332.40	334.43
**colour, habit**	colourless needles	colourless blocks	colourless tablets	colourless tablets
**temperature / K**	173	173	173	173
**crystal system**	monoclinic	orthorhombic	monoclinic	monoclinic
**space group**	$P2_1/c$ (no. 14)	$P2_12_12_1$ (no. 19)	$P2_1/c$ (no. 14)	$P2_1/c$ (no. 14)
***a* / Å**	5.6198(3)	9.1214(2)	7.0019(2)	13.0110(3)
***b* / Å**	6.9237(3)	11.1350(3)	11.1321(3)	5.89527(15)
***c* / Å**	25.4955(12)	15.0959(4)	20.8963(6)	21.4203(6)
**α / deg**	90	90	90	90
**β / deg**	96.029(5)	90	95.061(3)	91.614(3)
**γ / deg**	90	90	90	90
**V / Å^3**	986.54(8)	1533.24(6)	1622.43(8)	1642.35(8)
Z	4	4	4	4
**D_c / g cm^{-3}**	1.537	1.379	1.361	1.353
**radiation used**	Cu-Kα	Mo-Kα	Cu-Kα	Cu-Kα
**μ / mm^{-1}**	2.876	0.227	1.927	3.018
**no. of unique reflns**				
**measured (R_{int})**	1938 (0.0296)	3464 (0.0397)	3130 (0.0358)	3271 (0.0395)
**obs, $\|F_o\| > 4\sigma(\|F_o\|)$**	1618	3161	2360	2673
**completeness (%)** [a]	99.9	99.9	98.7	99.8
**no. of variables**	142	206	216	202
**R_1(obs), wR_2(all)** [b]	0.0363, 0.1035	0.0335, 0.0800	0.0423, 0.1181	0.0372, 0.1027
**CCDC code**	2368330	2368331	2368332	2368333

[a] Completeness to 0.84 Å resolution. [b] $R_1 = \Sigma||F_o| - |F_c||/\Sigma|F_o|$; $wR_2 = \{\Sigma[w(F_o^2 - F_c^2)^2] / \Sigma[w(F_o^2)^2]\}^{1/2}$; $w^{-1} = \sigma^2(F_o^2) + (aP)^2 + bP$.

Table S1 provides a summary of the crystallographic data for the structures of **2a**, **3aa**, **3ac** and **5aa**. Data were collected using Agilent Xcalibur PX Ultra A (**2a**, **3ac** and **5aa**) and Agilent Xcalibur 3 E (**3aa**) diffractometers, and the structures were solved and refined using the OLEX2,[1] SHELXTL[2] and SHELX-2013[3] program systems. The absolute structure of **3aa** was determined by use of the Flack parameter [x = 0.00(3)]. CCDC 2368330 to 2368333.

X-Ray crystallography

The O15–H hydrogen atom in the structure of **2a** was located from a Δ*F* map and refined freely subject to an O–H distance constraint of 0.90 Å. Whilst the structure of **2a** can be modelled using a half volume unit cell based on halving the *c* axis length, this results in a significantly disordered model in a chiral space group with an indeterminate Flack parameter [$x = 0.25(11)$]. The version presented here using the longer *c* axis, however, has no disorder in a centrosymmetric space group and is thus much preferred. The O22–H hydrogen atom in the structure of **3aa** was located from a Δ*F* map and refined freely subject to an O–H distance constraint of 0.90 Å. The absolute structure of **3aa** was determined by use of the Flack parameter [$x = 0.00(3)$]. The O22–H hydrogen atom in the structure of **3ac** was located from a Δ*F* map and refined freely subject to an O–H distance constraint of 0.90 Å.

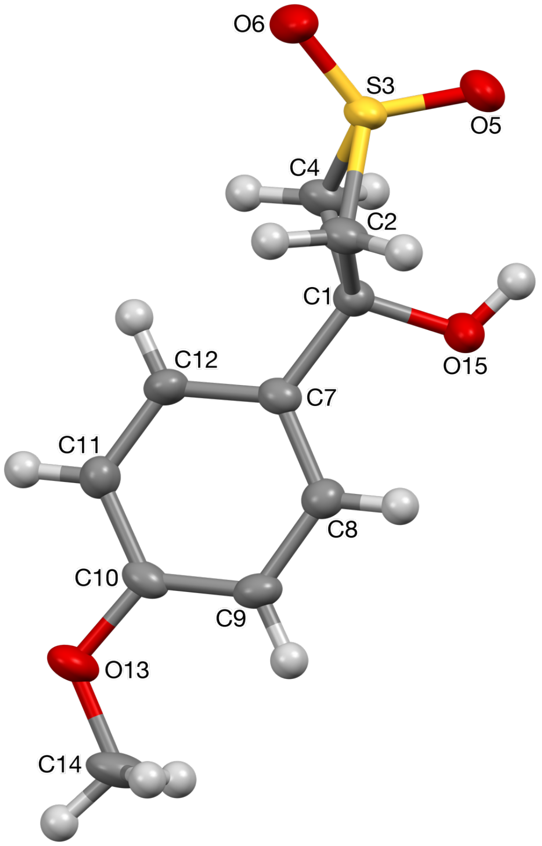

Fig. S1 The crystal structure of **2a** (65% probability ellipsoids).

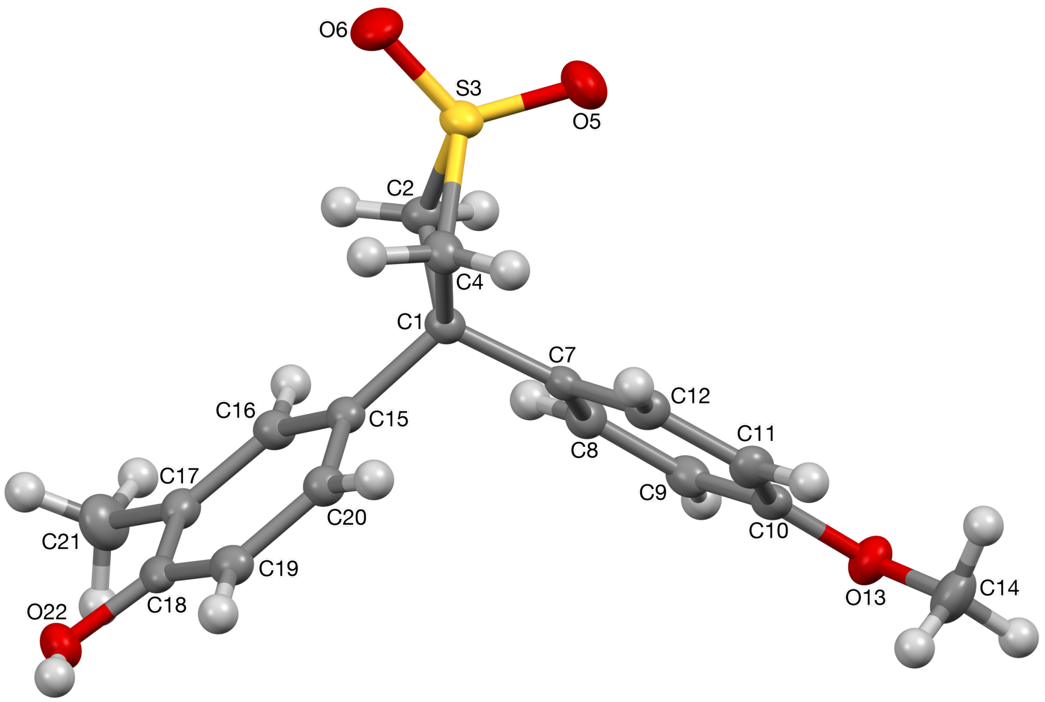

Fig. S2 The crystal structure of **3aa** (50% probability ellipsoids).

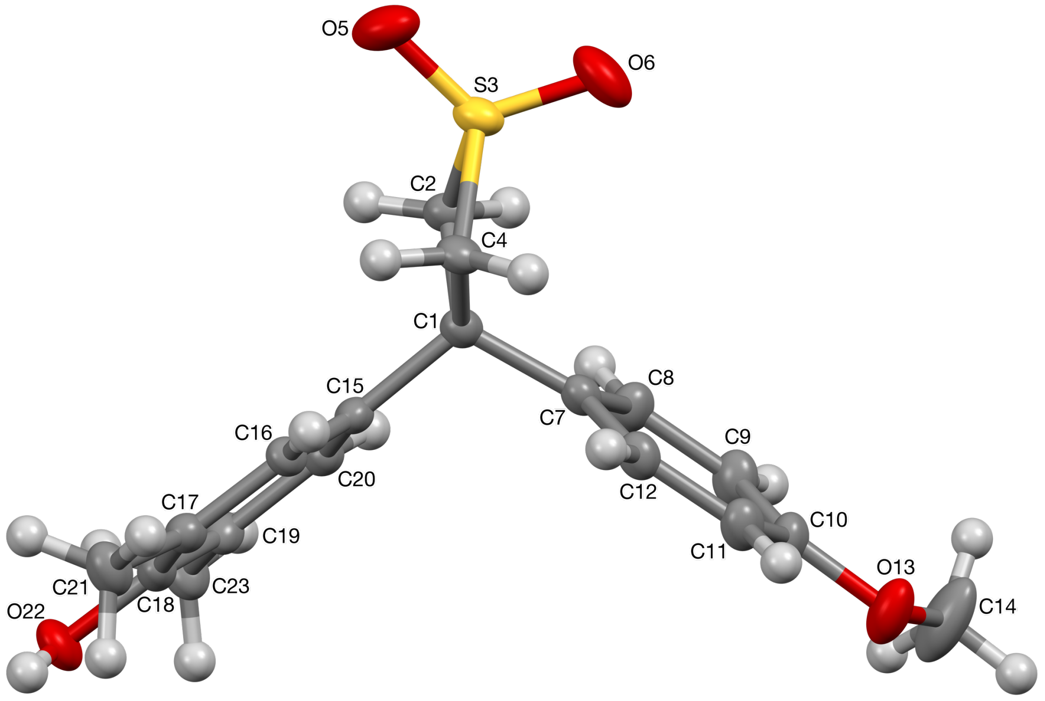

Fig. S3 The crystal structure of **3ac** (50% probability ellipsoids).

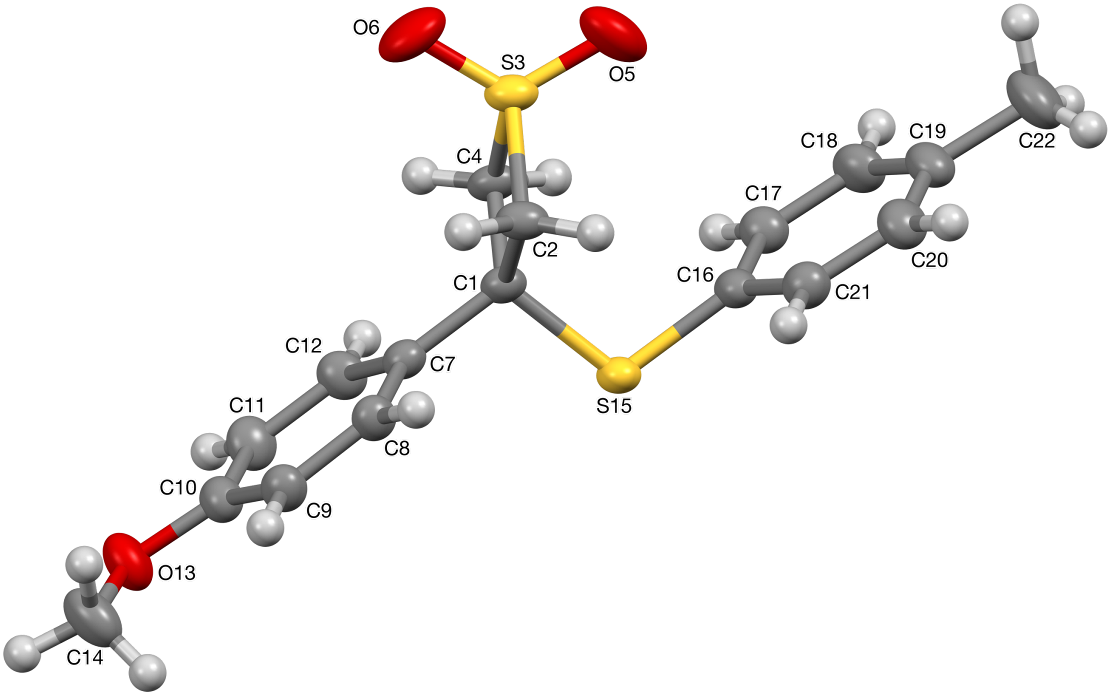

Fig. S4 The crystal structure of **5aa** (50% probability ellipsoids).

^{1}H and ^{13}C NMR Spectra of Selected Compounds

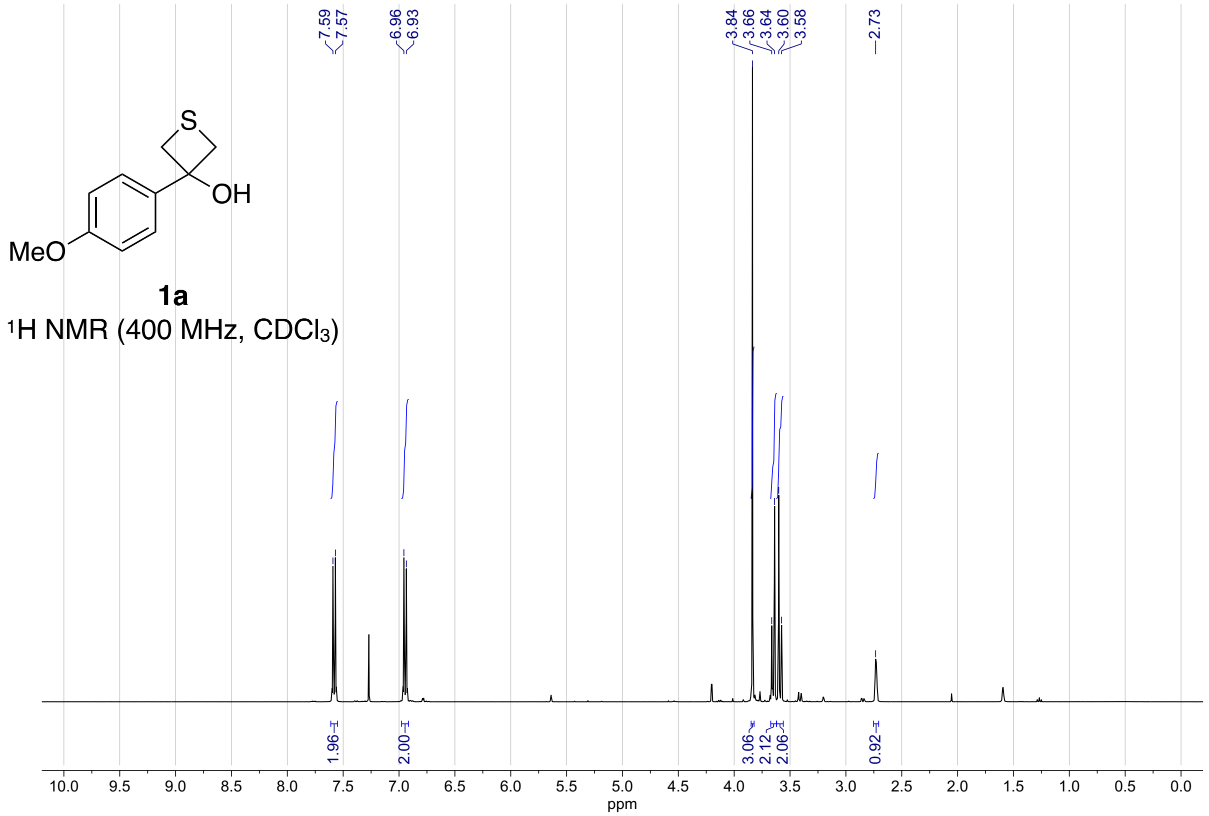
S
OH
MeO
1a
¹H NMR (400 MHz, CDCl₃)
7.59
7.57
6.96
6.93
3.84
3.66
3.64
3.60
3.58
2.73
1.96
2.00
3.06
2.12
2.06
0.92
ppm

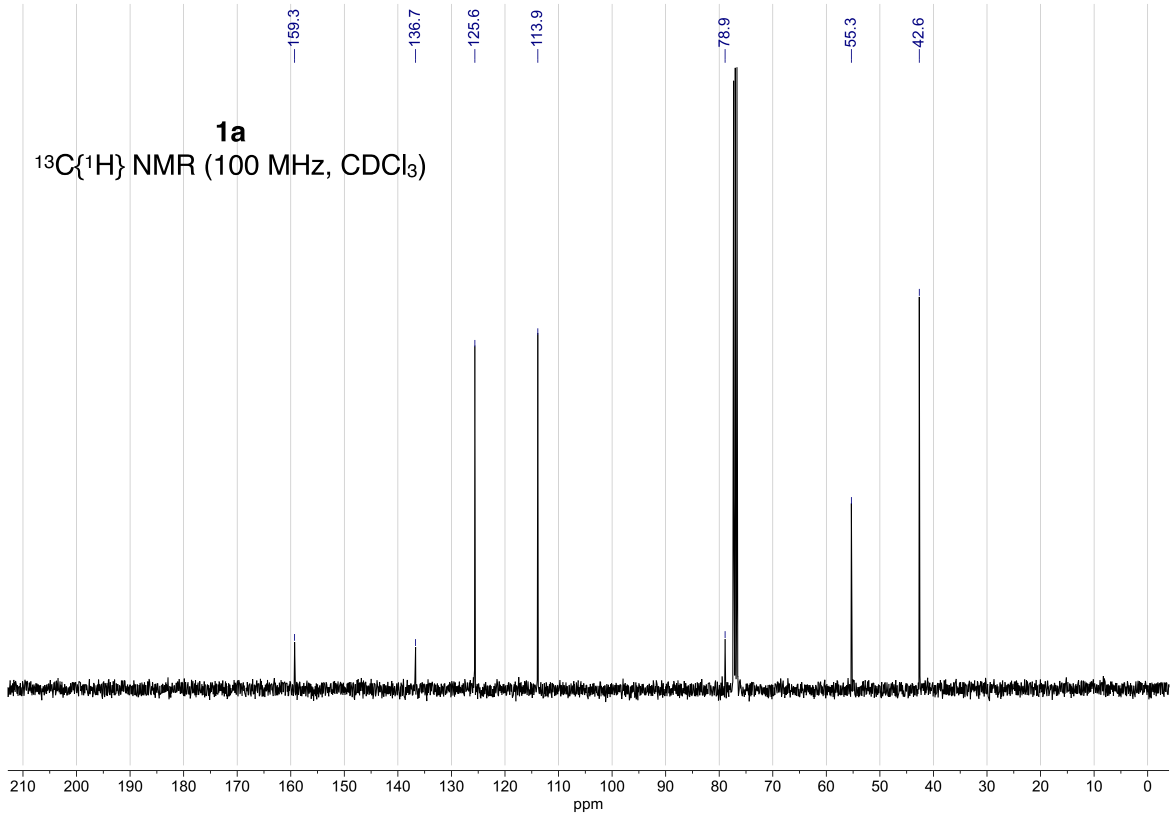
1a
¹³C{¹H} NMR (100 MHz, CDCl₃)
159.3
136.7
125.6
113.9
78.9
55.3
42.6
ppm

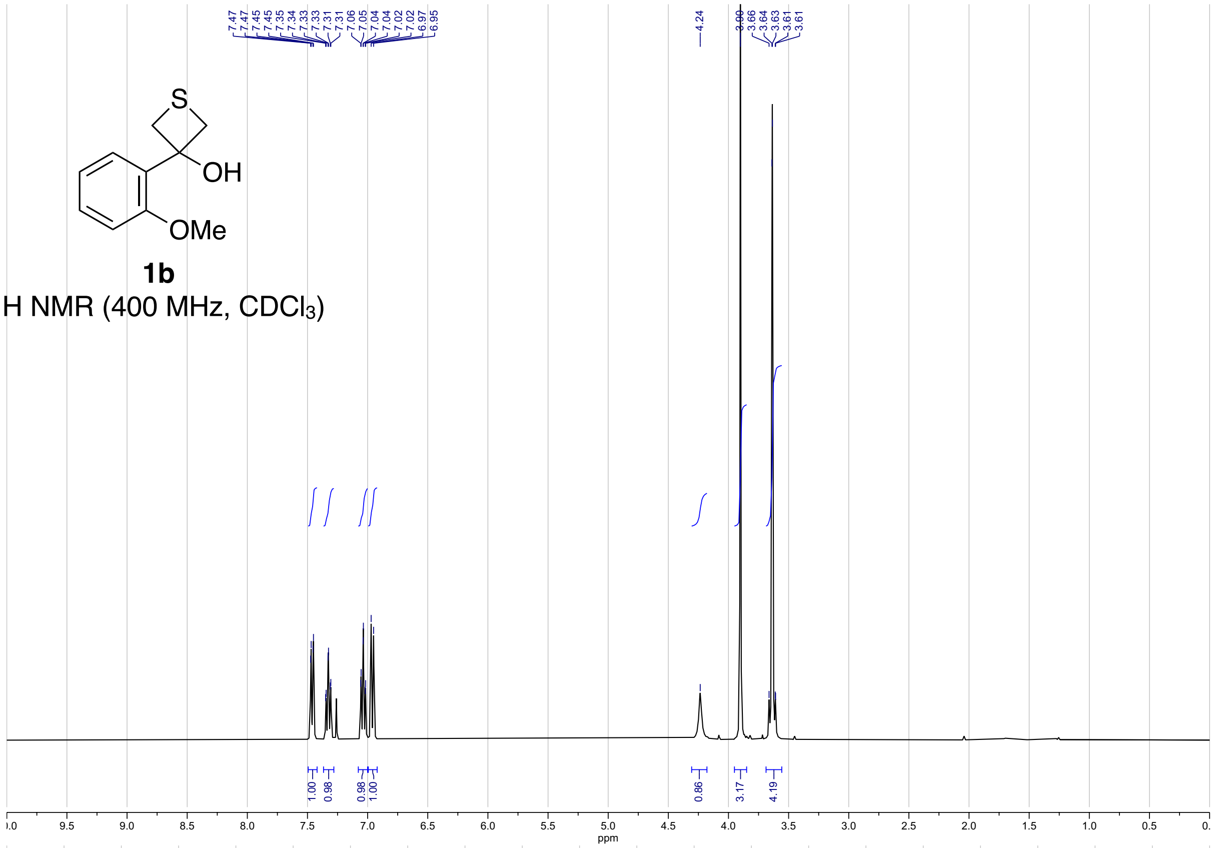
S
OH
OMe
1b
¹H NMR (400 MHz, CDCl₃)
7.47 7.47 7.45 7.45 7.35 7.34 7.33 7.33 7.31 7.31 7.06 7.05 7.04 7.04 7.02 7.02 6.97 6.95
4.24
3.90 3.66 3.64 3.63 3.61 3.61
1.00 0.98 0.98 1.00 0.86 3.17 4.19
ppm

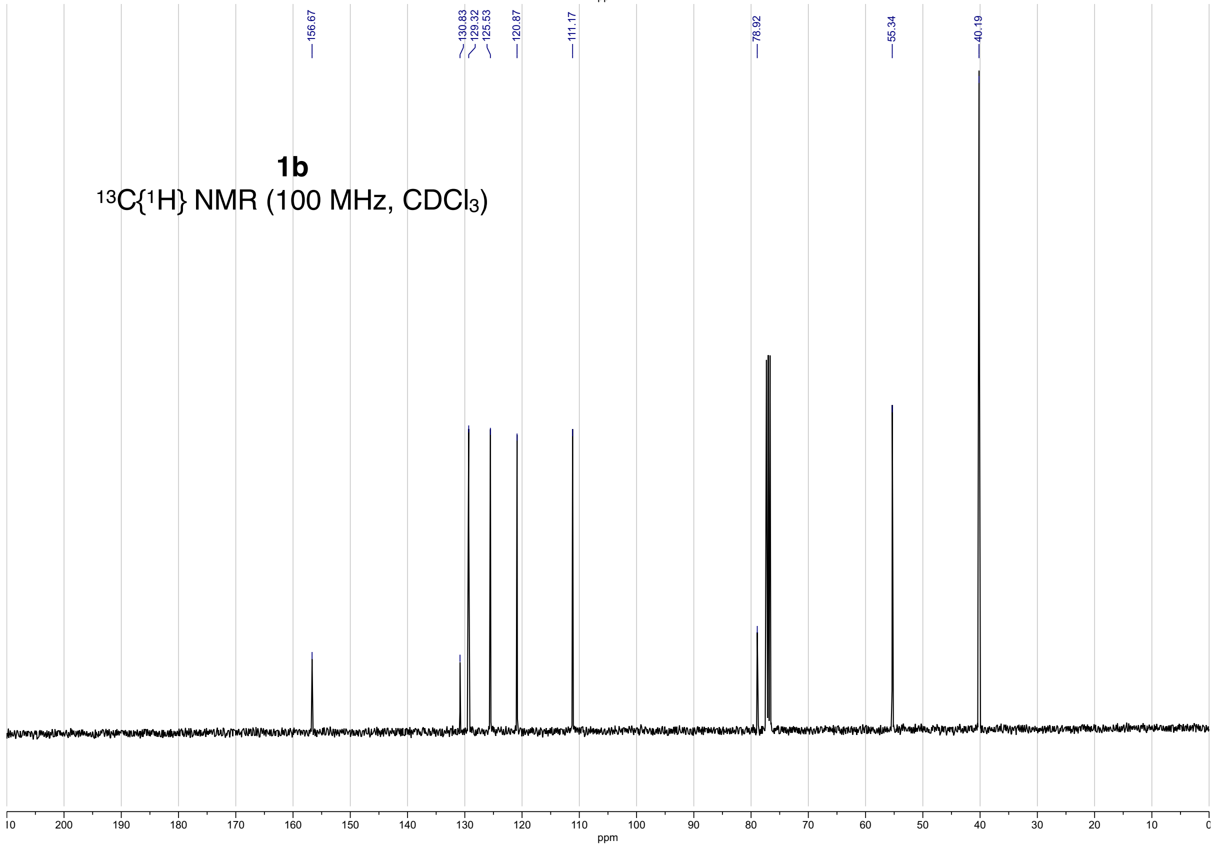
1b
¹³C{¹H} NMR (100 MHz, CDCl₃)
156.67
130.83 129.32 125.53 120.87
111.17
78.92
55.34
40.19
ppm

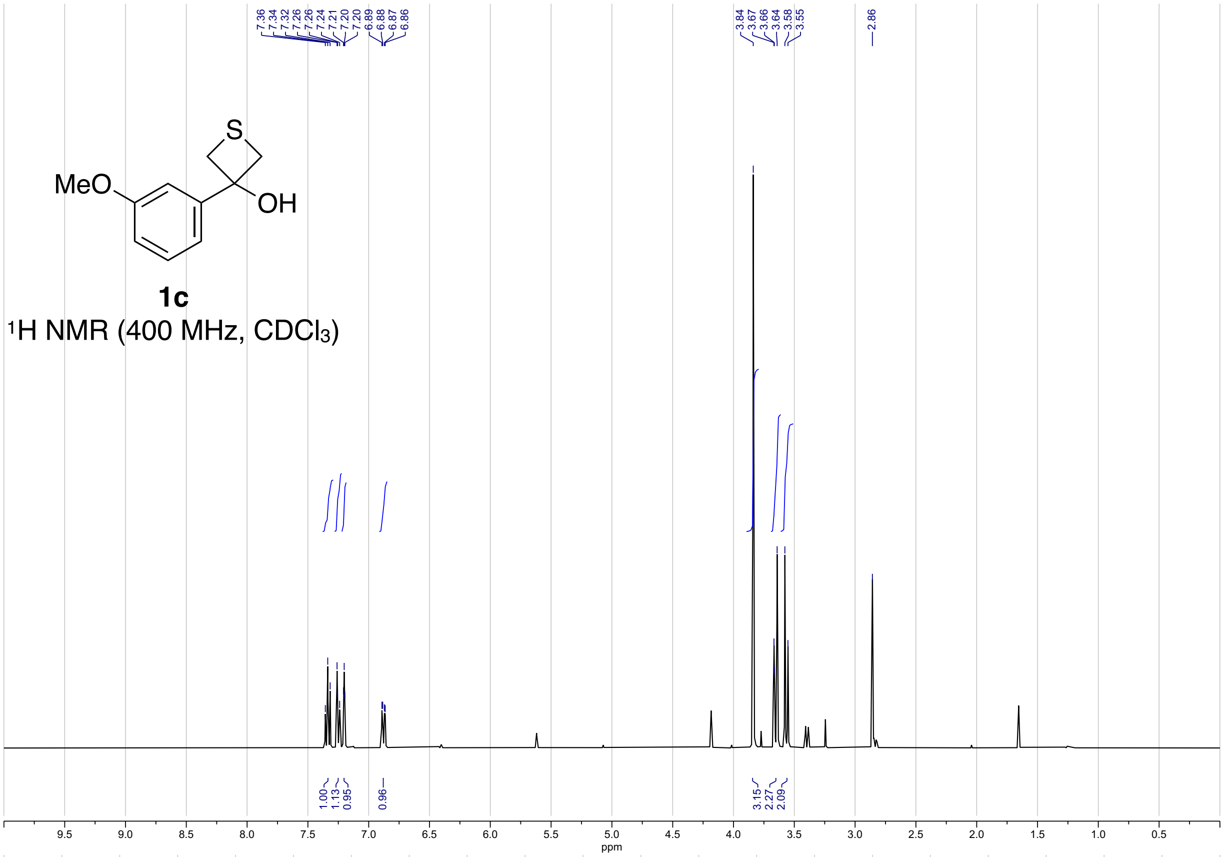
7.36 7.34 7.32 7.26 7.24 7.21 7.20 7.20 6.89 6.88 6.87 6.86
3.84 3.67 3.66 3.64 3.58 3.55
2.86
S
MeO
OH
1c
¹H NMR (400 MHz, CDCl₃)
1.00 1.13 0.95 0.96
3.15 2.27 2.09
9.5 9.0 8.5 8.0 7.5 7.0 6.5 6.0 5.5 5.0 4.5 4.0 3.5 3.0 2.5 2.0 1.5 1.0 0.5
ppm

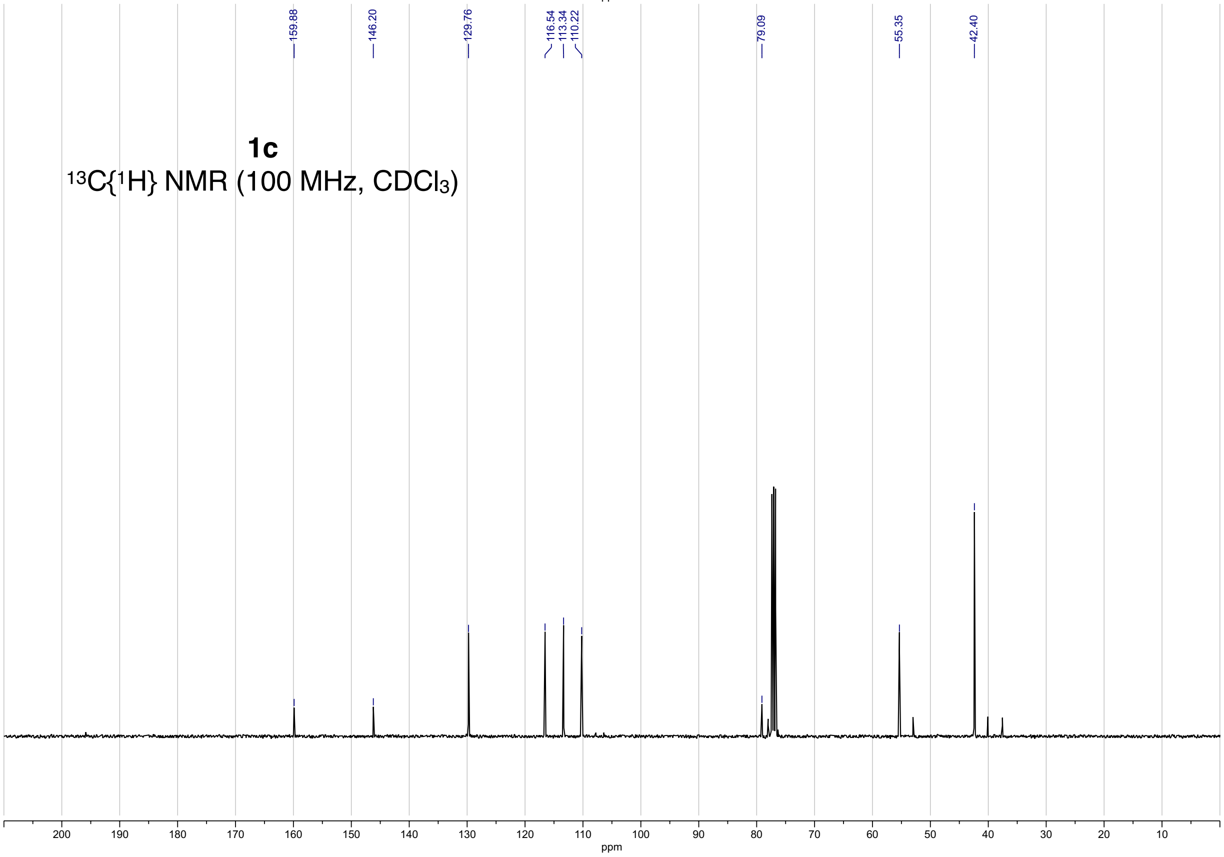
159.88
146.20
129.76
116.54 113.34 110.22
79.09
55.35
42.40
1c
¹³C{¹H} NMR (100 MHz, CDCl₃)
200 190 180 170 160 150 140 130 120 110 100 90 80 70 60 50 40 30 20 10
ppm

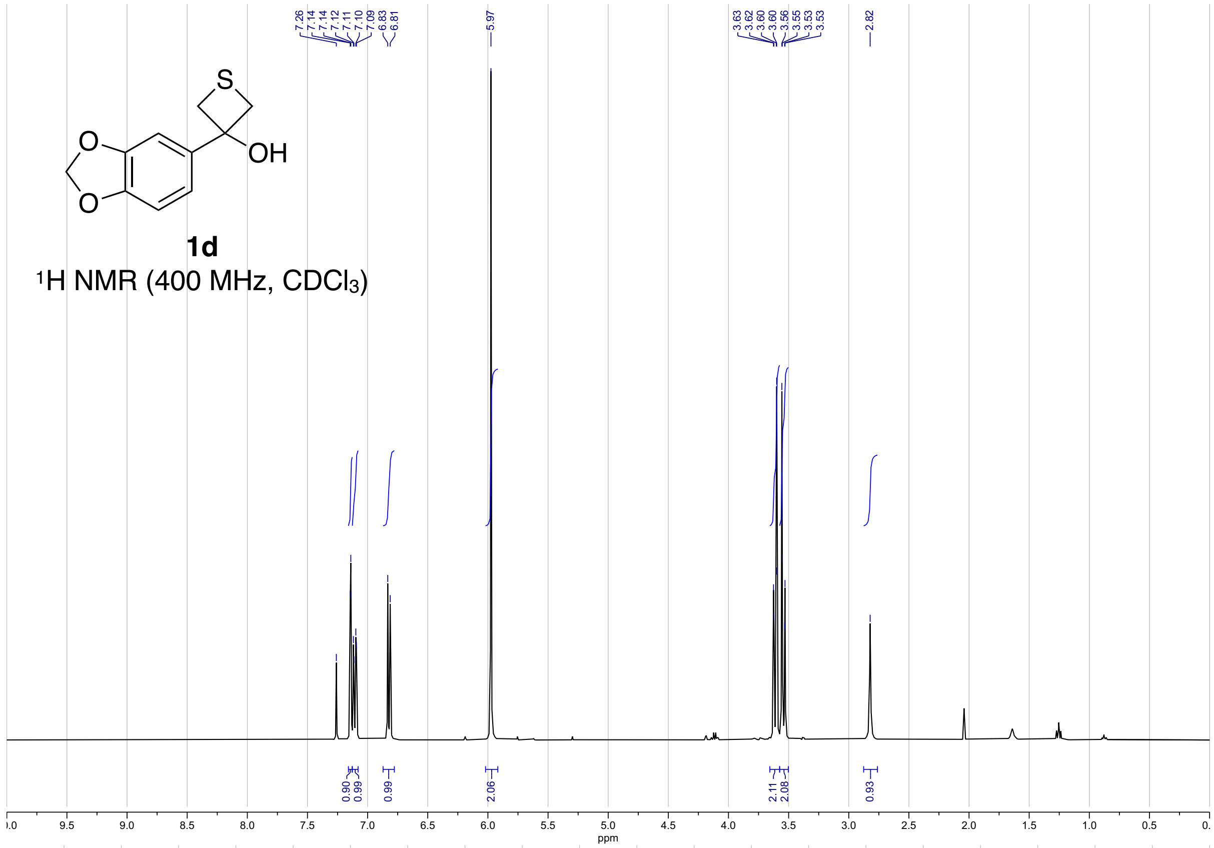
7.26
7.14
7.14
7.12
7.11
7.10
7.09
6.83
6.81
5.97
3.63
3.62
3.60
3.60
3.56
3.55
3.53
3.53
2.82
S
O
O
OH
1d
1H NMR (400 MHz, CDCl3)
0.90
0.99
0.99
2.06
2.11
2.08
0.93
9.5
9.0
8.5
8.0
7.5
7.0
6.5
6.0
5.5
5.0
4.5
4.0
3.5
3.0
2.5
2.0
1.5
1.0
0.5
ppm

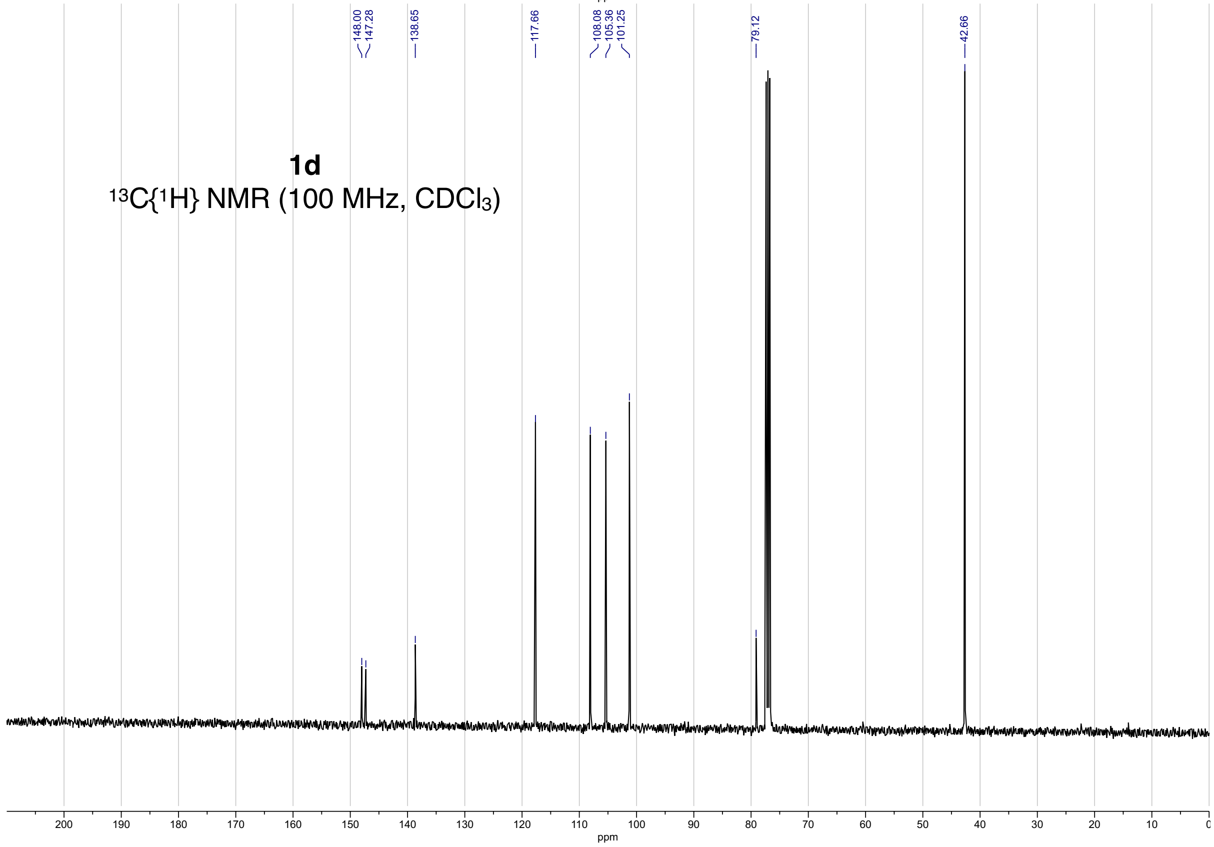
148.00
147.28
138.65
117.66
108.08
105.36
101.25
79.12
42.66
1d
13C{1H} NMR (100 MHz, CDCl3)
200
190
180
170
160
150
140
130
120
110
100
90
80
70
60
50
40
30
20
10
ppm

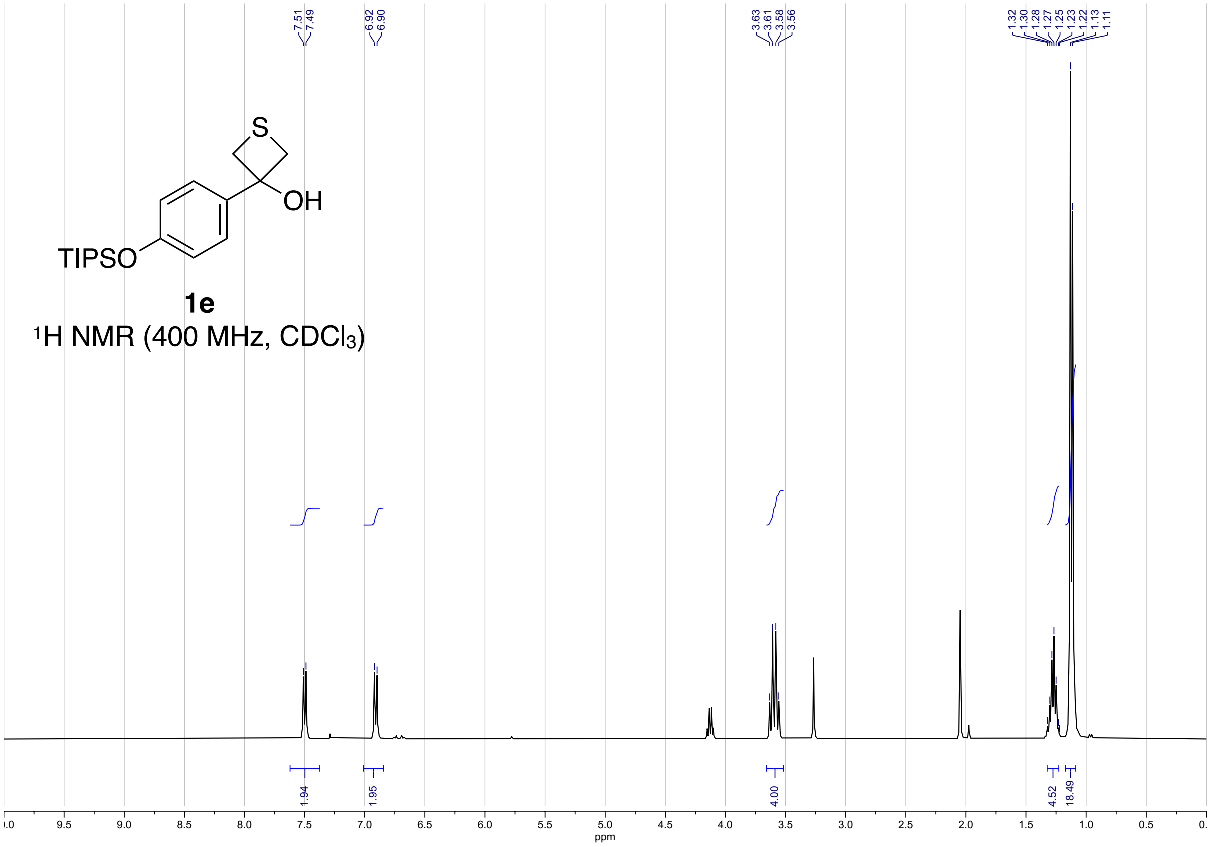
7.51
7.49
6.92
6.90
3.63
3.61
3.58
3.56
1.32
1.30
1.28
1.27
1.25
1.23
1.22
1.13
1.11
S
OH
TIPSO
1e
^{1}H NMR (400 MHz, $CDCl_3$)
1.94
1.95
4.00
4.52
18.49
ppm

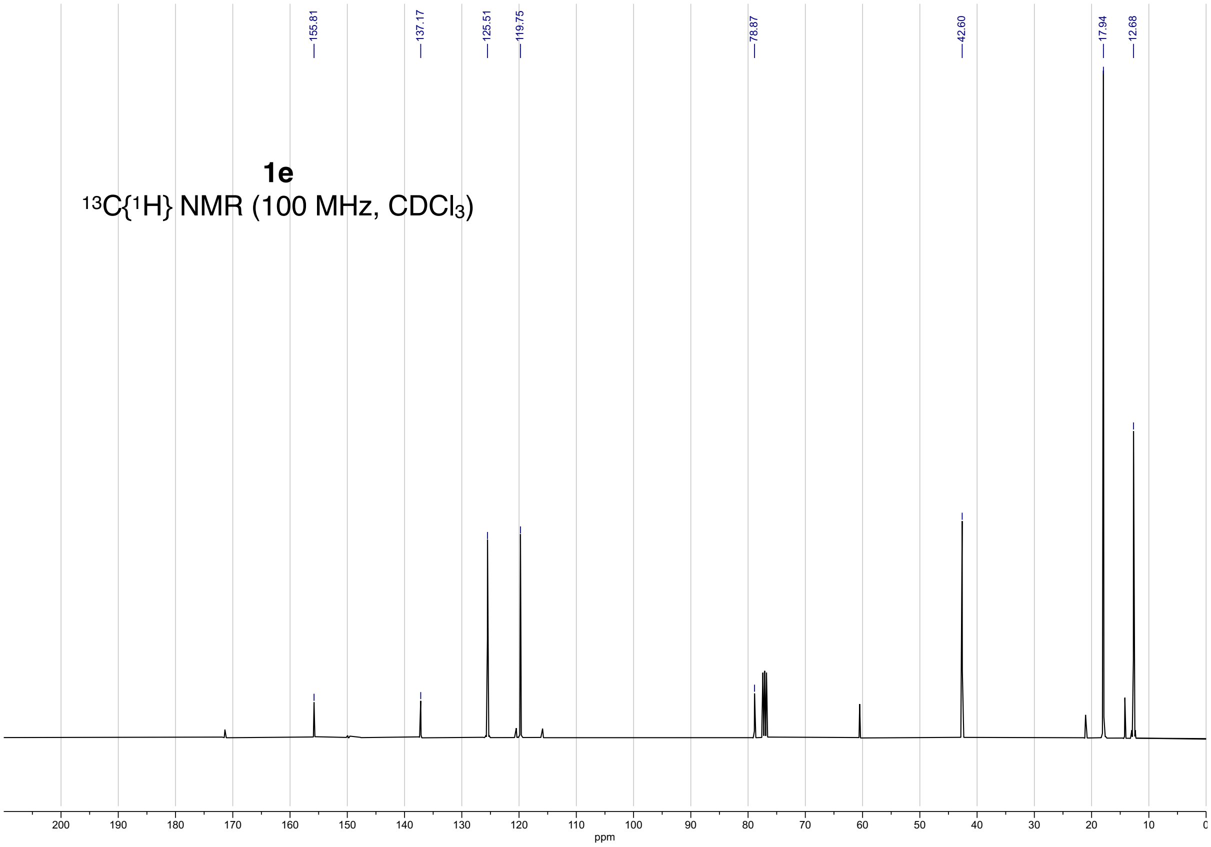
155.81
137.17
125.51
119.75
78.87
42.60
17.94
12.68
1e
$^{13}C\{^1H\}$ NMR (100 MHz, $CDCl_3$)
ppm

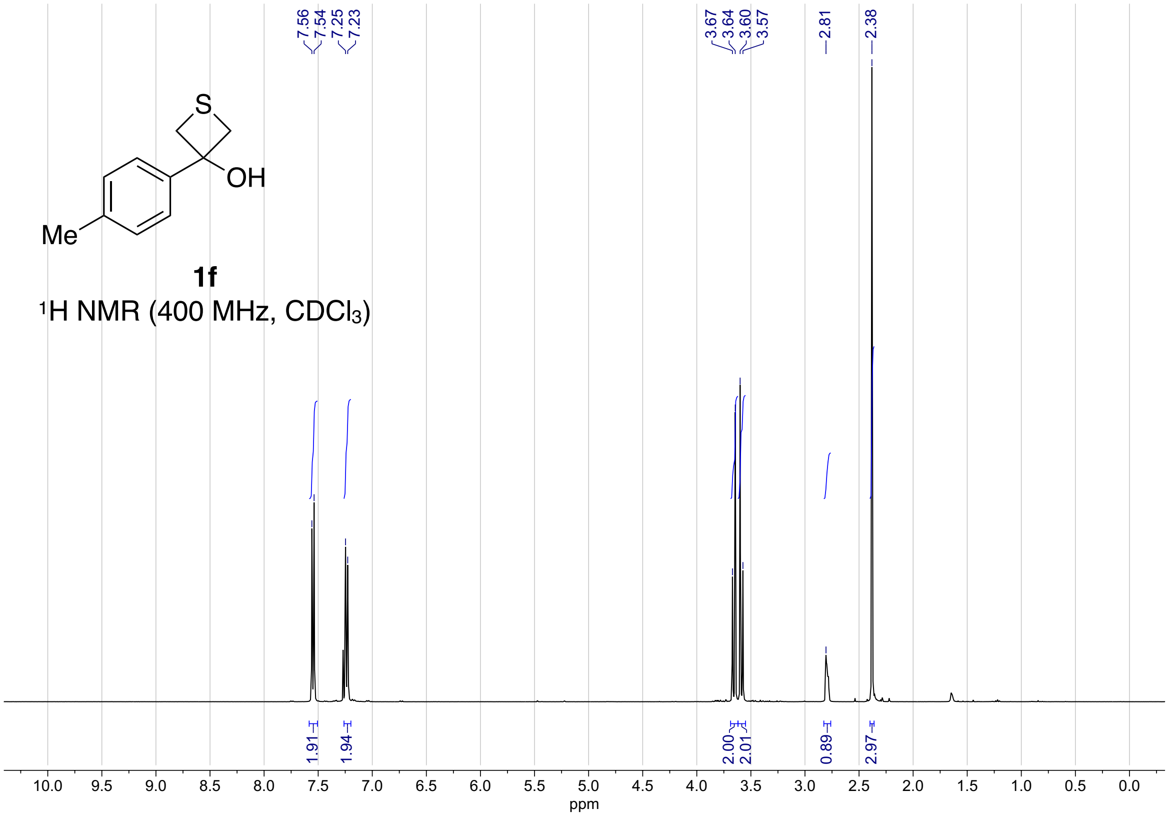
S
OH
Me
1f
1H NMR (400 MHz, CDCl3)
7.56
7.54
7.25
7.23
3.67
3.64
3.60
3.57
2.81
2.38
1.91
1.94
2.00
2.01
0.89
2.97
ppm

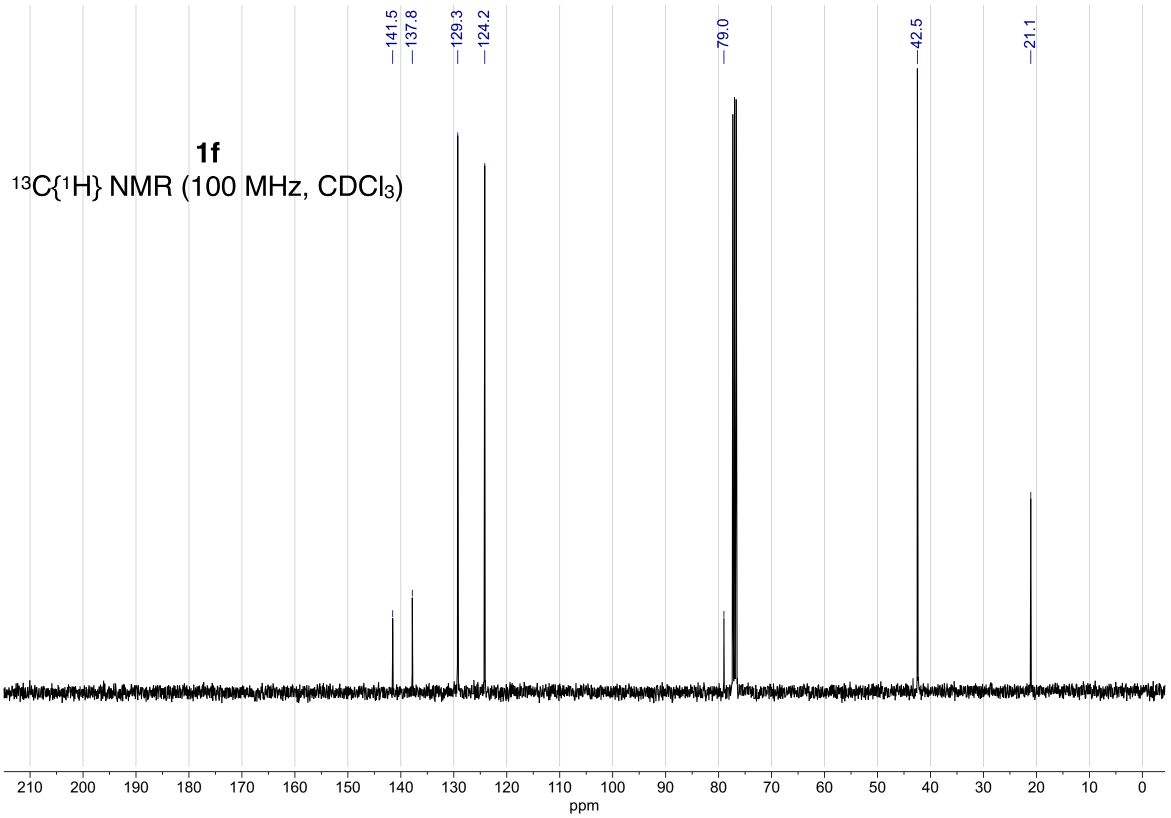
1f
13C{1H} NMR (100 MHz, CDCl3)
141.5
137.8
129.3
124.2
79.0
42.5
21.1
ppm

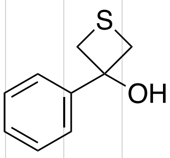

1g
^{1}H NMR (400 MHz, $CDCl_3$)

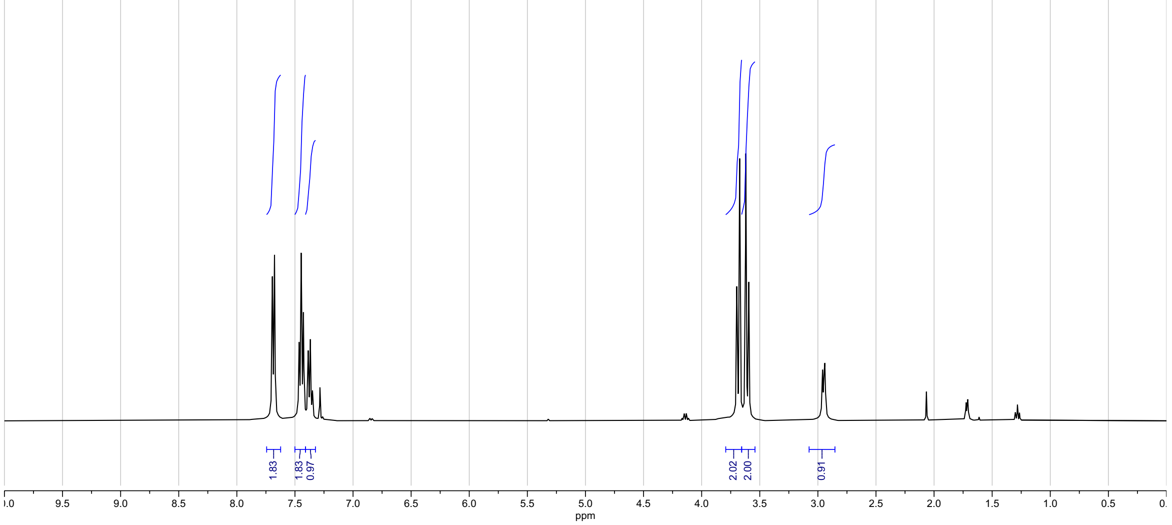

1g
^{13}C{^{1}H} NMR (100 MHz, $CDCl_3$)

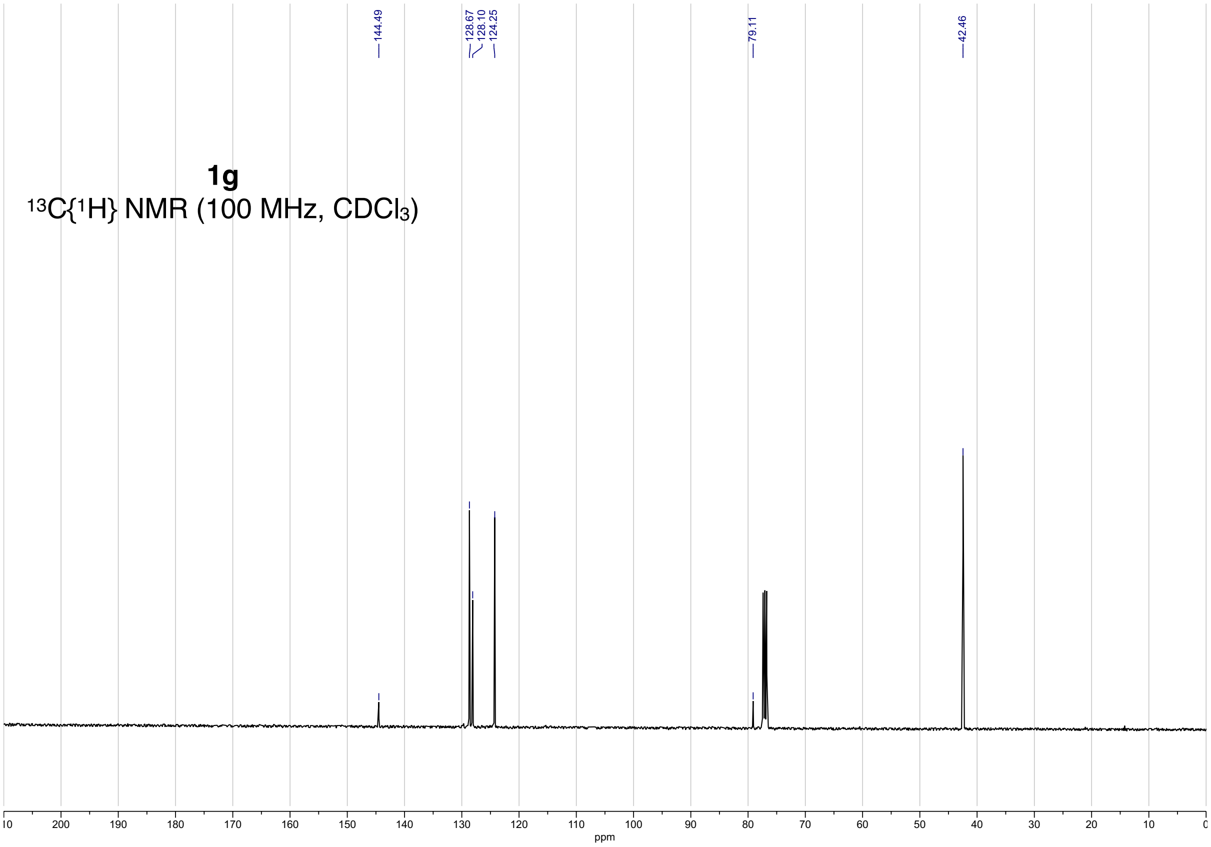

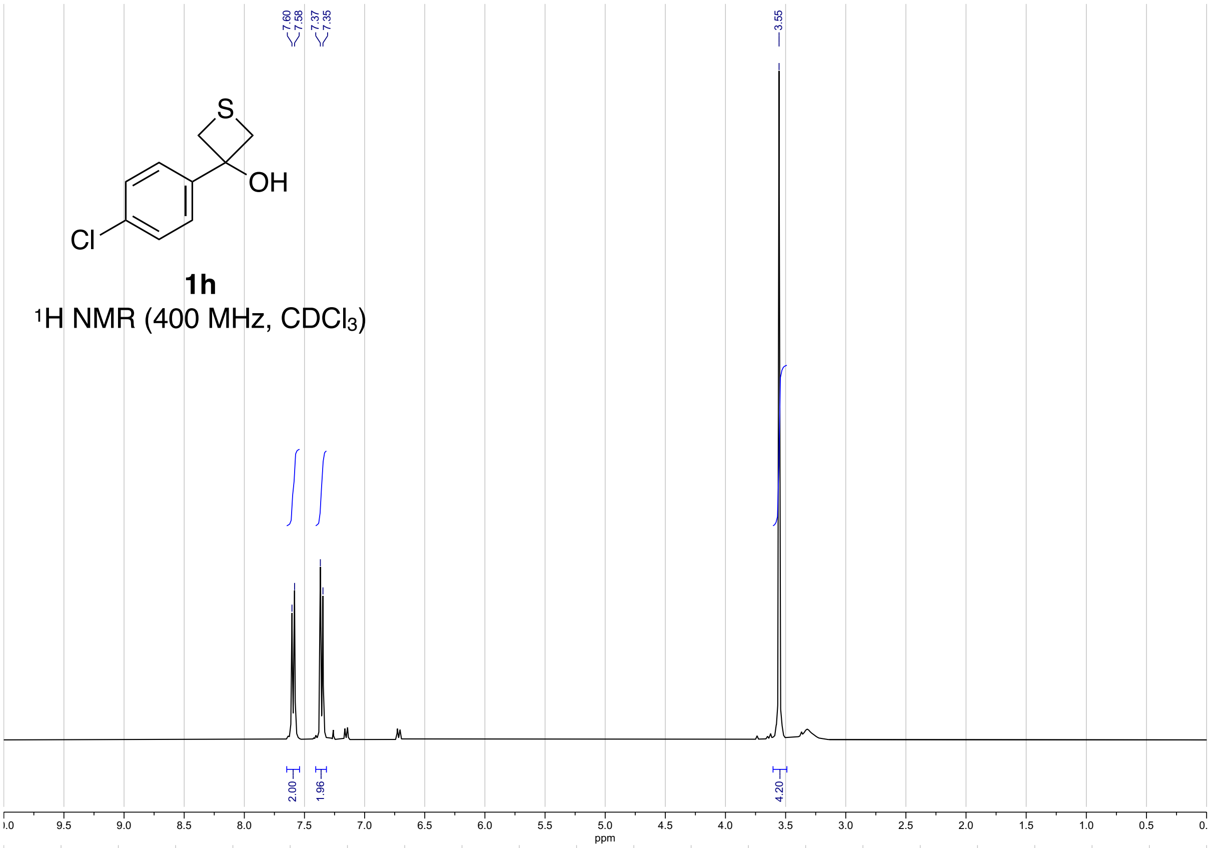
7.60
7.58
7.37
7.35
3.55
S
OH
Cl
1h
1H NMR (400 MHz, CDCl3)
2.00
1.96
4.20
ppm

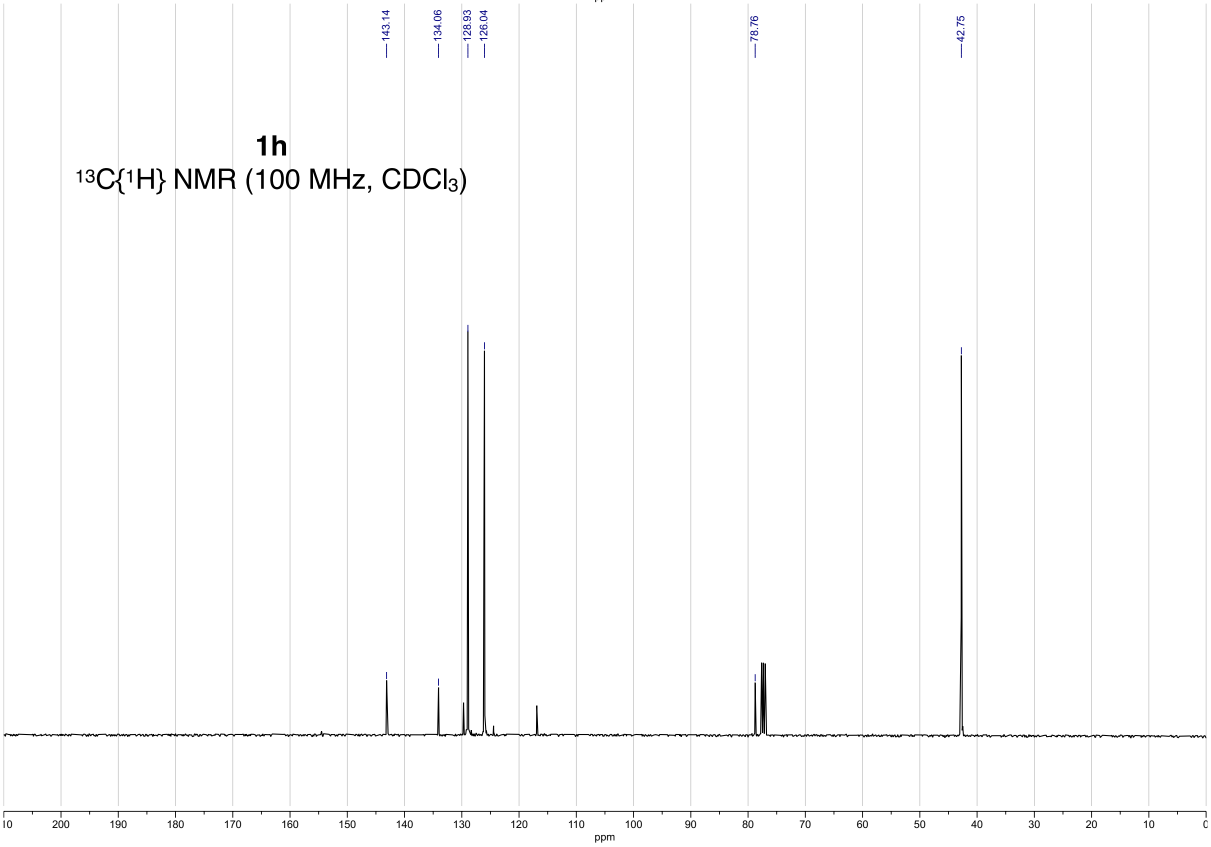
143.14
134.06
128.93
126.04
78.76
42.75
1h
13C{1H} NMR (100 MHz, CDCl3)
ppm

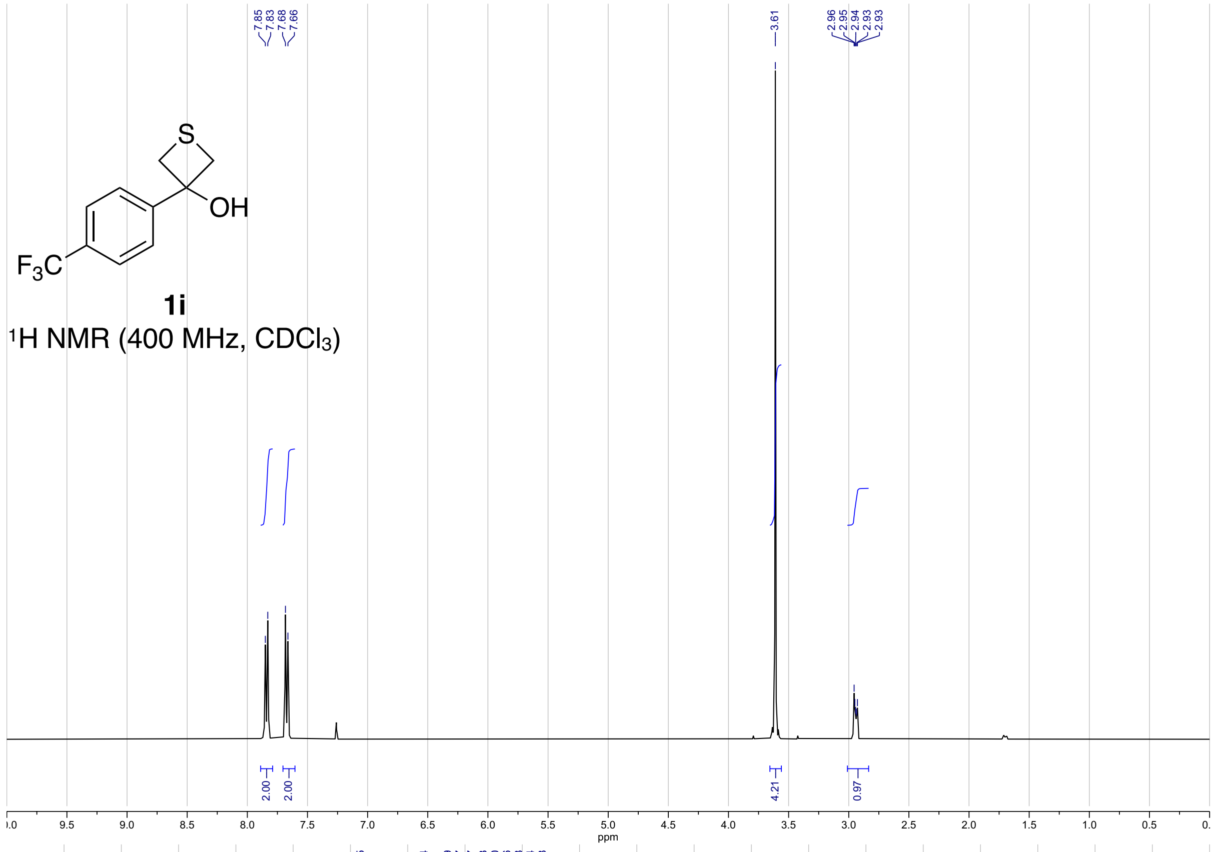
7.85
7.83
7.68
7.66
3.61
2.96
2.95
2.94
2.93
2.93
S
OH
F3C
1i
1H NMR (400 MHz, CDCl3)
2.00
2.00
4.21
0.97
ppm

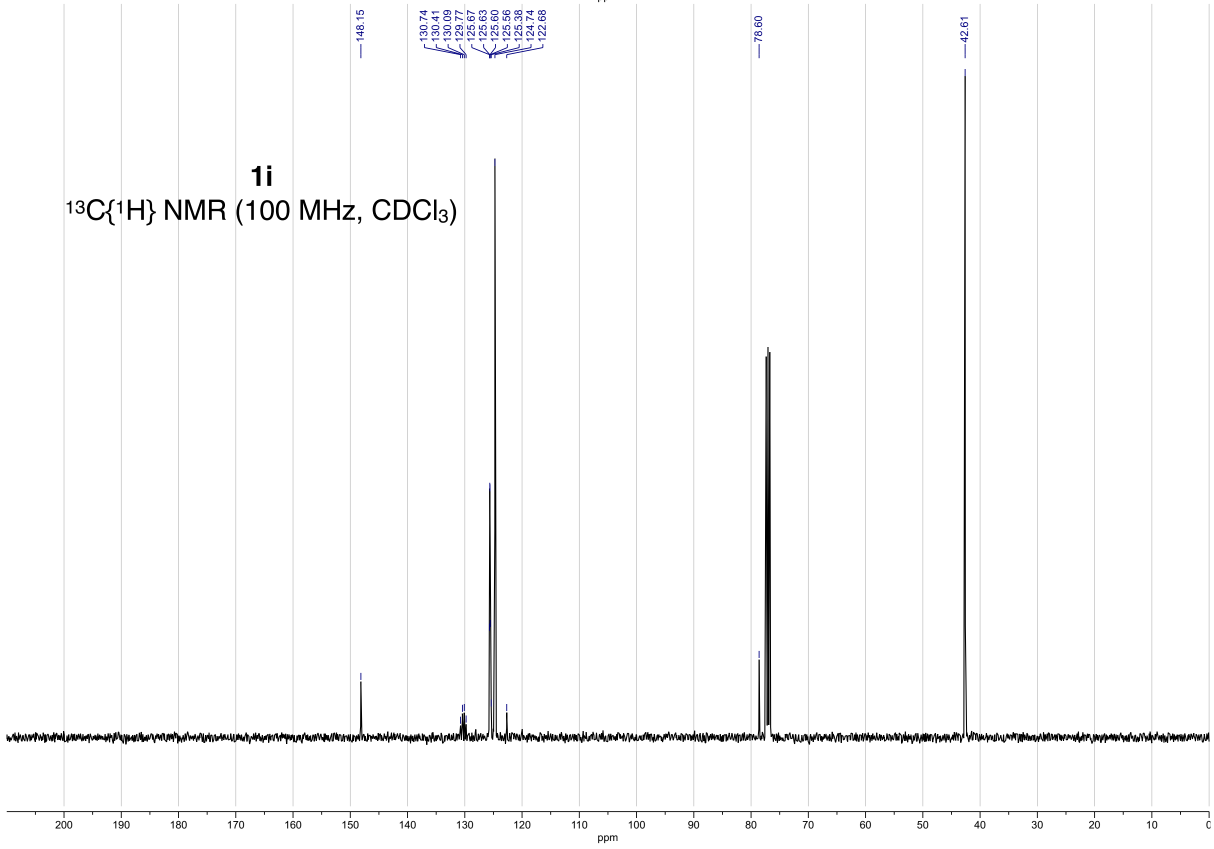
148.15
130.74
130.41
130.09
129.77
125.67
125.63
125.60
125.56
125.38
124.74
122.68
78.60
42.61
1i
13C{1H} NMR (100 MHz, CDCl3)
ppm

1i

^{19}F NMR (377 MHz, $CDCl_3$)

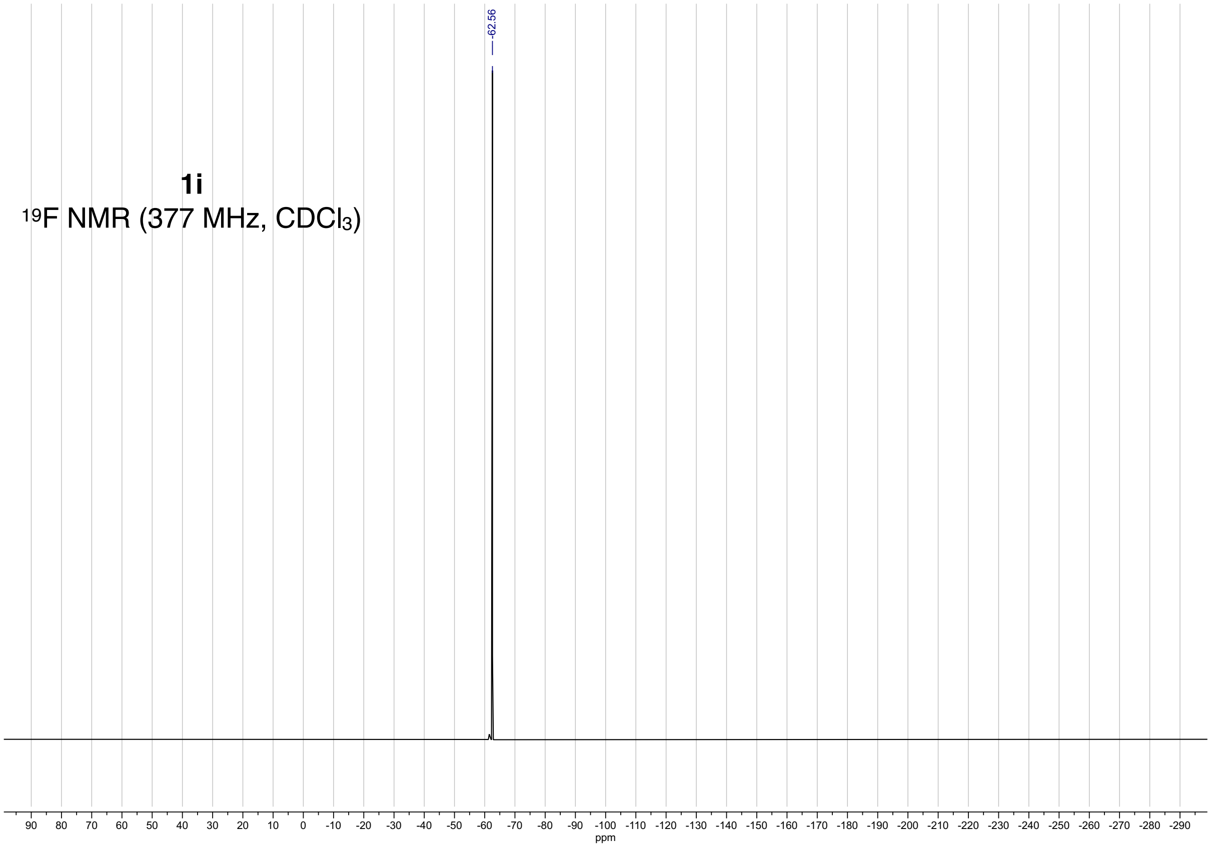

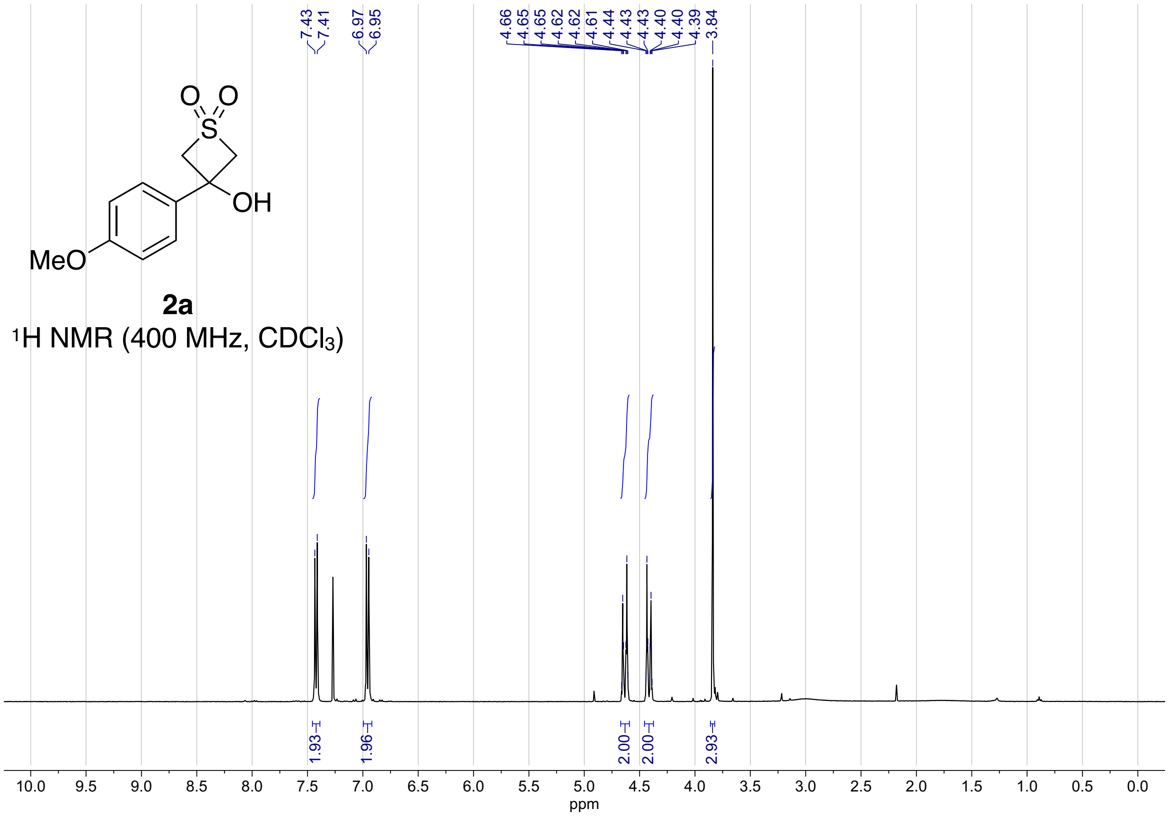
7.43
7.41
6.97
6.95
4.66
4.65
4.65
4.62
4.62
4.61
4.44
4.43
4.43
4.40
4.40
4.39
3.84
O O
S
OH
MeO
2a
1H NMR (400 MHz, CDCl3)
1.93
1.96
2.00
2.00
2.93
ppm

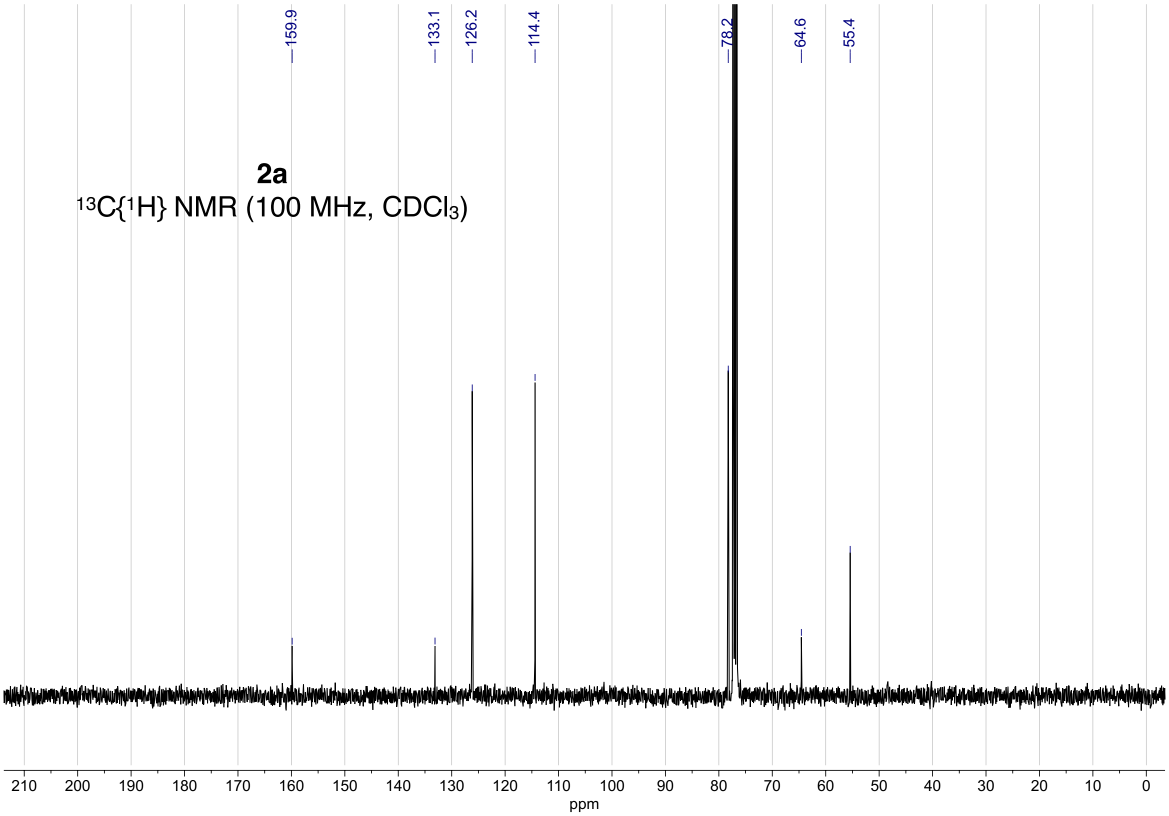
159.9
133.1
126.2
114.4
78.2
64.6
55.4
2a
13C{1H} NMR (100 MHz, CDCl3)
ppm

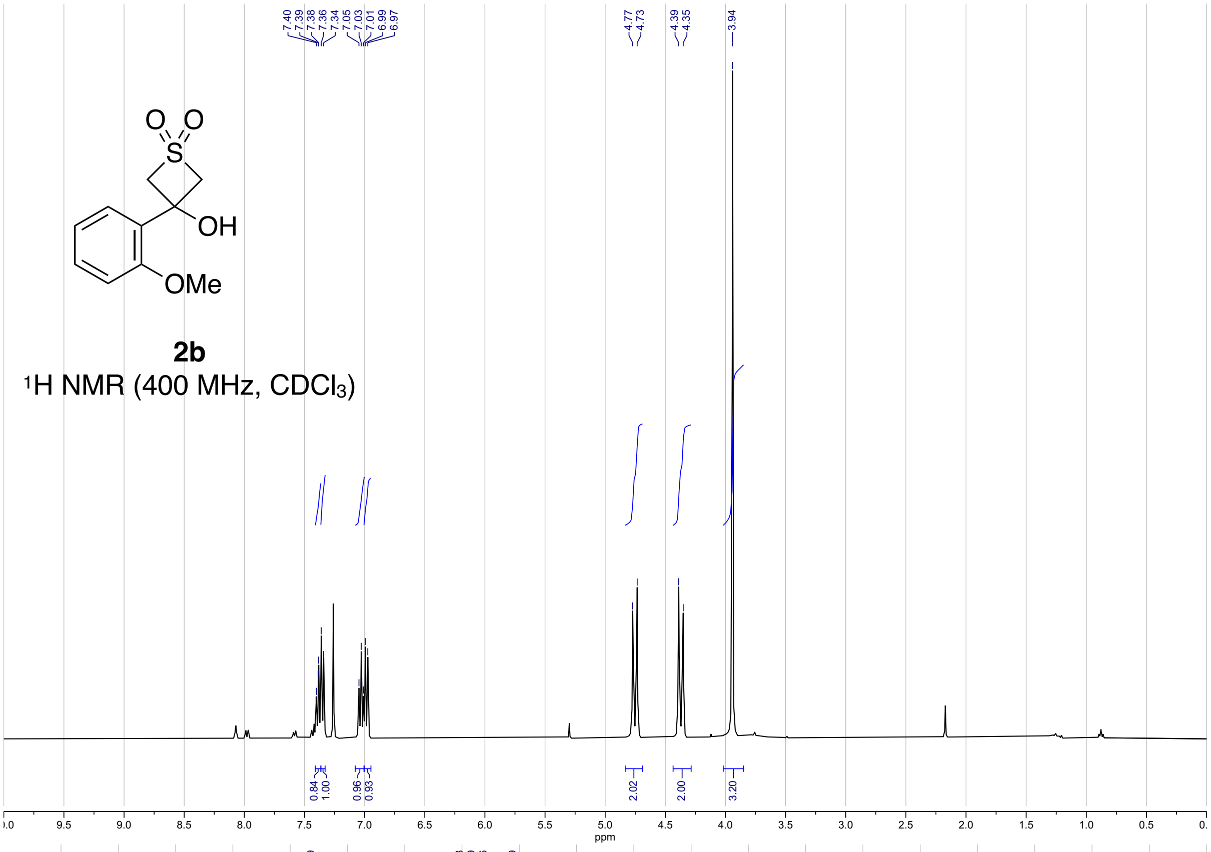
7.40
7.39
7.38
7.36
7.34
7.05
7.03
7.01
6.99
6.97
4.77
4.73
4.39
4.35
3.94
O
O
S
OH
OMe
2b
1H NMR (400 MHz, CDCl3)
0.84
1.00
0.96
0.93
2.02
2.00
3.20
ppm

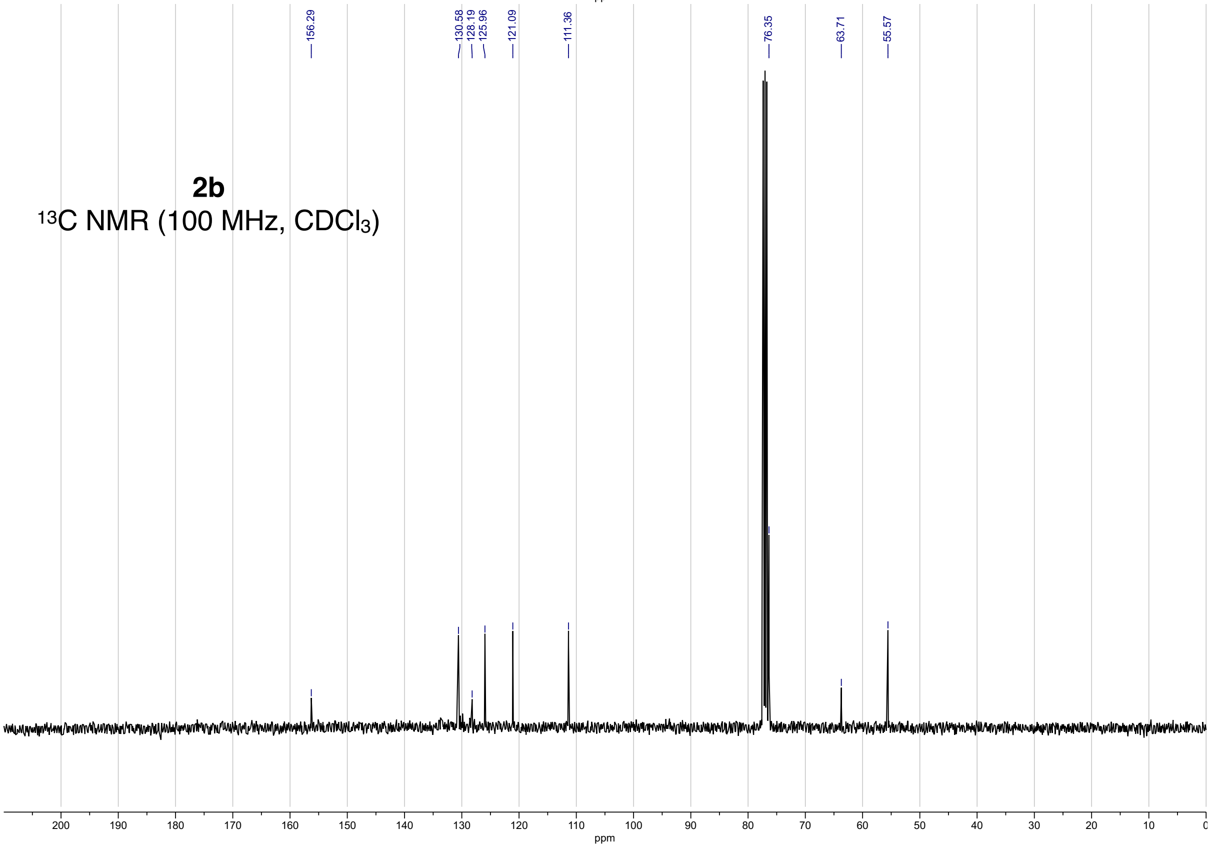
156.29
130.58
128.19
125.96
121.09
111.36
76.35
63.71
55.57
2b
13C NMR (100 MHz, CDCl3)
ppm

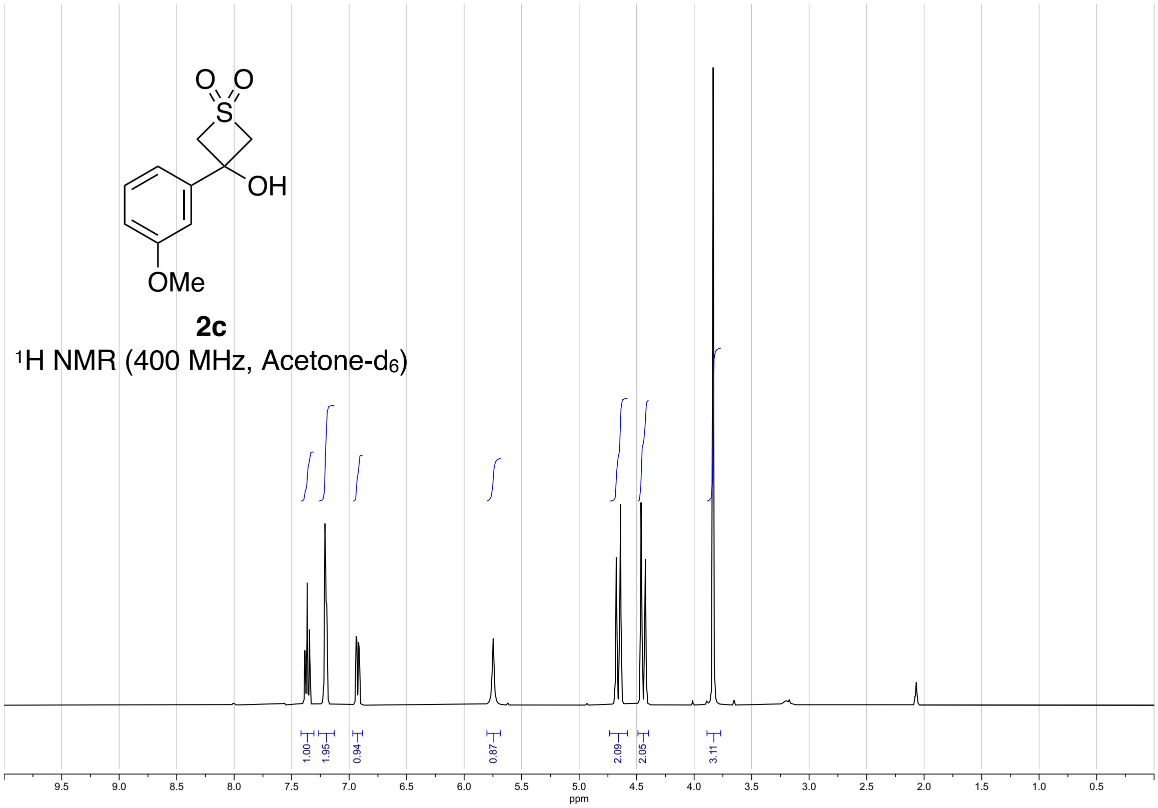
OH
OMe
2c
1H NMR (400 MHz, Acetone-d6)
1.00
1.95
0.94
0.87
2.09
2.05
3.11
ppm

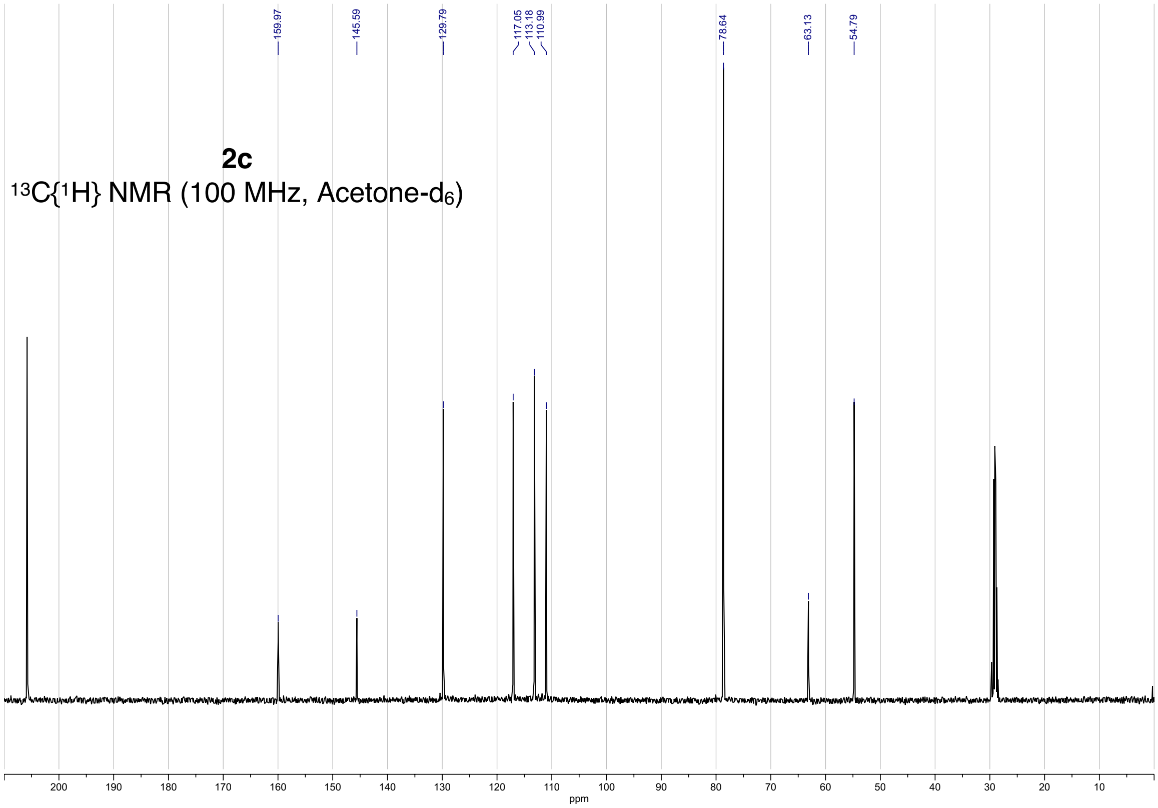
159.97
145.59
129.79
117.05
113.18
110.99
78.64
63.13
54.79
2c
13C{1H} NMR (100 MHz, Acetone-d6)
ppm

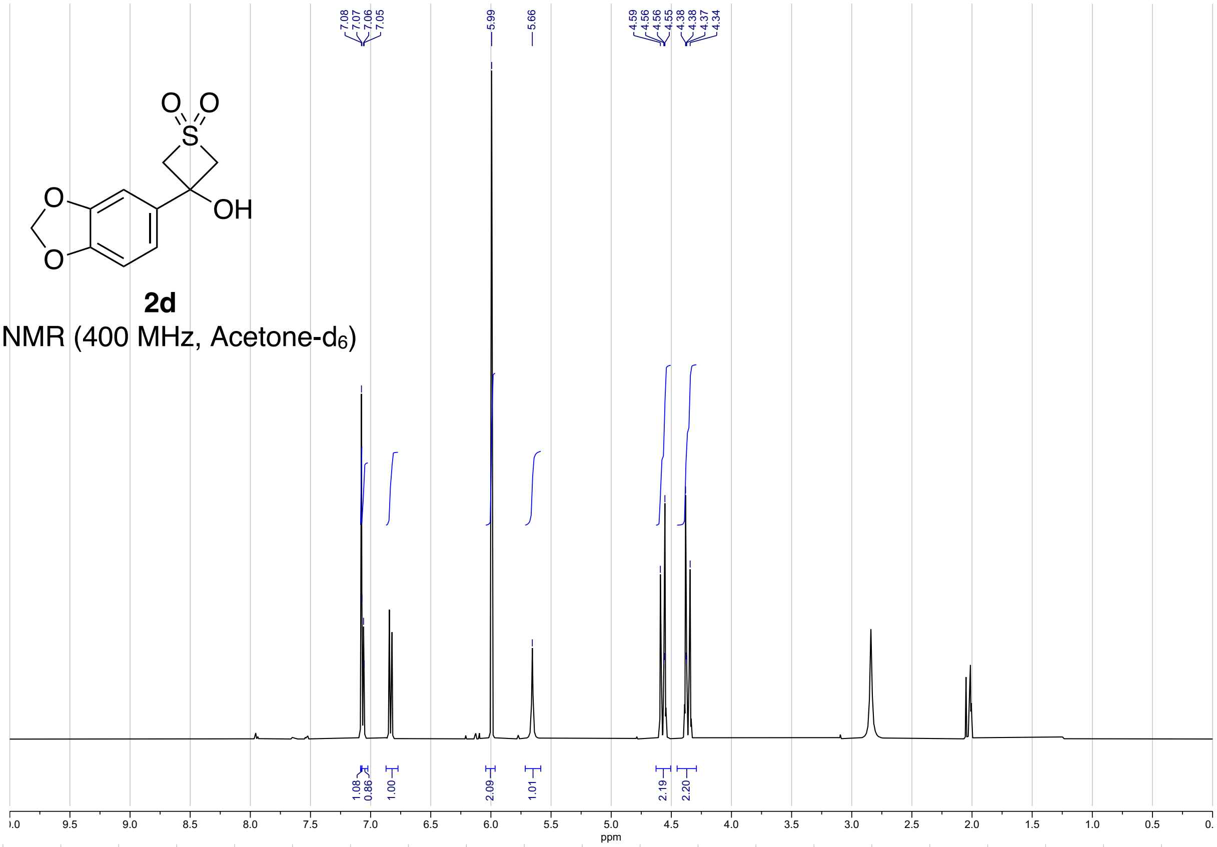

2d
^{1}H NMR (400 MHz, Acetone-d_6)

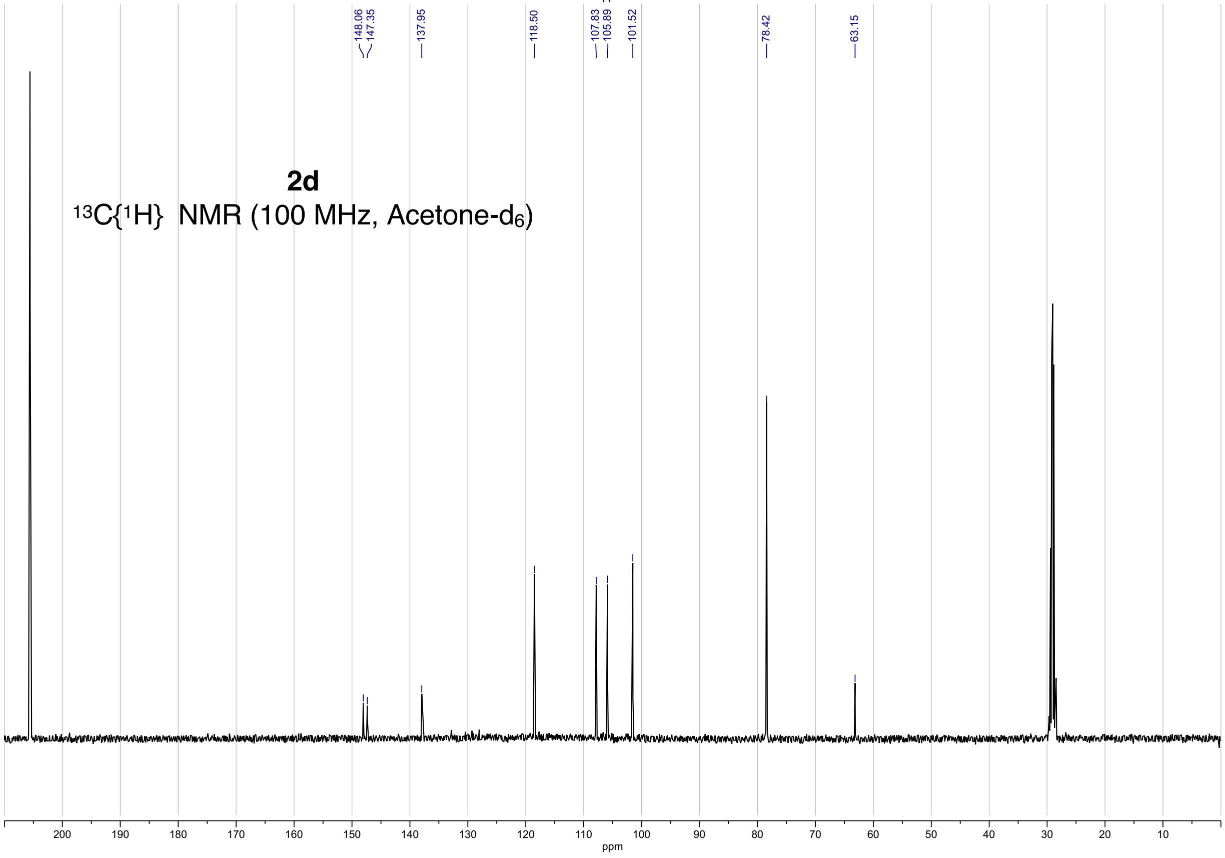

2d
^{13}C{^{1}H} NMR (100 MHz, Acetone-d_6)

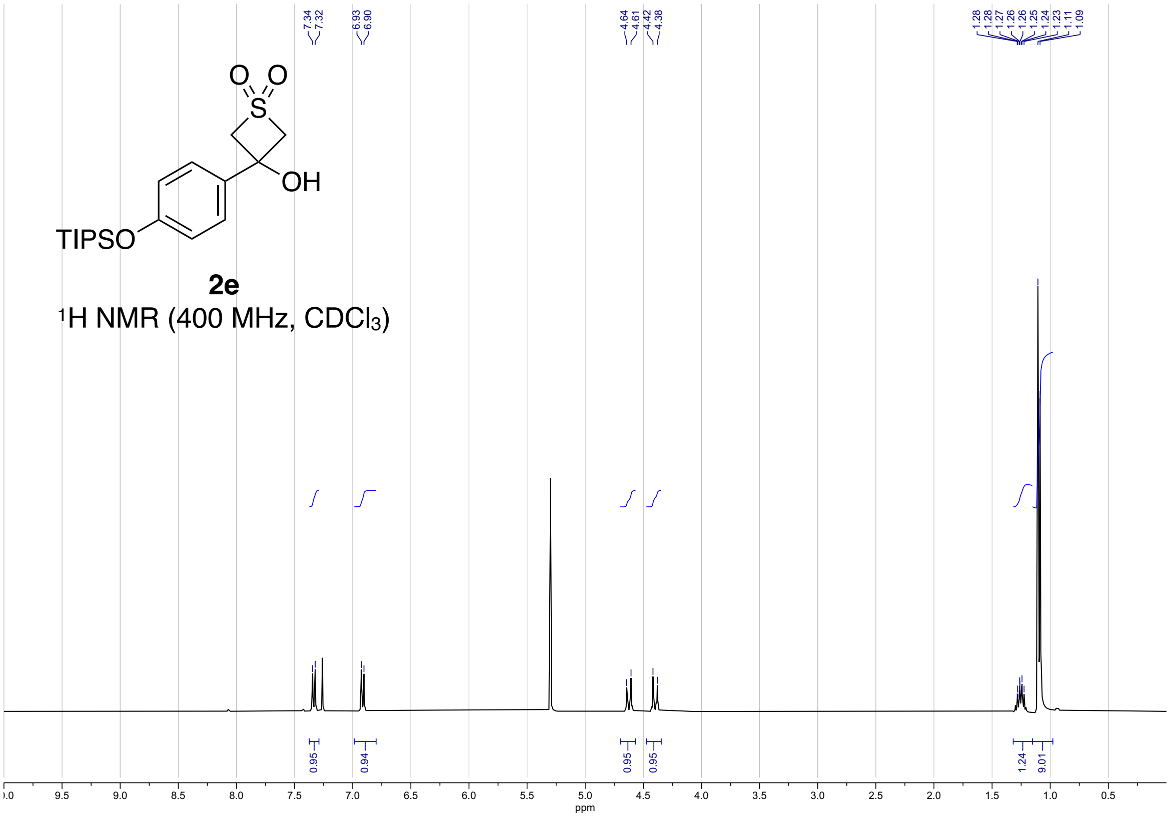
7.34
7.32
6.93
6.90
4.64
4.61
4.42
4.38
1.28
1.28
1.27
1.26
1.26
1.25
1.24
1.23
1.11
1.09
TIPSO
OH
O
O
S
2e
¹H NMR (400 MHz, CDCl₃)
0.95
0.94
0.95
0.95
1.24
9.01
ppm

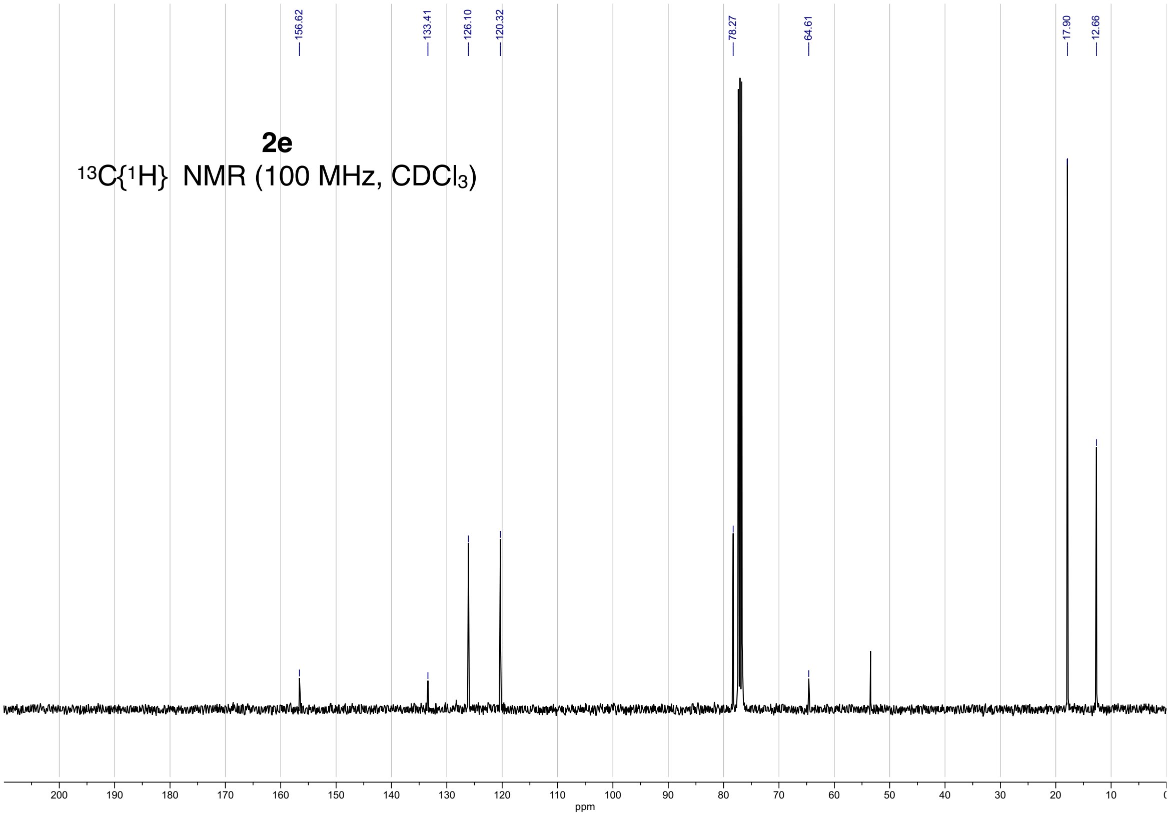
156.62
133.41
126.10
120.32
78.27
64.61
17.90
12.66
2e
¹³C{¹H} NMR (100 MHz, CDCl₃)
ppm

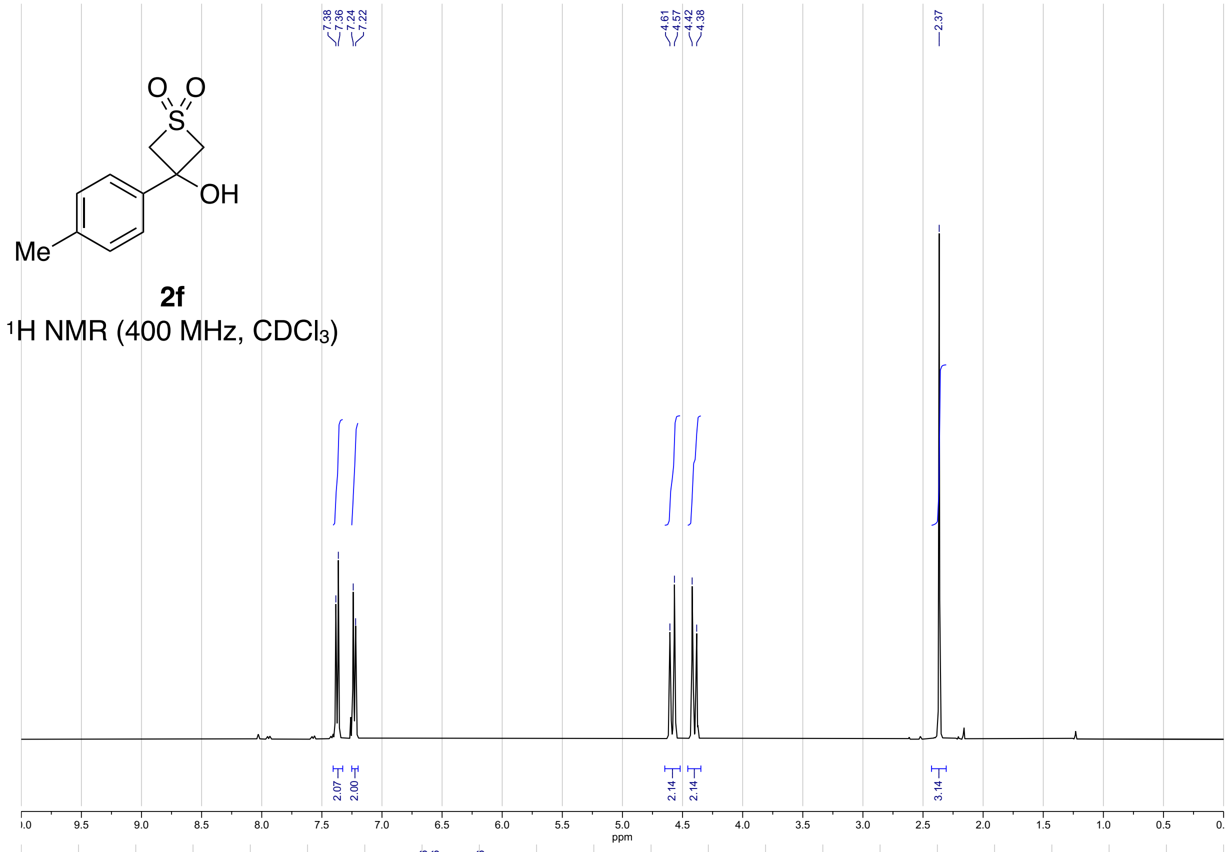
7.38
7.36
7.24
7.22
4.61
4.57
4.42
4.38
2.37
O O
S
OH
Me
2f
1H NMR (400 MHz, CDCl3)
2.07
2.00
2.14
2.14
3.14
ppm

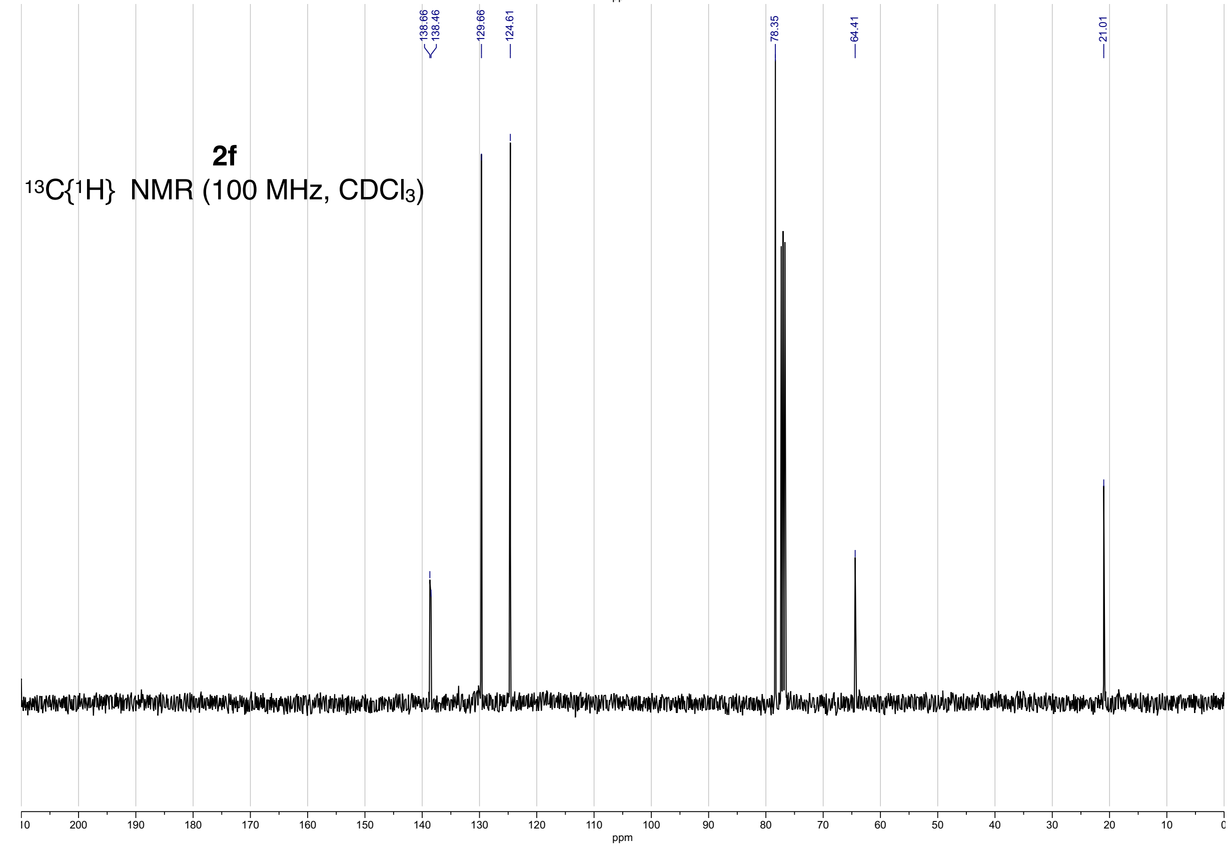
138.66
138.46
129.66
124.61
78.35
64.41
21.01
2f
13C{1H} NMR (100 MHz, CDCl3)
ppm

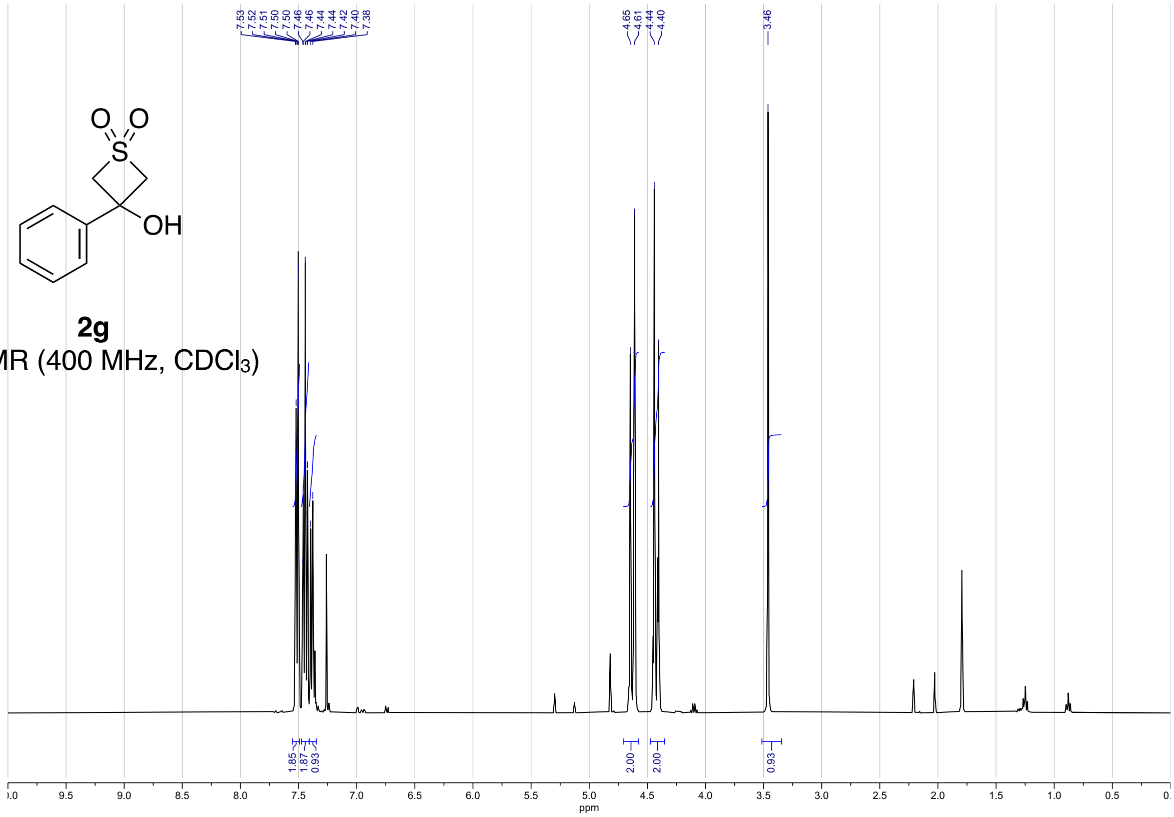

2g
^{1}H NMR (400 MHz, $CDCl_3$)

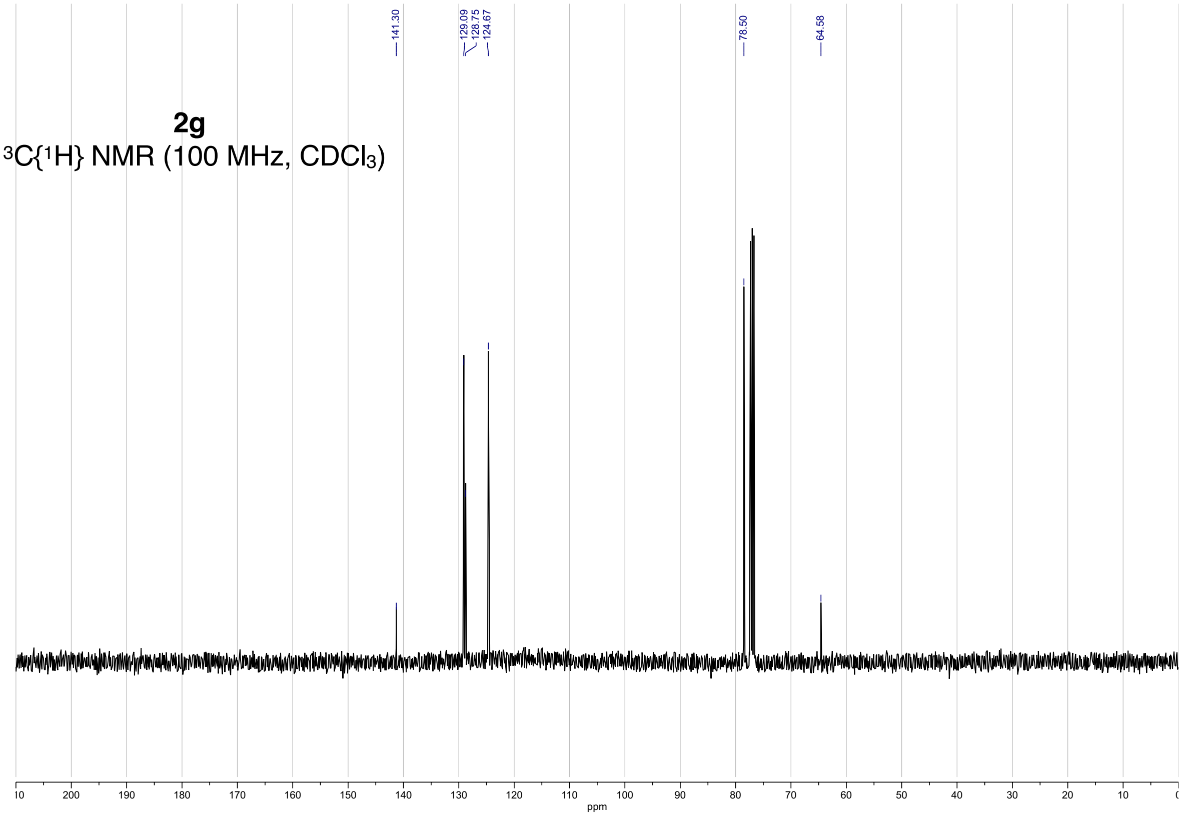

2g
$^{13}C\{^{1}H\}$ NMR (100 MHz, $CDCl_3$)

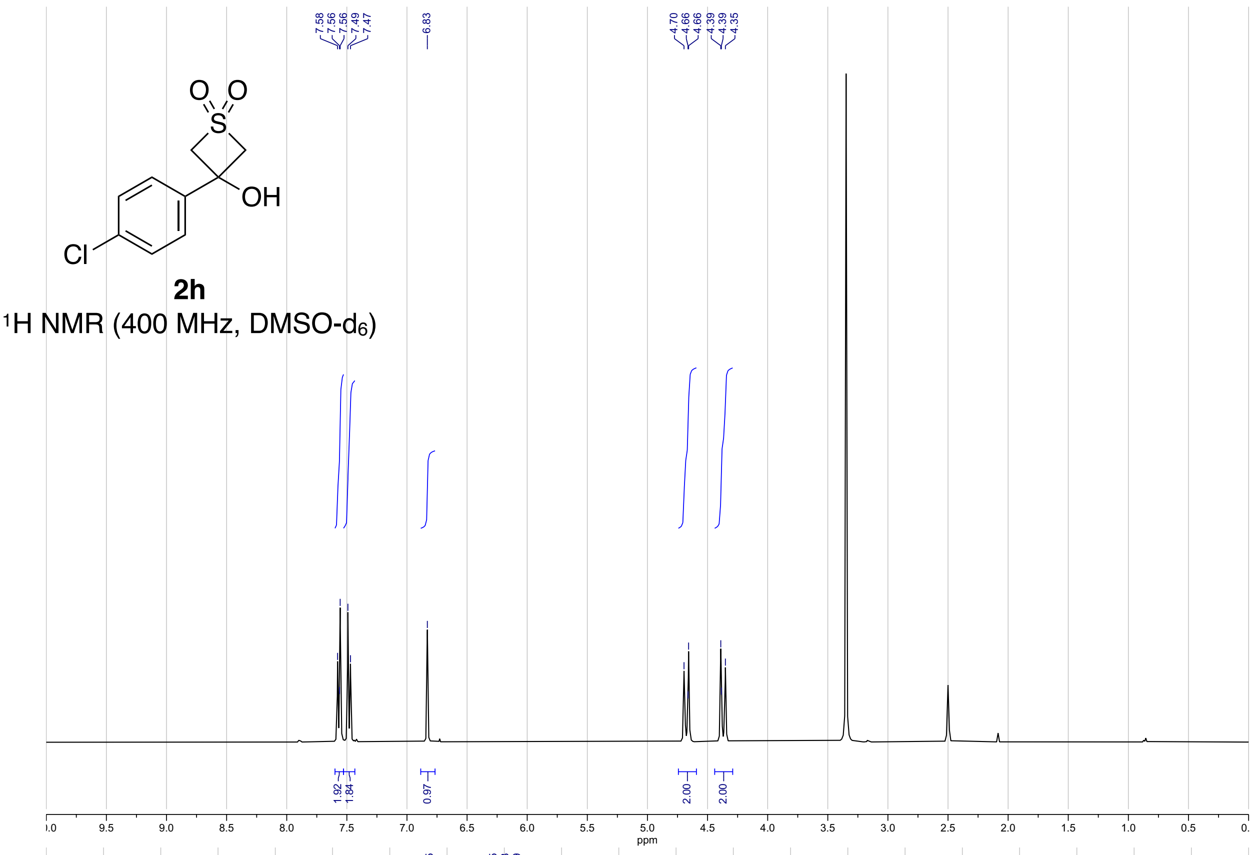

2h
^1H NMR (400 MHz, DMSO-d$_6$)
7.58 7.56 7.56 7.49 7.47
6.83
4.70 4.66 4.66 4.39 4.39 4.35
1.92 1.84 0.97 2.00 2.00
ppm

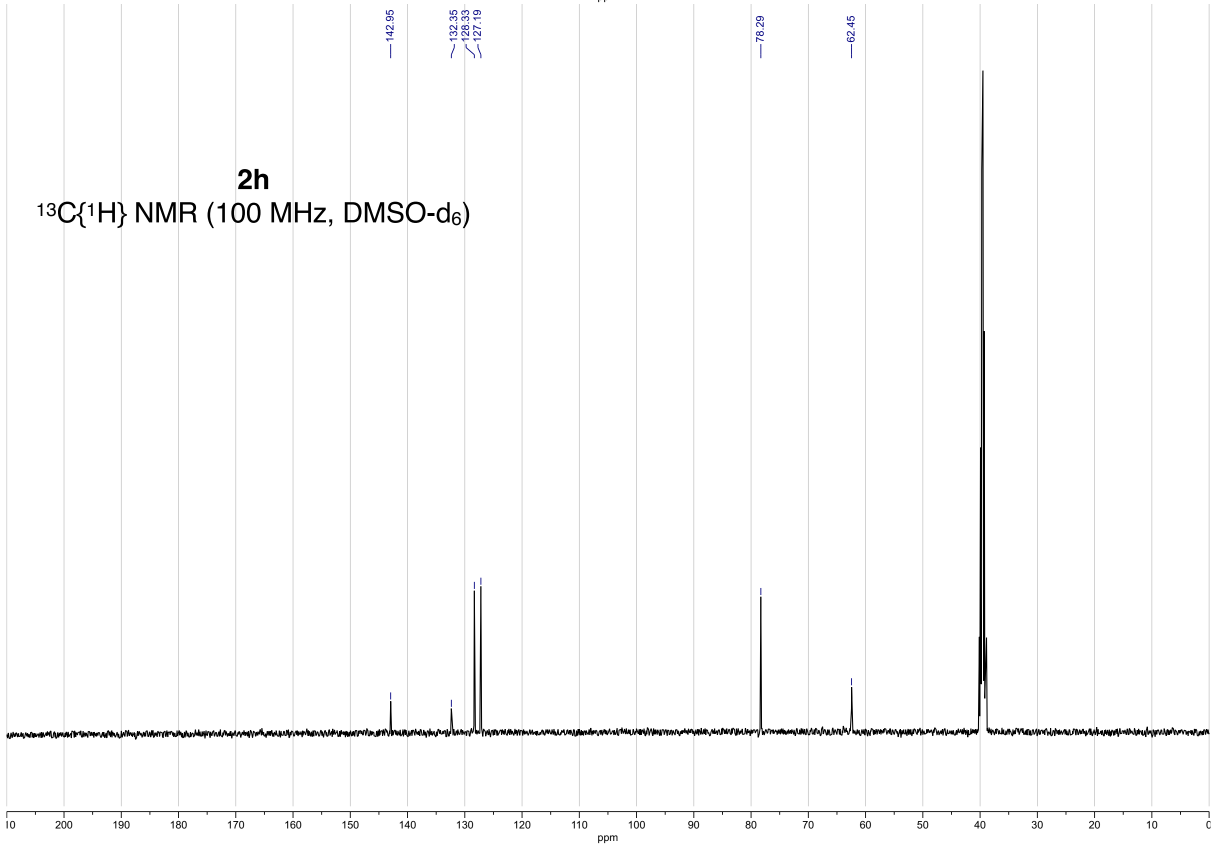

2h
$^{13}C\{^1H\}$ NMR (100 MHz, DMSO-d$_6$)
142.95
132.35 128.33 127.19
78.29
62.45
ppm

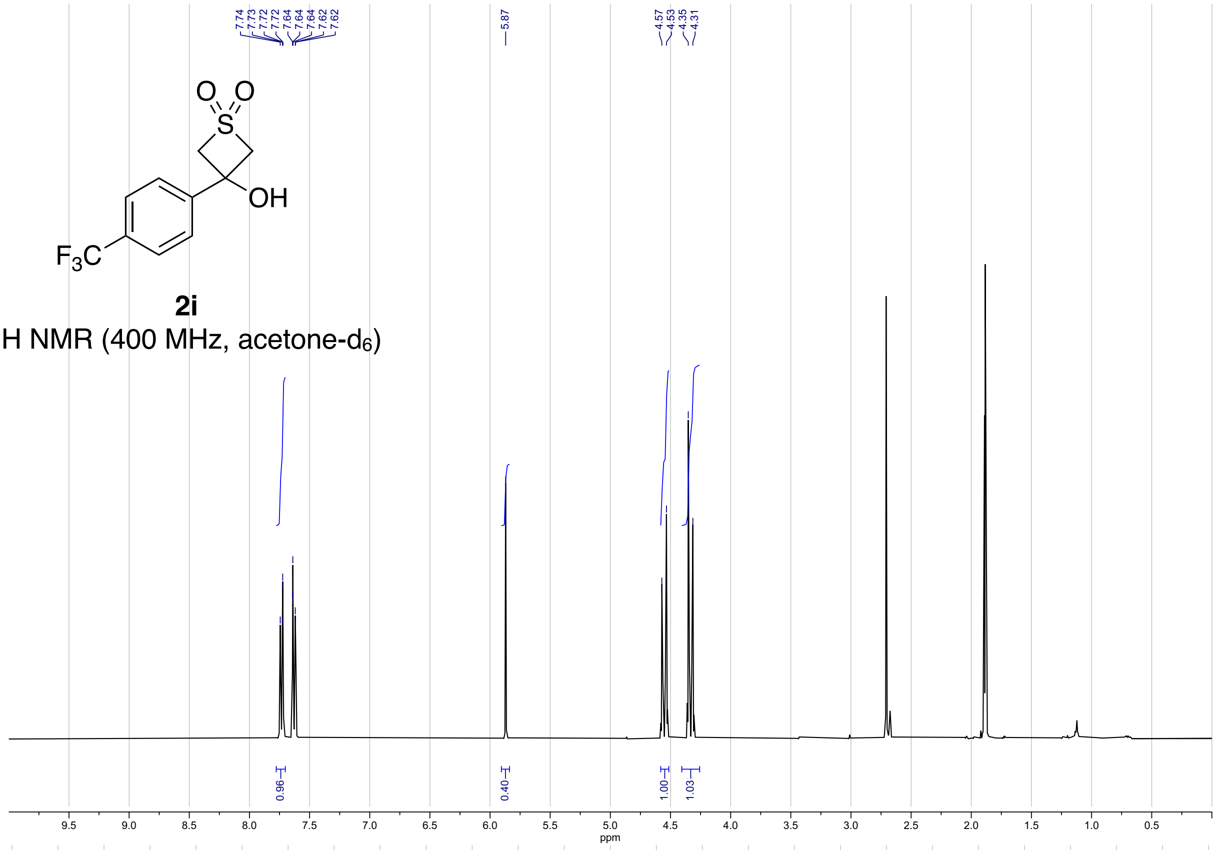
7.74
7.73
7.72
7.72
7.64
7.64
7.64
7.62
7.62
5.87
4.57
4.53
4.35
4.31
O O
S
OH
F_3C
2i
^{1}H NMR (400 MHz, acetone-d_6)
0.96
0.40
1.00
1.03
ppm

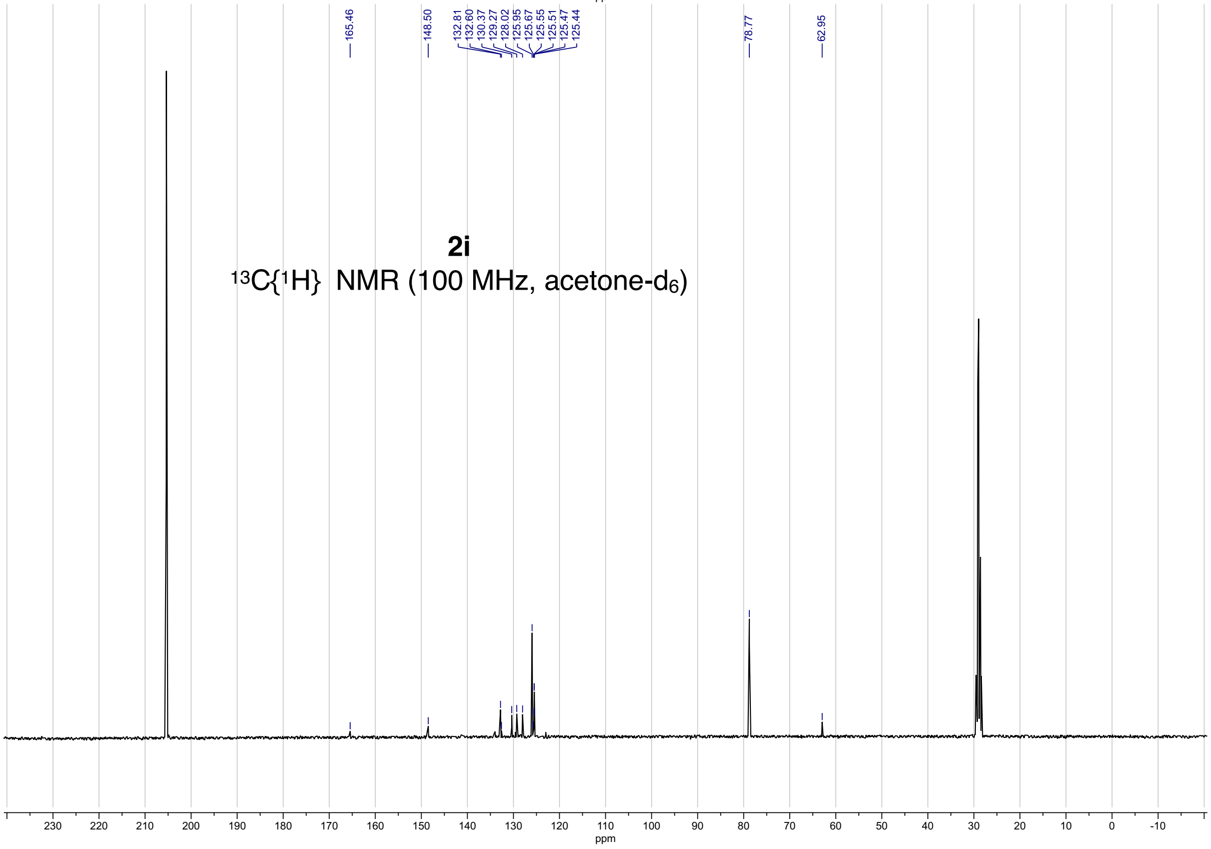
165.46
148.50
132.81
132.60
130.37
129.27
128.02
125.95
125.67
125.55
125.51
125.47
125.44
78.77
62.95
2i
^{13}C{^{1}H} NMR (100 MHz, acetone-d_6)
ppm

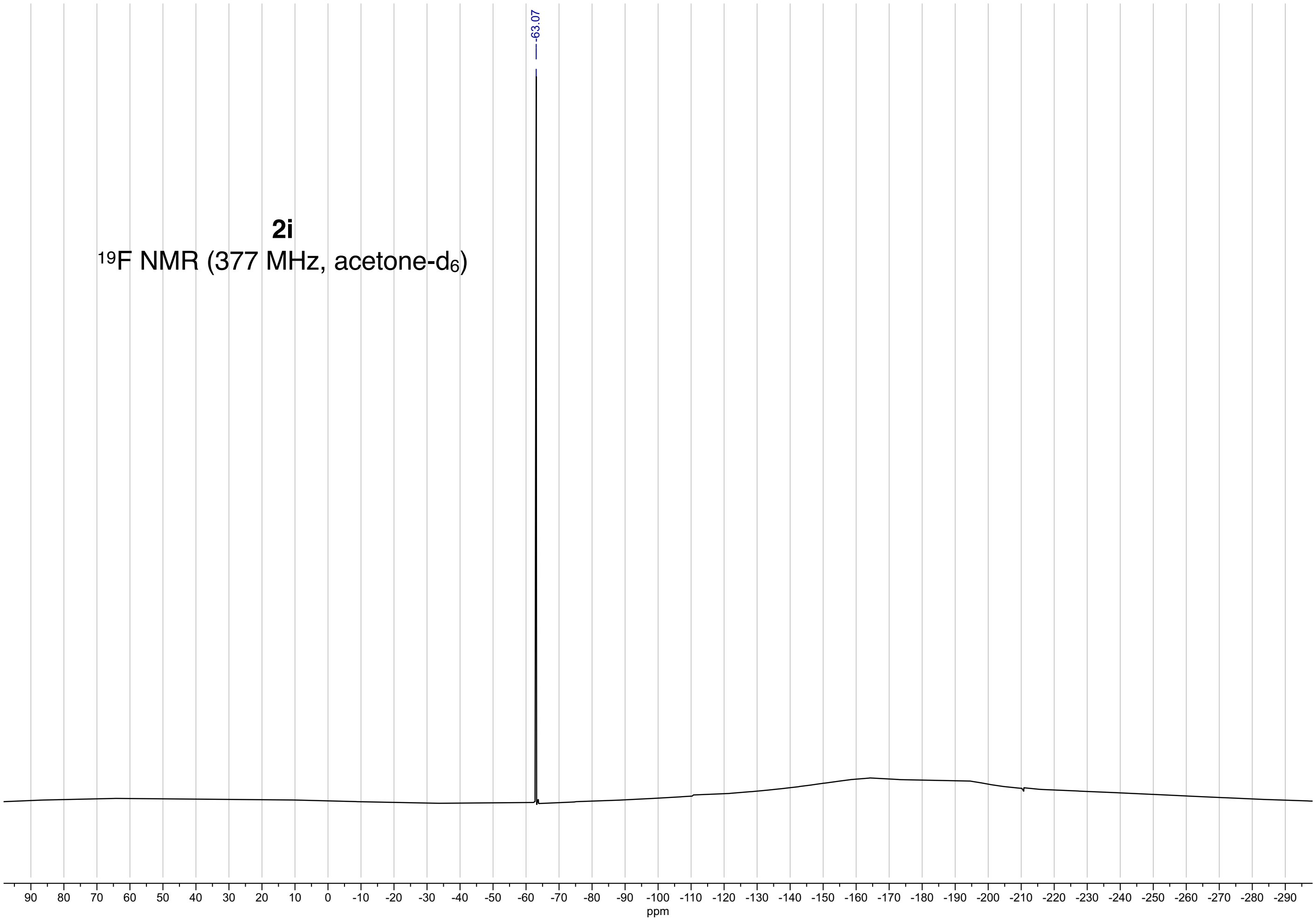
-63.07
2i
19F NMR (377 MHz, acetone-d6)
90 80 70 60 50 40 30 20 10 0 -10 -20 -30 -40 -50 -60 -70 -80 -90 -100 -110 -120 -130 -140 -150 -160 -170 -180 -190 -200 -210 -220 -230 -240 -250 -260 -270 -280 -290
ppm

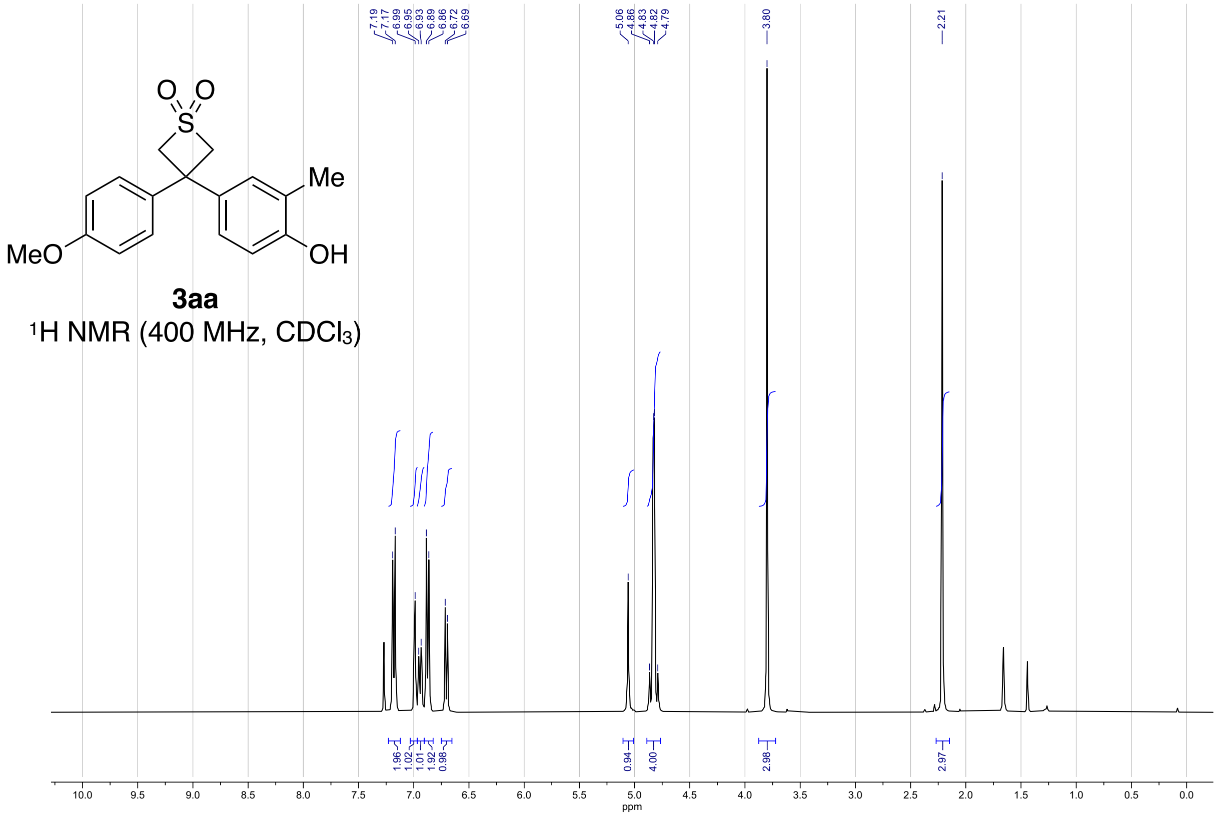

O O
S
Me
MeO
OH
3aa
1H NMR (400 MHz, CDCl3)
7.19 7.17 6.99 6.95 6.93 6.89 6.86 6.72 6.69
5.06 4.86 4.83 4.82 4.79
3.80
2.21
1.96 1.02 1.01 1.92 0.98
0.94 4.00
2.98
2.97
10.0 9.5 9.0 8.5 8.0 7.5 7.0 6.5 6.0 5.5 5.0 4.5 4.0 3.5 3.0 2.5 2.0 1.5 1.0 0.5 0.0
ppm

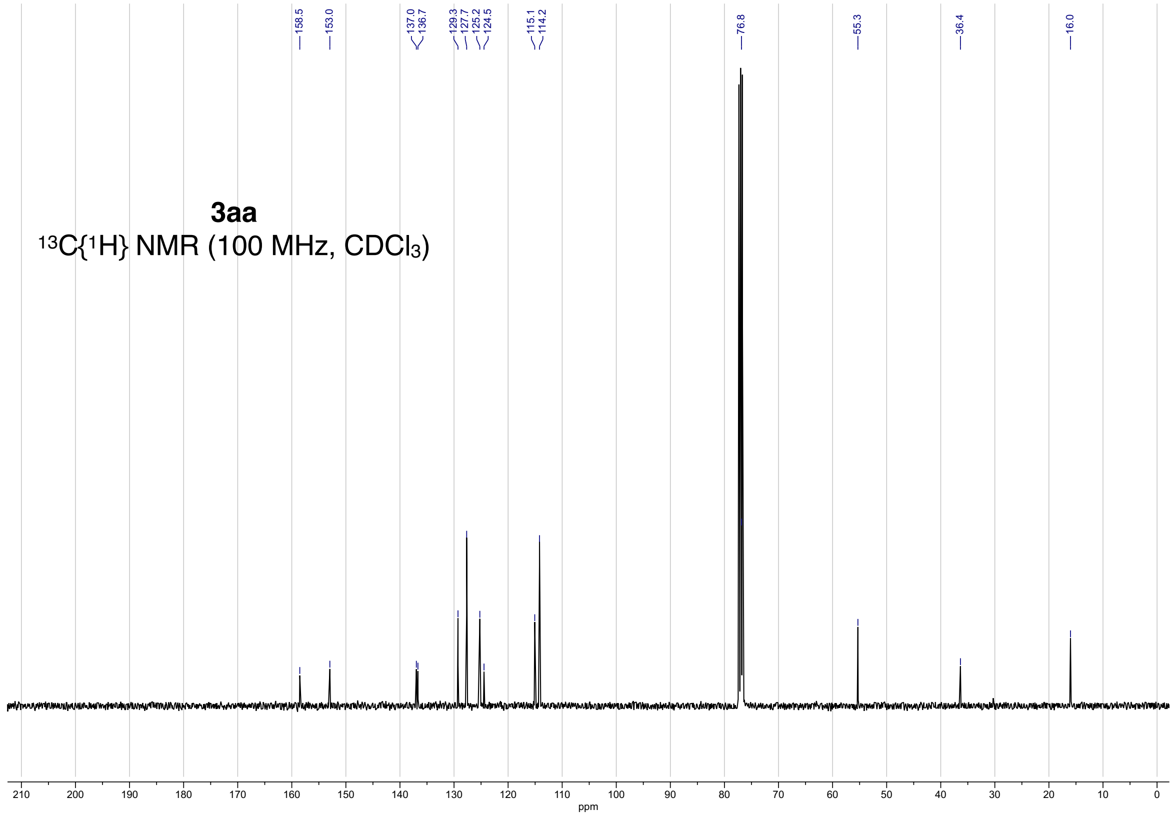

158.5
153.0
137.0 136.7
129.3 127.7 125.2 124.5
115.1 114.2
76.8
55.3
36.4
16.0
3aa
13C{1H} NMR (100 MHz, CDCl3)
210 200 190 180 170 160 150 140 130 120 110 100 90 80 70 60 50 40 30 20 10 0
ppm

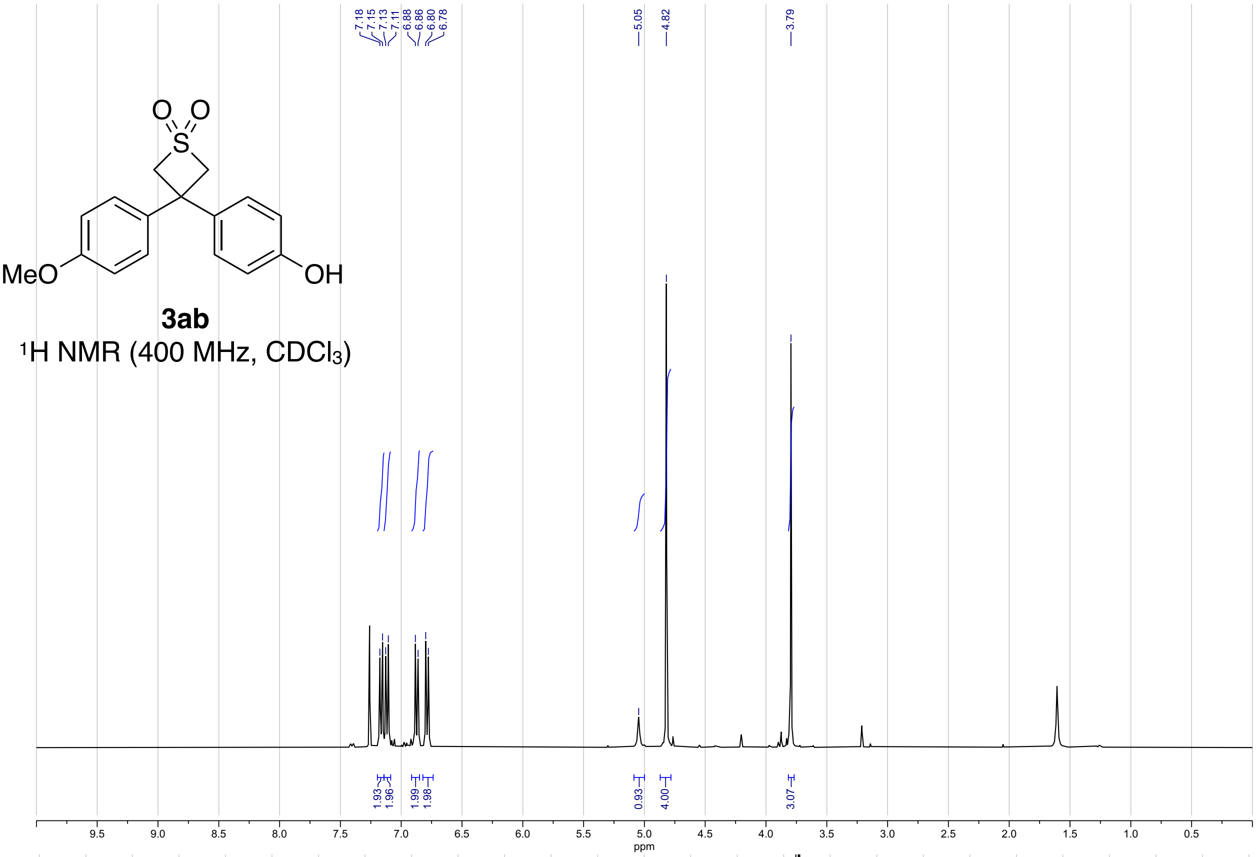

3ab
^1H NMR (400 MHz, $CDCl_3$)

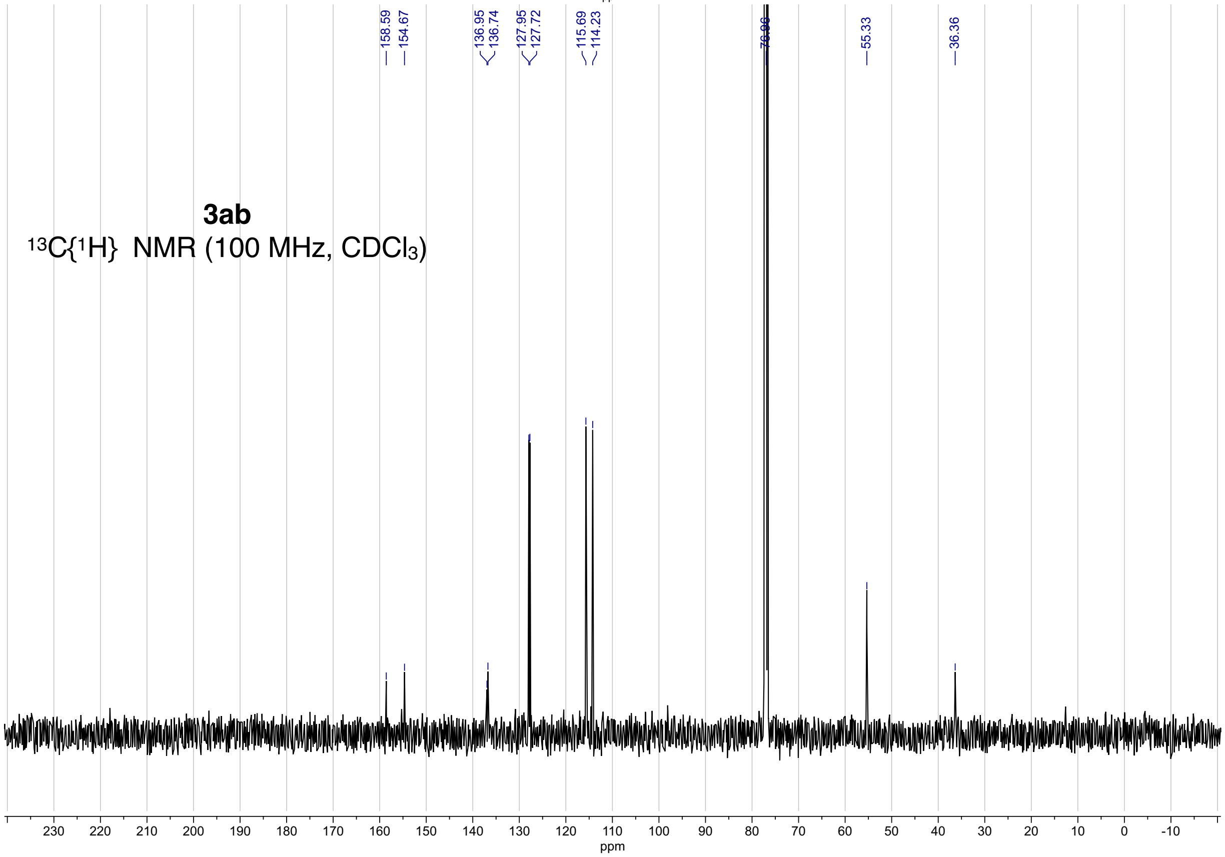

3ab
$^{13}C\{^1H\}$ NMR (100 MHz, $CDCl_3$)

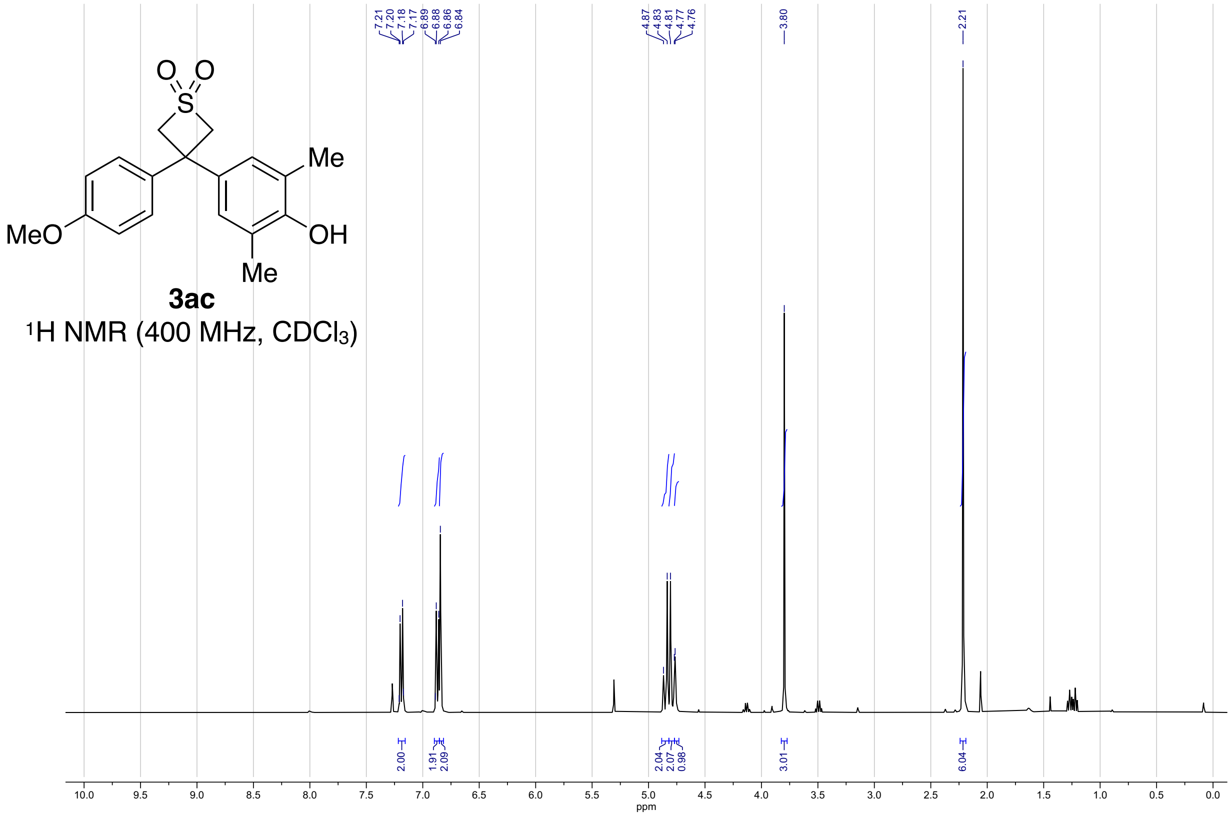

3ac

^{1}H NMR (400 MHz, $CDCl_3$)

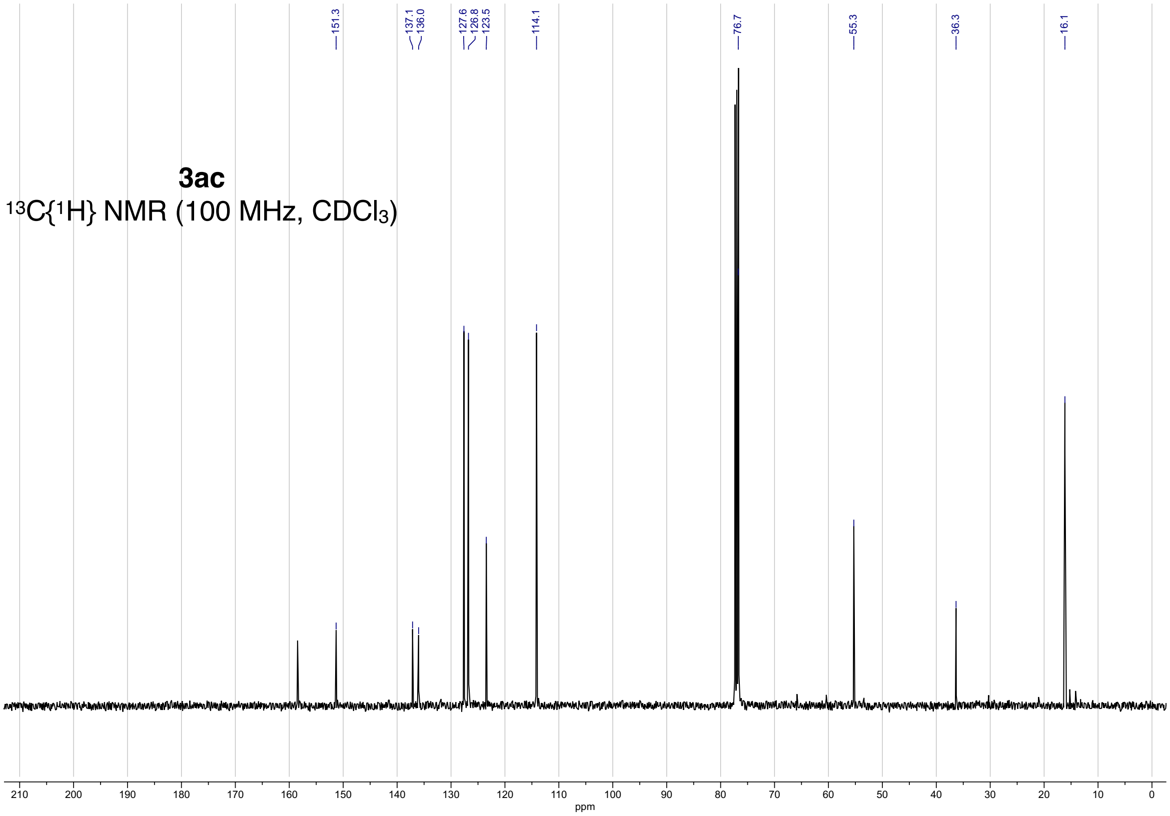

3ac

^{13}C{^{1}H} NMR (100 MHz, $CDCl_3$)

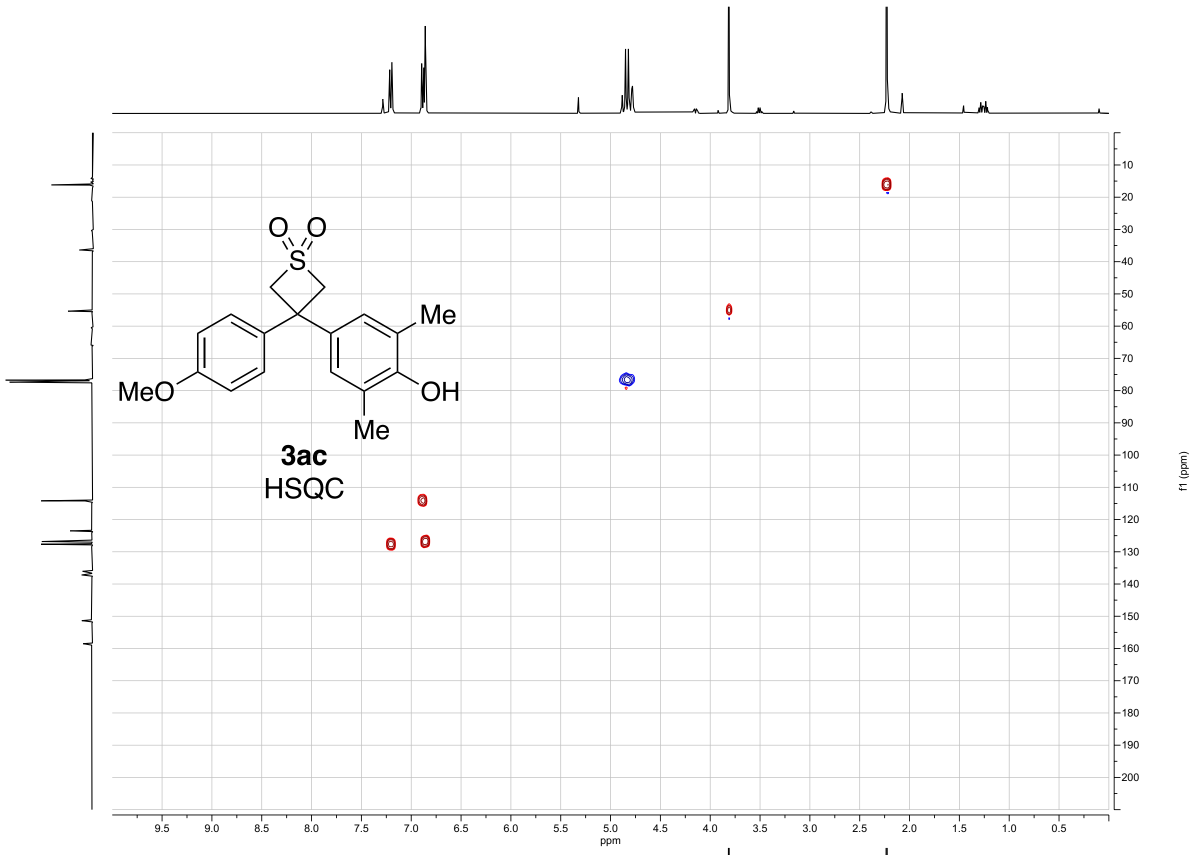

O
O
S
Me
MeO
OH
Me
3ac
HSQC
ppm
f1 (ppm)

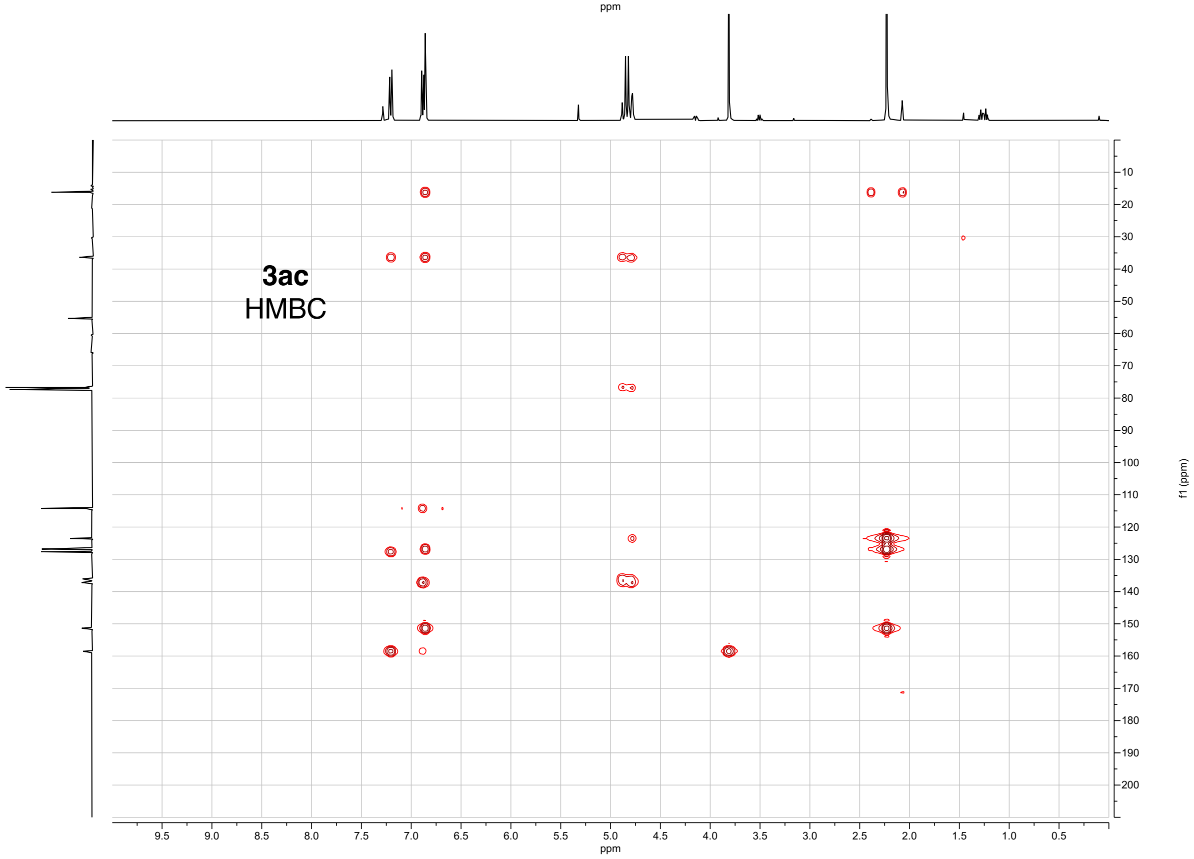

3ac
HMBC
ppm
f1 (ppm)

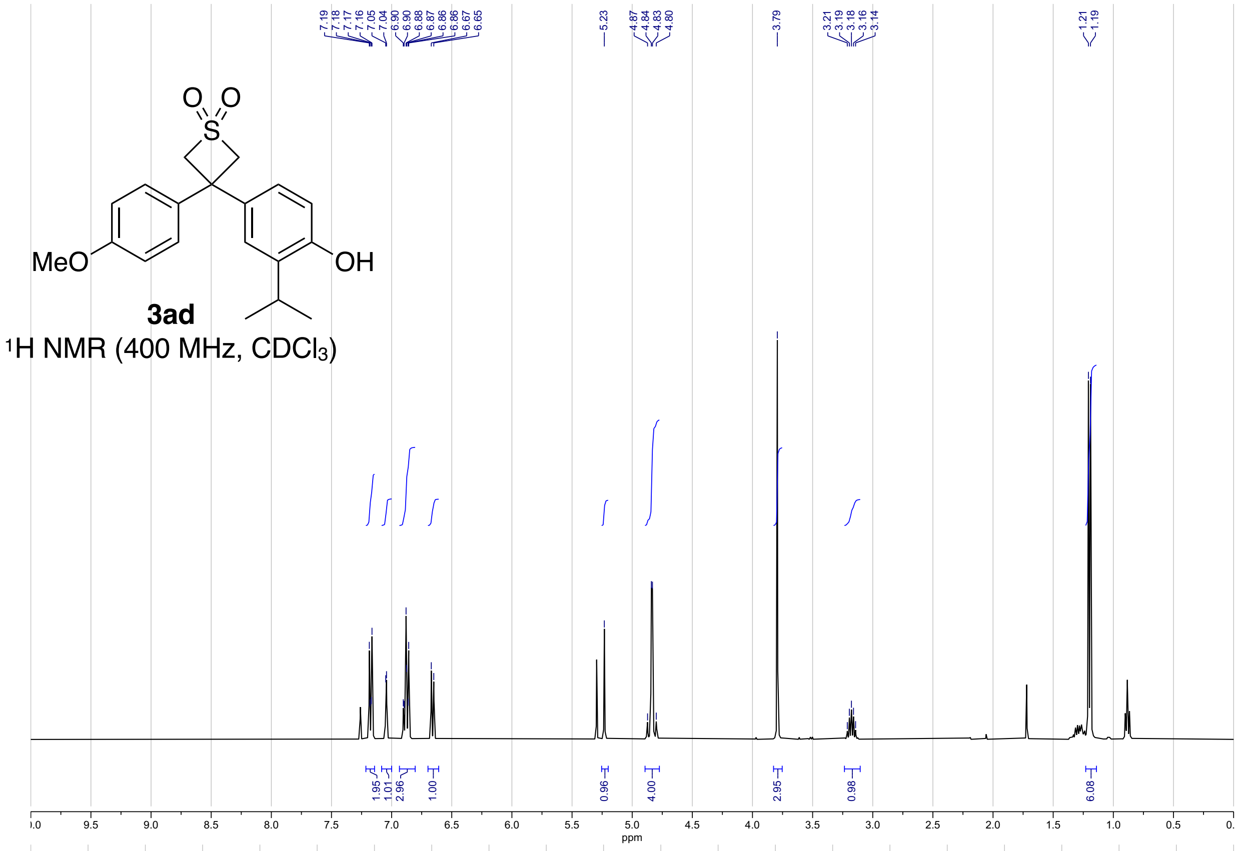
MeO
OH
3ad
¹H NMR (400 MHz, CDCl₃)
7.19 7.18 7.17 7.16 7.05 7.04 6.90 6.90 6.88 6.87 6.86 6.86 6.67 6.65
5.23
4.87 4.84 4.83 4.80
3.79
3.21 3.19 3.18 3.16 3.14
1.21 1.19
1.95 1.01 2.96 1.00 0.96 4.00 2.95 0.98 6.08
ppm

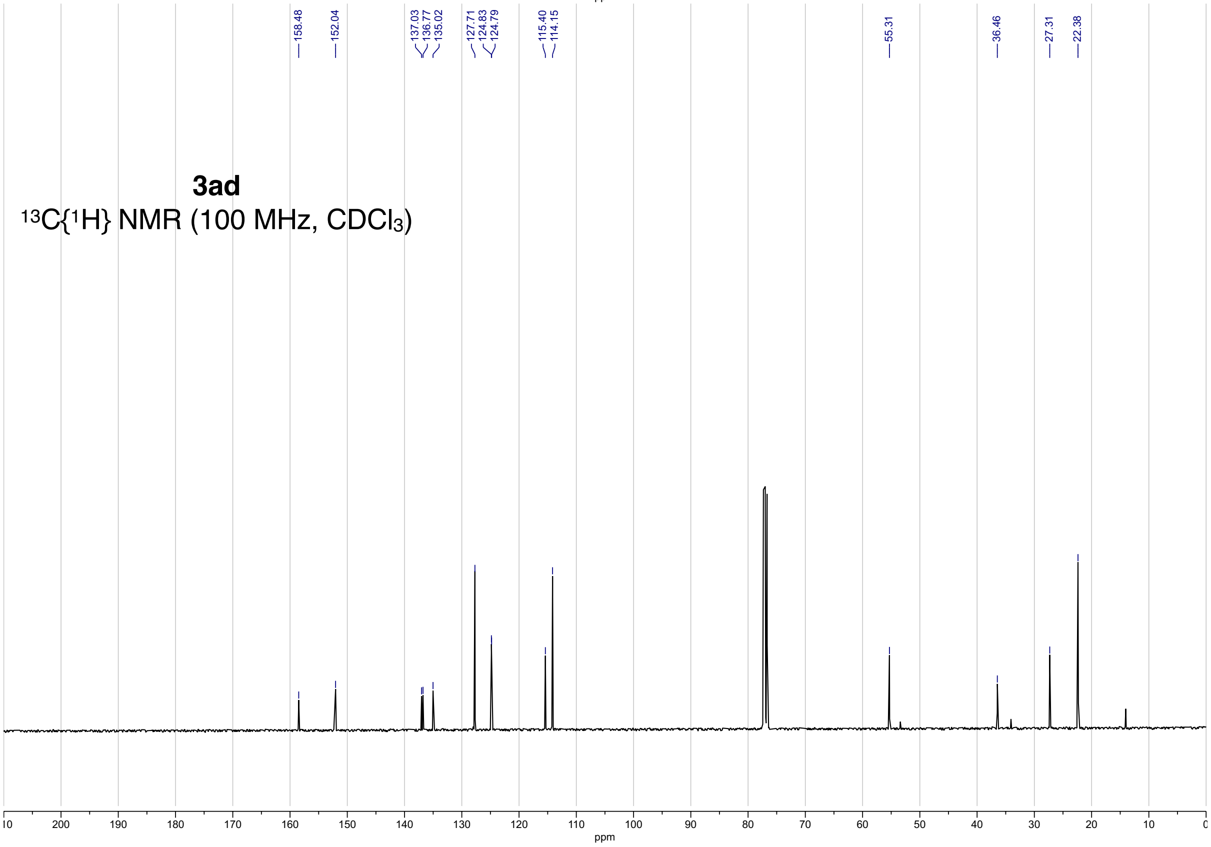
158.48
152.04
137.03 136.77 135.02
127.71 124.83 124.79
115.40 114.15
55.31
36.46
27.31
22.38
3ad
¹³C{¹H} NMR (100 MHz, CDCl₃)
ppm

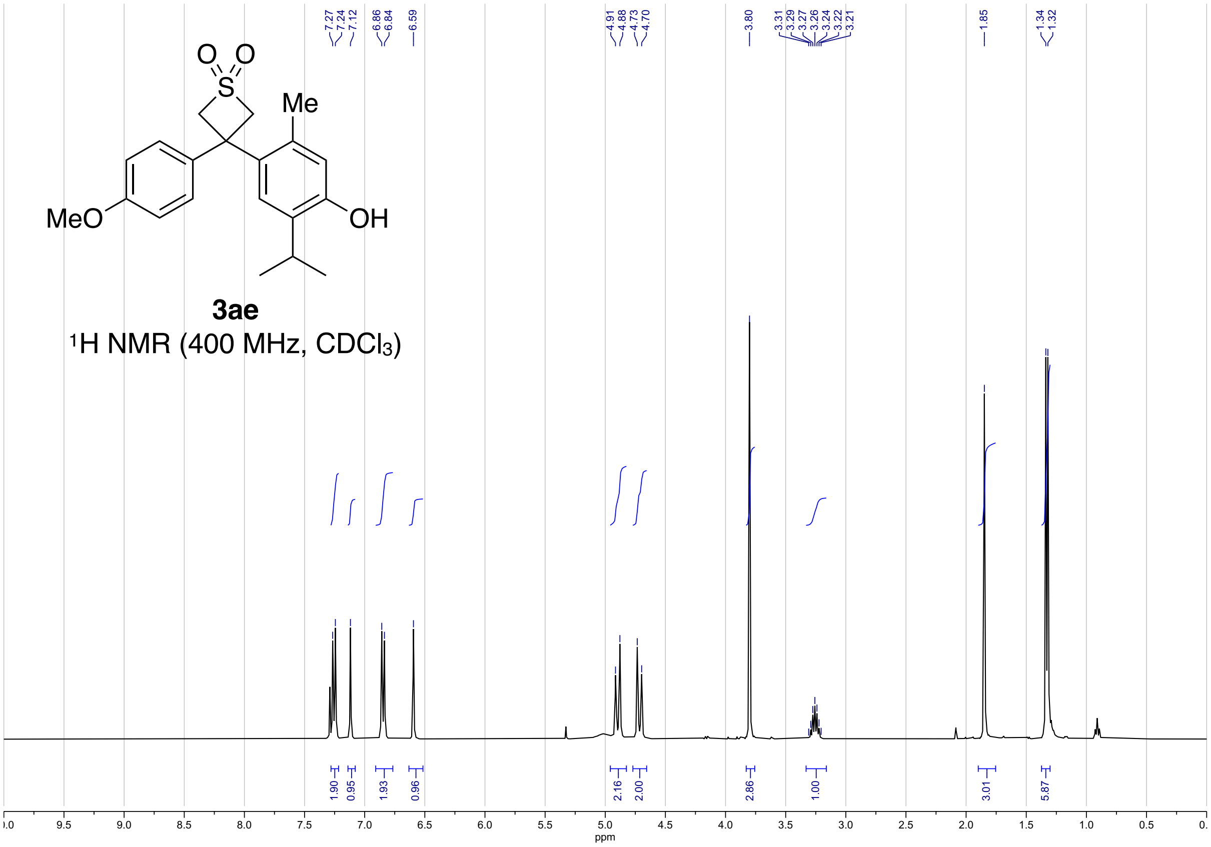

3ae

^{1}H NMR (400 MHz, $CDCl_3$)

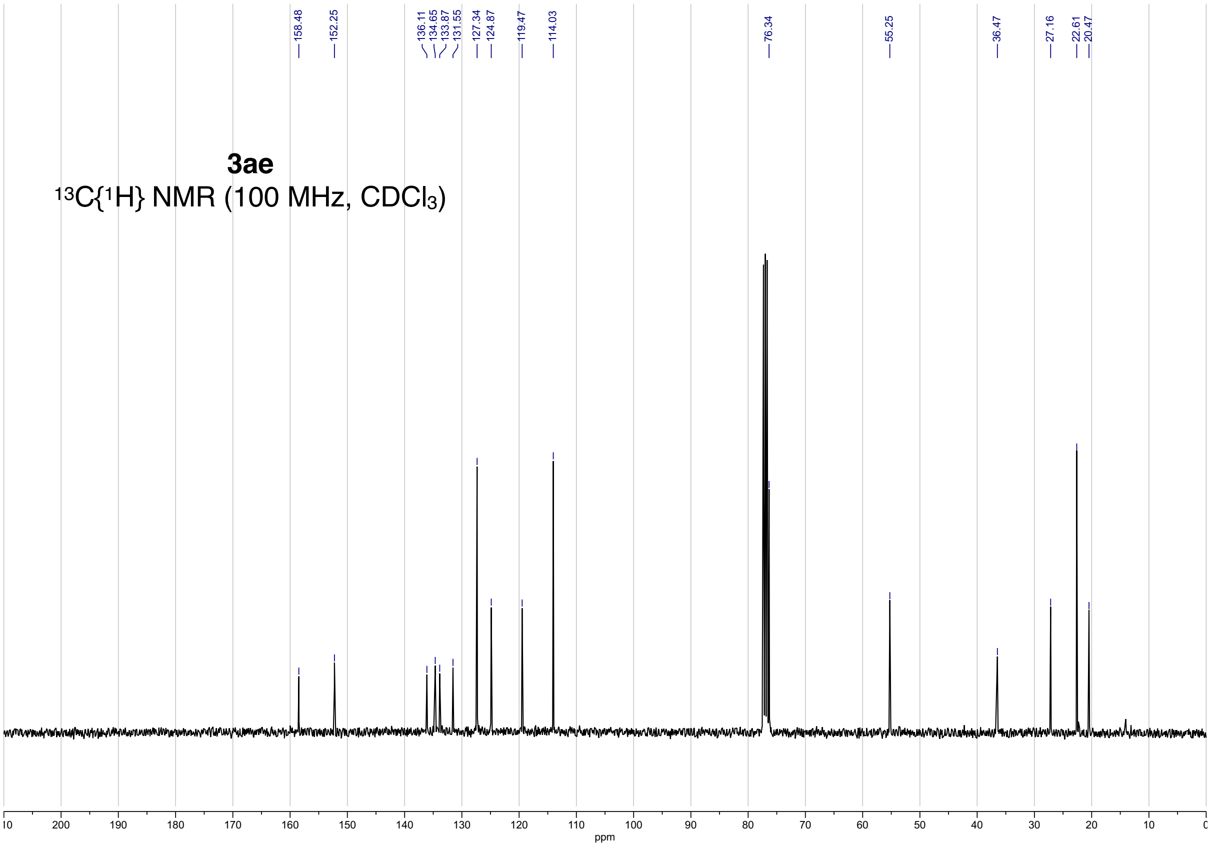

3ae

$^{13}C\{^{1}H\}$ NMR (100 MHz, $CDCl_3$)

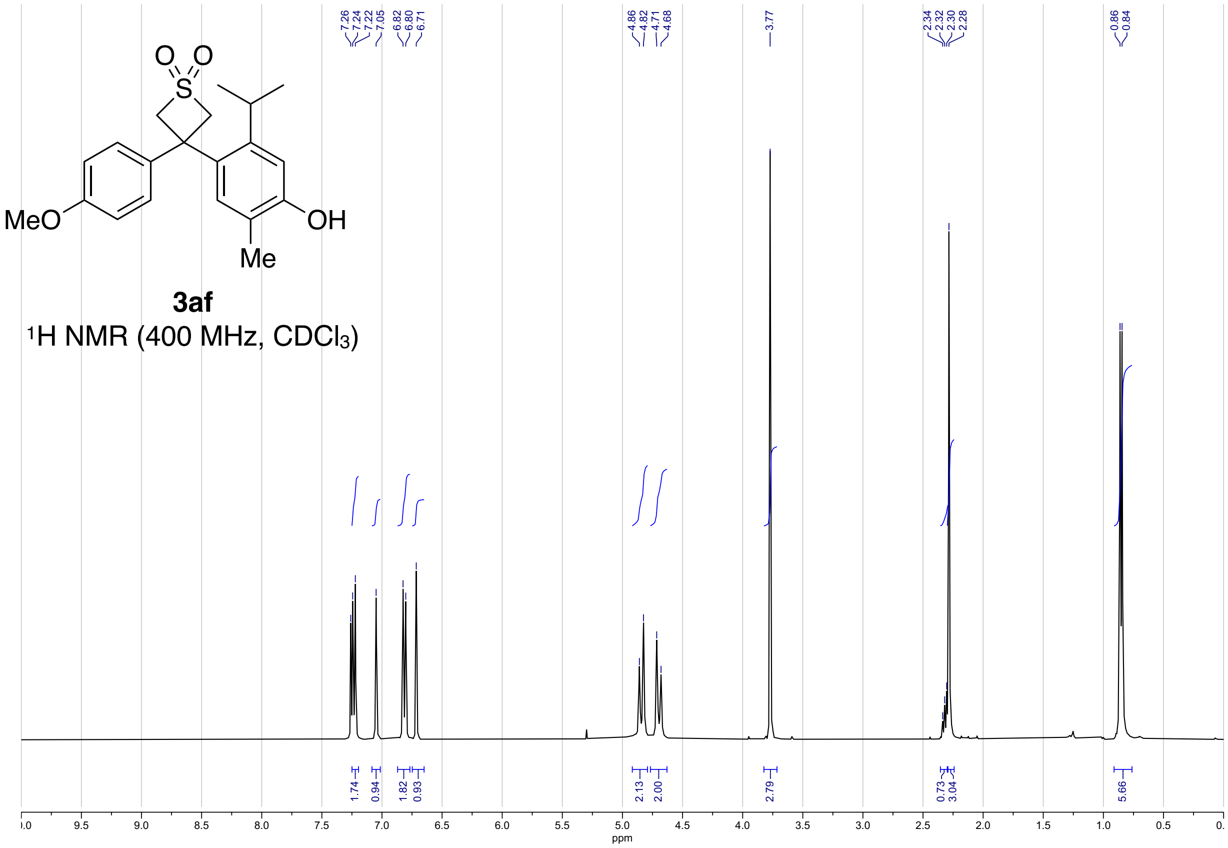

3af
^{1}H NMR (400 MHz, $CDCl_3$)

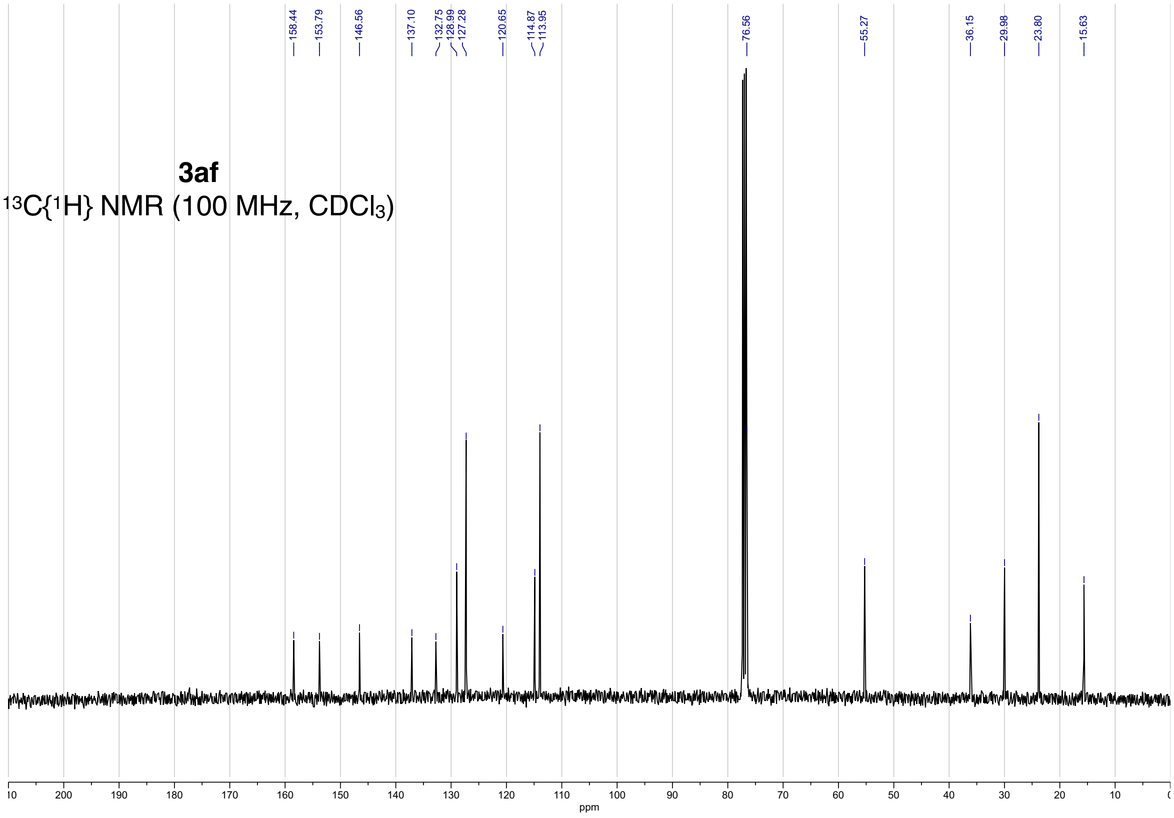

3af
^{13}C{^{1}H} NMR (100 MHz, $CDCl_3$)

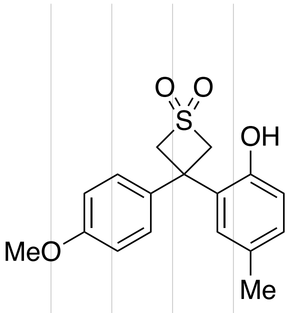

3ag
^{1}HNMR (400 MHz, DMSO-d_6)

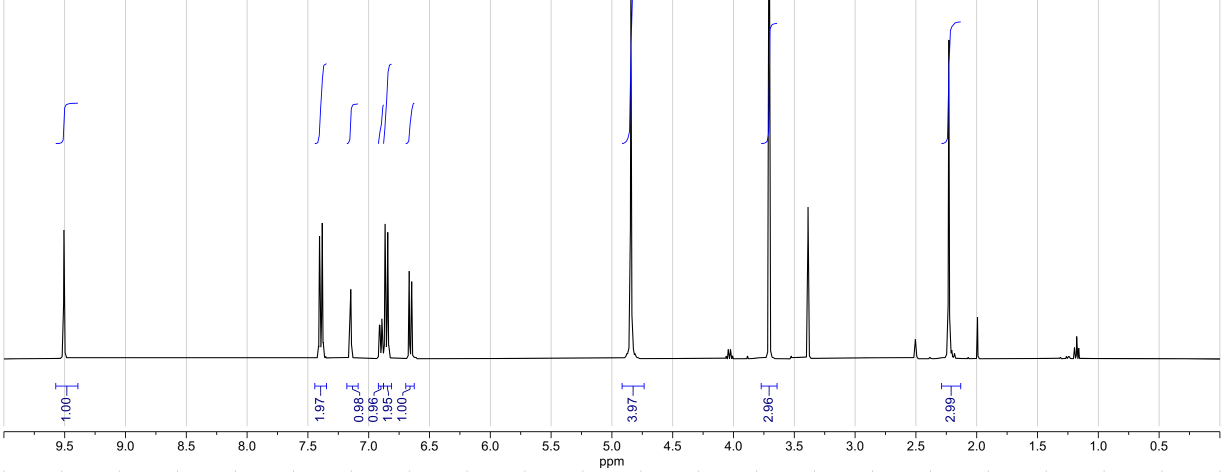

3ag
^{13}C{^{1}H} NMR (100 MHz, DMSO-d_6)

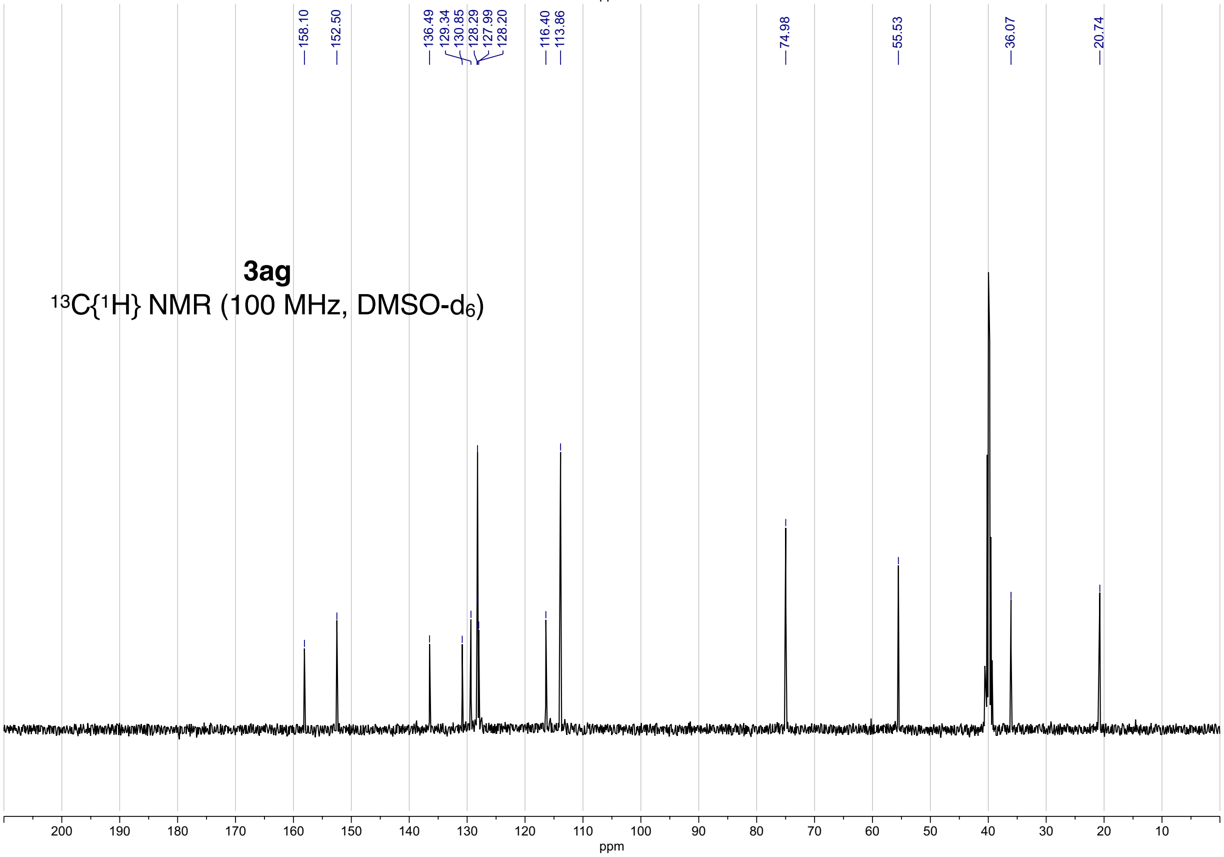

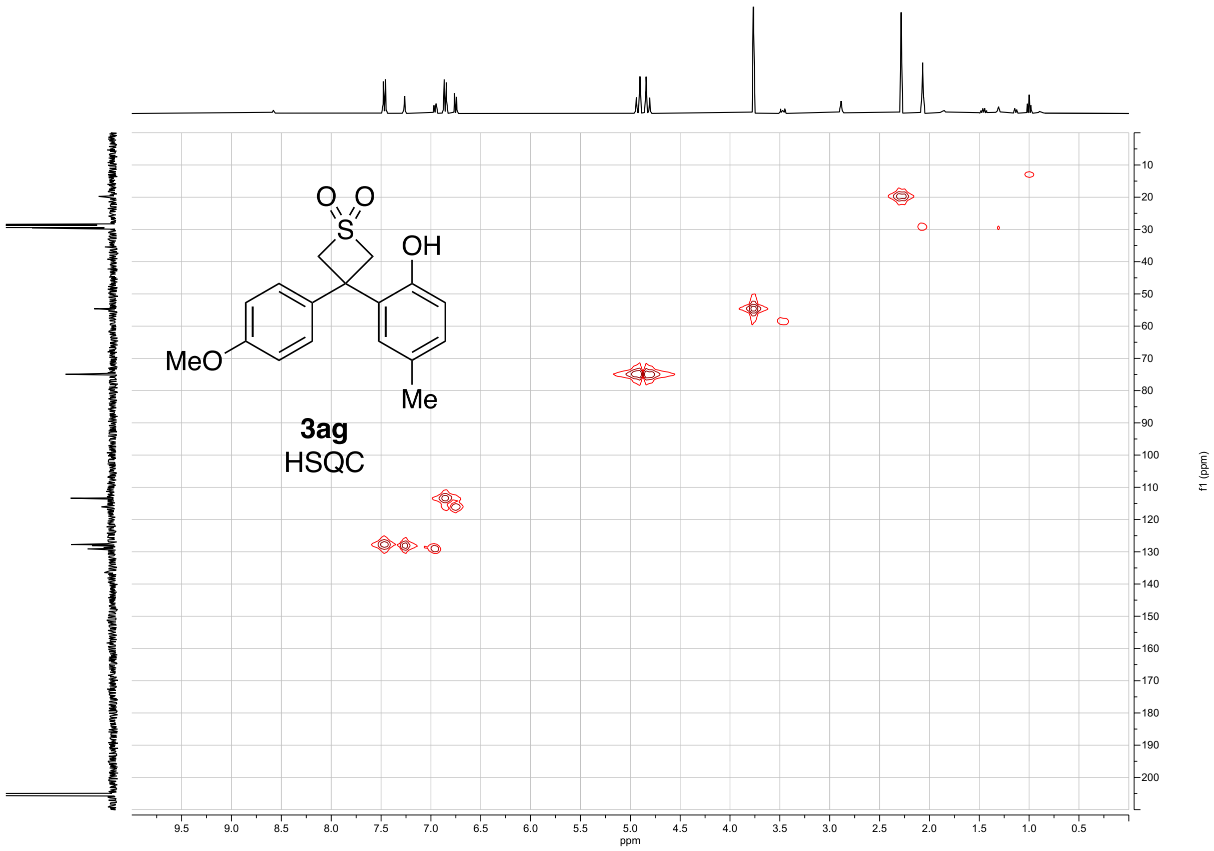
O
O
S
OH
MeO
Me
3ag
HSQC
ppm
f1 (ppm)

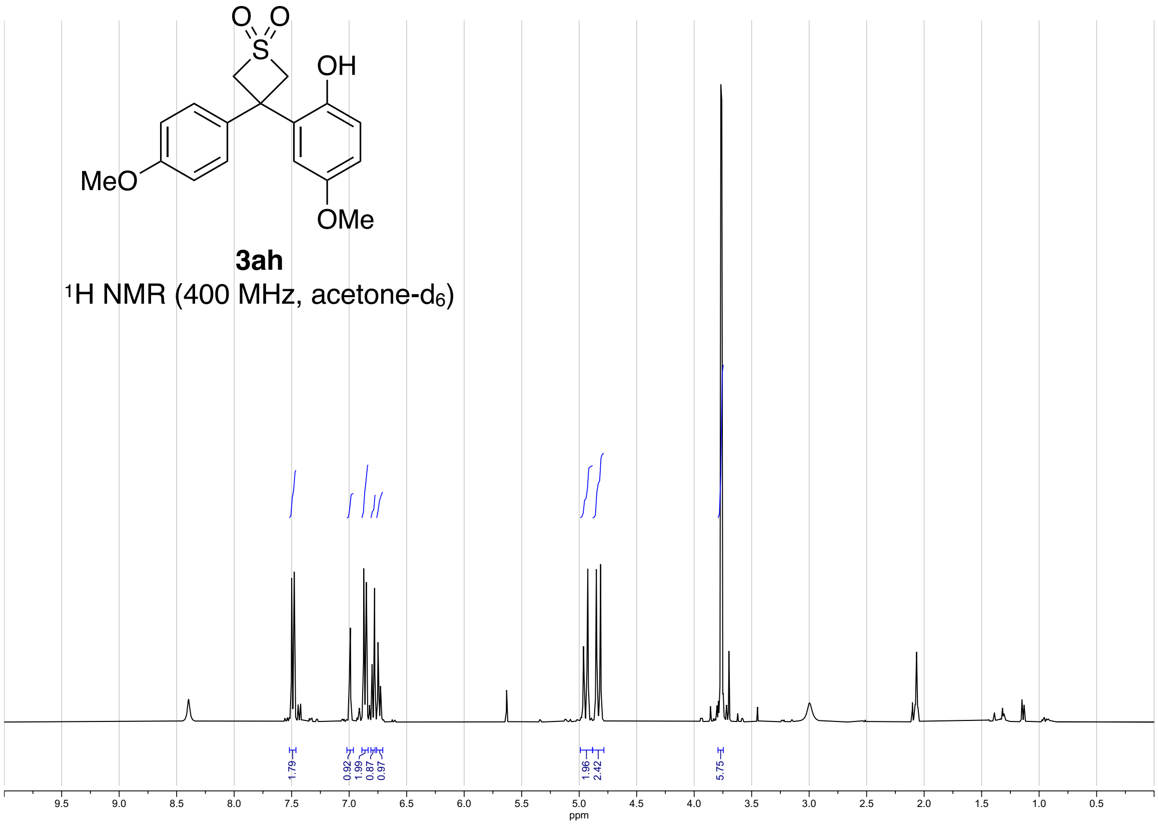

3ah
^{1}H NMR (400 MHz, acetone-d_6)

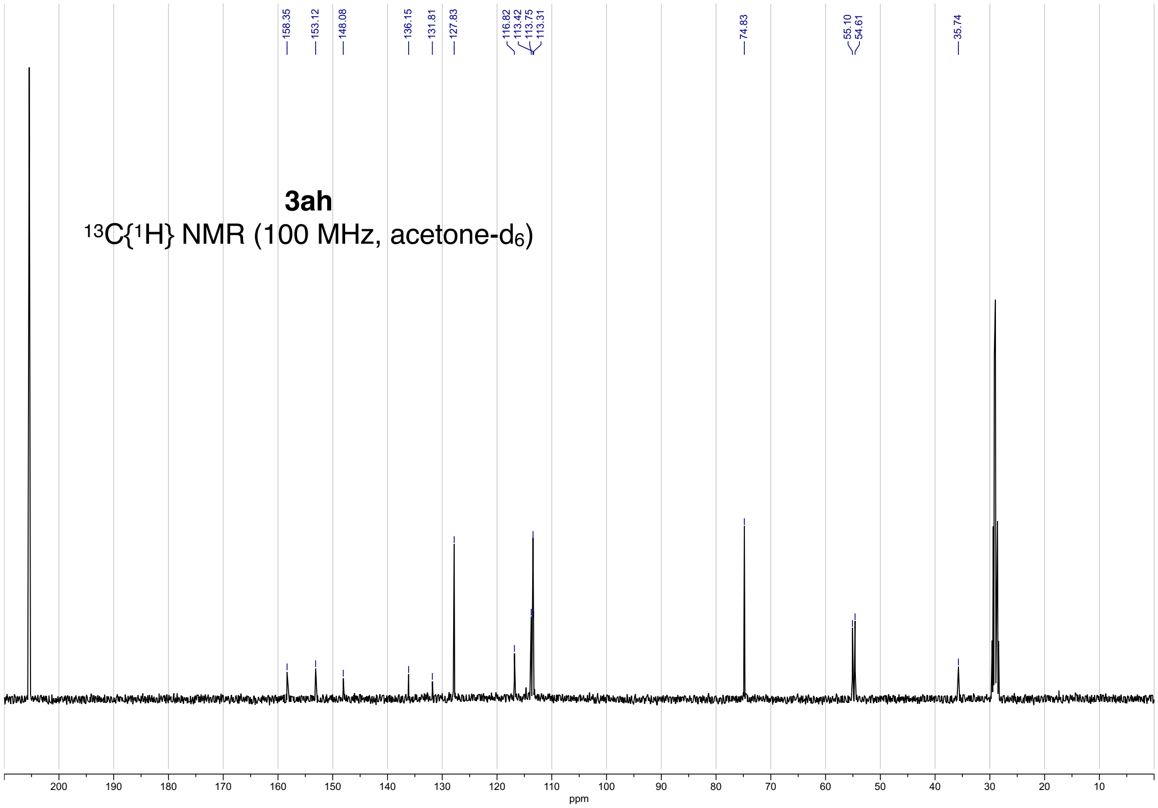

3ah
^{13}C{^{1}H} NMR (100 MHz, acetone-d_6)

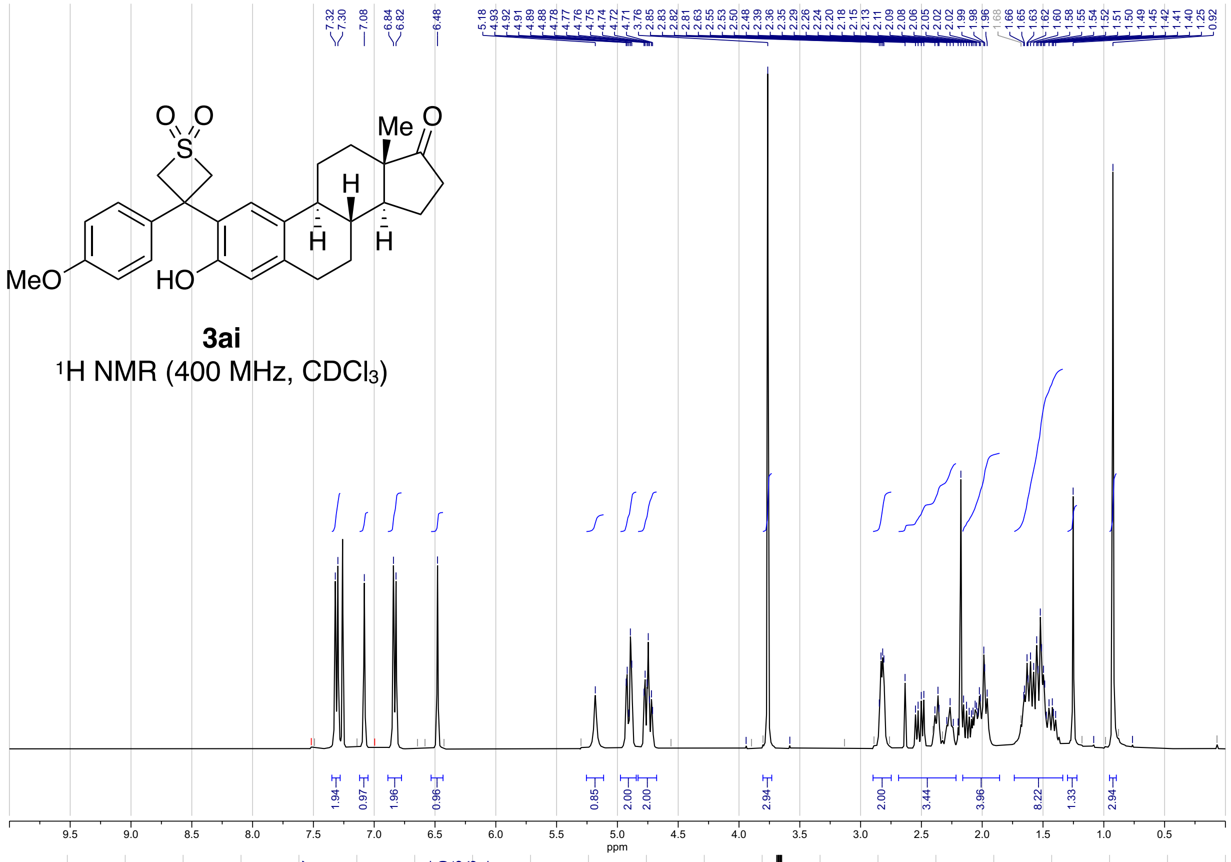

3ai
1H NMR (400 MHz, CDCl3)
MeO
HO
Me
ppm

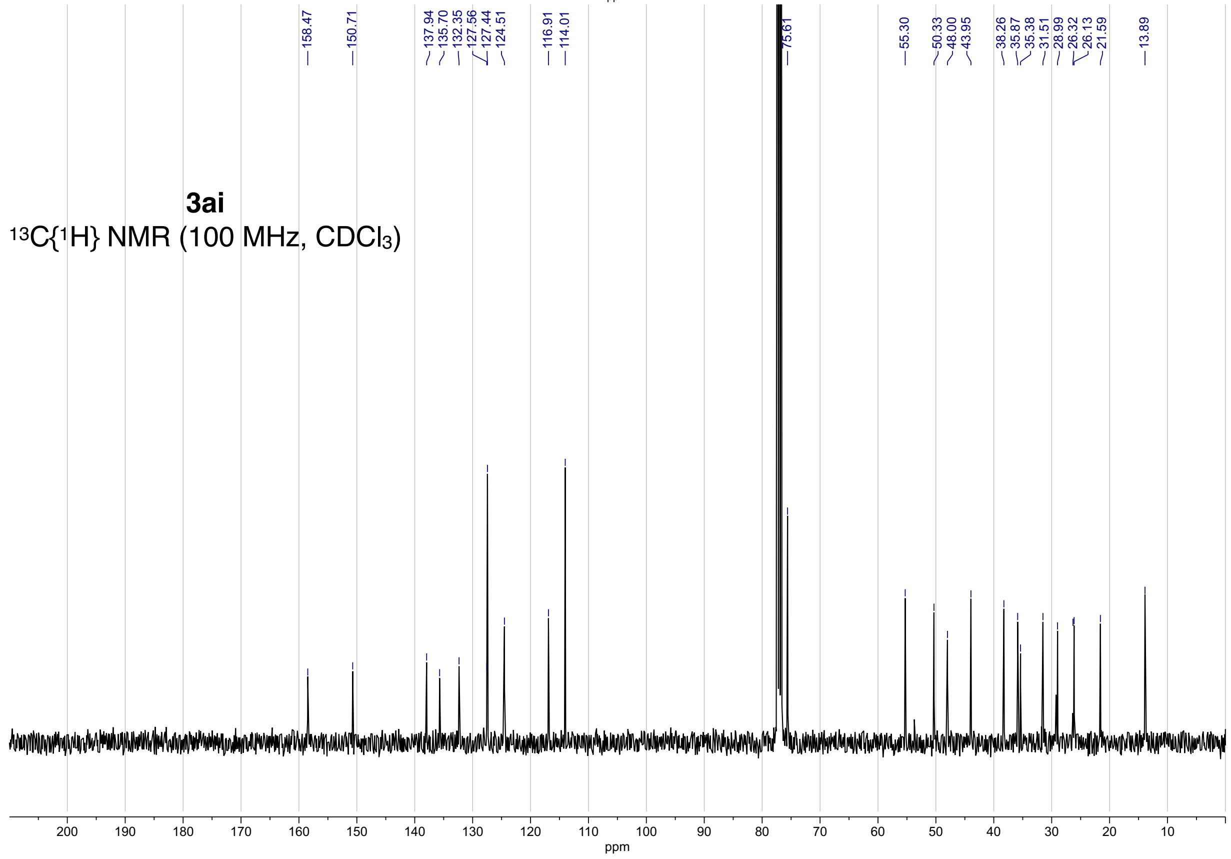

3ai
13C{1H} NMR (100 MHz, CDCl3)
ppm

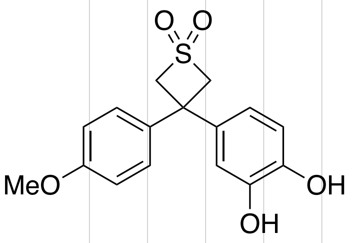

3aj

^{1}H NMR (400 MHz, DMSO-d_6)

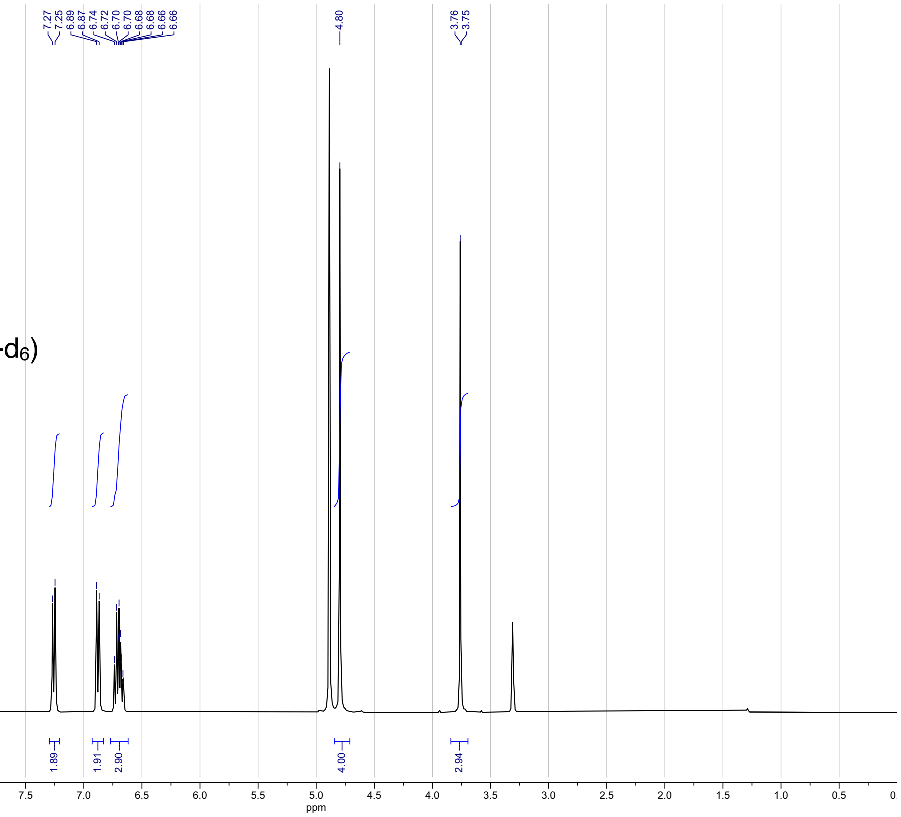

3aj

^{13}C{^{1}H} NMR (100 MHz, DMSO-d_6)

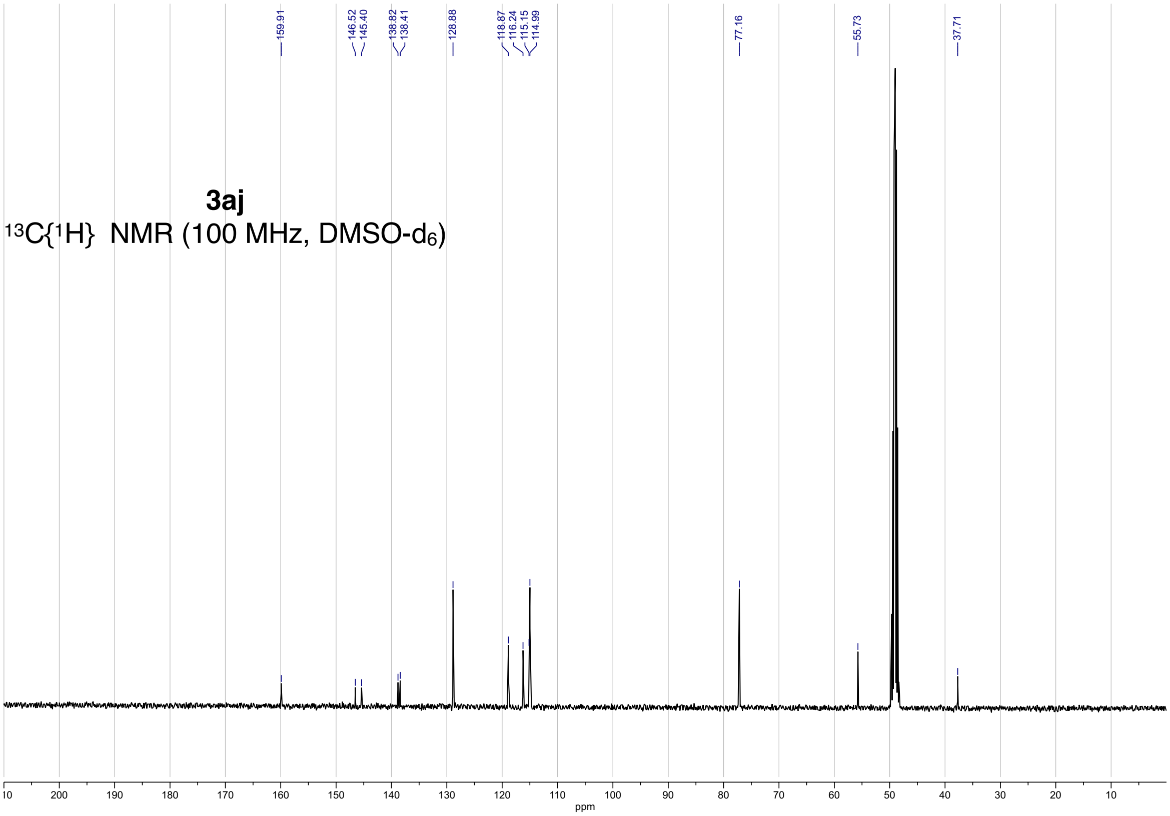

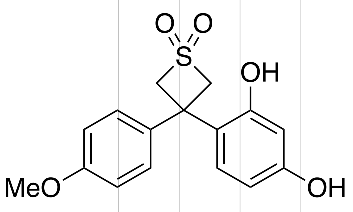

3ak

^{1}H NMR (400 MHz, acetone-d_6)

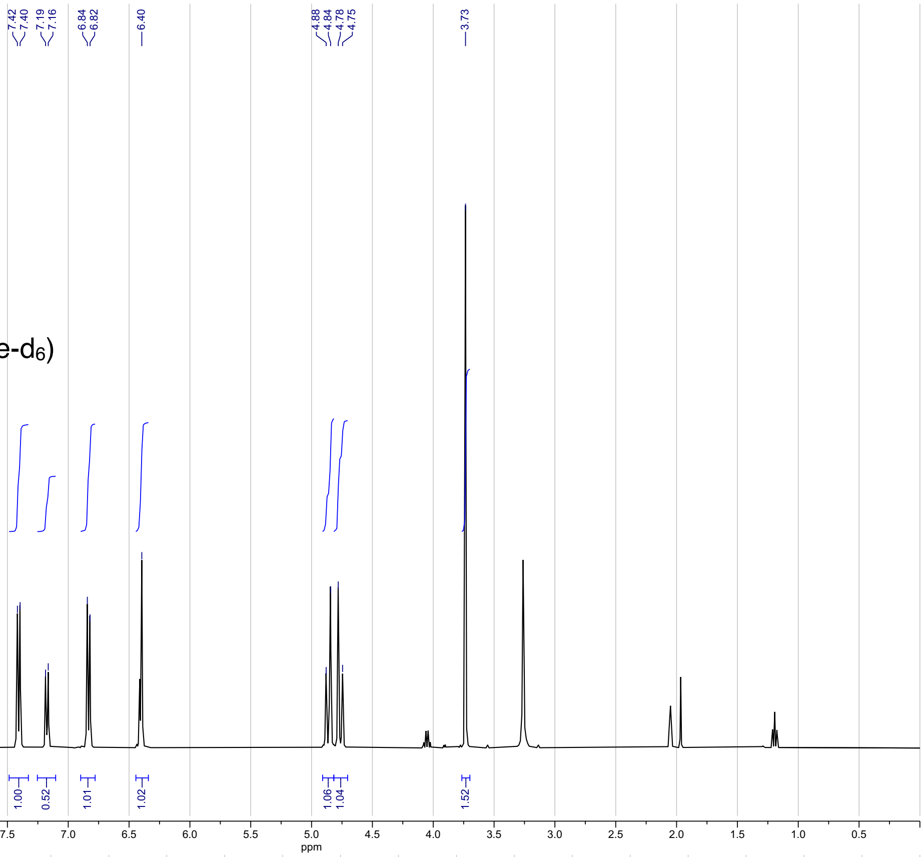

3ak

$^{13}C\{^{1}H\}$ NMR (400 MHz, acetone-[illegible]

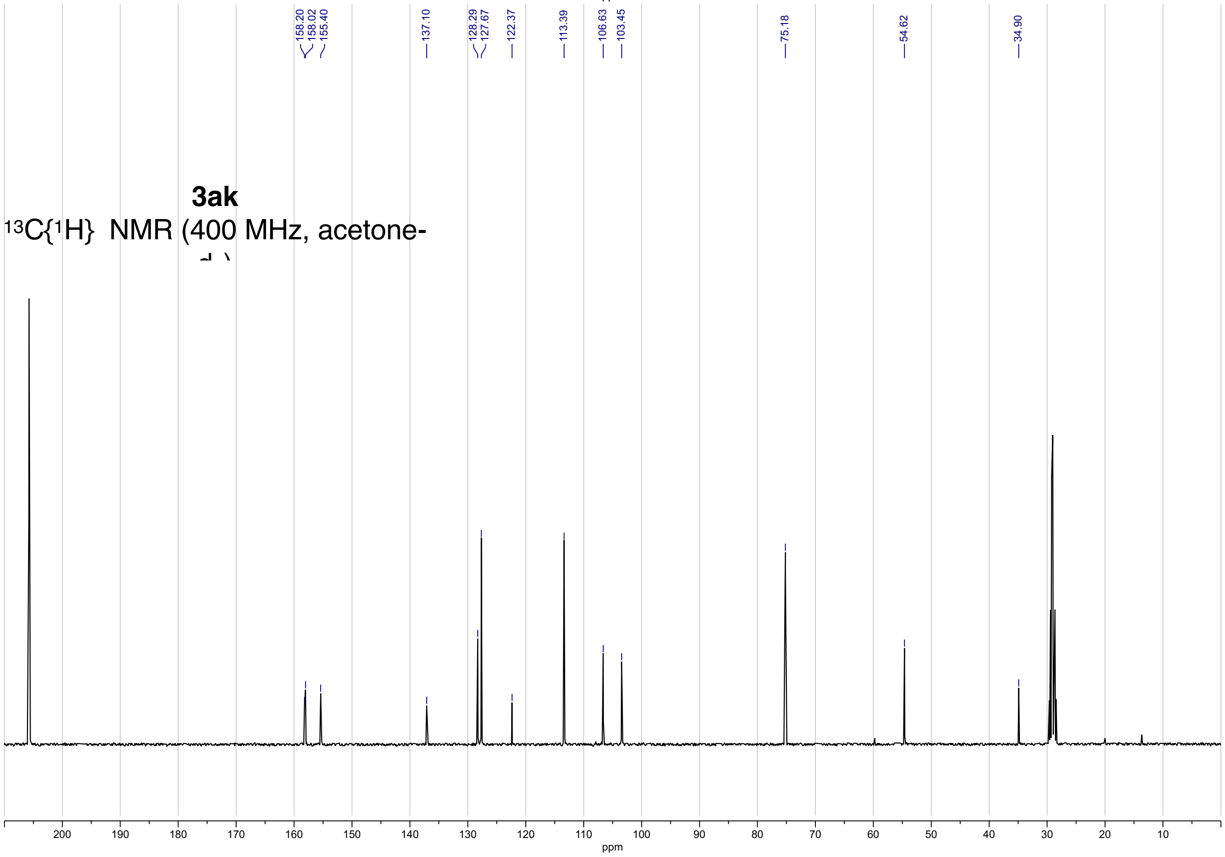

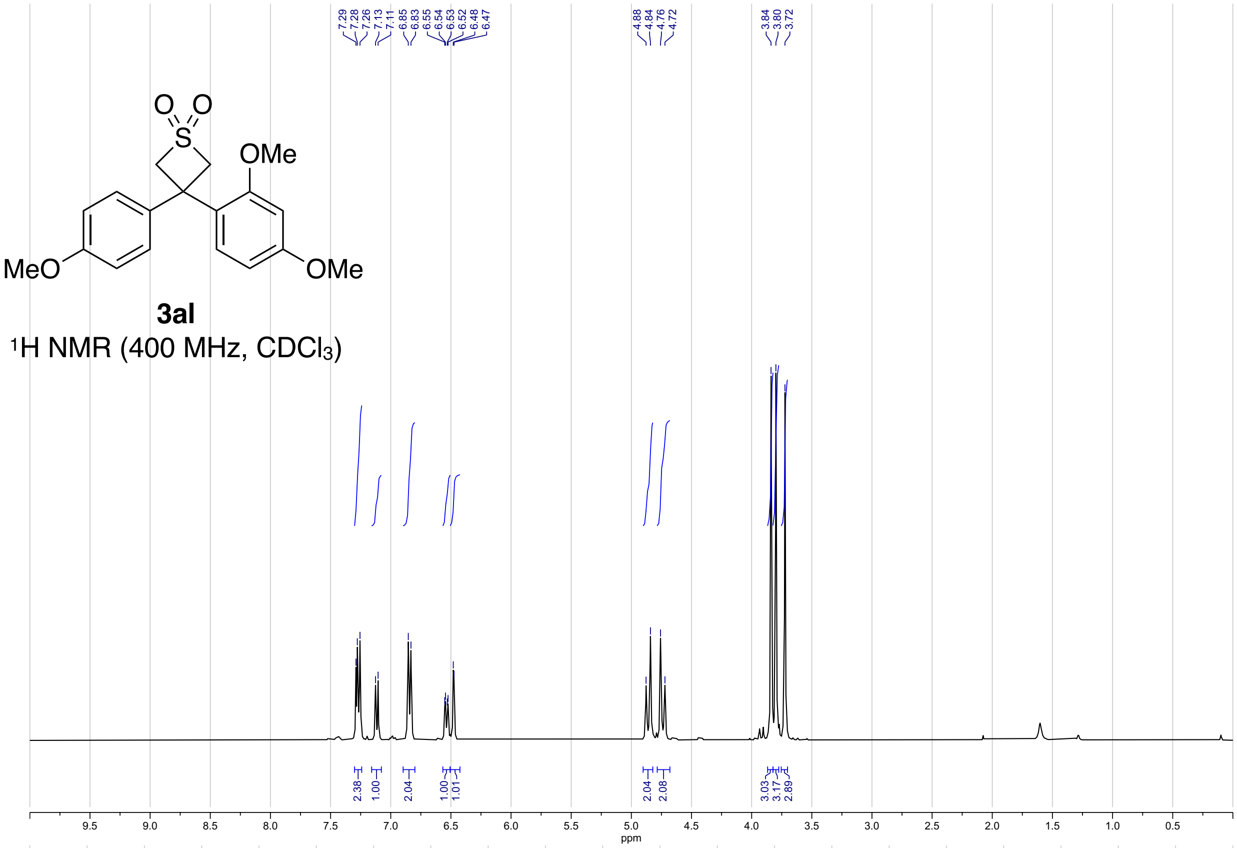

O O
S
OMe
MeO
OMe
3al
1H NMR (400 MHz, CDCl3)
7.29 7.28 7.26 7.13 7.11 6.85 6.83 6.55 6.54 6.53 6.52 6.48 6.47
4.88 4.84 4.76 4.72
3.84 3.80 3.72
2.38 1.00 2.04 1.00 1.01 2.04 2.08 3.03 3.17 2.89
ppm

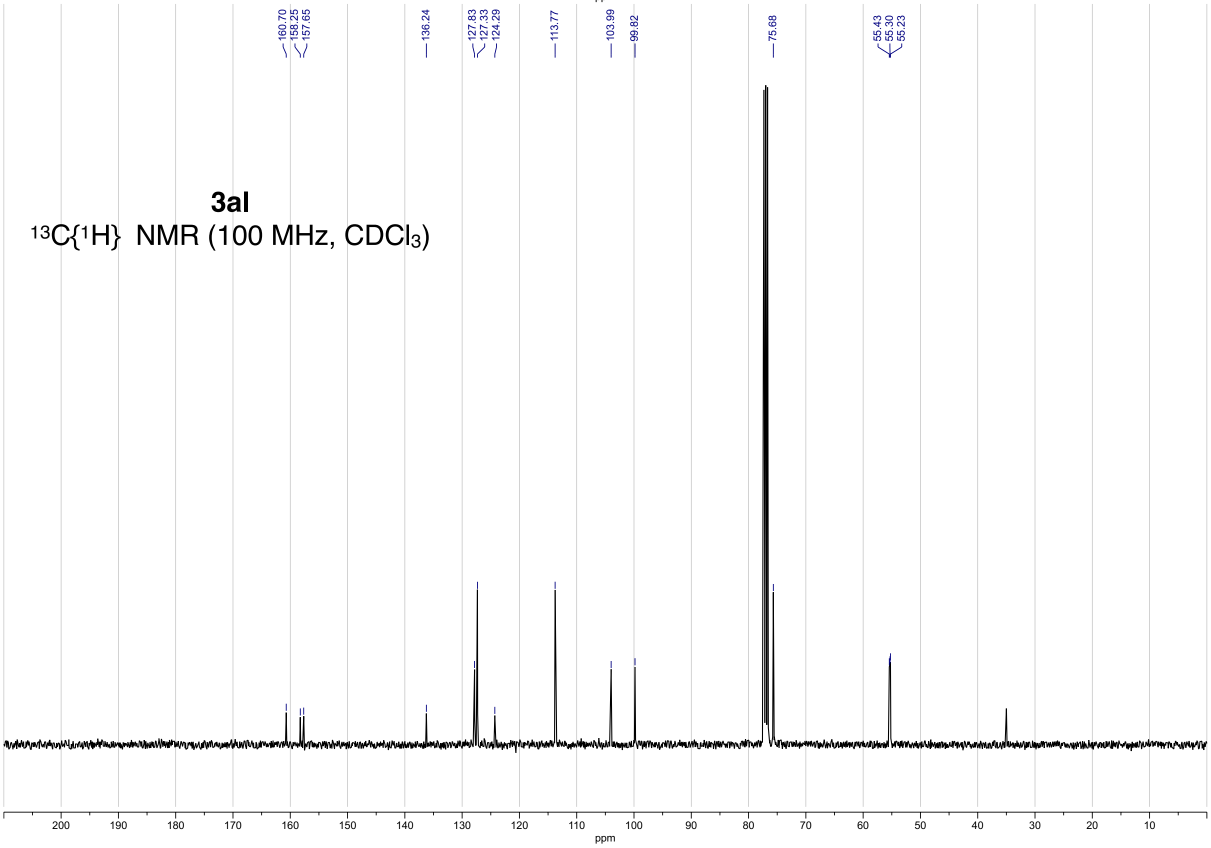

3al
13C{1H} NMR (100 MHz, CDCl3)
160.70 158.25 157.65 136.24 127.83 127.33 124.29 113.77 103.99 99.82 75.68 55.43 55.30 55.23
ppm

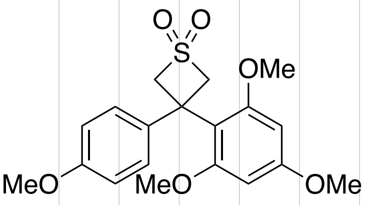

3am

^{1}H NMR (400 MHz, $CDCl_3$)

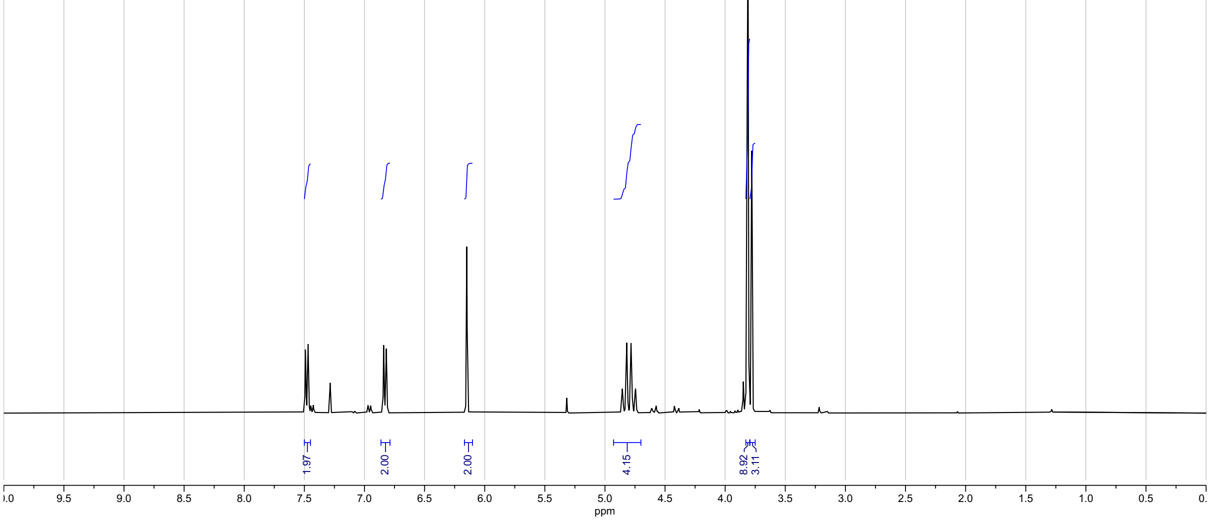

3am

$^{13}C\{^{1}H\}$ NMR (100 MHz, $CDCl_3$)

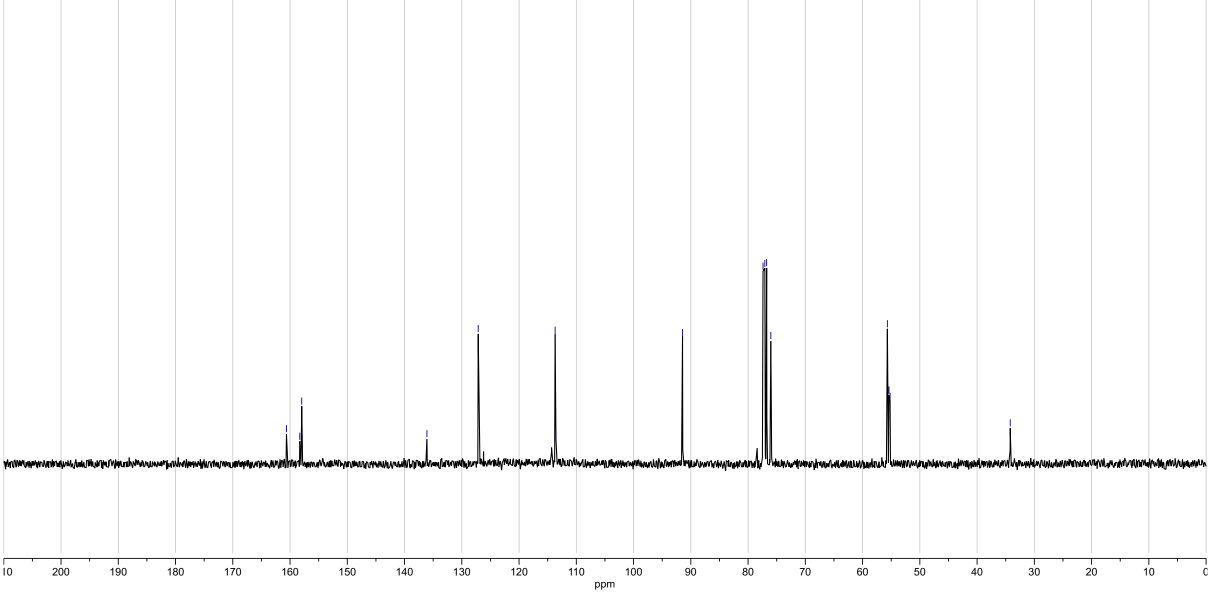

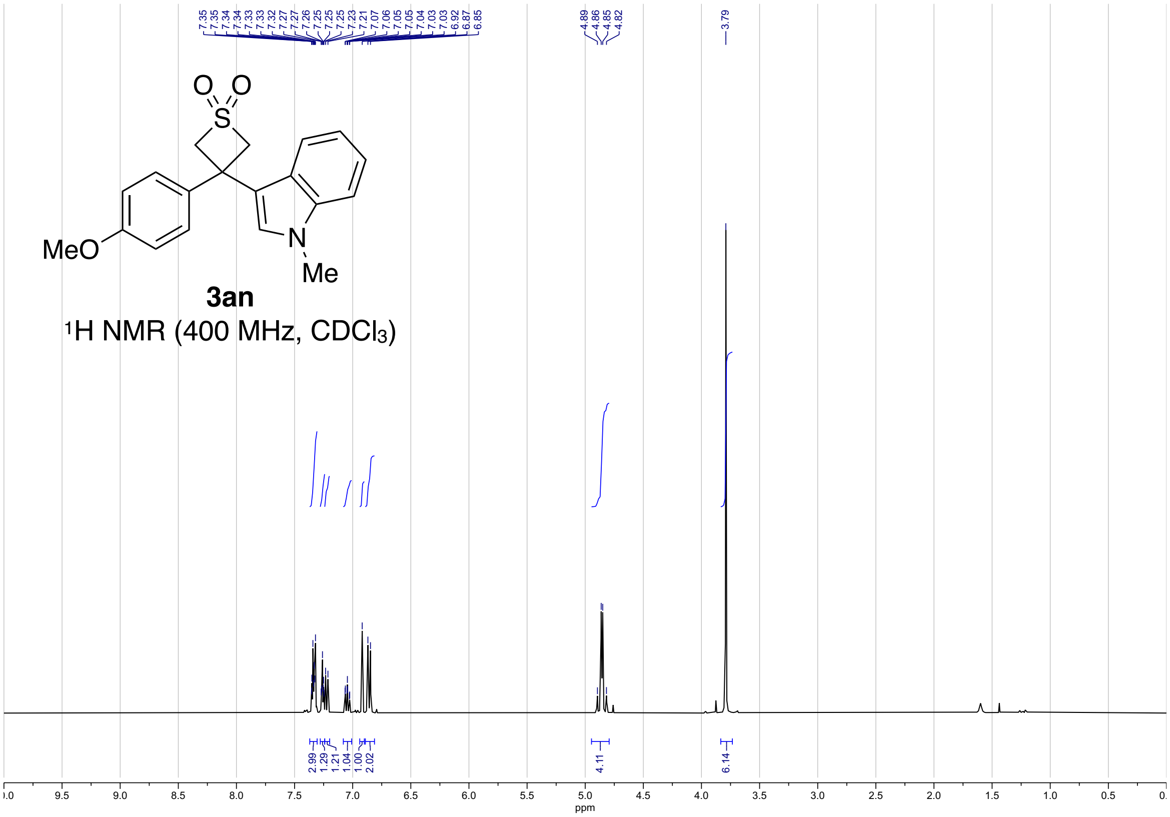
MeO
N
Me
O
S
O
3an
1H NMR (400 MHz, CDCl3)
ppm

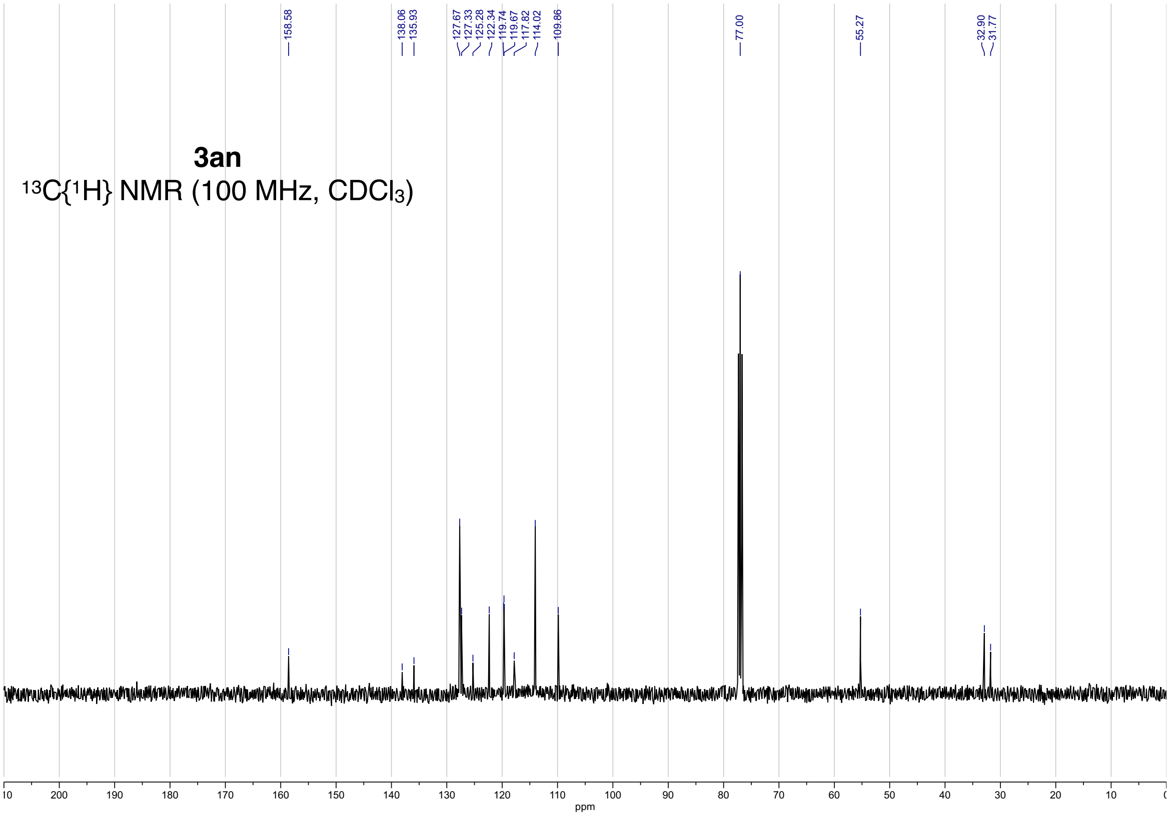
3an
13C{1H} NMR (100 MHz, CDCl3)
ppm

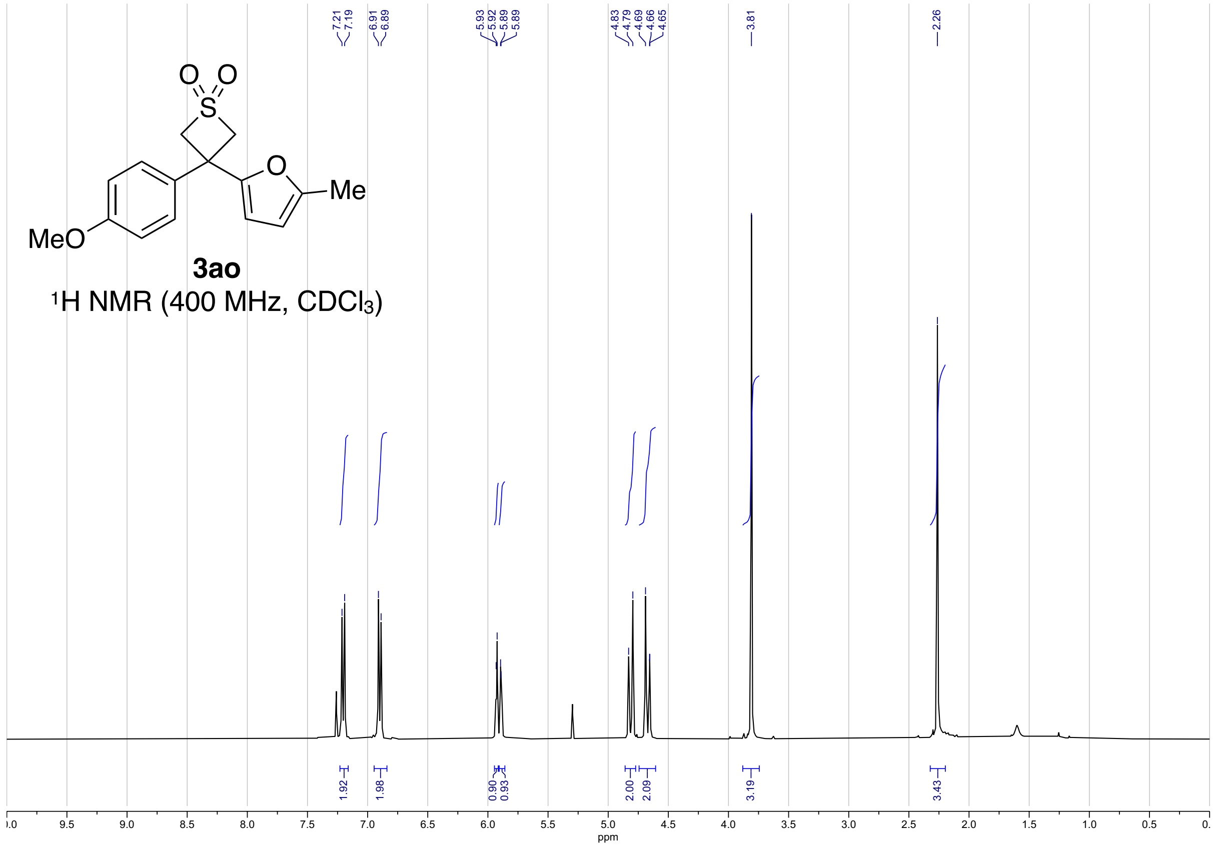
7.21
7.19
6.91
6.89
5.93
5.92
5.89
5.89
4.83
4.79
4.69
4.66
4.65
3.81
2.26
O O
S
O
Me
MeO
3ao
1H NMR (400 MHz, CDCl3)
1.92
1.98
0.90
0.93
2.00
2.09
3.19
3.43
ppm

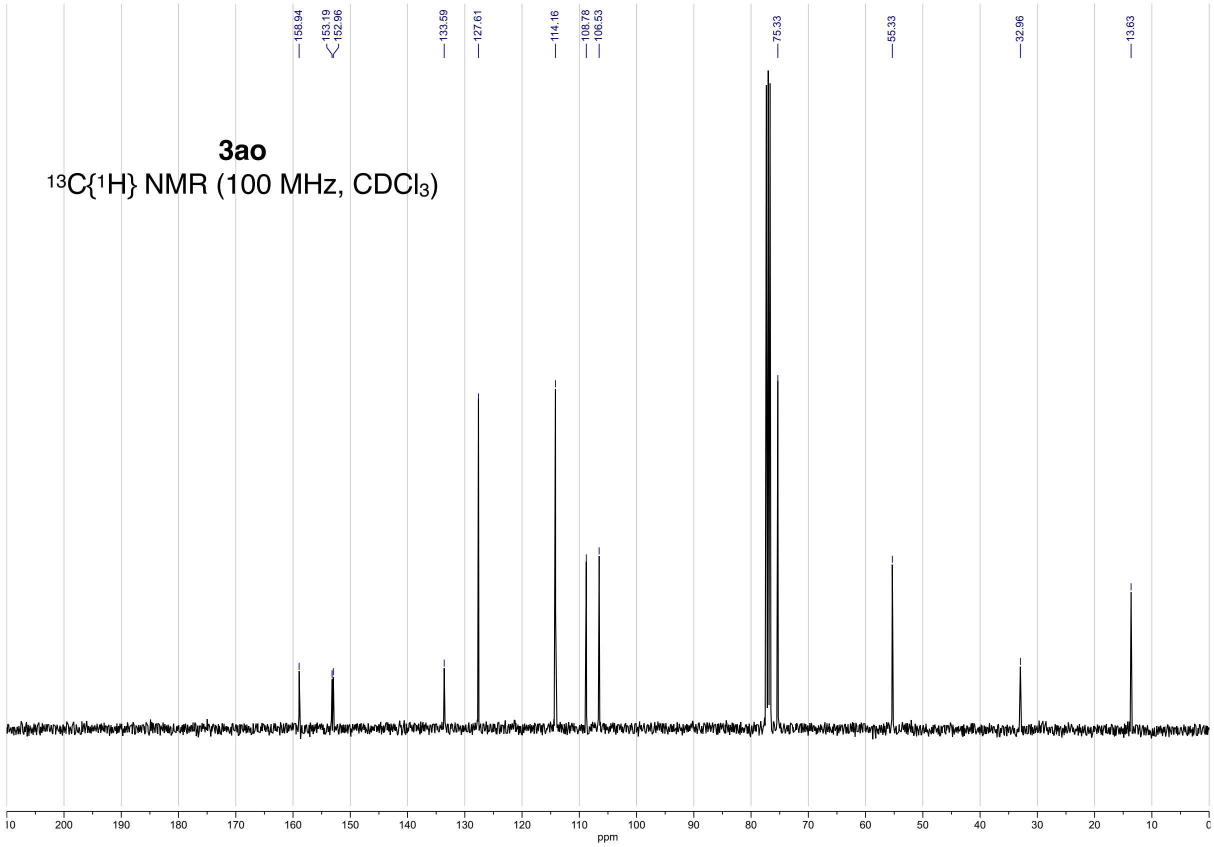
158.94
153.19
152.96
133.59
127.61
114.16
108.78
106.53
75.33
55.33
32.96
13.63
3ao
13C{1H} NMR (100 MHz, CDCl3)
ppm

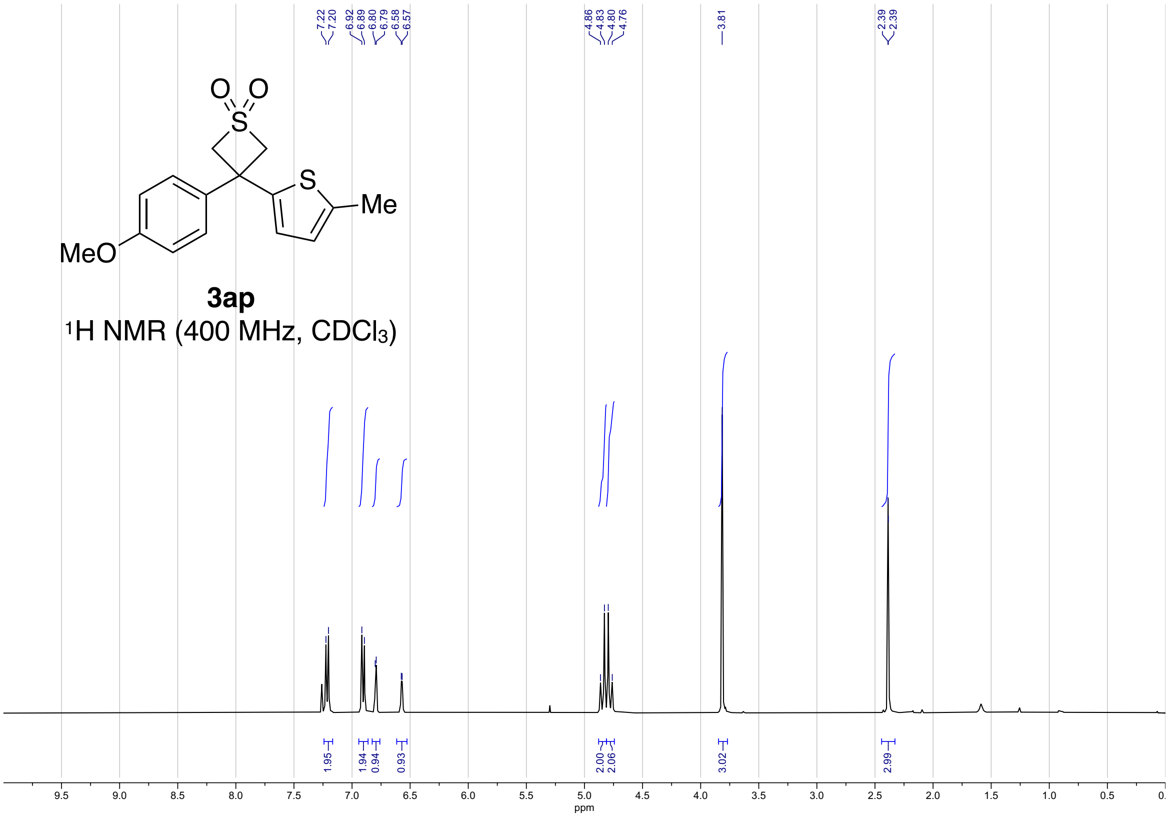

7.22
7.20
6.92
6.89
6.80
6.79
6.58
6.57
4.86
4.83
4.80
4.76
3.81
2.39
2.39
O
O
S
S
Me
MeO
3ap
¹H NMR (400 MHz, CDCl₃)
1.95
1.94
0.94
0.93
2.00
2.06
3.02
2.99
ppm

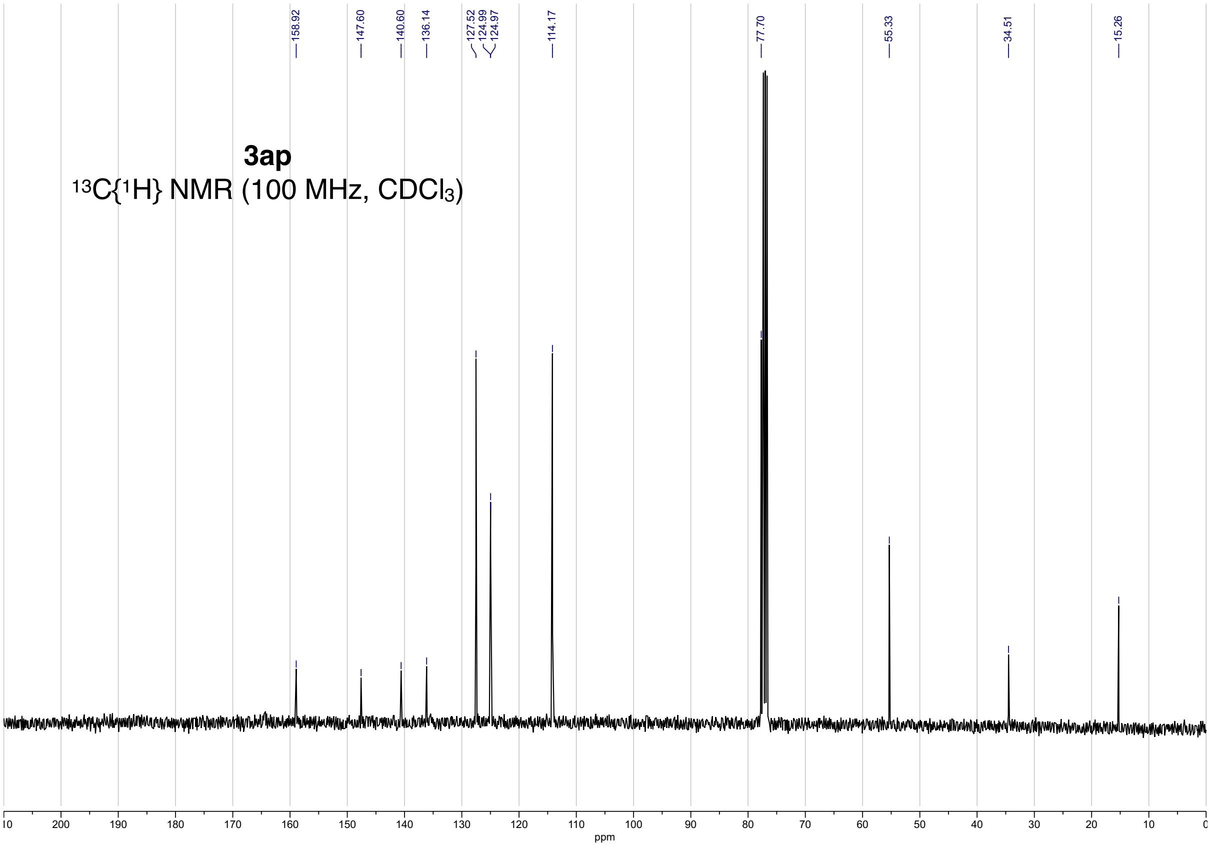

158.92
147.60
140.60
136.14
127.52
124.99
124.97
114.17
77.70
55.33
34.51
15.26
3ap
¹³C{¹H} NMR (100 MHz, CDCl₃)
ppm

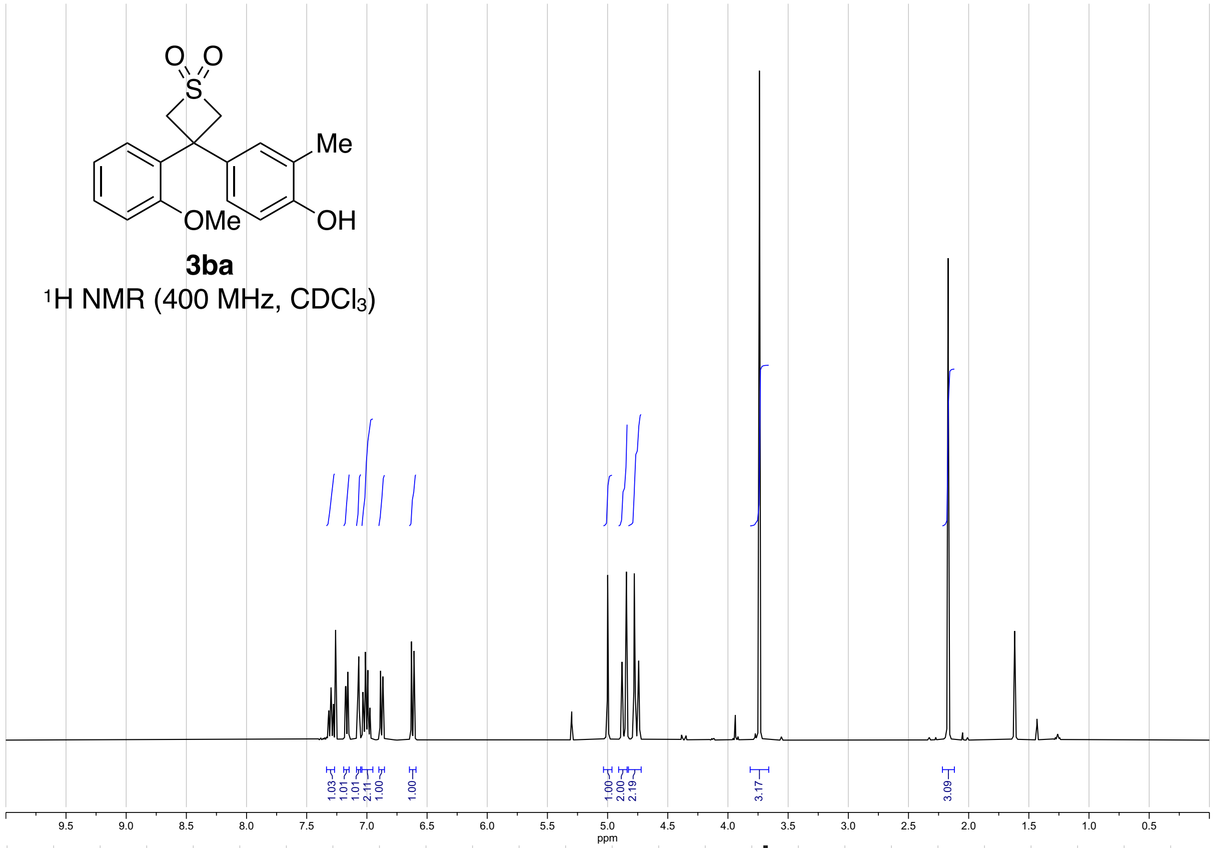
Me
OH
OMe
3ba
1H NMR (400 MHz, CDCl3)
1.03
1.01
1.01
2.11
1.00
1.00
1.00
2.00
2.19
3.17
3.09
ppm

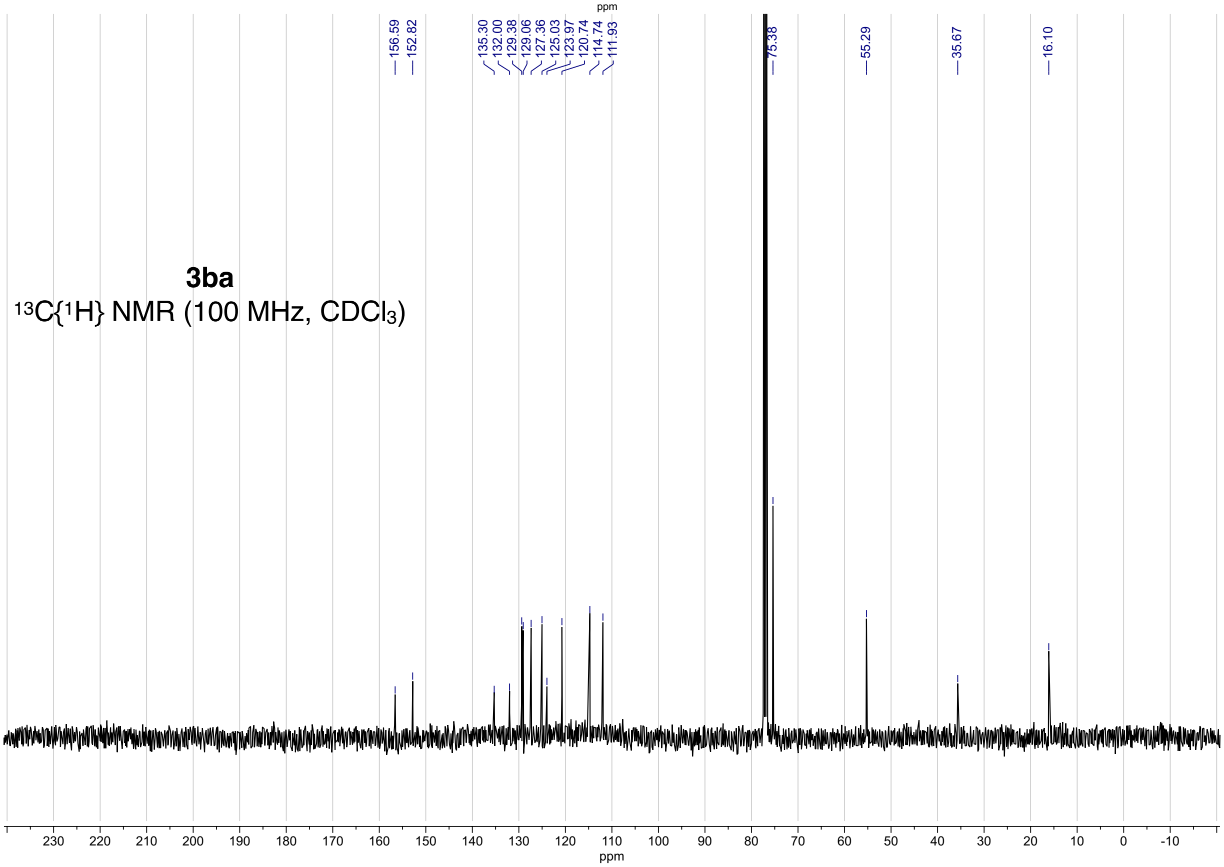
156.59
152.82
135.30
132.00
129.38
129.06
127.36
125.03
123.97
120.74
114.74
111.93
75.38
55.29
35.67
16.10
3ba
13C{1H} NMR (100 MHz, CDCl3)
ppm

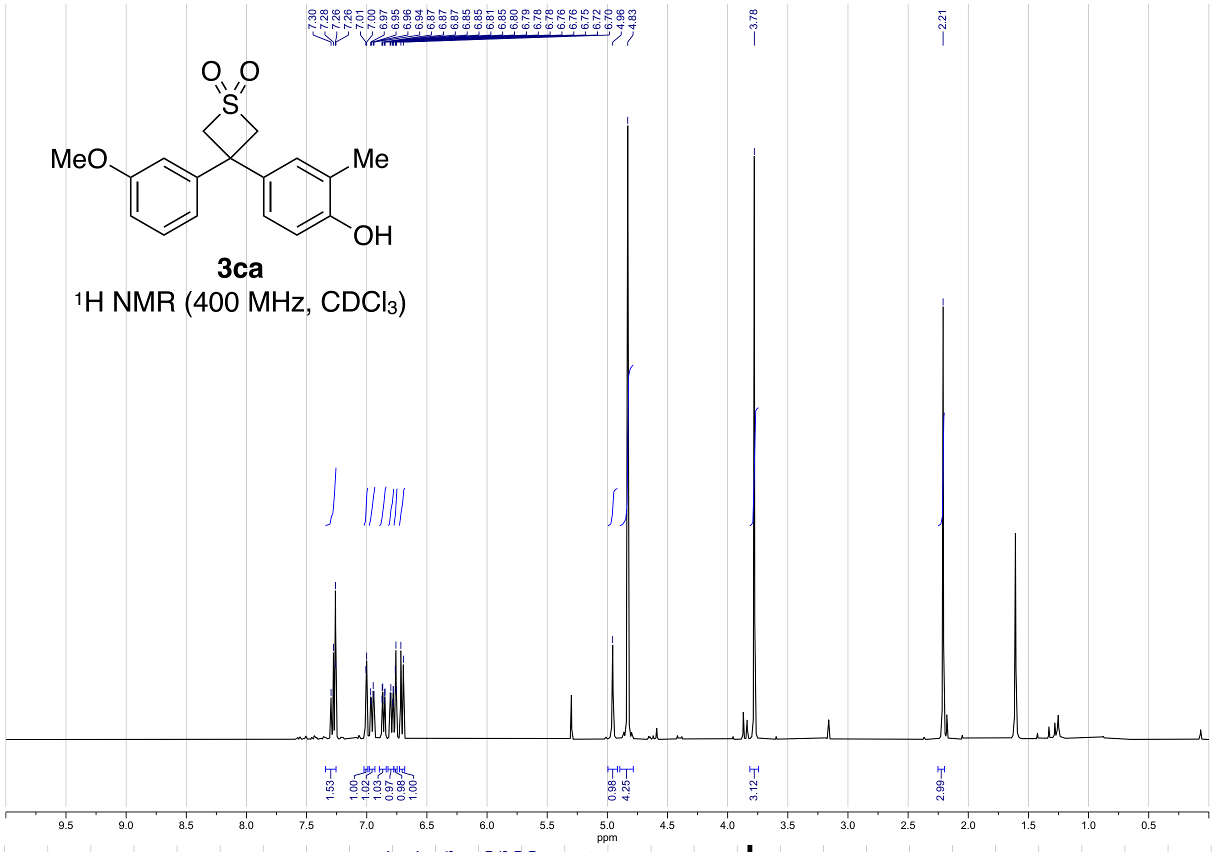
3ca
1H NMR (400 MHz, CDCl3)

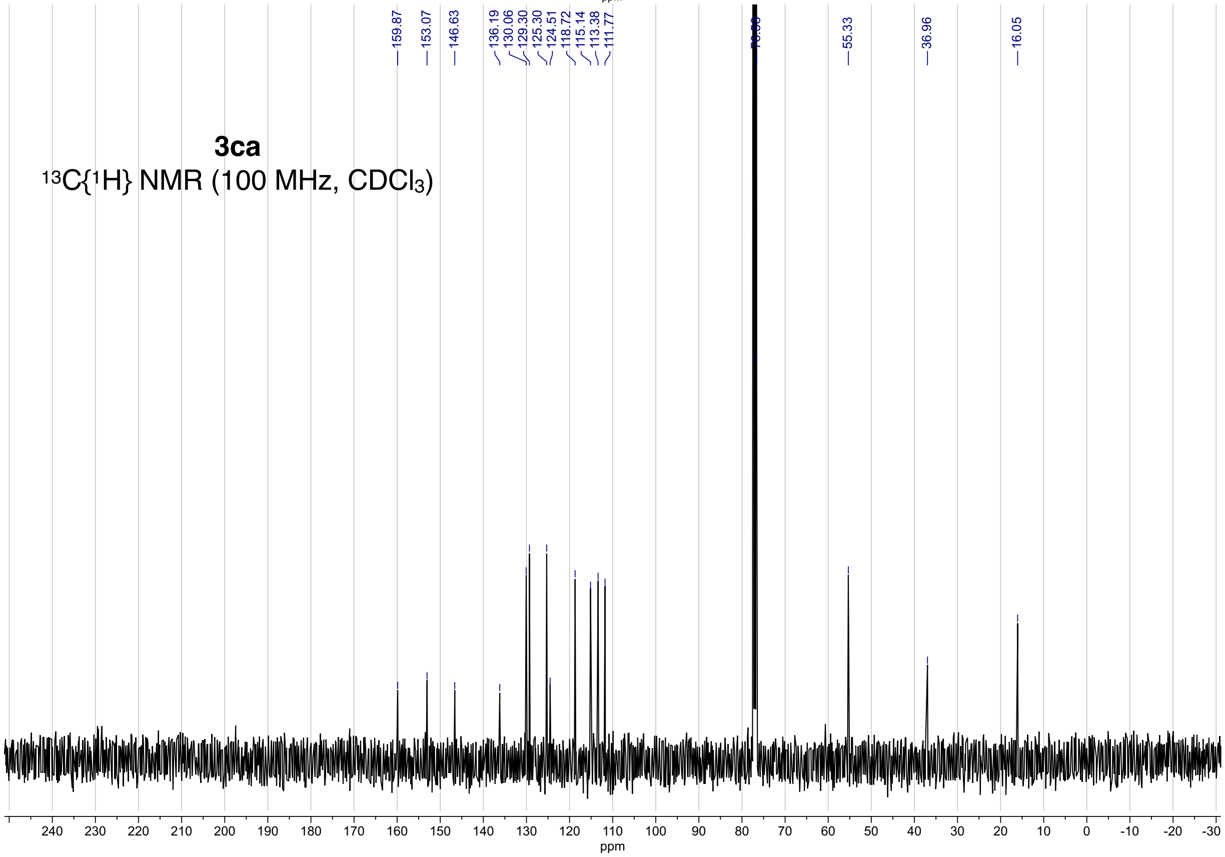
3ca
13C{1H} NMR (100 MHz, CDCl3)

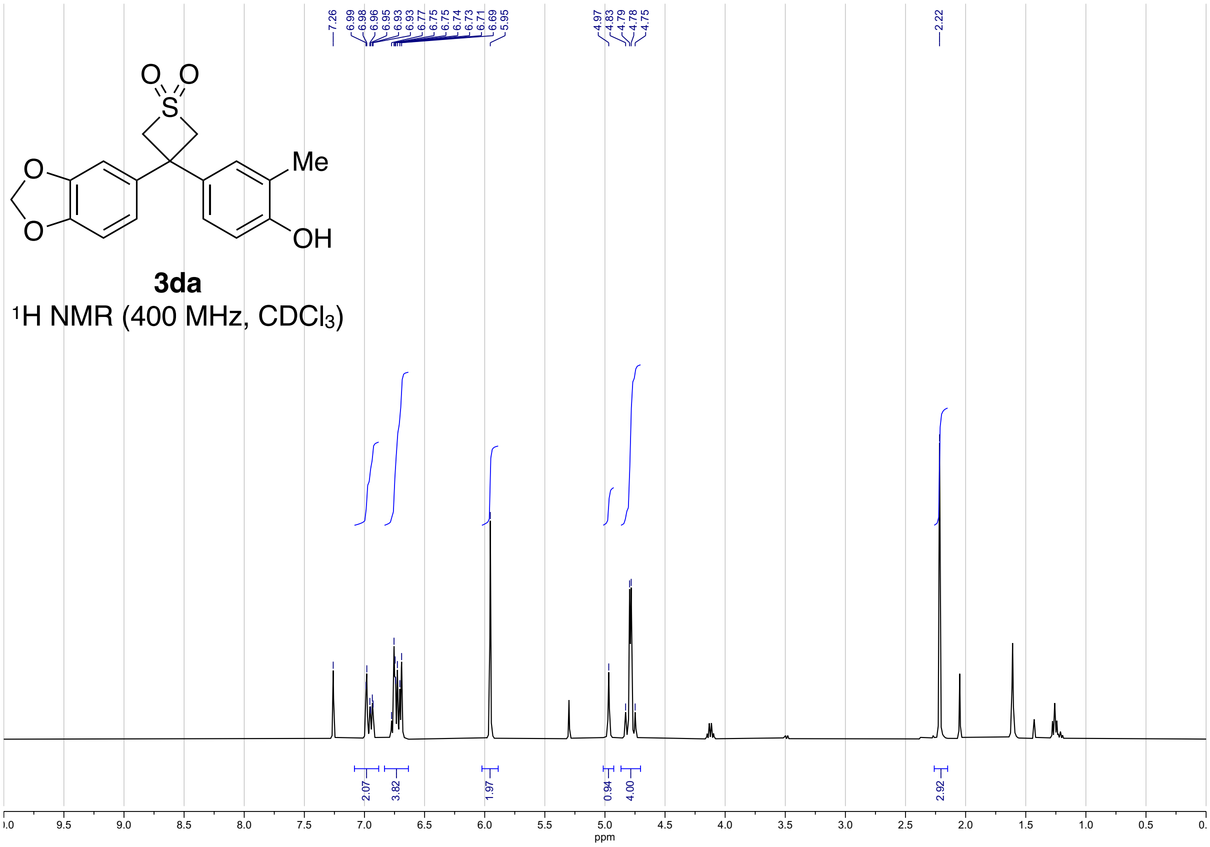
7.26
6.99
6.98
6.96
6.95
6.93
6.93
6.77
6.75
6.75
6.74
6.73
6.71
6.69
5.95
4.97
4.83
4.79
4.78
4.75
2.22
Me
OH
3da
¹H NMR (400 MHz, CDCl₃)
2.07
3.82
1.97
0.94
4.00
2.92
ppm

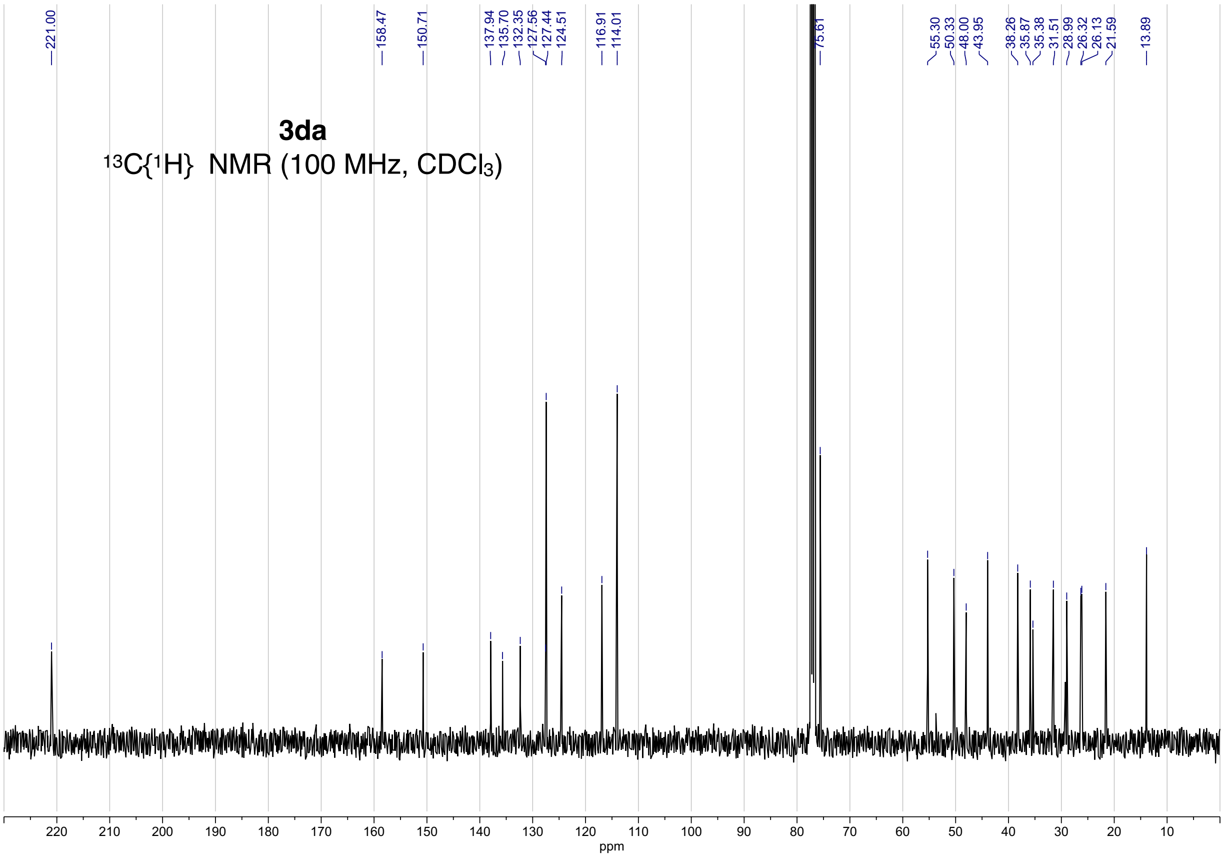
221.00
158.47
150.71
137.94
135.70
132.35
127.56
127.44
124.51
116.91
114.01
75.61
55.30
50.33
48.00
43.95
38.26
35.87
35.38
31.51
28.99
26.32
26.13
21.59
13.89
3da
¹³C{¹H} NMR (100 MHz, CDCl₃)
ppm

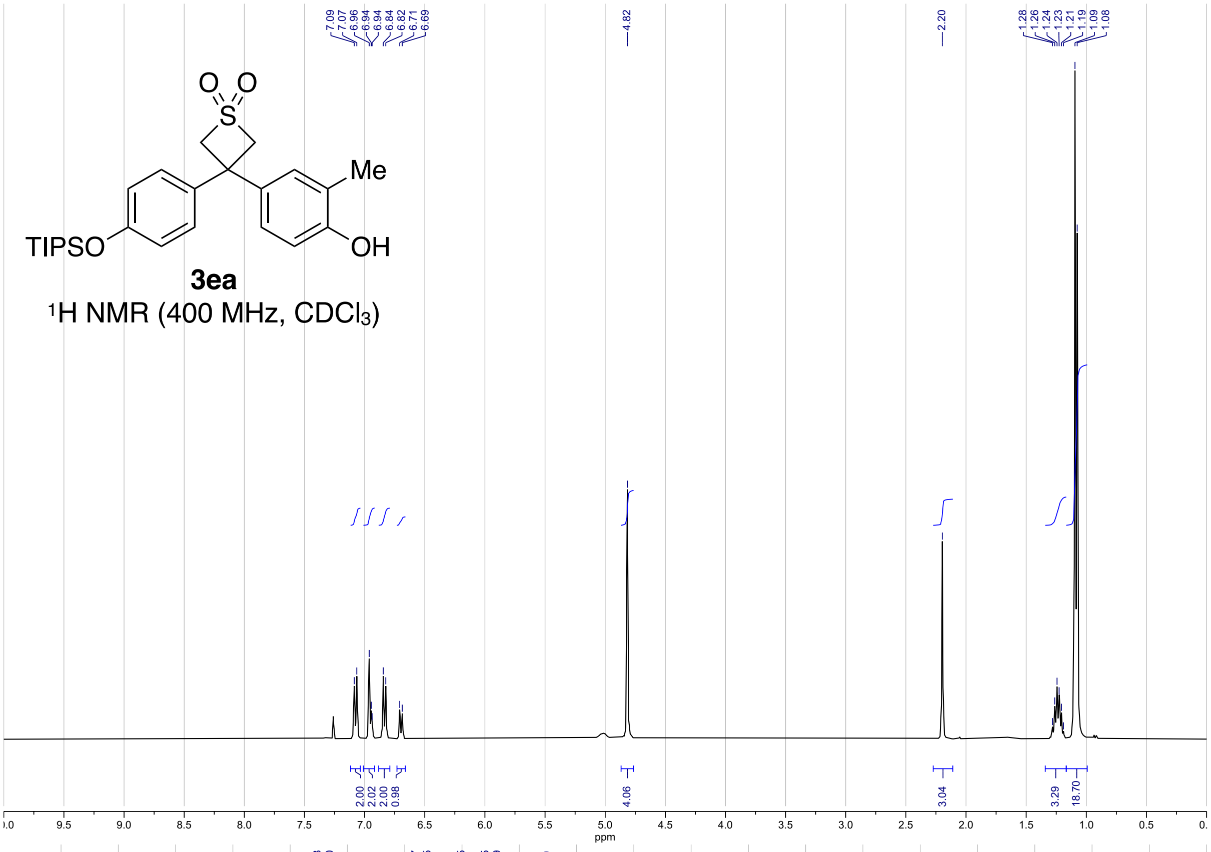

7.09
7.07
6.96
6.94
6.94
6.84
6.82
6.71
6.69
4.82
2.20
1.28
1.26
1.24
1.23
1.21
1.19
1.09
1.08
O
O
S
Me
TIPSO
OH
3ea
^{1}H NMR (400 MHz, $CDCl_3$)
2.00
2.02
2.00
0.98
4.06
3.04
3.29
18.70
ppm

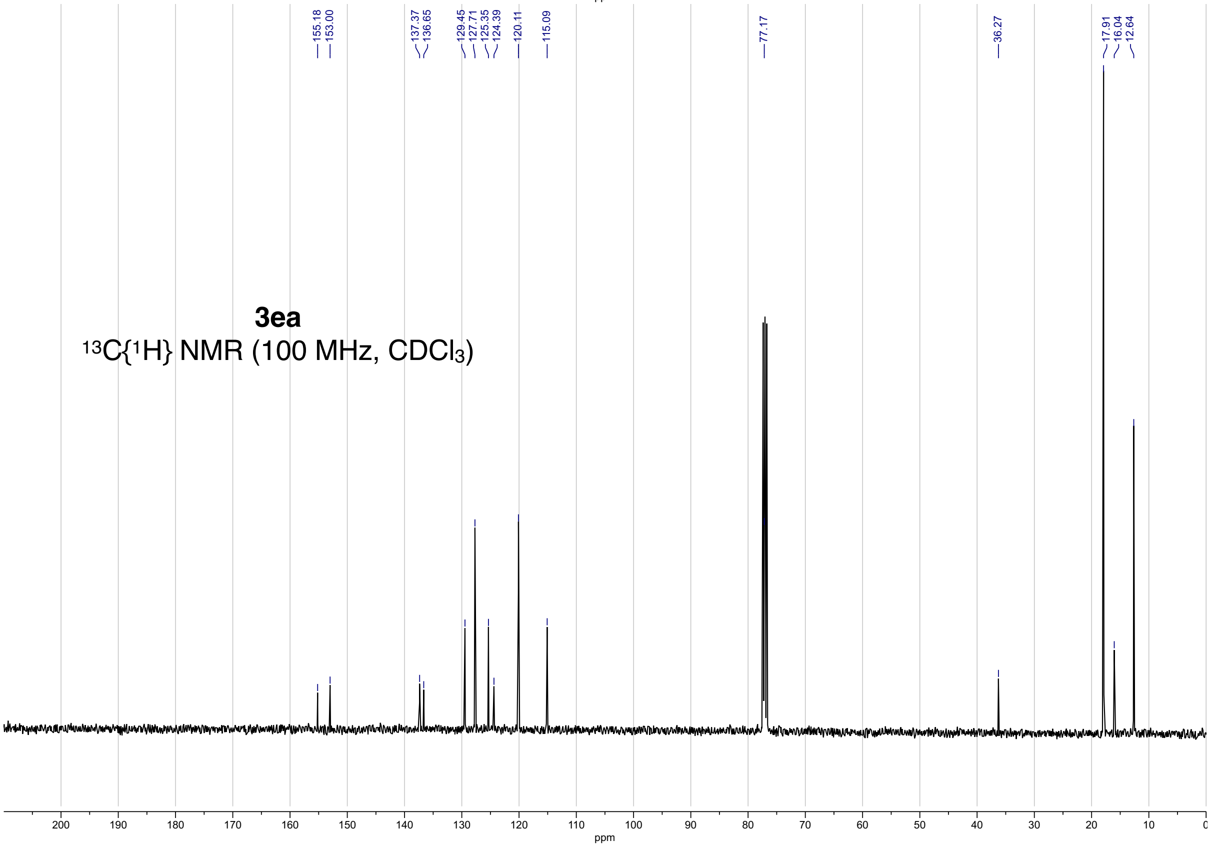

155.18
153.00
137.37
136.65
129.45
127.71
125.35
124.39
120.11
115.09
77.17
36.27
17.91
16.04
12.64
3ea
$^{13}C\{^1H\}$ NMR (100 MHz, $CDCl_3$)
ppm

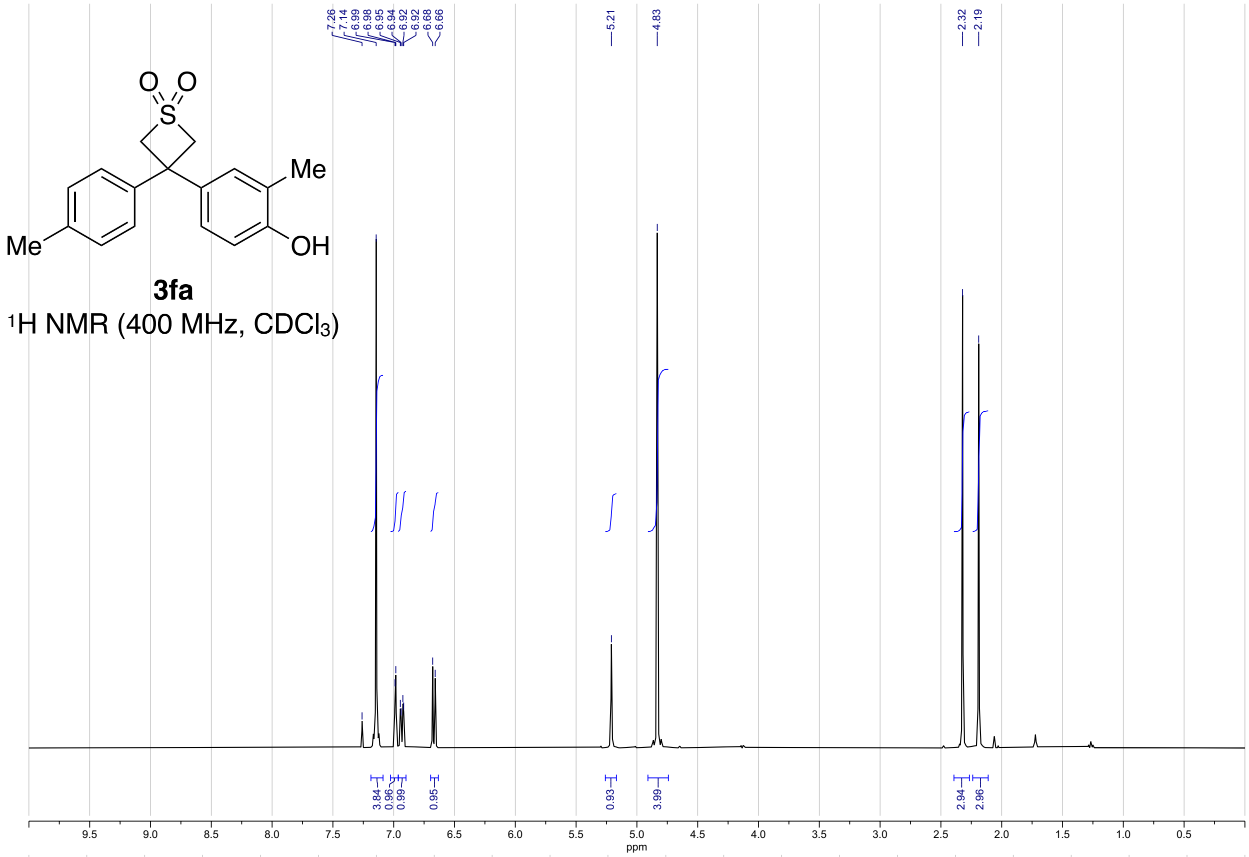
Me
OH
Me
3fa
¹H NMR (400 MHz, CDCl₃)
ppm

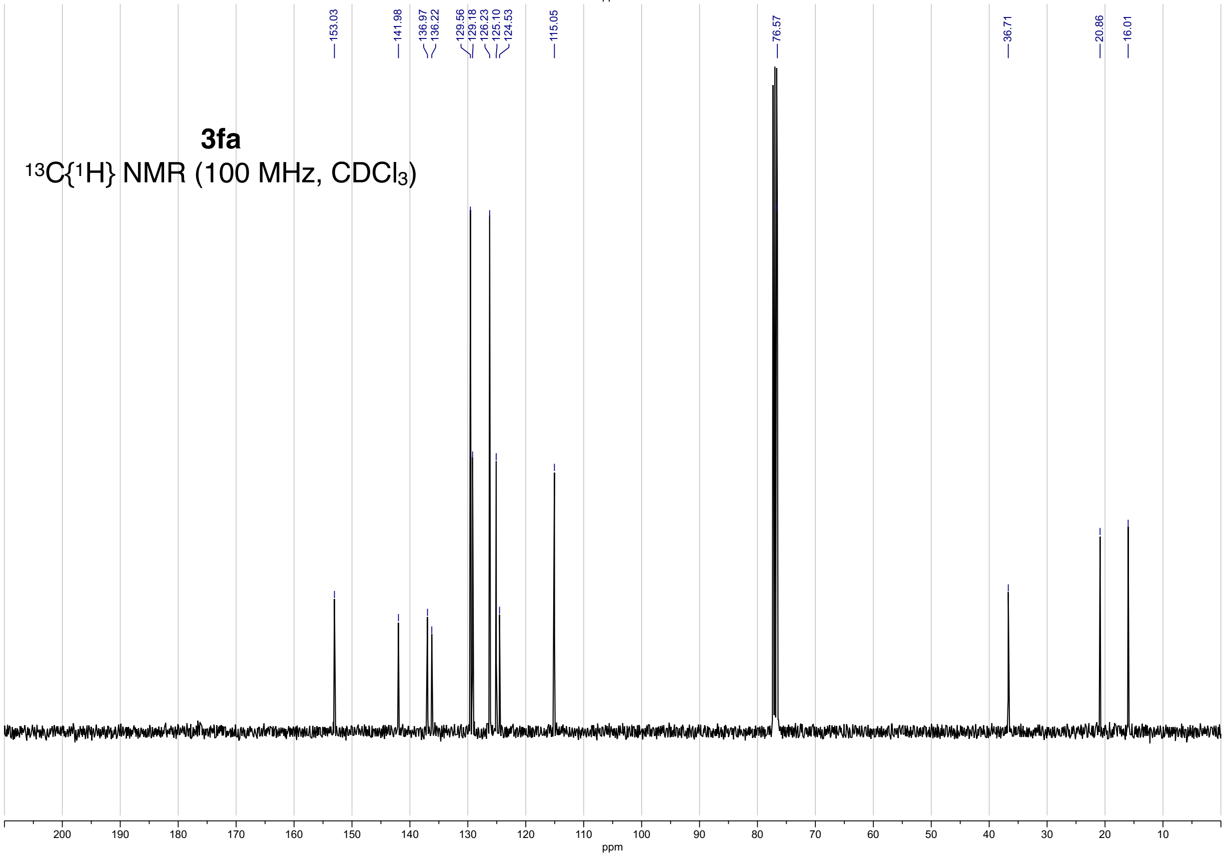
3fa
¹³C{¹H} NMR (100 MHz, CDCl₃)
ppm

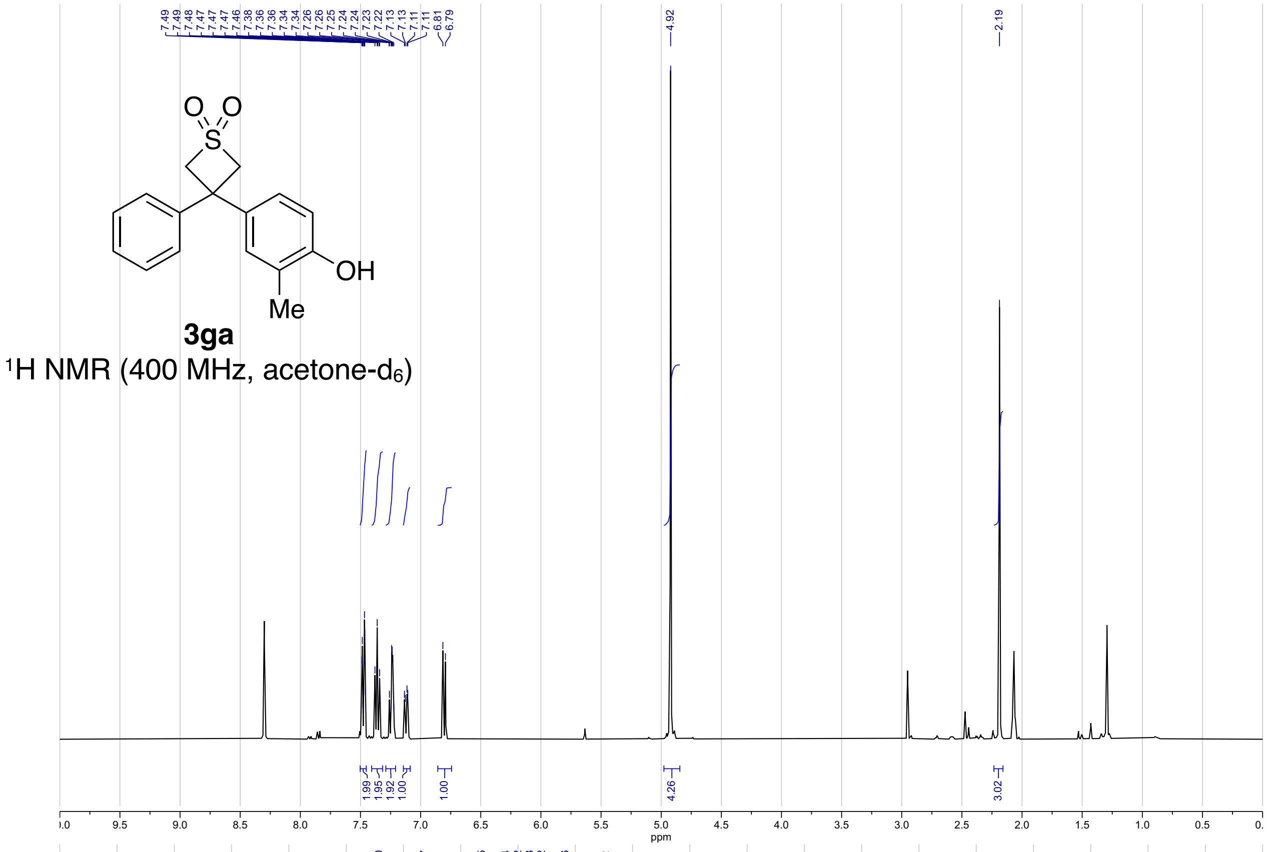
OH
Me
3ga
1H NMR (400 MHz, acetone-d6)
ppm

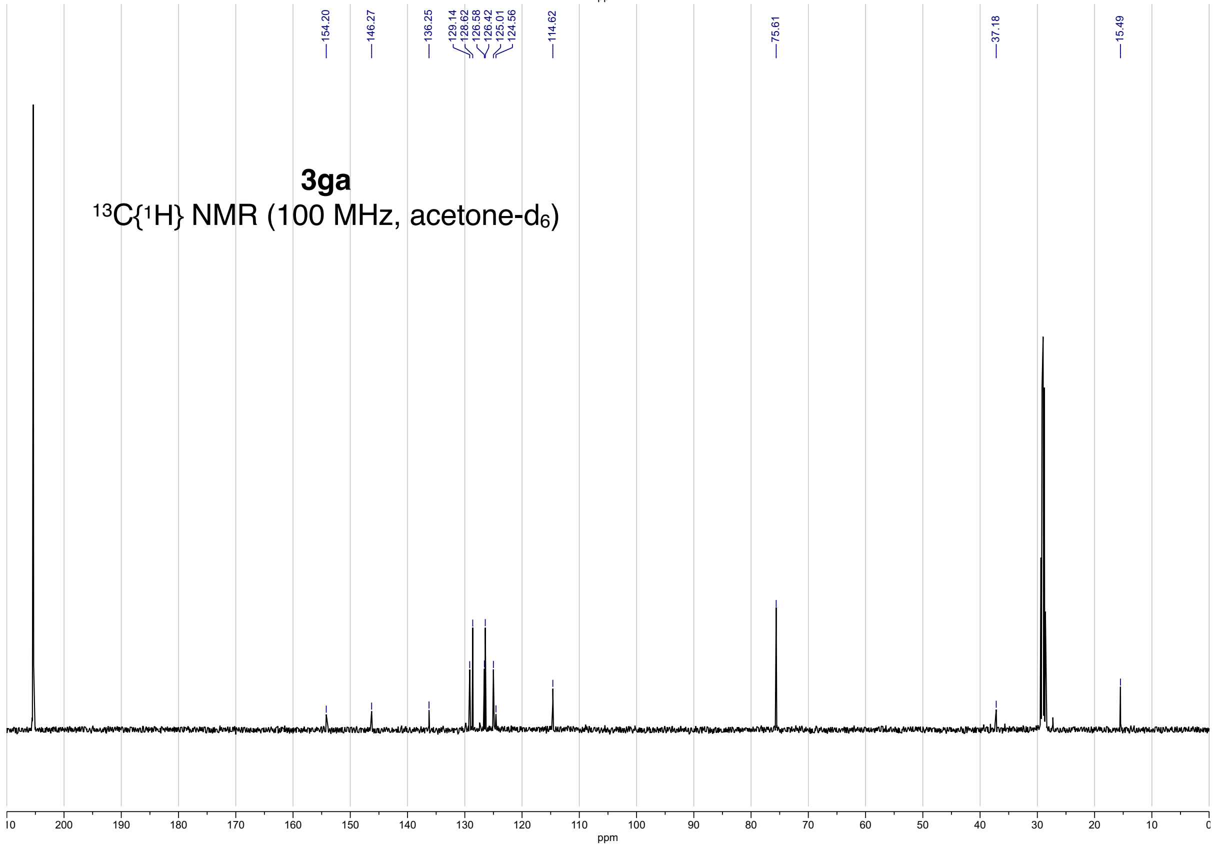
3ga
13C{1H} NMR (100 MHz, acetone-d6)
ppm

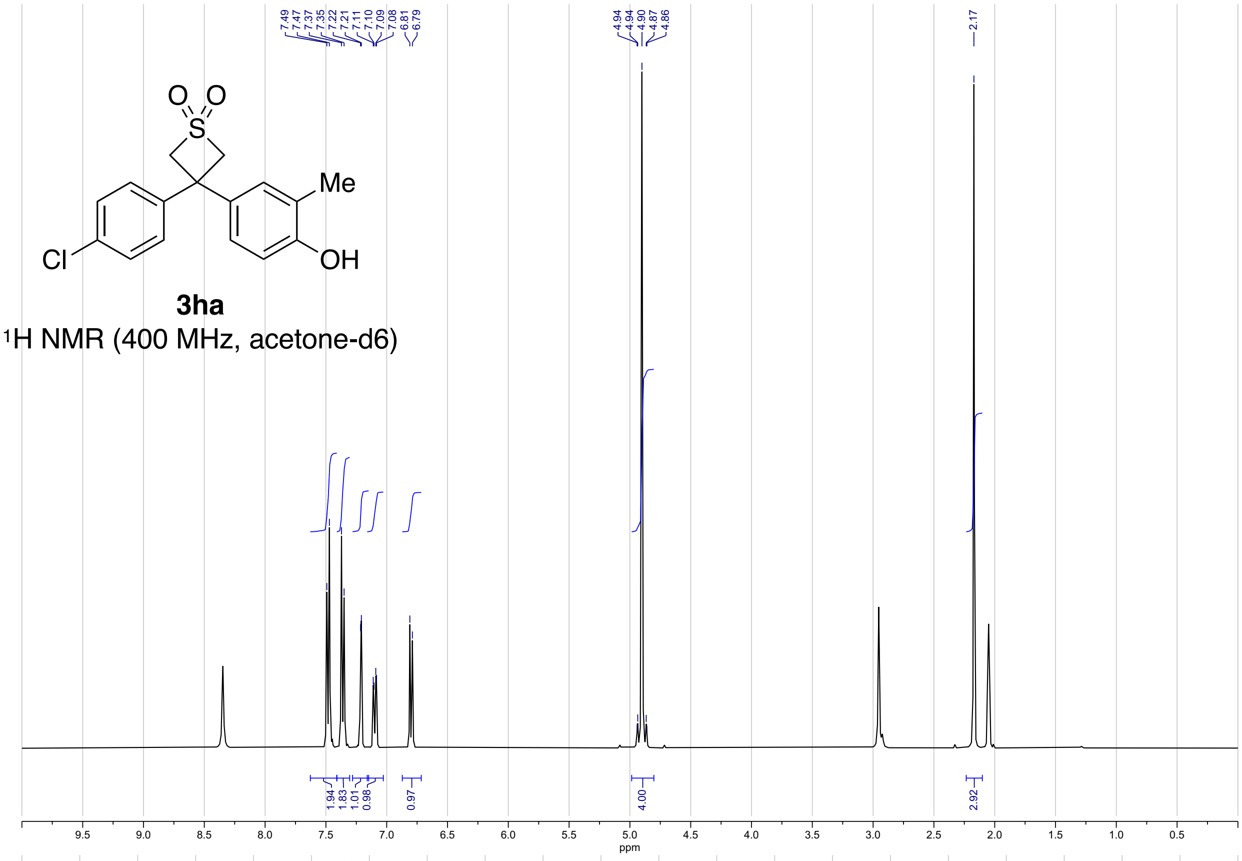

3ha
^{1}H NMR (400 MHz, acetone-d6)

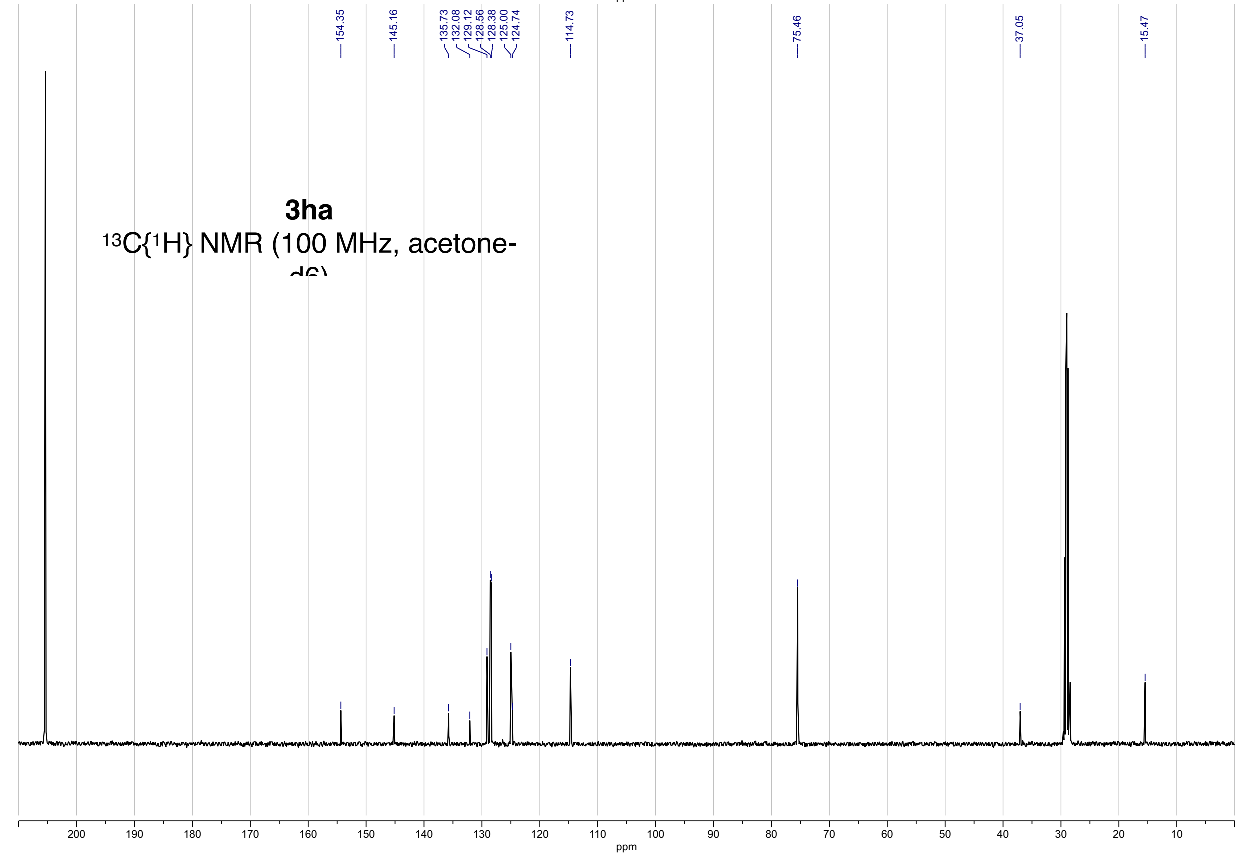

3ha
^{13}C{^{1}H} NMR (100 MHz, acetone-d6)

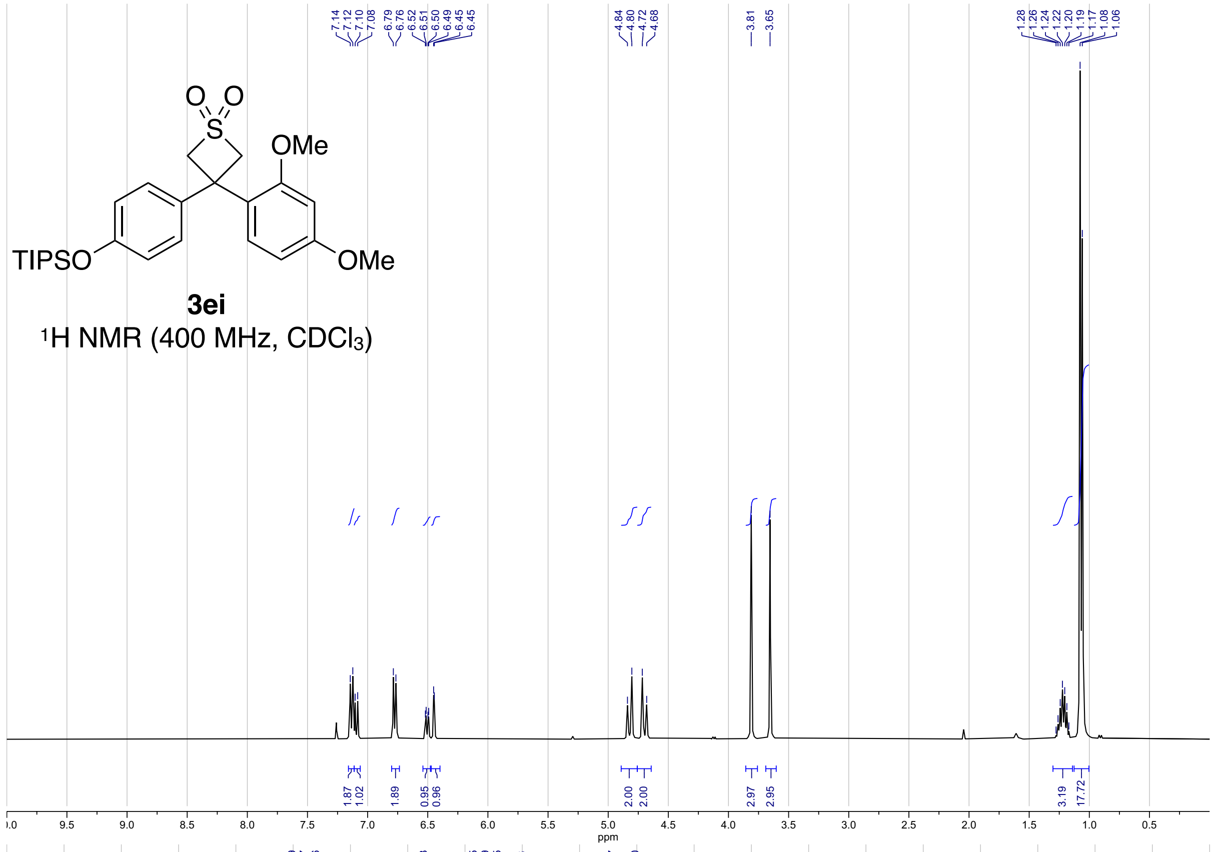
OMe
TIPSO
OMe
3ei
¹H NMR (400 MHz, CDCl₃)
7.14 7.12 7.10 7.08 6.79 6.76 6.52 6.51 6.50 6.49 6.45 6.45
4.84 4.80 4.72 4.68
3.81 3.65
1.28 1.26 1.24 1.22 1.20 1.19 1.17 1.08 1.06
1.87 1.02 1.89 0.95 0.96 2.00 2.00 2.97 2.95 3.19 17.72
ppm

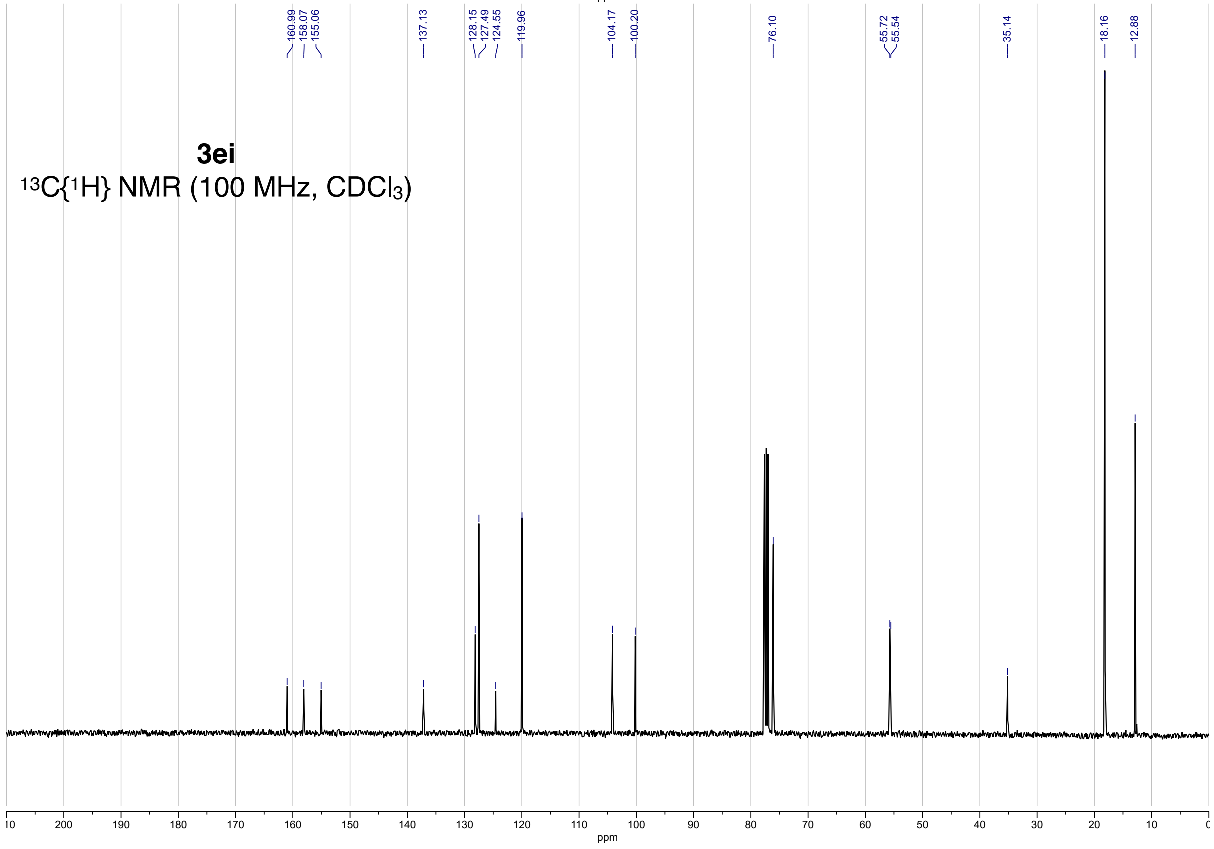
3ei
¹³C{¹H} NMR (100 MHz, CDCl₃)
160.99 158.07 155.06 137.13 128.15 127.49 124.55 119.96 104.17 100.20 76.10 55.72 55.54 35.14 18.16 12.88
ppm

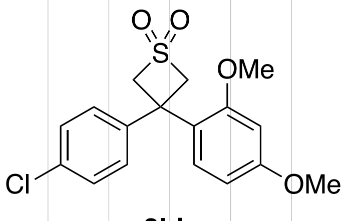

3hi
^{1}H NMR (400 MHz, $CDCl_3$)

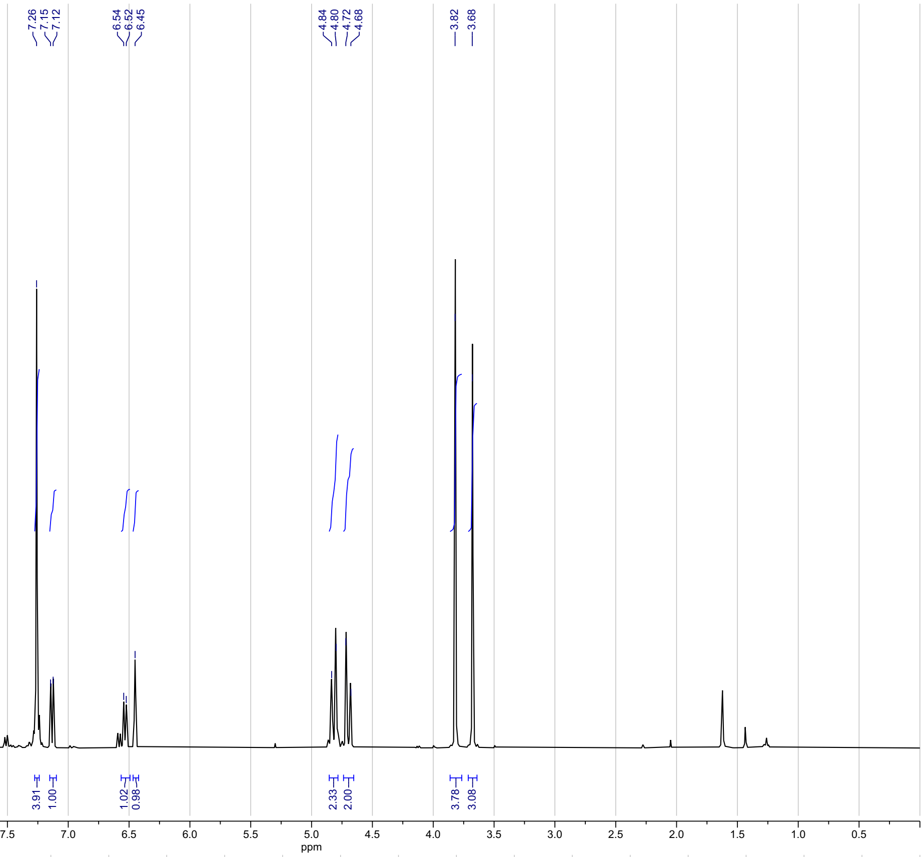

3hi
^{13}C{^{1}H} NMR (100 MHz, $CDCl_3$)

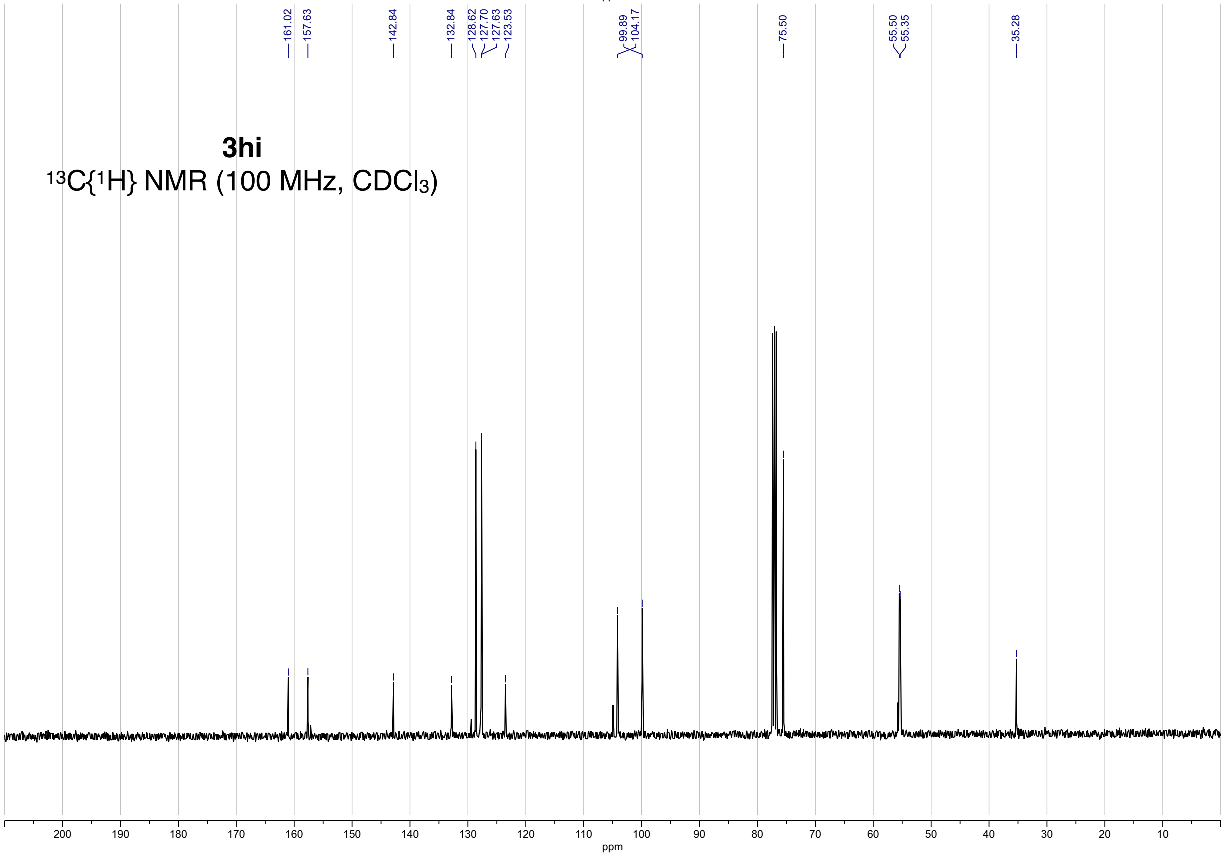

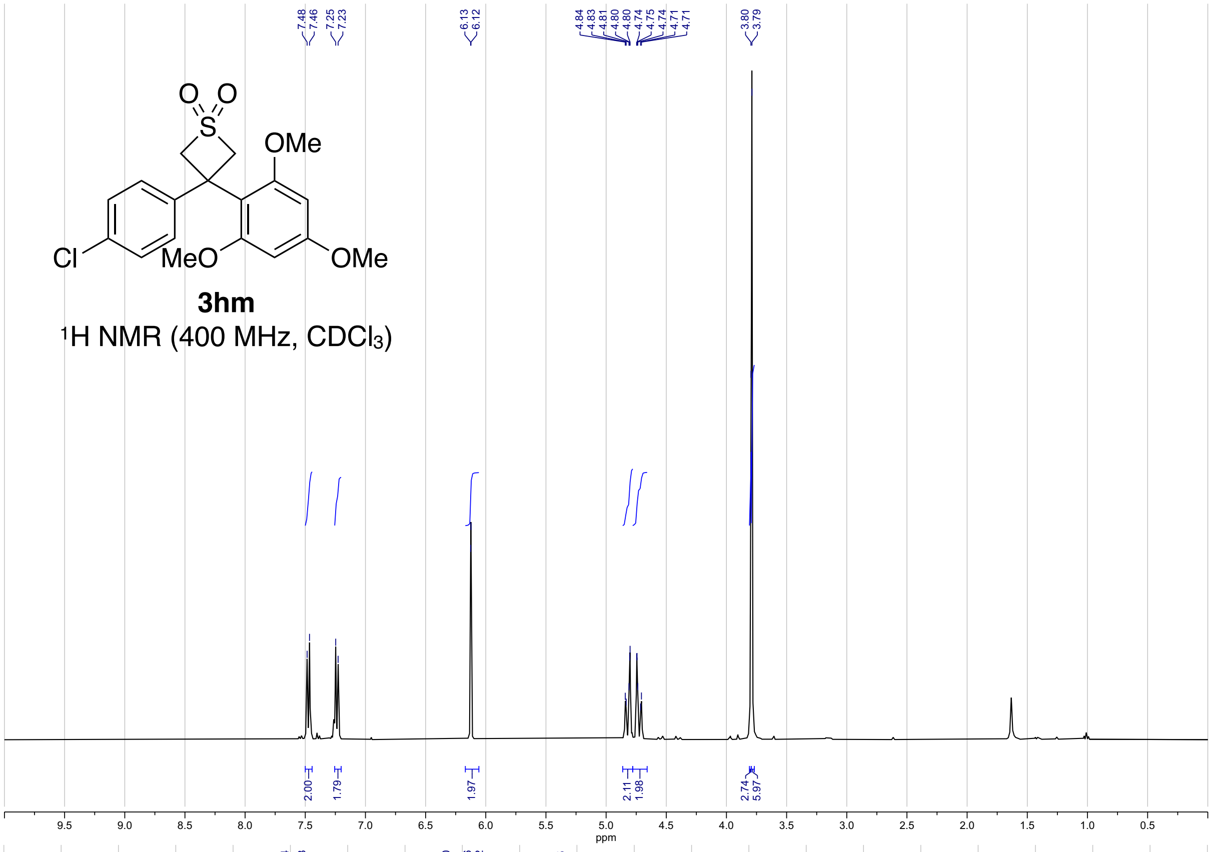

3hm

^{1}H NMR (400 MHz, $CDCl_3$)

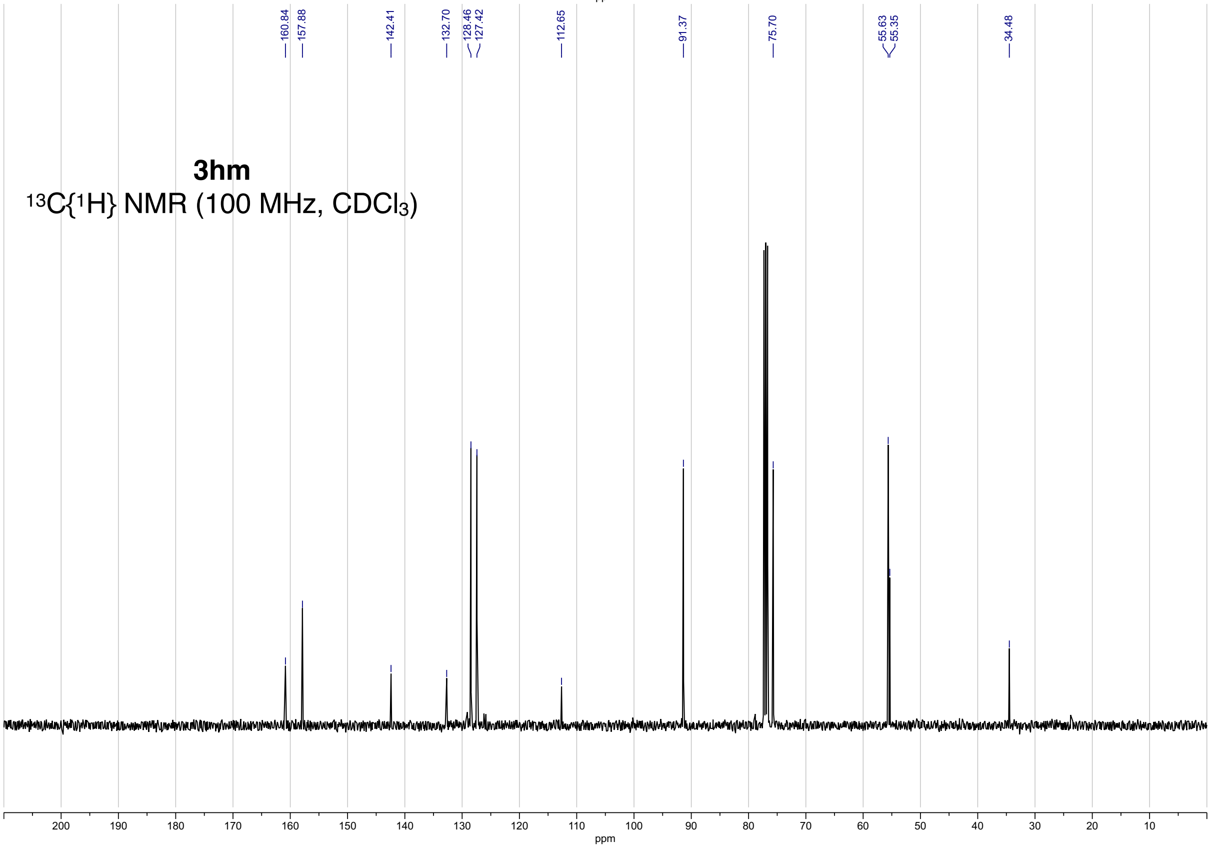

3hm

$^{13}C\{^1H\}$ NMR (100 MHz, $CDCl_3$)

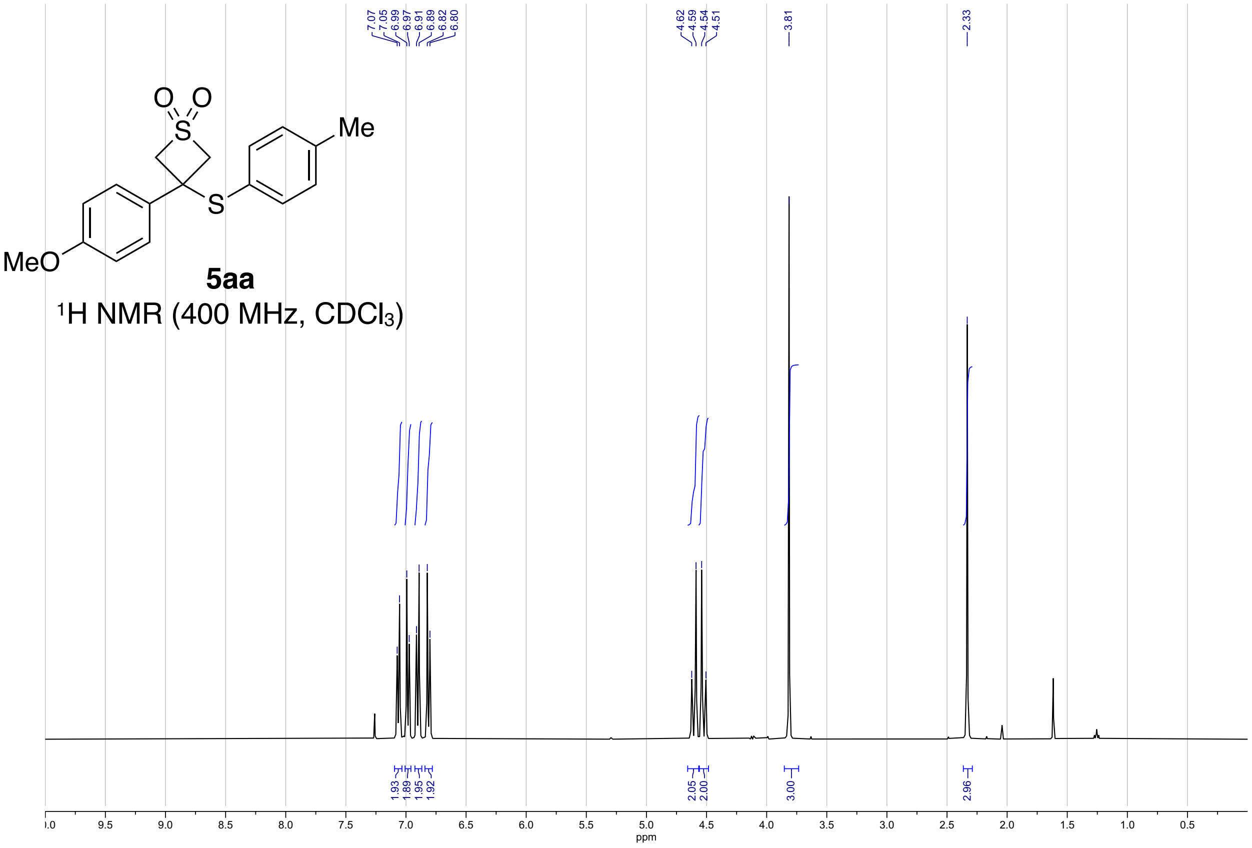
7.07
7.05
6.99
6.97
6.91
6.89
6.82
6.80
4.62
4.59
4.54
4.51
3.81
2.33
O
O
S
Me
S
MeO
5aa
¹H NMR (400 MHz, CDCl₃)
1.93
1.89
1.95
1.92
2.05
2.00
3.00
2.96
ppm

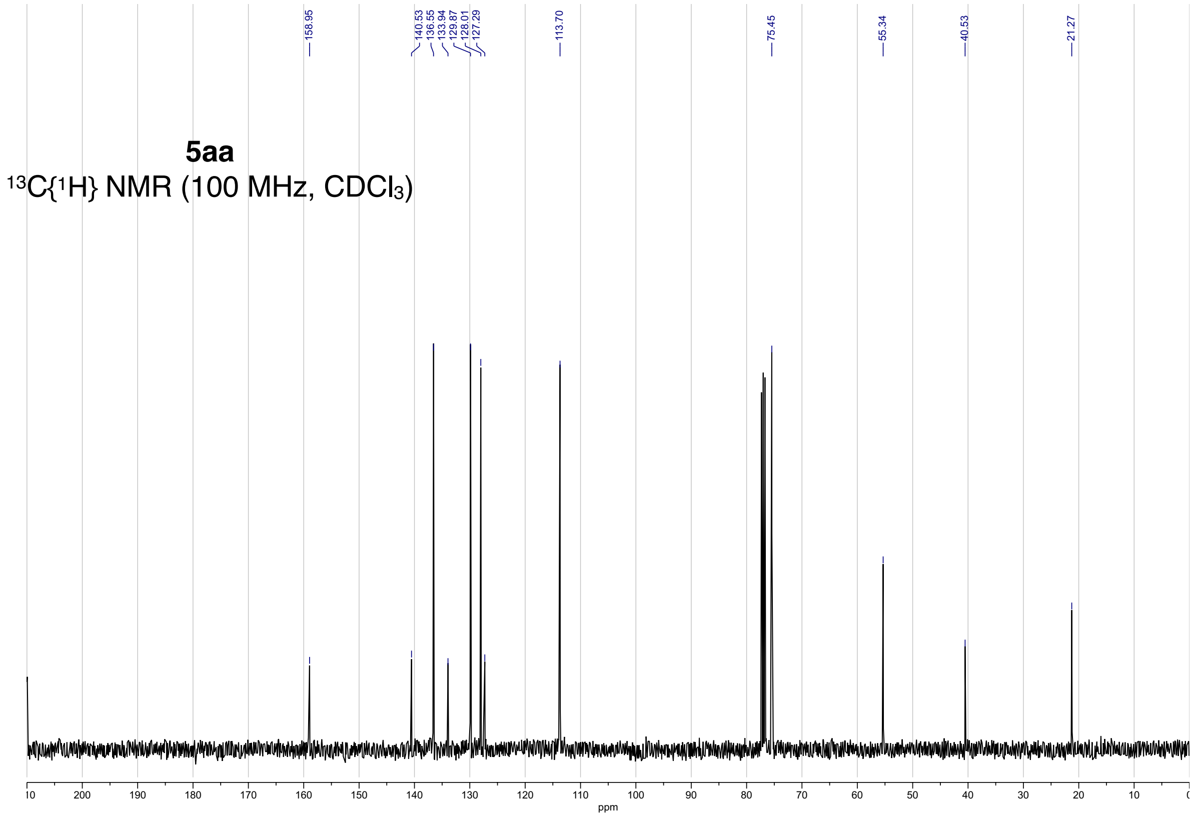
158.95
140.53
136.55
133.94
129.87
128.01
127.29
113.70
75.45
55.34
40.53
21.27
5aa
¹³C{¹H} NMR (100 MHz, CDCl₃)
ppm

5ab
^{1}H NMR (400 MHz, $CDCl_3$)

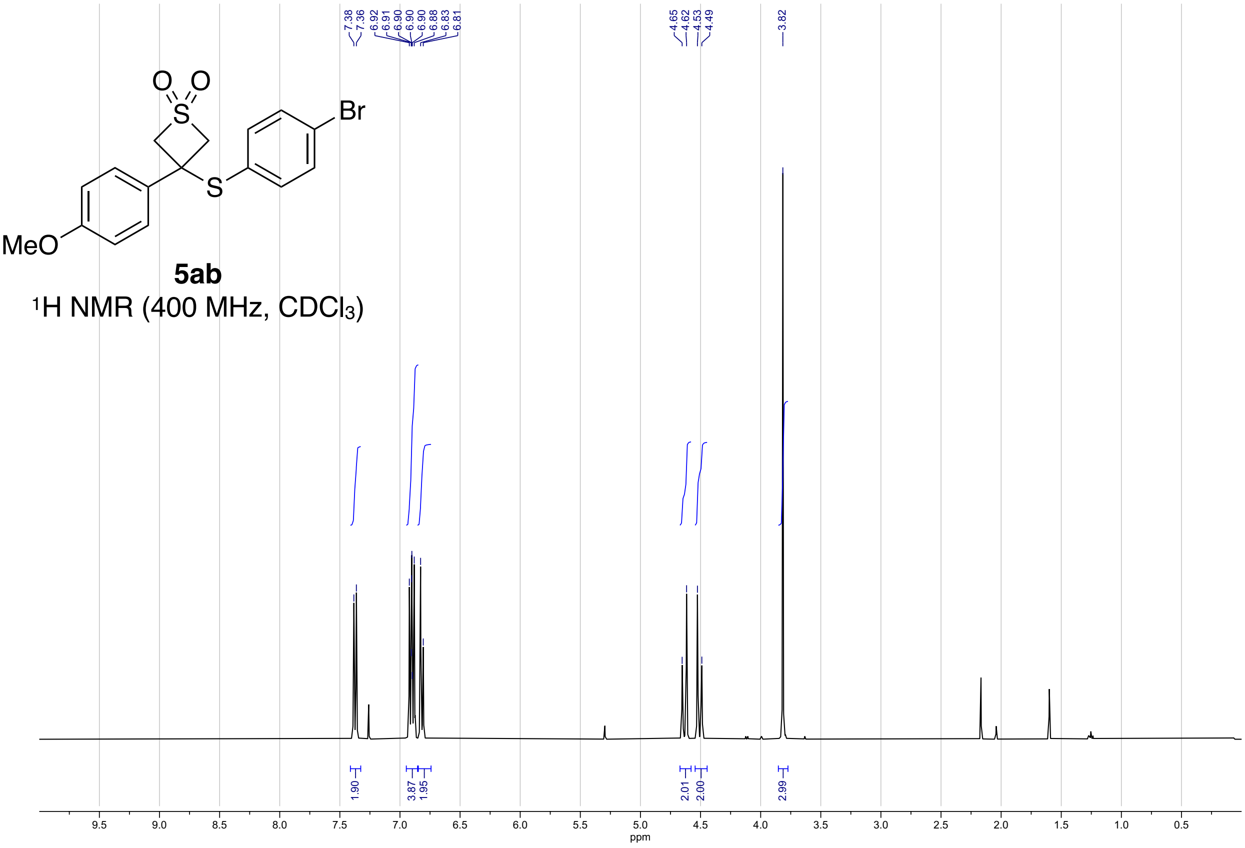

5ab
$^{13}C\{^{1}H\}$ NMR (100 MHz, $CDCl_3$)

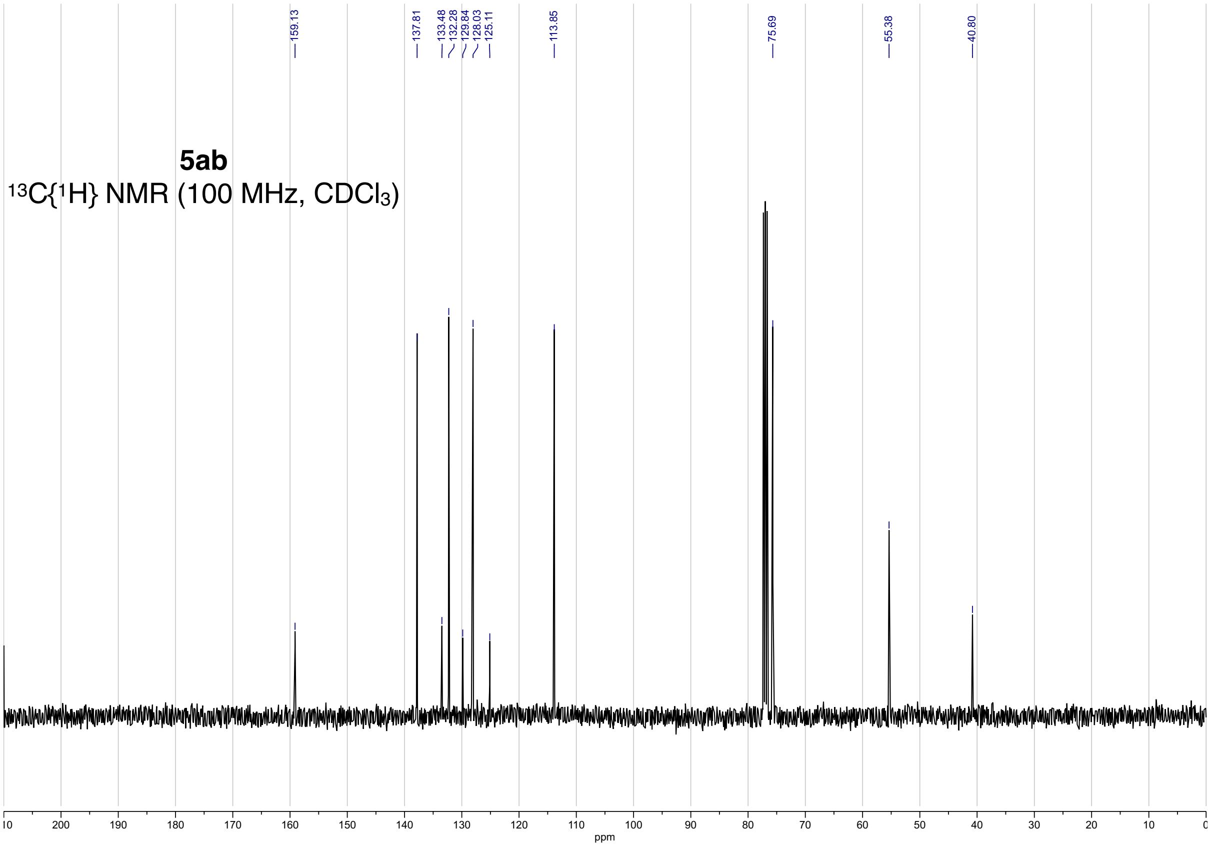

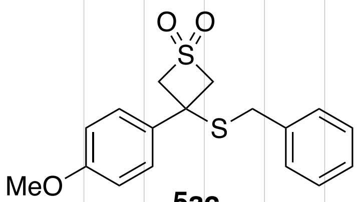

5ac

^1H NMR (400 MHz, $CDCl_3$)

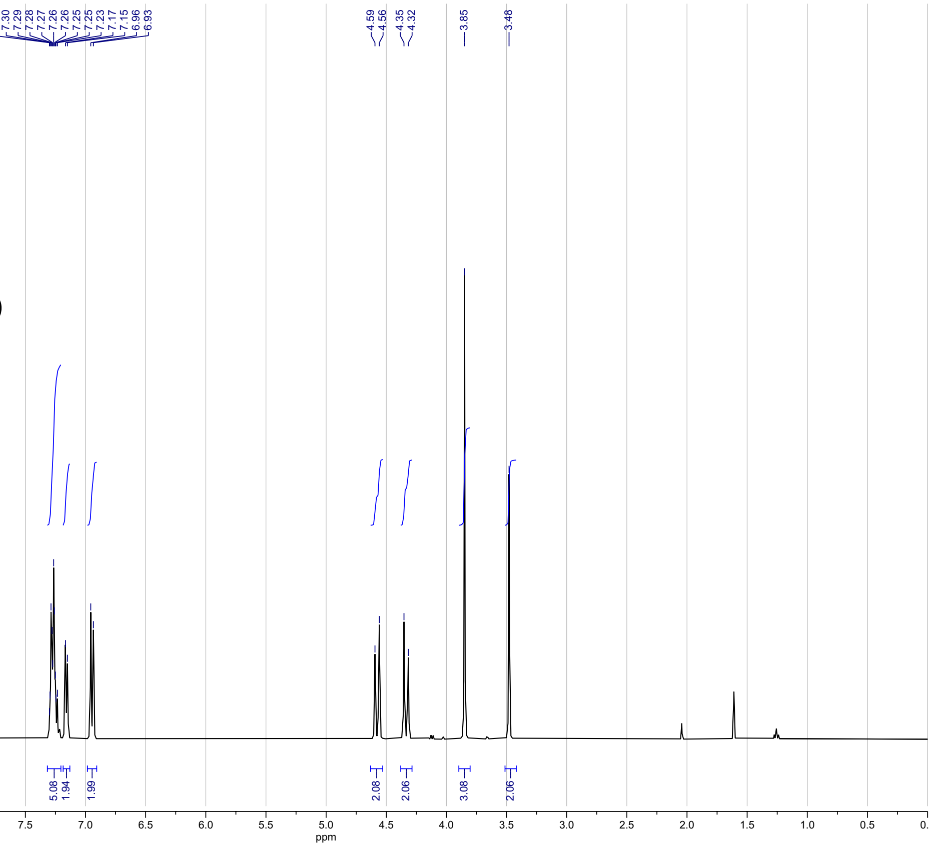

5ac

$^{13}C\{^1H\}$ NMR (100 MHz, $CDCl_3$)

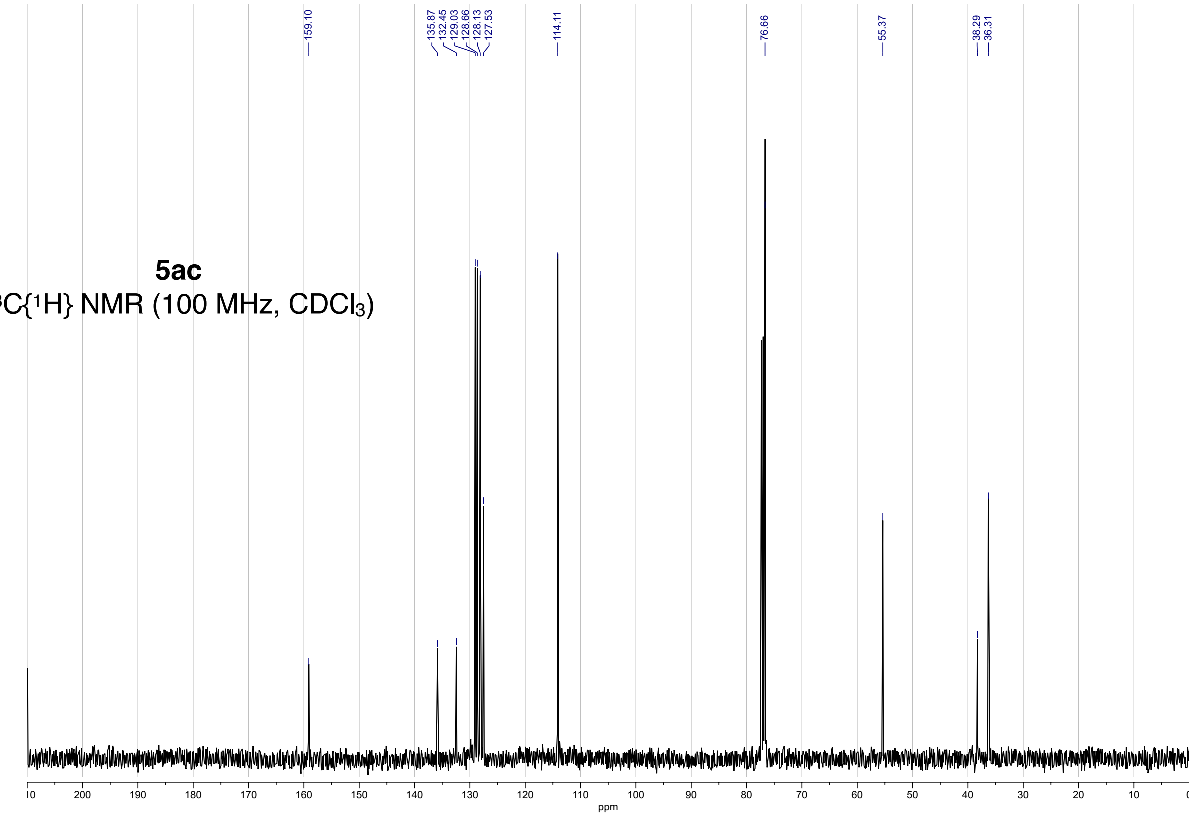

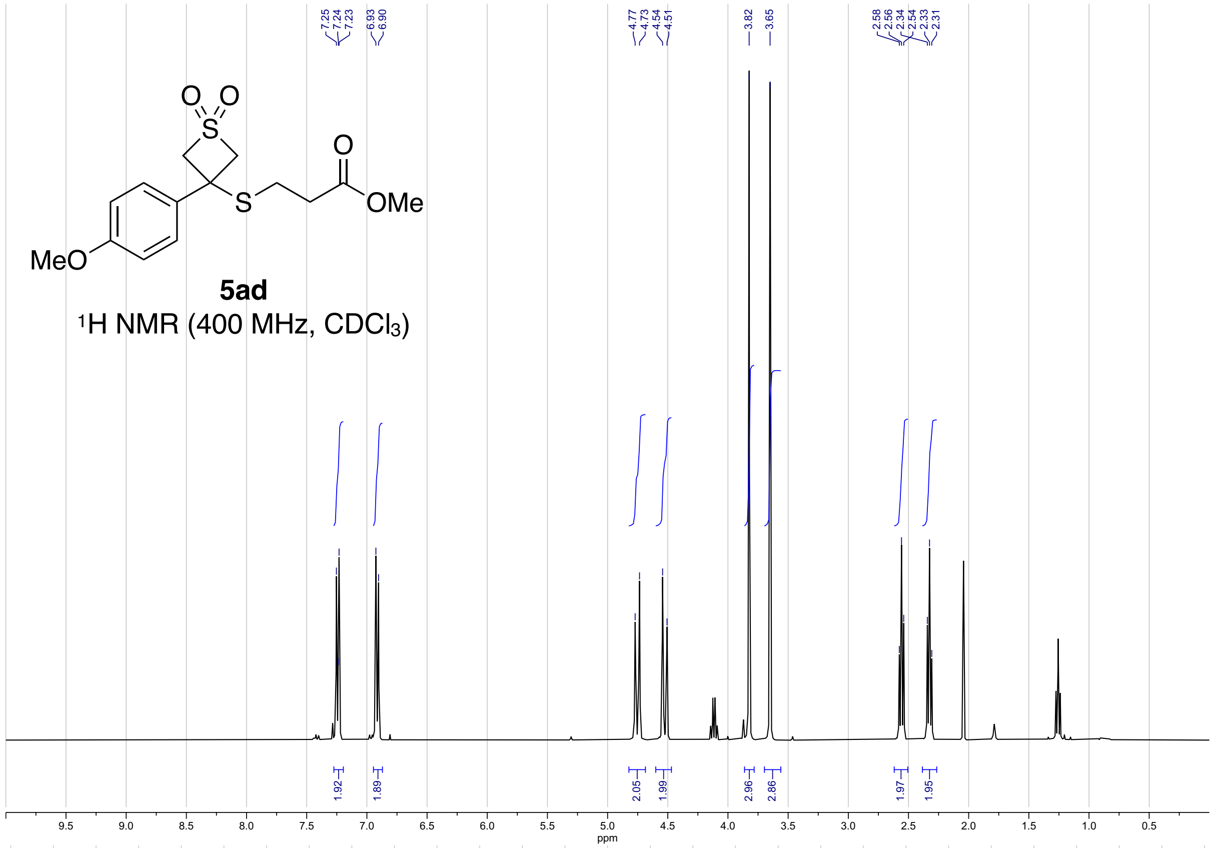

O O
S
O
S
OMe
MeO
5ad
1H NMR (400 MHz, CDCl3)
7.25 7.24 7.23 6.93 6.90
4.77 4.73 4.54 4.51
3.82 3.65
2.58 2.56 2.34 2.54 2.33 2.31
1.92 1.89 2.05 1.99 2.96 2.86 1.97 1.95
9.5 9.0 8.5 8.0 7.5 7.0 6.5 6.0 5.5 5.0 4.5 4.0 3.5 3.0 2.5 2.0 1.5 1.0 0.5
ppm

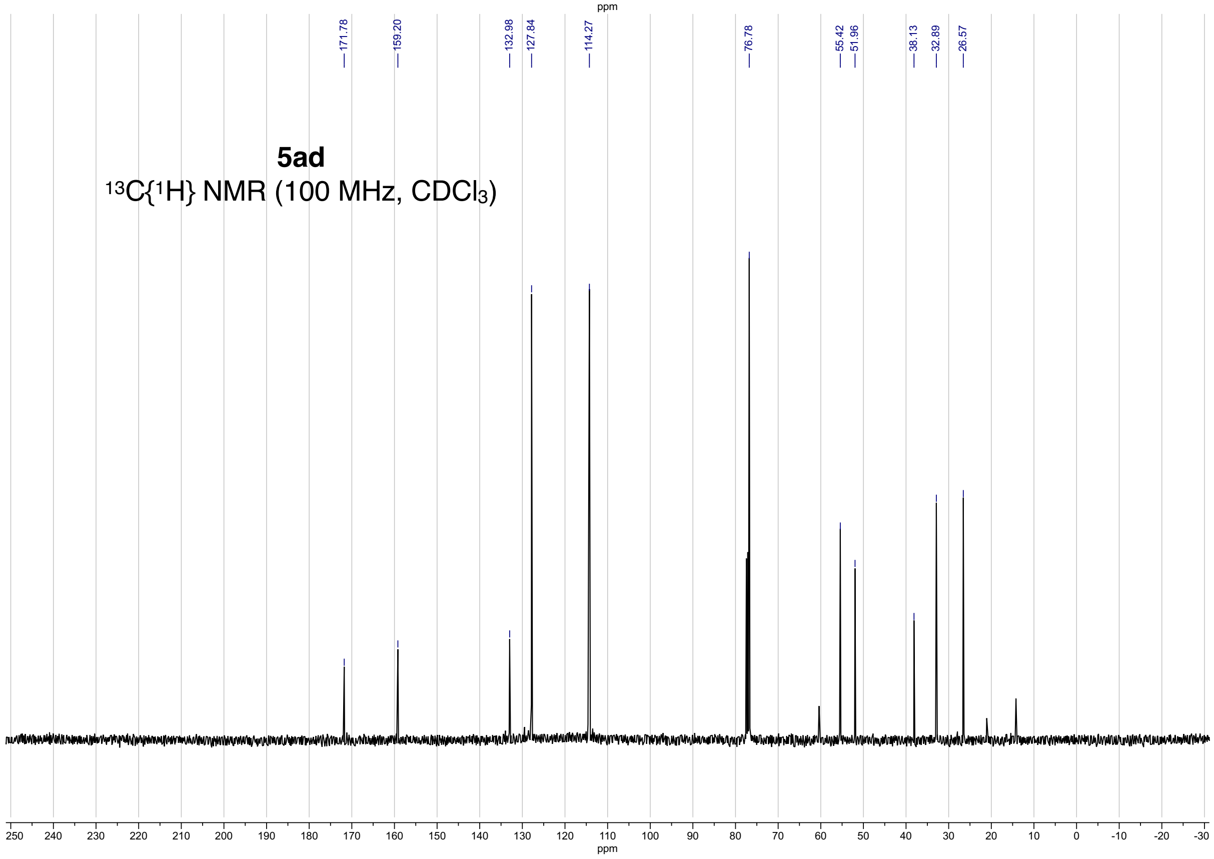

5ad
13C{1H} NMR (100 MHz, CDCl3)
171.78 159.20 132.98 127.84 114.27 76.78 55.42 51.96 38.13 32.89 26.57
250 240 230 220 210 200 190 180 170 160 150 140 130 120 110 100 90 80 70 60 50 40 30 20 10 0 -10 -20 -30
ppm

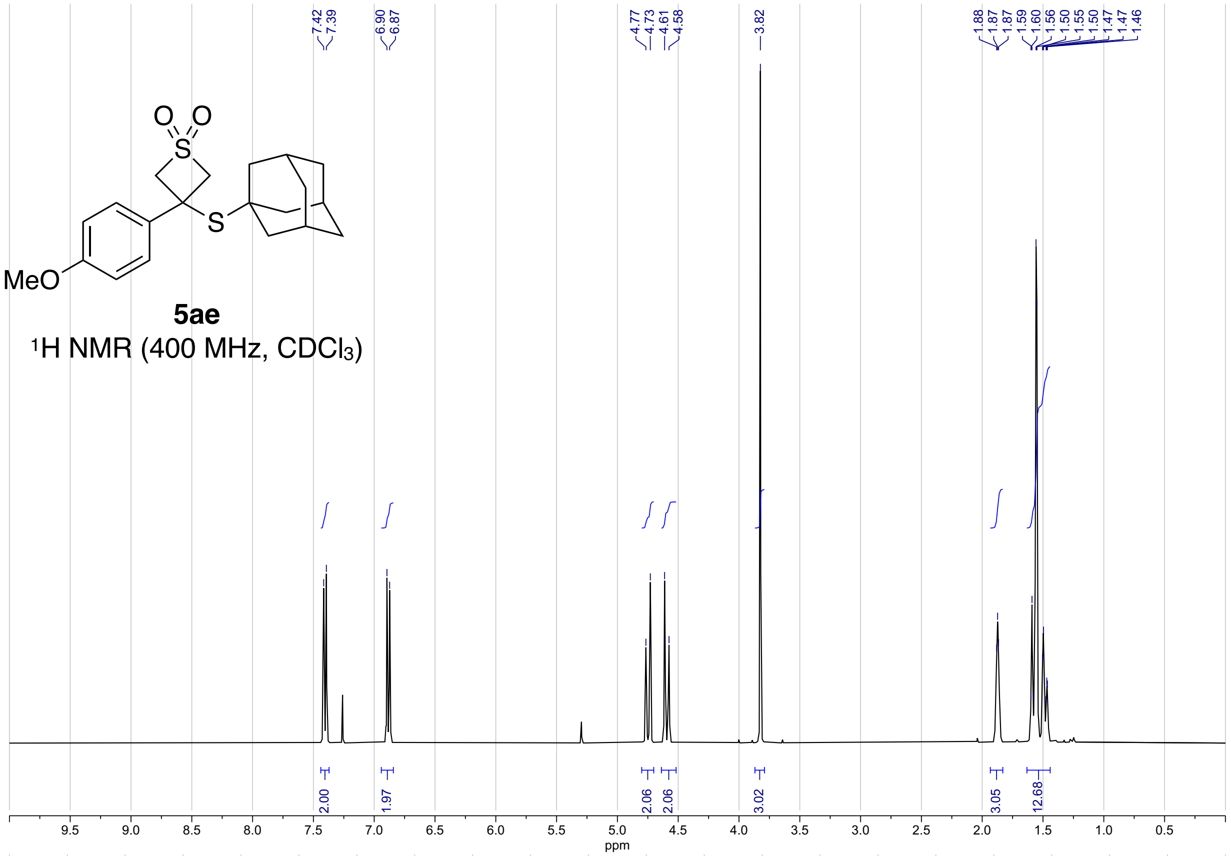
7.42
7.39
6.90
6.87
4.77
4.73
4.61
4.58
3.82
1.88
1.87
1.87
1.59
1.60
1.56
1.50
1.55
1.50
1.47
1.47
1.46
MeO
O
O
S
S
5ae
¹H NMR (400 MHz, CDCl₃)
2.00
1.97
2.06
2.06
3.02
3.05
12.68
ppm

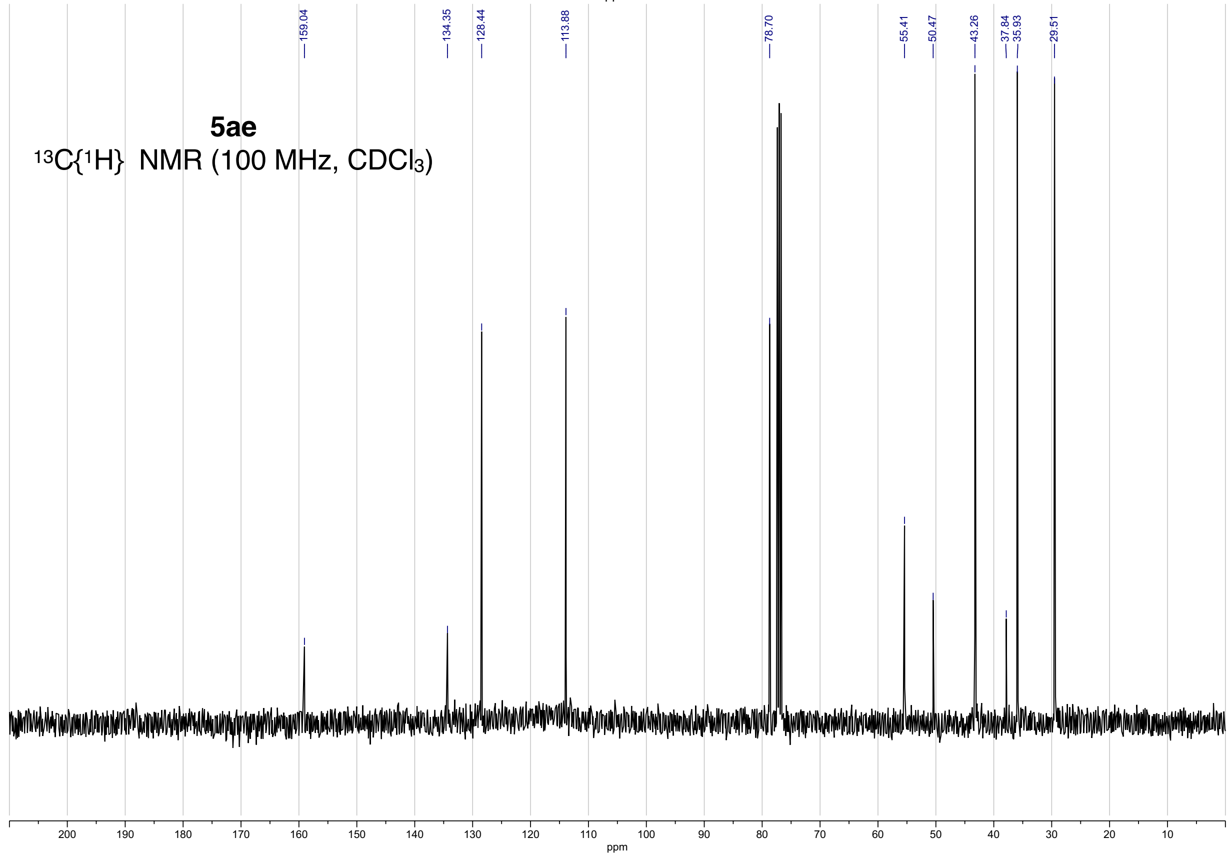
159.04
134.35
128.44
113.88
78.70
55.41
50.47
43.26
37.84
35.93
29.51
5ae
¹³C{¹H} NMR (100 MHz, CDCl₃)
ppm

5hb

^{1}H NMR (400 MHz, $CDCl_3$)

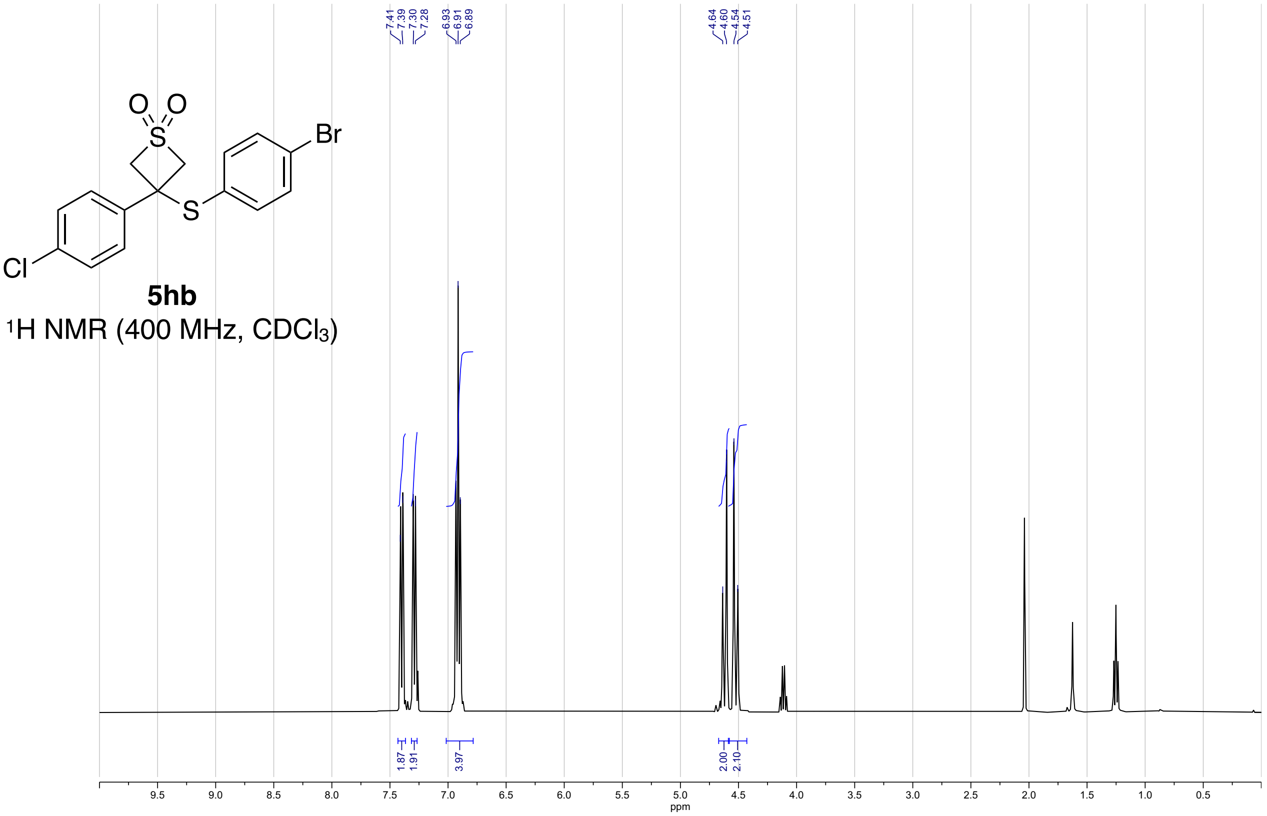

5hb

$^{13}C\{^{1}H\}$ NMR (100 MHz, $CDCl_3$)

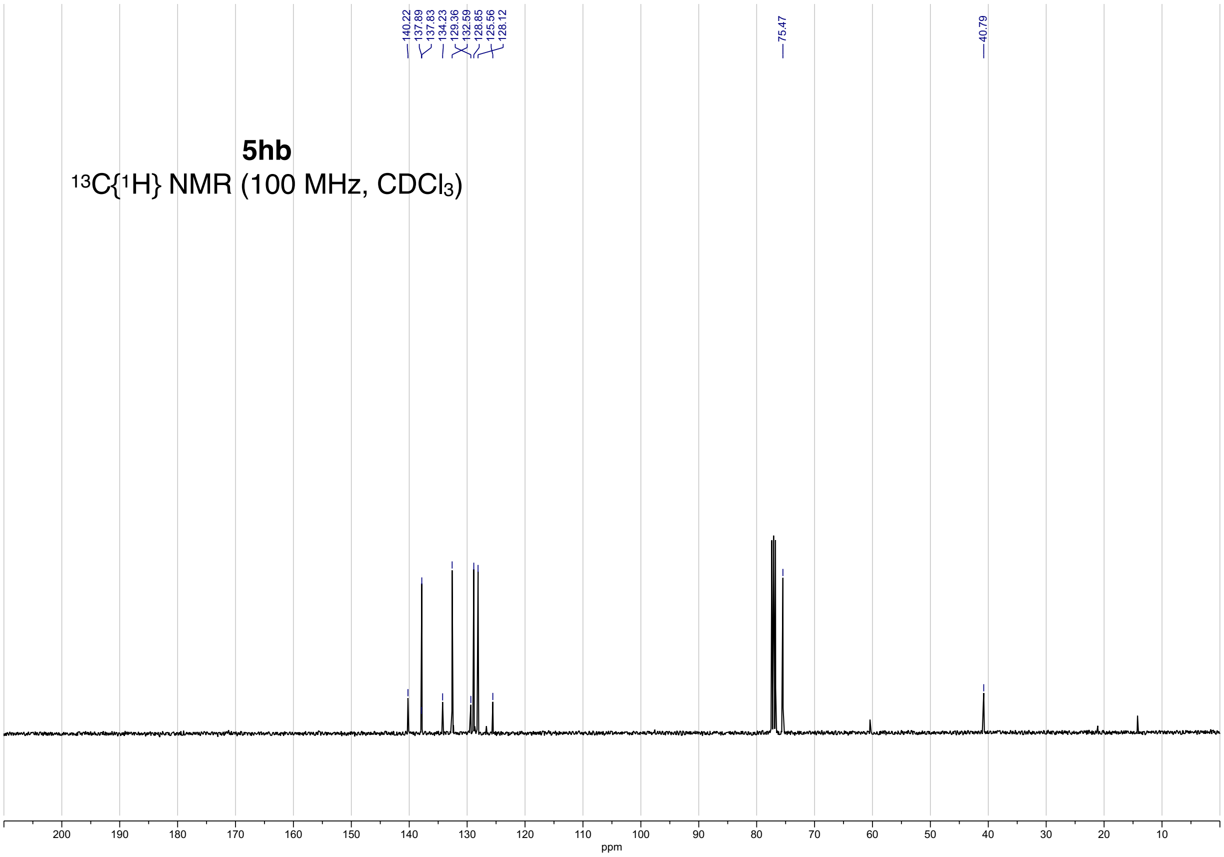

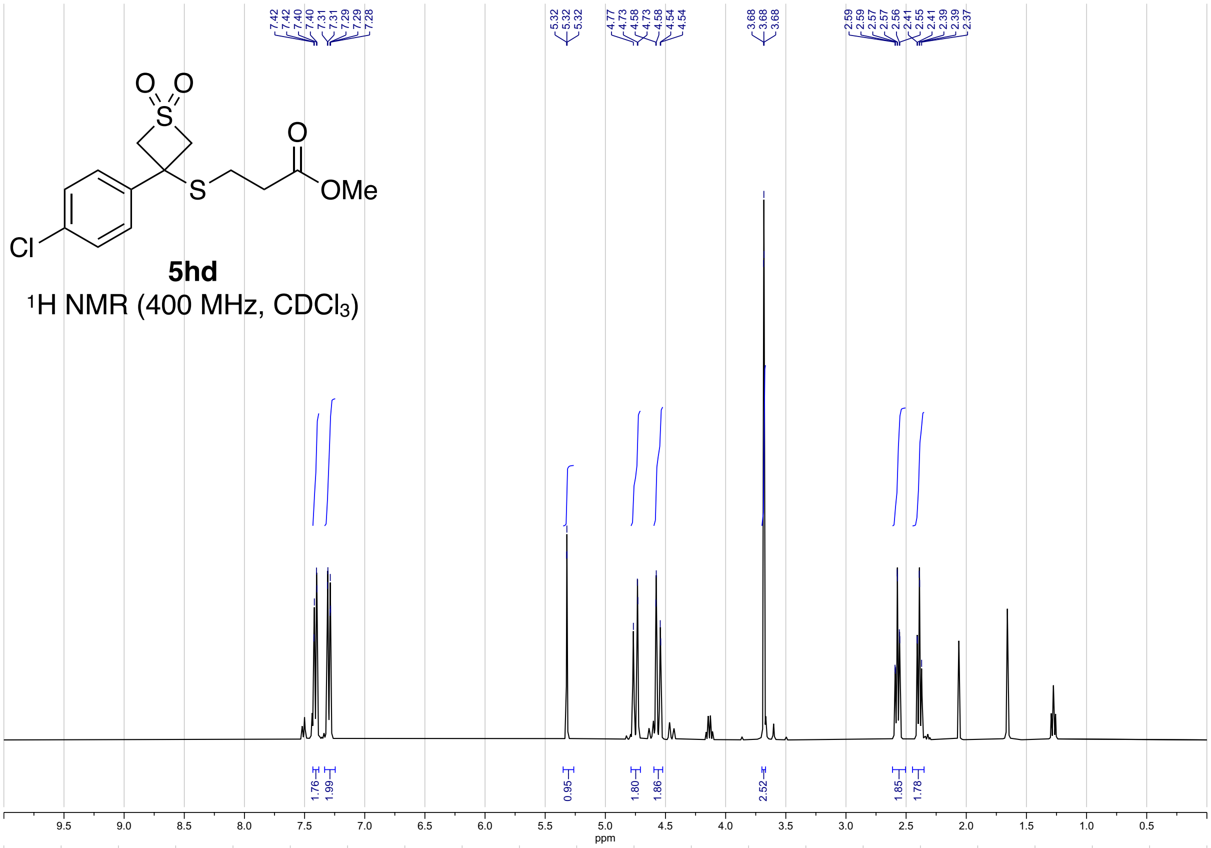

5hd
^{1}H NMR (400 MHz, $CDCl_3$)

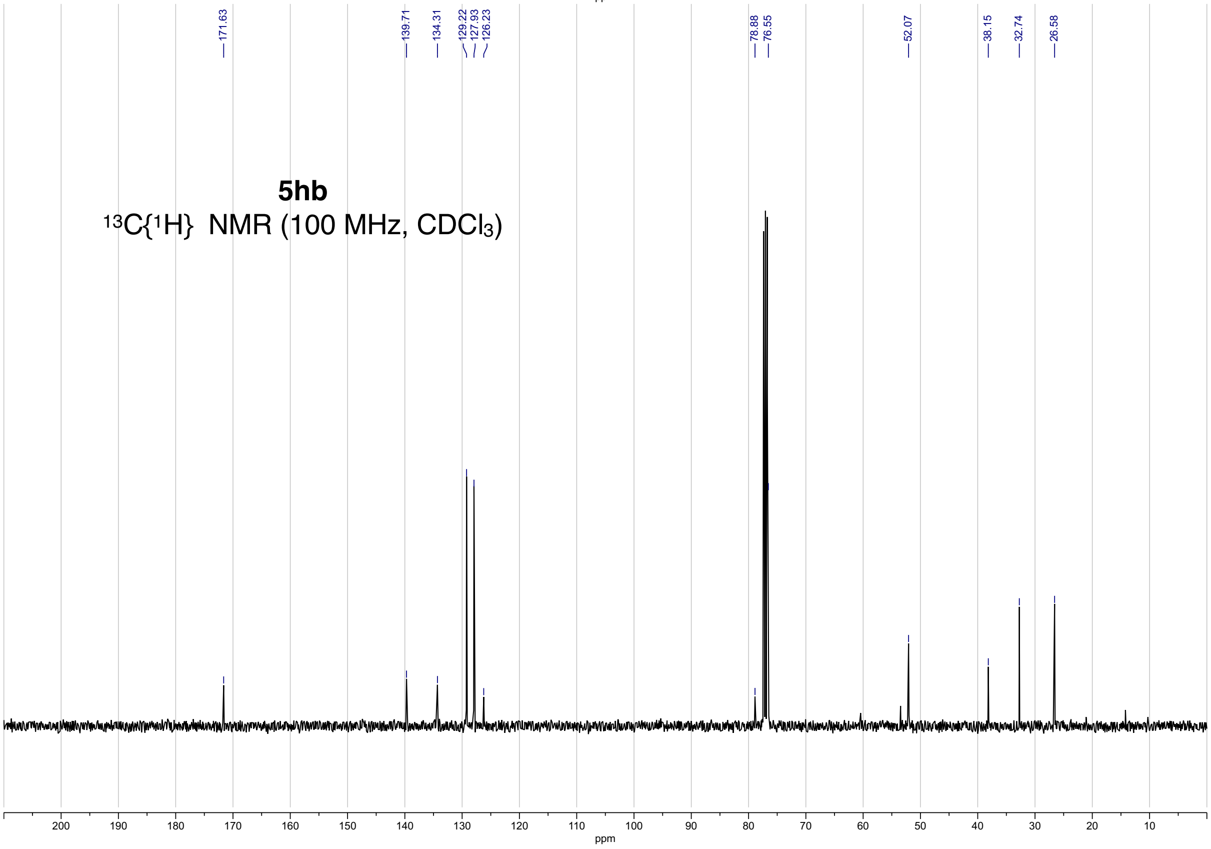

5hb
$^{13}C\{^{1}H\}$ NMR (100 MHz, $CDCl_3$)

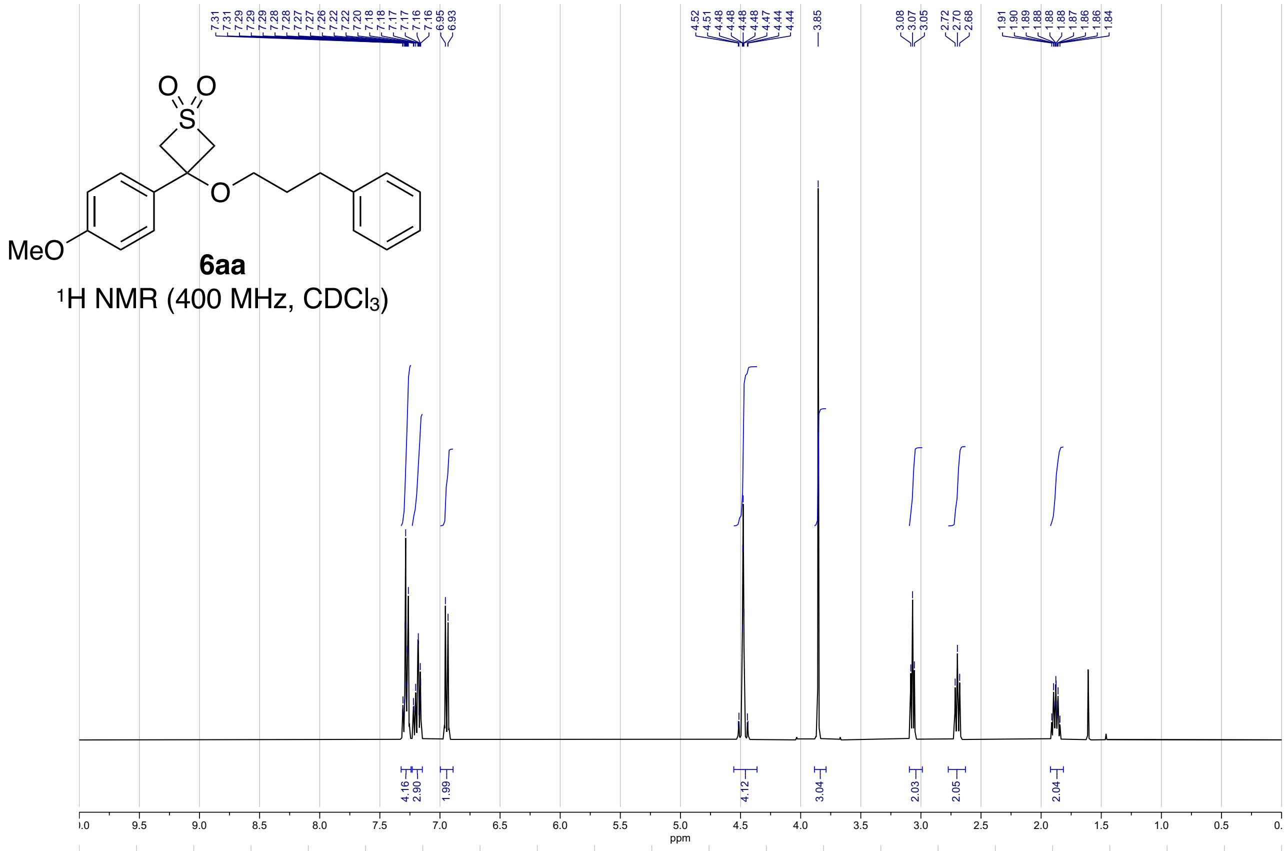
MeO
6aa
1H NMR (400 MHz, CDCl3)
ppm

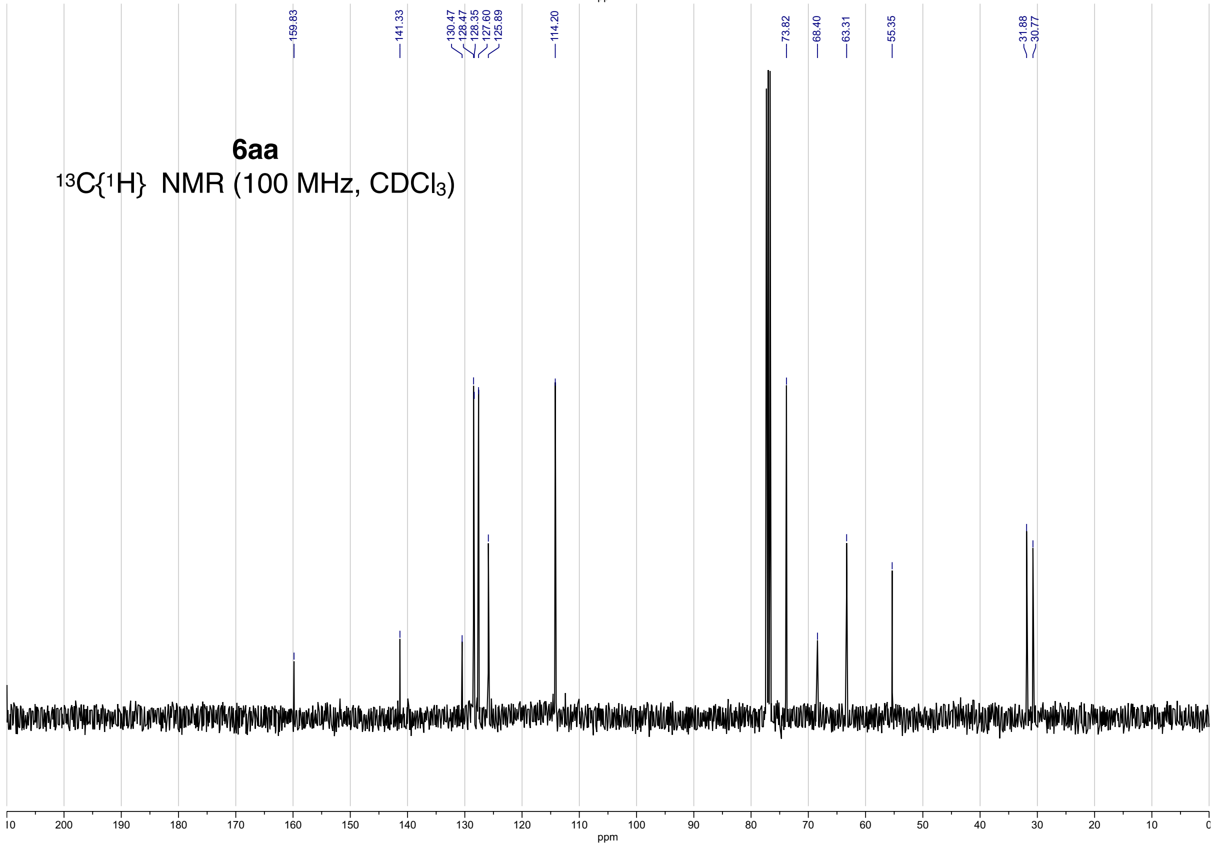
6aa
13C{1H} NMR (100 MHz, CDCl3)
ppm

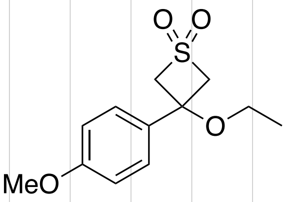

6ab

^{1}H NMR (400 MHz, $CDCl_3$)

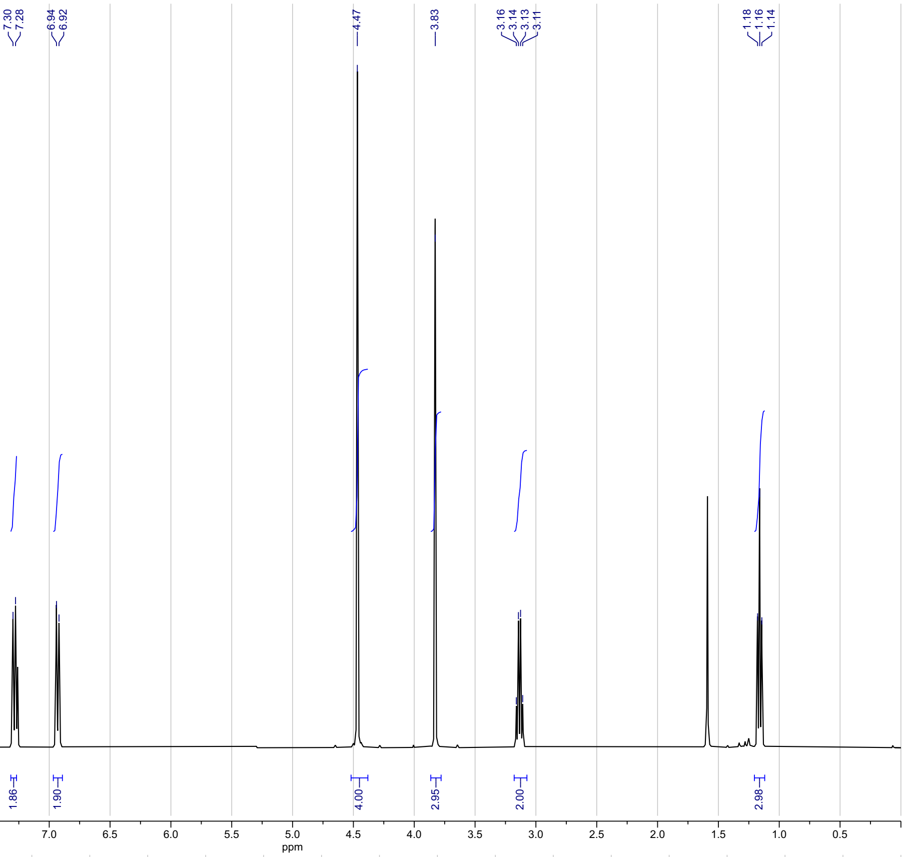

6ab

$^{13}C\{^1H\}$ NMR (100 MHz, $CDCl_3$)

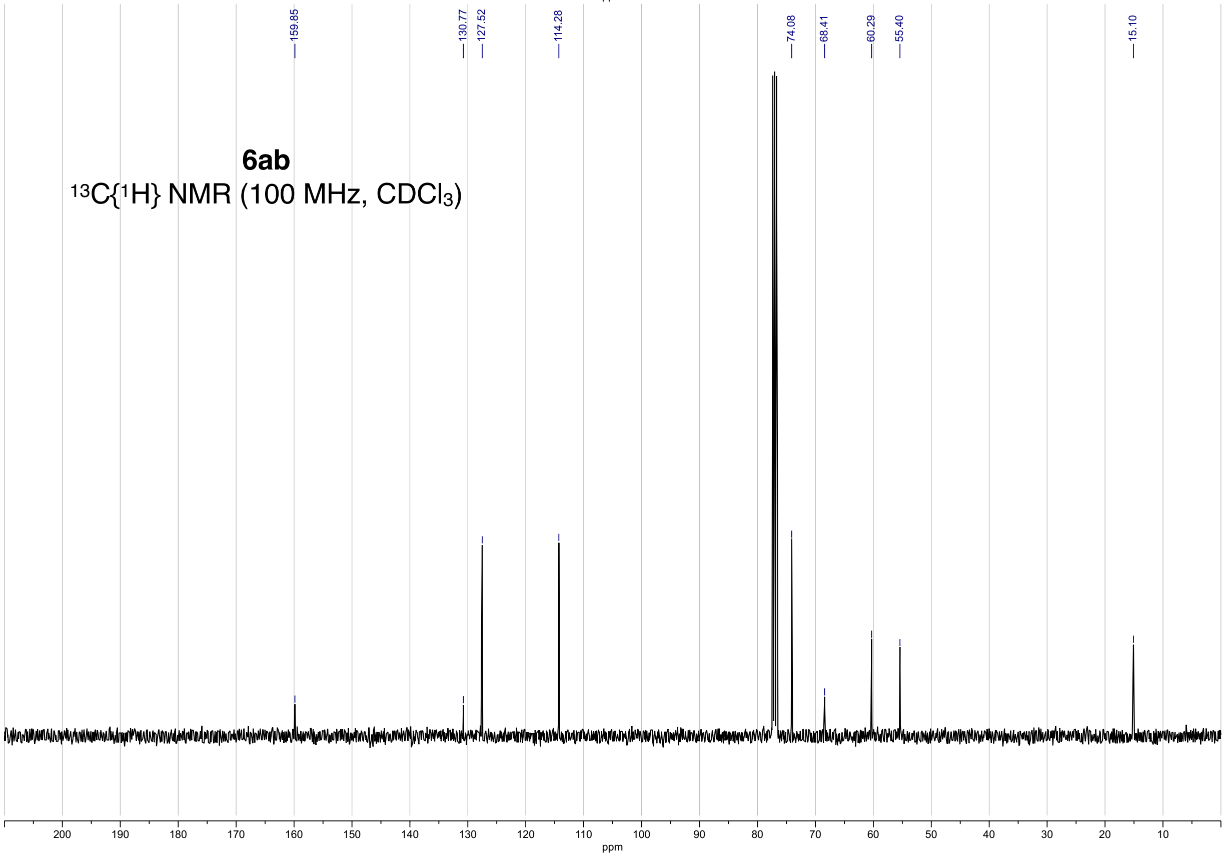

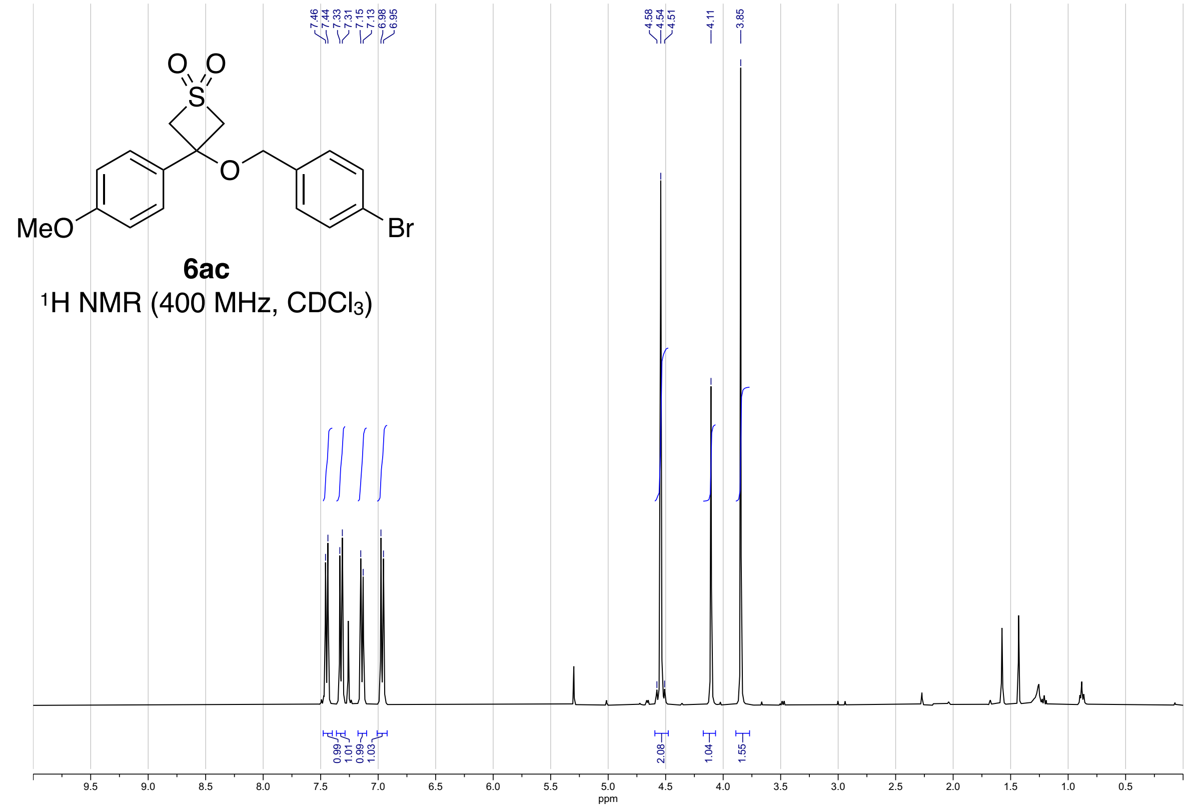
MeO
Br
6ac
1H NMR (400 MHz, CDCl3)
ppm

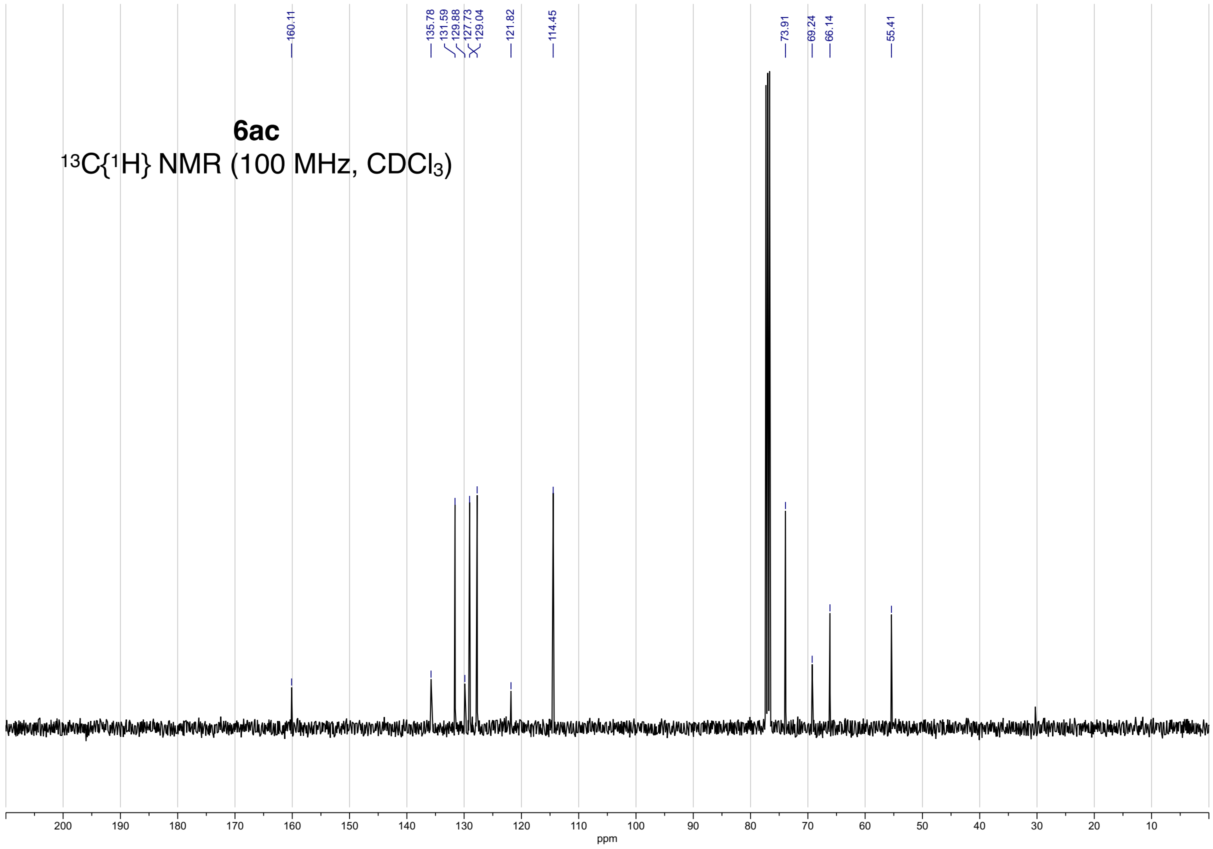
6ac
13C{1H} NMR (100 MHz, CDCl3)
160.11
135.78
131.59
129.88
127.73
129.04
121.82
114.45
73.91
69.24
66.14
55.41
ppm

7

^{1}H NMR (400 MHz, $CDCl_3$)

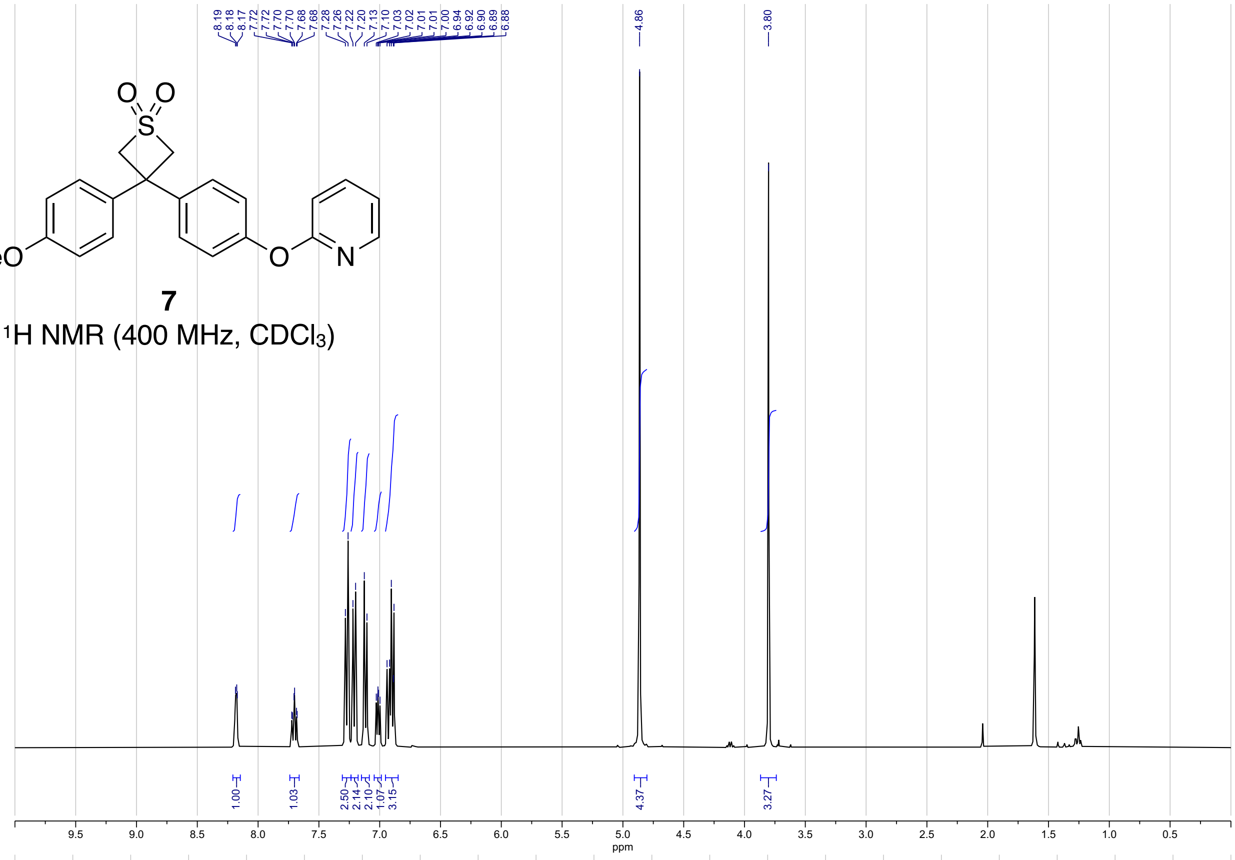

7

^{13}C{^{1}H} NMR (100 MHz, $CDCl_3$)

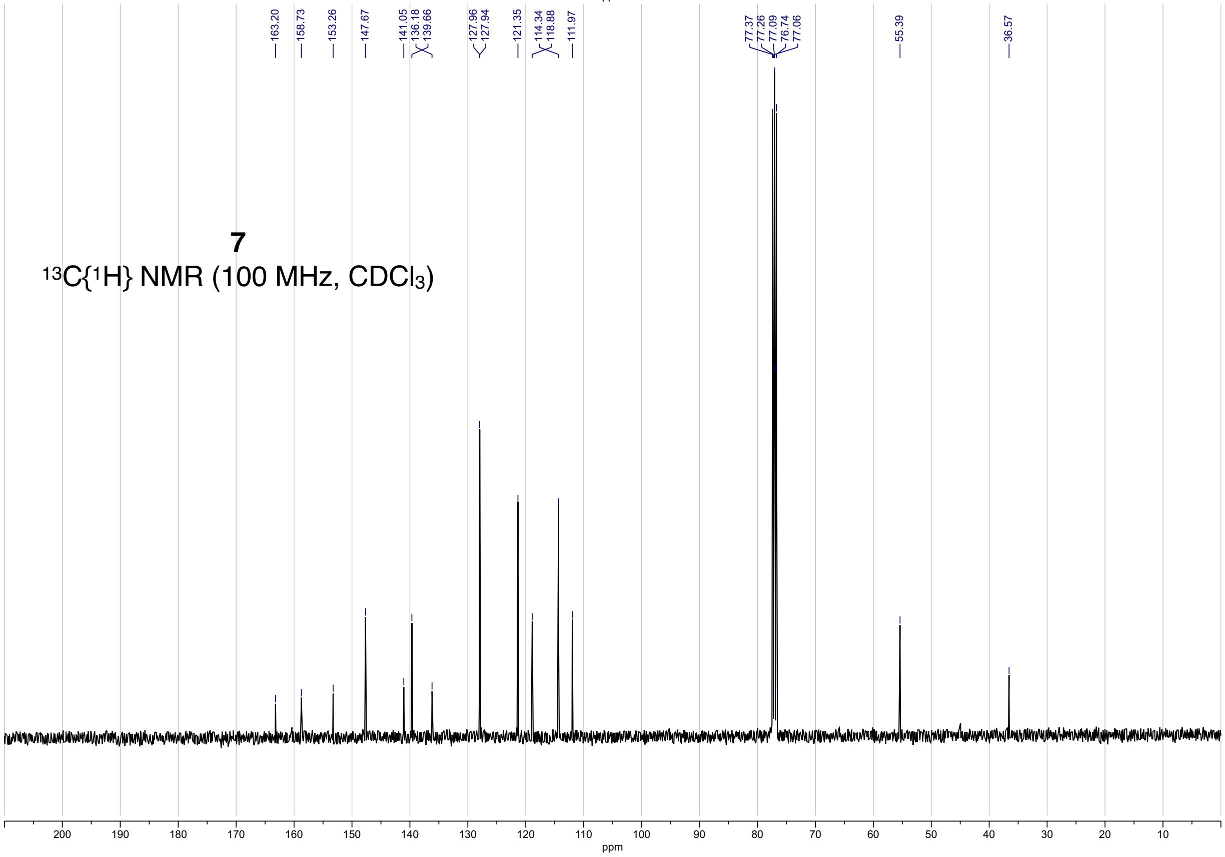

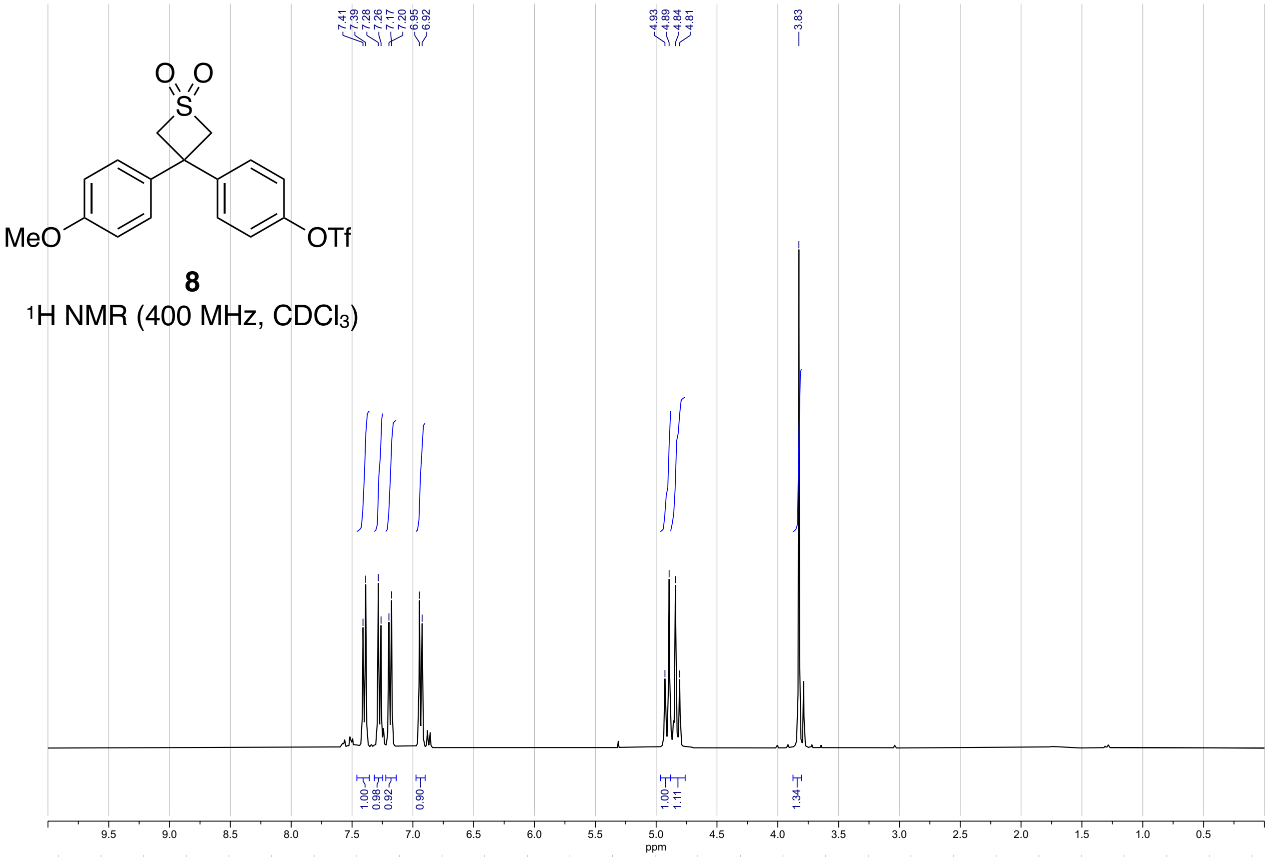
MeO
OTf
8
¹H NMR (400 MHz, CDCl₃)
7.41 7.39 7.28 7.26 7.20 7.17 6.95 6.92
4.93 4.89 4.84 4.81
3.83
1.00 0.98 0.92 0.90 1.00 1.11 1.34
ppm

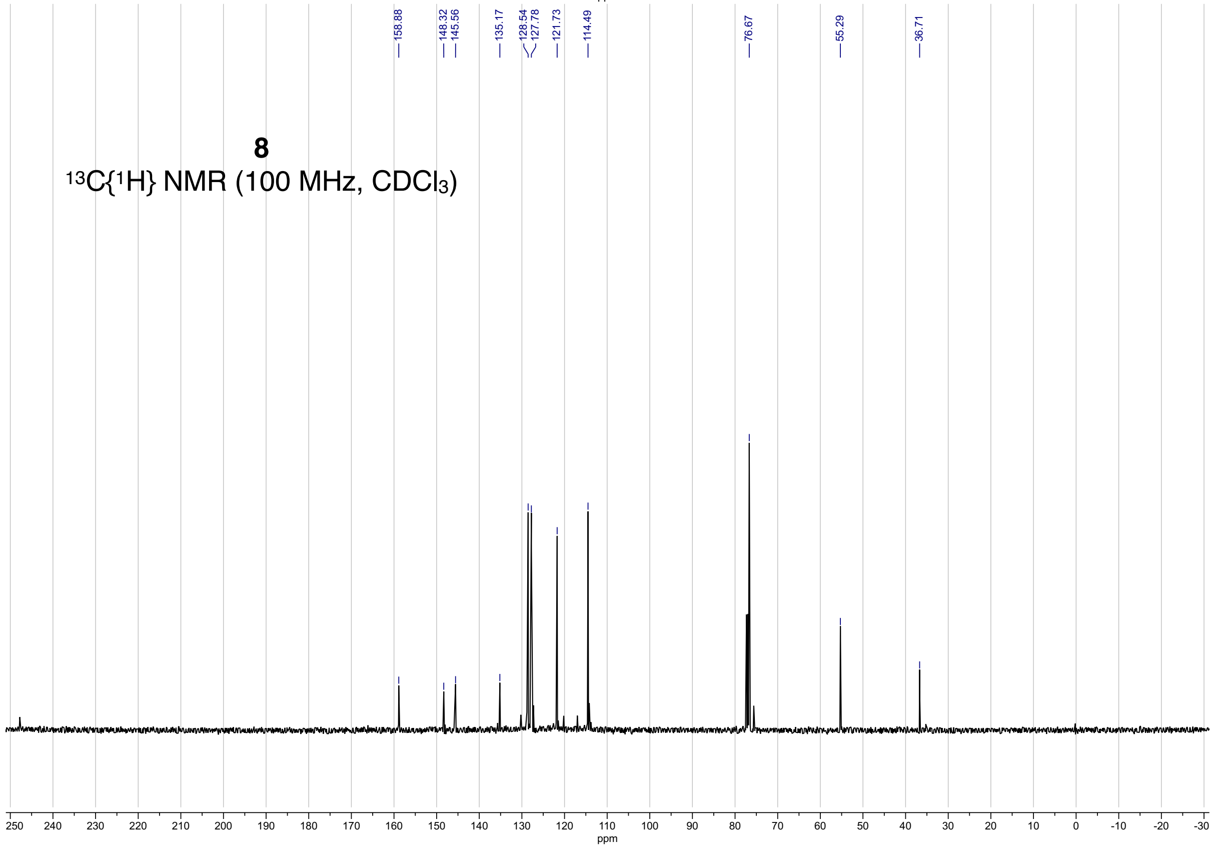
8
¹³C{¹H} NMR (100 MHz, CDCl₃)
158.88 148.32 145.56 135.17 128.54 127.78 121.73 114.49 76.67 55.29 36.71
ppm

8

^{19}F NMR (377 MHz, $CDCl_3$)

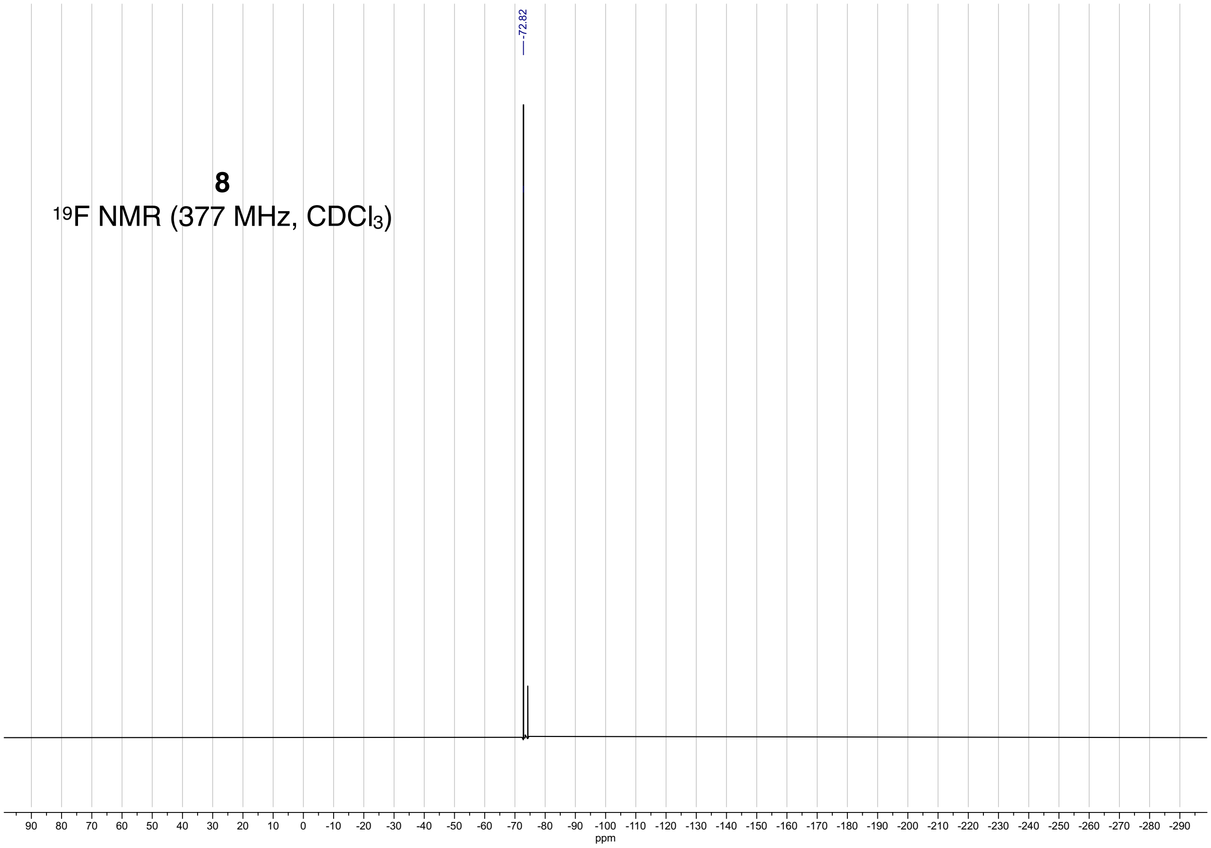

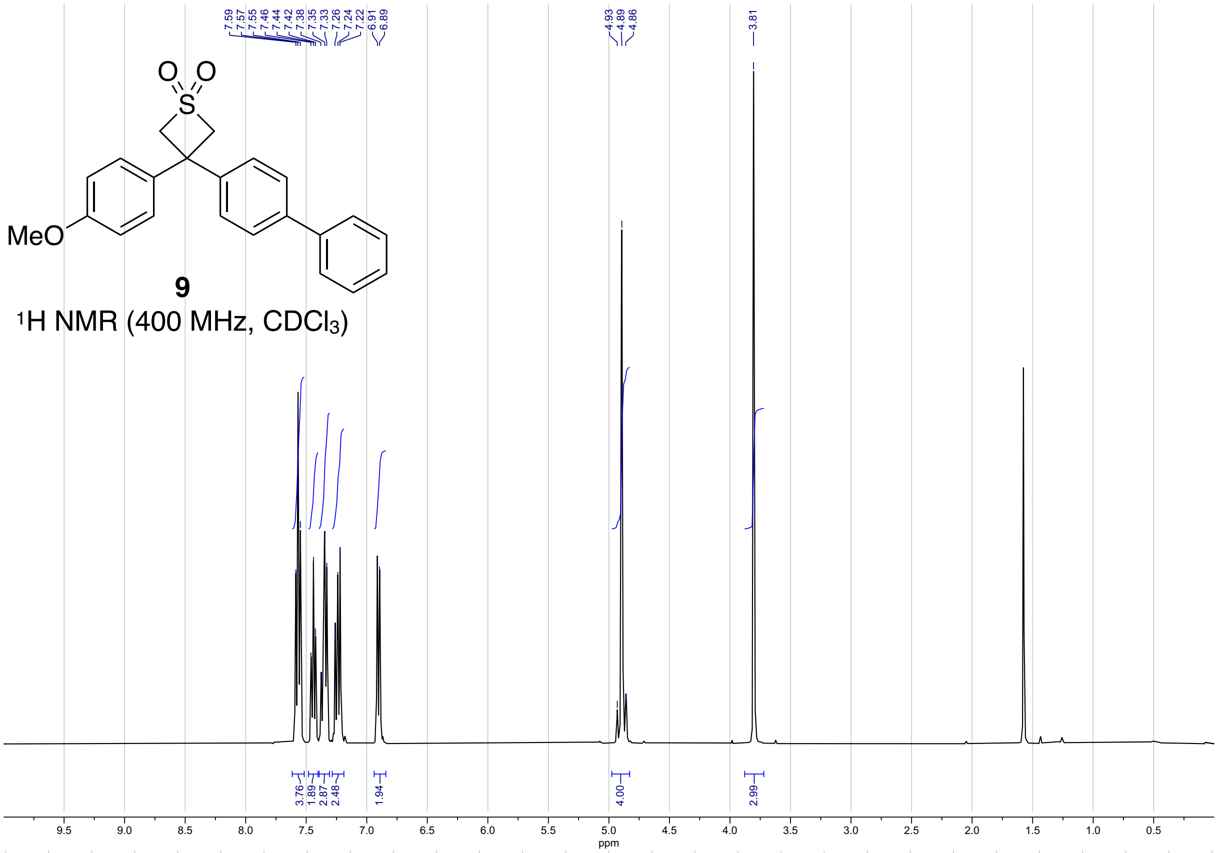
7.59
7.57
7.55
7.46
7.44
7.42
7.38
7.35
7.33
7.26
7.24
7.22
6.91
6.89
4.93
4.89
4.86
3.81
O
O
S
MeO
9
¹H NMR (400 MHz, CDCl₃)
3.76
1.89
2.87
2.48
1.94
4.00
2.99
ppm

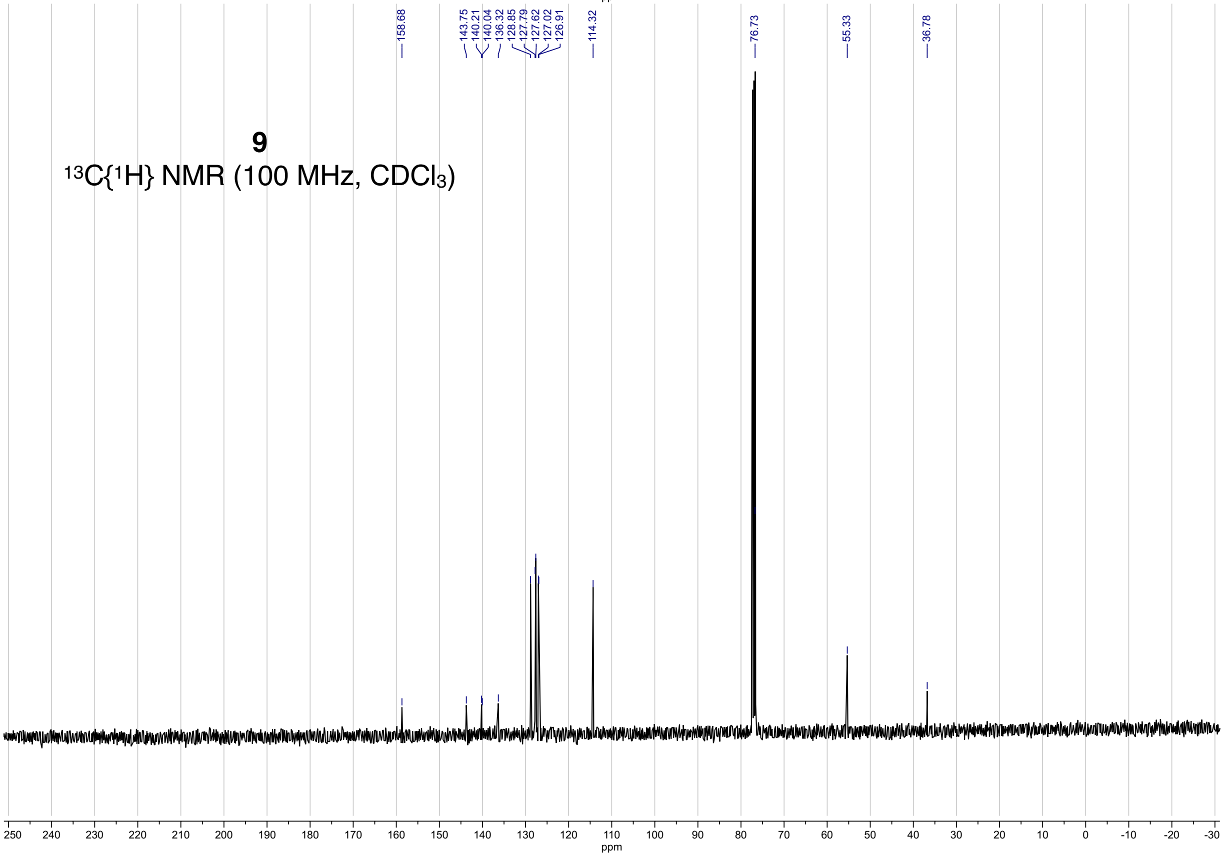
158.68
143.75
140.21
140.04
136.32
128.85
127.79
127.62
127.02
126.91
114.32
76.73
55.33
36.78
9
¹³C{¹H} NMR (100 MHz, CDCl₃)
ppm

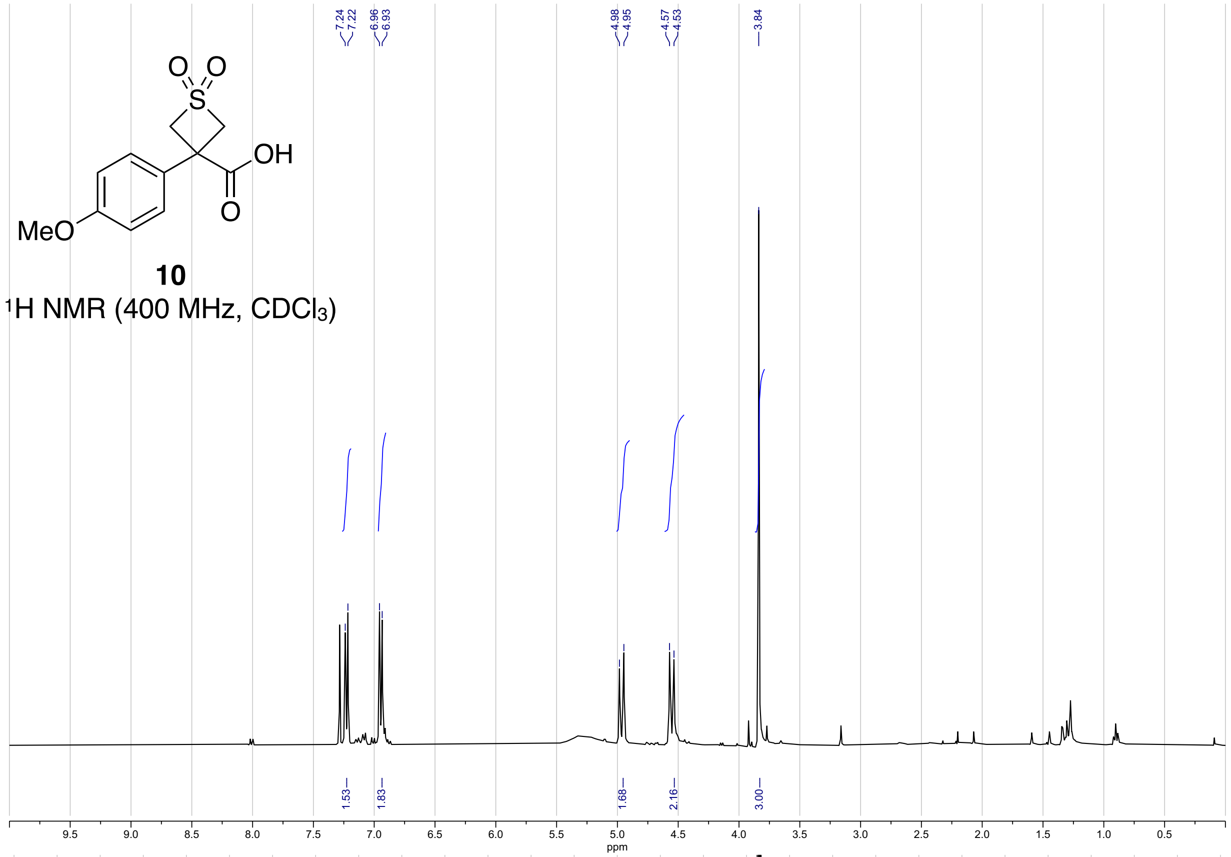

10
^{1}H NMR (400 MHz, $CDCl_3$)

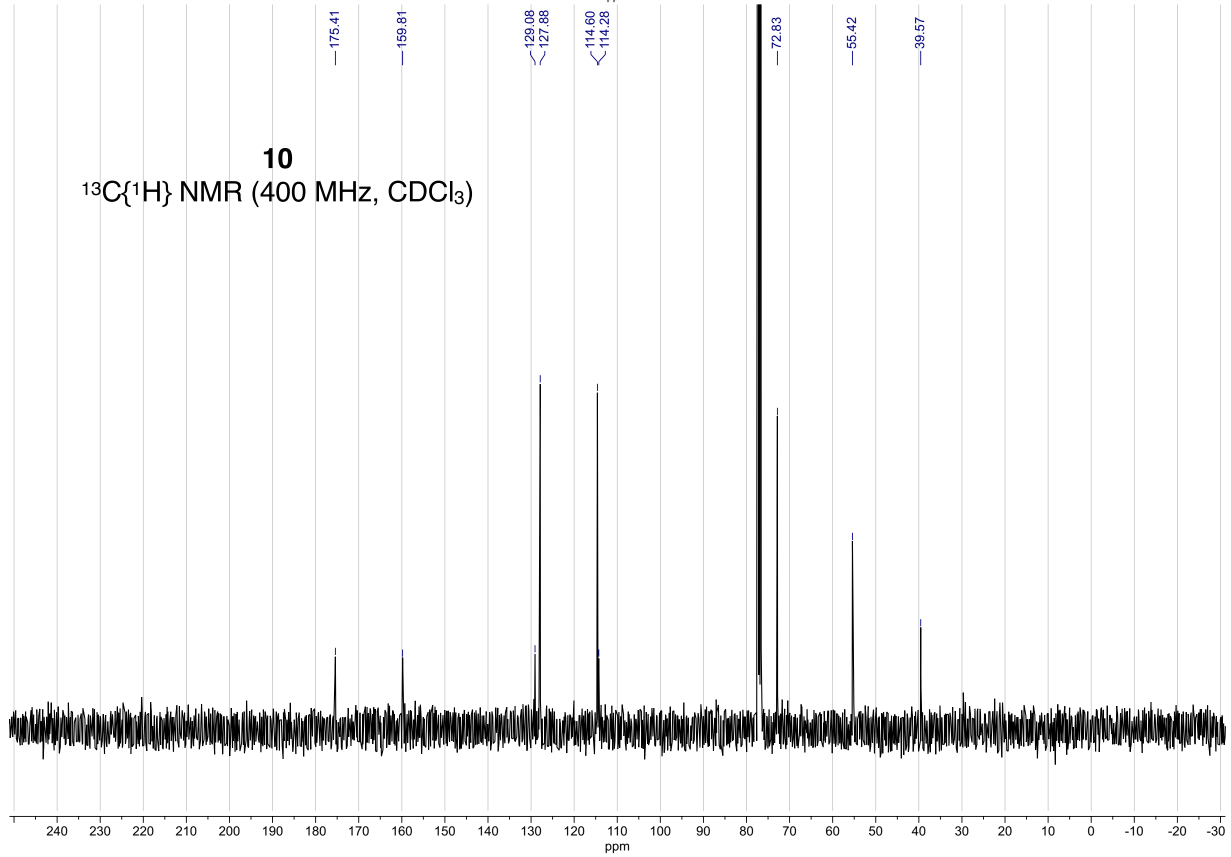

10
^{13}C{^{1}H} NMR (400 MHz, $CDCl_3$)

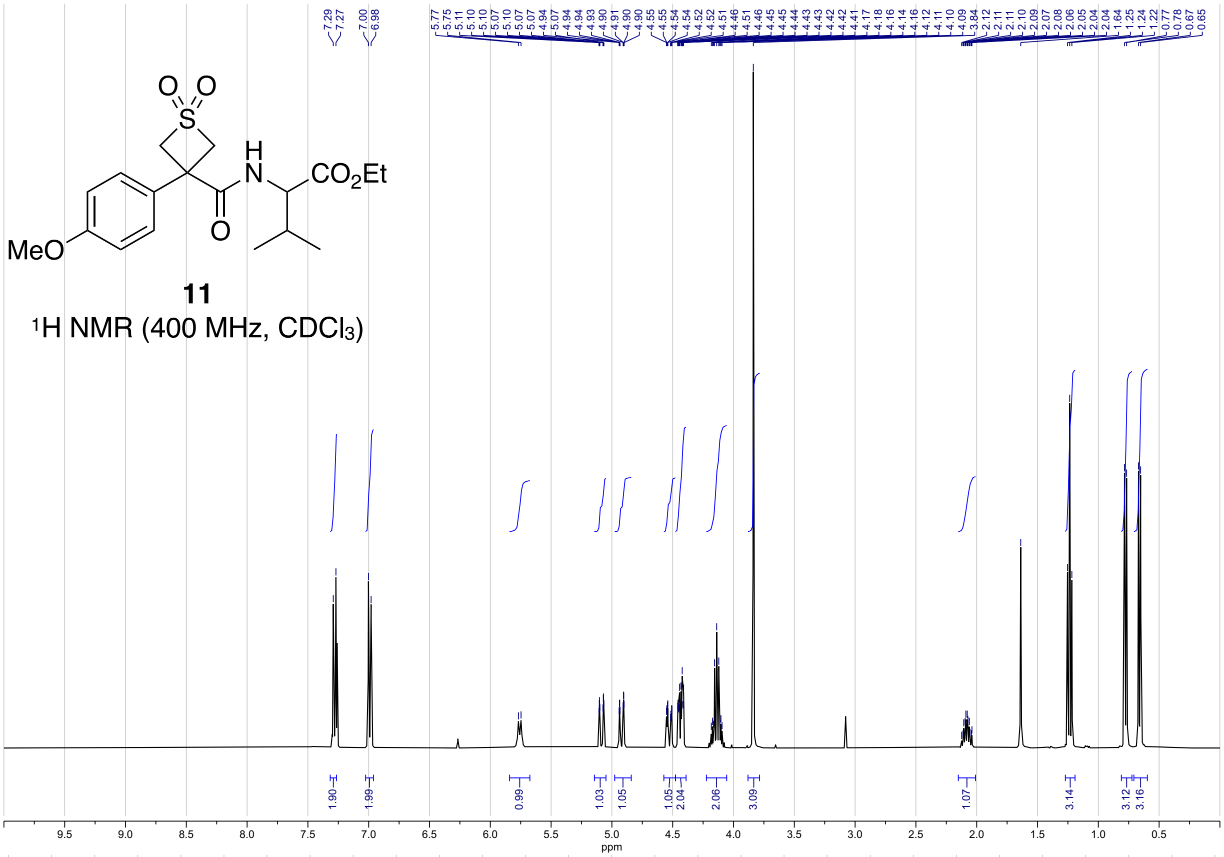
MeO
H
N
CO_2Et
O
S
O O
11
^{1}H NMR (400 MHz, $CDCl_3$)
1.90
1.99
0.99
1.03
1.05
1.05
2.04
2.06
3.09
1.07
3.14
3.12
3.16
9.5 9.0 8.5 8.0 7.5 7.0 6.5 6.0 5.5 5.0 4.5 4.0 3.5 3.0 2.5 2.0 1.5 1.0 0.5
ppm

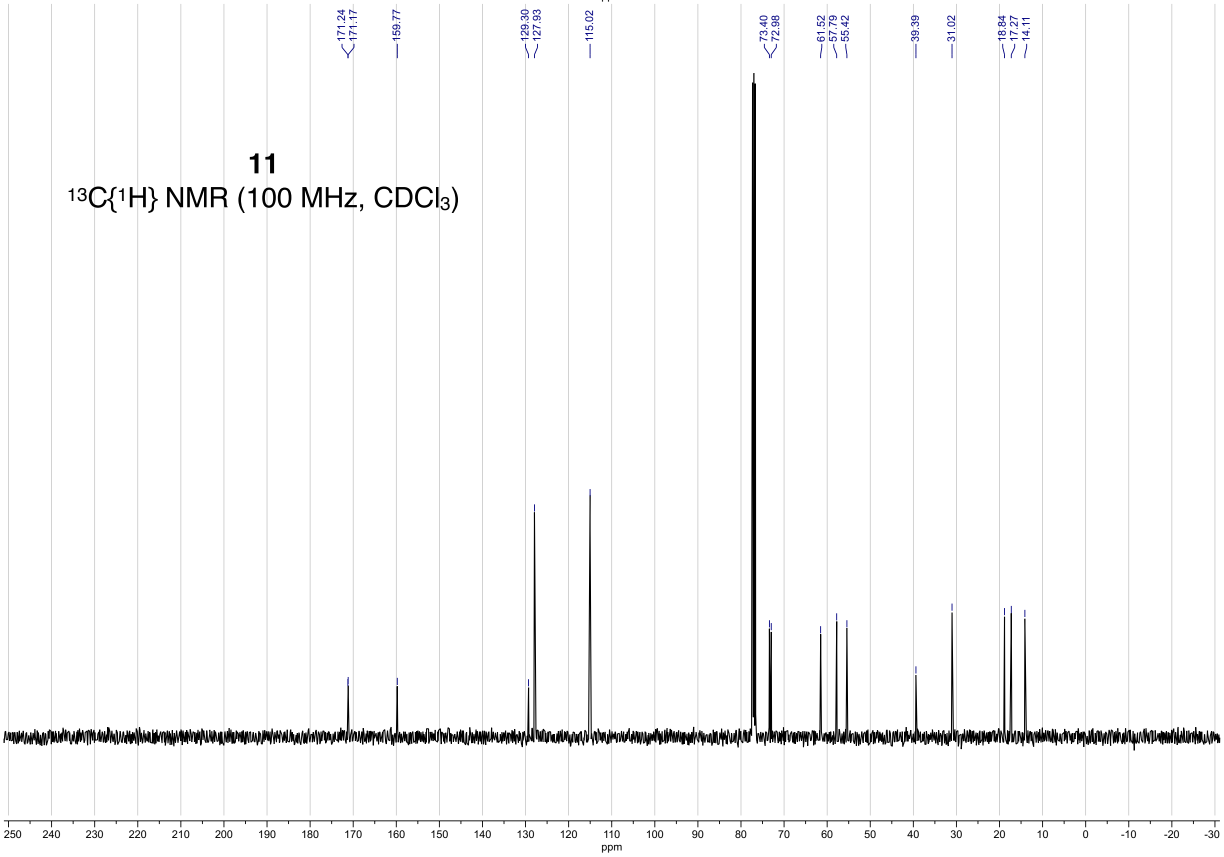
171.24
171.17
159.77
129.30
127.93
115.02
73.40
72.98
61.52
57.79
55.42
39.39
31.02
18.84
17.27
14.11
11
$^{13}C\{^1H\}$ NMR (100 MHz, $CDCl_3$)
250 240 230 220 210 200 190 180 170 160 150 140 130 120 110 100 90 80 70 60 50 40 30 20 10 0 -10 -20 -30
ppm

References

(1) Dolomanov, O. V.; Bourhis, L. J.; Howard, J. A. K; Puschmann, H. OLEX2:a Complete Structure Solution, Refinement and Analysis Program. *J. Appl. Cryst.* **2009**, *42*, 339–341.
(2) SHELXTL v5.1, Bruker AXS, Madison, *WI*, **1998.**
(3) SHELX-2013, G.M. Sheldrick, *Acta Cryst.*, **2015**, *C71*, 3–8.
